# Catalytic hydroboration of aldehydes and ketones with an electron-rich acyclic metallasilylene†

**DOI:** 10.1039/d3sc06842k

**Published:** 2024-02-12

**Authors:** Leon Kapp, Christoph Wölper, Hannah Siera, Gebhard Haberhauer, Stephan Schulz

**Affiliations:** a Institute for Inorganic Chemistry, University of Duisburg-Essen Universitätsstraße 5–7 45117 Essen Germany stephan.schulz@uni-due.de; b Institute for Organic Chemistry, University of Duisburg-Essen Universitätsstraße 5–7 45117 Essen Germany gebhard.haberhauer@uni-due.de; c Center for Nanointegration Duisburg-Essen (Cenide), University of Duisburg-Essen Carl-Be Germany

## Abstract

The application of main group metal complexes in catalytic reactions is of increasing interest. Here we show that the electron-rich, acyclic metallasilylene L′(Cl)GaSiL C (L′ = HC[C(Me)NDipp]_2_, Dipp = 2,6-^i^Pr_2_C_6_H_3_; L = PhC(N^*t*^Bu)_2_) acts as a precatalyst in the hydroboration of aldehydes with HBPin. Mechanistic studies with iso-valeraldehyde show that silylene C first reacts with the aldehyde with [2 + 1] cycloaddition in an oxidative addition to the oxasilirane 1, followed by formation of the alkoxysilylene LSiOCH[Ga(Cl)L′]CH_2_CHMe_2_ (2), whose formation formally results from a reductive elimination reaction at the Si center. Alkoxysilylene 2 represents the active hydroboration catalyst and shows the highest catalytic activity with *n*-hexanal (reaction time: 40 min, yield: >99%, TOF = 150 h^−1^) at room temperature with a catalytic load of only 1 mol%. Furthermore, the hydroboration reaction catalysed by alkoxysilylene 2 is a living reaction with good chemoselectivity. Quantum chemical calculations not only provide mechanistic insights into the formation of alkoxysilylene 2 but also show that two completely different hydroboration mechanisms are possible.

## Abstract 2

Catalytic hydroboration of aldehydes and ketones is achieved with alkoxysilylene 2. Quantum chemical calculations gave insights into the energetics of its formation and provide possible catalytic reaction mechanism.

## Graphical abstract

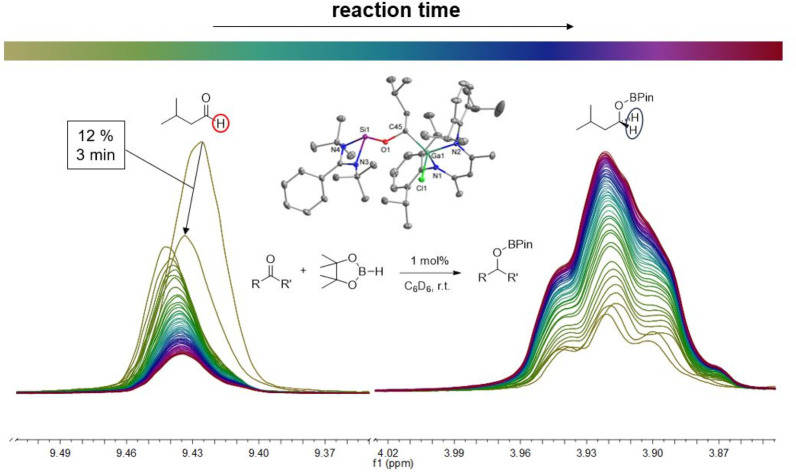

## Introduction

The addition of a boron–hydrogen bond to an unsaturated organic group, the so-called hydroboration reaction, has received increasing interest since its initial report by H. C. Brown in 1956.^1^ Substantial progress has been made with the development of new boranes such as pinacolborane (HBPin), catecholborane (HBcat),^2^ and Piers' borane (HB(C_6_F_5_)_2_),^3^ respectively, and a large number of active early and late transition metal catalysts have been established since the first reports of transition metal-catalysed hydroboration of alkynes and alkenes.^4^ Moreover, the concept of hidden boron catalysis in the hydroboration of alkynes and alkenes has recently been discussed.^5^ In marked contrast, the number of metal-free catalysts,^6^ which were first reported in 2012,^7^ as well as main group metal catalysts including s-block^8^ and p-block elements^9^ is still limited.

**Scheme 1:**
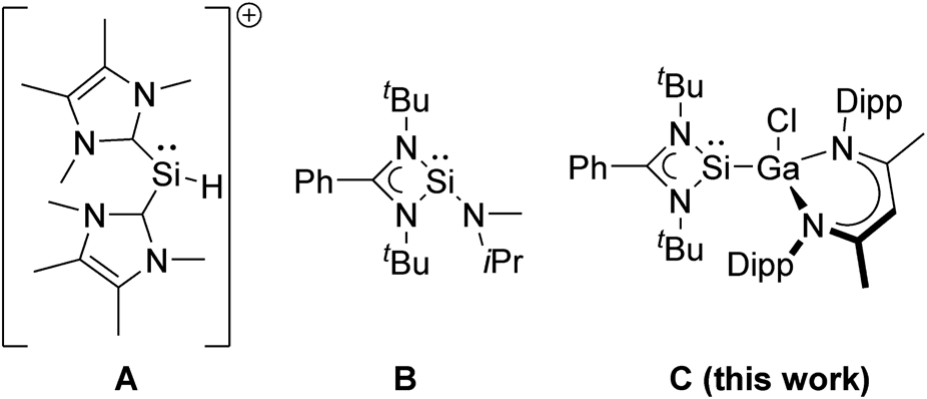
Silylenes applied in catalytic hydroboration reactions of carbonyl compounds.

The catalytic hydroboration of aldehydes often uses transition metal catalysts, which promote the addition of pinacolborane to aryl and alkyl aldehydes,^10^ but aldehyde hydroboration can be also performed without any catalyst.^11^ Hydroboration catalysts based on main group metals have only recently attracted increasing interest because they are often less expensive and less toxic than transition metal catalysts.^12–15^ In particular Al(iii)-based complexes have been investigated, and also silanes are known to hydroborate aldehydes (Scheme 1).^16–20^

Divalent tetrylenes are also of general interest in catalytic hydroboration transformations^21^ due to their Lewis ambiphilic behavior, which results from the presence of an electron lonepair and a vacant p-orbital, thus allowing transition metal-like reactivity such as oxidative addition reactions. While neutral and cationic germylenes,^22–27^ stannylenes,^27–29^ and plumbylenes^30^ have been successfully used in hydroboration catalysis, the catalytic activity of silylenes has been much less explored. Silylenes showed promising potential in (stoichiometric) small molecule activation reactions,^31,32^ including CO activation,^33–35^ but their catalytic activity is often limited by the use of sterically demanding substituents (kinetic stabilisation)^36^ and only a few silylene hydroboration catalysts are known, to date. The NHC-coordinated silyliumylidene cation [(IMe)_2_SiH]^+^ (IMe=:C{N(Me)C(Me)}_2_) A has been reported to catalyse the hydroboration of CO_2_ and carbonyl compounds including benzaldehyde PhC(O)H and derivatives with electron-donating and electron-withdrawing substituents.^37,38^ Moderate activities (TOF 17–115.8 h^−1^) were observed with rather high catalyst loading (10 mol%) at room temperature, which are intermediate between those reported for amido(hydrido)germylene (17–67 h^−1^) and -stannylene (400–800 h^−1^) [Ar*(^i^Pr_3_Si)NMH] (M = Ge, Sn; Ar* = 4-^i^Pr-2,6-(CHPh_2_)_2_C_6_H_2_), respectively.^39^ In addition, silylene B was found to catalyse the chemoselective hydroboration of aldehydes and ketones with HBPin at high temperature (90 °C) and high catalyst loading (5 mol%).^40^

Unfortunately, mechanistic studies of catalytic hydroboration reactions of carbonyl compounds using tetrylenes are also very rare. The hydridogermylene [Ar*(^i^Pr_3_Si)NGeH] was reported to react *via* Ge–H and B–H σ-bond metathesis reaction,^39^ while the phosphine-substituted tetrylenes [Ar^iPr^EC(H)(Ph)PPh_2_] (E = Ge, Sn; Ar^iPr^ = 2,6-(2,4,6-^i^Pr_3_C_6_H_2_)_2_C_6_H_3_) either activate the aldehyde by adduct formation with the phosphine donor and the tetrylene acceptor, respectively, or activate pinacolborane (HBPin) at the tetrylene center.^30^ The diaminogermylene-mediated catalytic hydroboration of aldehydes occurred *via* adduct formation between Lewis basic HBPin and the Lewis acidic Ge(ii) center, which facilitates the insertion of the aldehyde into the B–H bond.^41^

Here we report the catalytic hydroboration of aldehydes and ketones with HBPin using the acyclic, electron-rich metallasilylene L′(Cl)GaSiL C.^42^ Silylene C serves as precatalyst, which initially reacts with the aldehyde at low temperature in a [2 + 1] cycloaddition reaction to form oxasilirane 1. Upon heating to room temperature, oxasilirane 1 rearranges to form the active catalyst LSiOCH[Ga(Cl)L′]CH_2_CHMe_2_2. Quantum chemical calculations provide deeper insights into the mechanism and energetics of the reactions. Alkoxysilylene 2 is a chemoselective catalyst that combines the Lewis acidity of group 13 complexes with the Lewis basicity of silylenes.

## Results and discussion

### Synthetic procedures

We first tested the catalytic activity of gallasilylene C in the hydroboration of various aldehydes with HBPin at room temperature. C did not react with HBPin in the absence of any aldehydes according to *in situ*^1^H and ^11^B NMR studies (Fig. S26 and S27†), whereas amidinate-substituted silylenes were reported to undergo cooperative B–H bond activation of HBPin^43^ In contrast, C was found to hydroborate iso-valeraldehyde, benzaldehyde, 4-hexylbenzaldehyde and 3-bromobenzaldehyde, which was accompanied by an immediate color change of the solution from yellow to colorless, whereas no reaction occurred with *n*-heptanal, 4-nitrobenzaldehyde, and 4-hydroxybenzaldehyde under identical reaction conditions. Obviously, the catalytic activity of gallasilylene C strongly depends on the electronic/steric properties of the aldehyde.

**Scheme 2:**
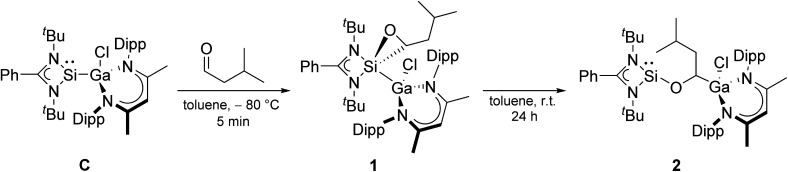
Reaction of silylene C with one equivalent of iso-valeraldehyde (Dipp = 2,6-^i^Pr_2_C_6_H_3_).

To obtain more detailed information about the initial step of the reactions and the true nature of the catalytically active species, we studied the equimolar reaction of compound C with iso-valeraldehyde at low temperature (−80 °C). The reaction proceeded by [2 + 1] cycloaddition (oxidative addition) of the aldehyde to the silylene center to give the oxasilirane 1, followed by Si–C bond cleavage and Ga–C bond formation upon warming to room temperature and formation of the alkoxysilylene LSiOCH[Ga(Cl)L′]CH_2_CHMe_2_2 (Scheme 2). The conversion of oxasilirane 1, in which the Si atom adopts the formal oxidation state of +IV, into the alkoxysilylene 2 formally represents a reductive elimination reaction at the Si center. Even more remarkable is that this reaction starts at –5 °C according to *in situ*^1^H NMR studies. Moreover, comparable to our findings with gallasilylene C, alkoxysilylene 2 also did not react with HBPin in the absence of any aldehydes according to *in situ*^1^H and ^11^B NMR studies (Fig. S28 and S29†).

The ^1^H and ^13^C NMR spectra of oxasilirane 1 and alkoxysilylene 2 show the expected resonances of the organic ligands as well as those for the aldehyde H atom (^1^H NMR: 1: 2.18 ppm, 2: 4.33 ppm) and the carbonyl C atom (^13^C NMR: 1: 59.20 ppm, 2: 68.31 ppm), respectively. The ^29^Si NMR spectrum of oxasilirane 1 shows a resonance at −112.79 ppm, which is consistent with the chemical shifts of known oxasiliranes (−99 to −123 ppm),^44–47^ while the ^29^Si chemical shift of alkoxysilylene 2 (−12.59 ppm) is in the typical range of alkoxy-^48^ and siloxysilylenes,^49^ respectively.

### Crystallographic studies

Single crystals suitable for X-ray diffraction were obtained from saturated solutions in benzene after storage at 4 °C (1) for only 10 min and in *n*-hexane after storage at 25 °C (2), respectively. 1 and 2 crystallize in the triclinic space group *P*1̄ with one molecule (accompanied by solvent) in the asymmetric unit (Fig. 2). The SiNCN units in oxasilirane 1 and alkoxysilylene 2 adopt almost planar conformations, in contrast to the boat-type conformations of the GaN_2_C_3_ ring. The Si–Ga bond length in oxasilirane 1 (2.4054(5) Å) fits to the sum of the covalence radii (Si–Ga 2.40 Å),^50^ but is significantly shorter than that in metallasilylene C (2.5170(4) Å) despite the higher coordination number at the Si atom (five *versus* three). The Si–O (1.7488(16) Å) and Si–C (1.835(3) Å) bond lengths in oxasilirane 1 largely differ from those of known oxasiliranes, which range from 1.6486(13) Å to 1.6520(10) Å (Si–O) and 1.8834(15) Å to 1.8924(18) Å (Si–C),^45–48^ respectively. The O–Si–C bond angle of oxasilirane 1 (49.52(11)°) is almost identical with those of known oxasiliranes (50.70(6)°, 50.31(6)°),^44,45^ while the Si–O–C bond angle of oxasilirane 1 (68.23(12)°) is slightly smaller than those of other oxasiliranes (70.42–73.18°).^44,51^ The N–Si–N bond angle of oxasilirane 1 (68.68(5)°) is consistent with that of amidinate-substituted silylenes,^40,52^ and the N–Ga–N bond angle of oxasilirane 1 (96.68(5)°) is slightly larger than that of compound C (92.65(4)°). The Ga atoms adopt distorted tetrahedral coordination geometries in both compounds, whereas the silicon atom in 1 is five-coordinated and in 2 three-coordinated, resulting in a much larger sum of bond angles at Si1 in 1 (N3–Si1–N4 + N3/4–Si1–Ga1 = 297.7(4)°) compared to 2 (N3–Si1–N4 + N3/4–Si1–O1 = 269.25(12)°). The Si–O bond length in alkoxysilylene 2 (1.6849(8) Å) is shorter than that of oxasilirane 1 (1.7488(16) Å), but in the range of the calculated Si–O single bond radius.^50^ In addition, the C–O bond of alkoxysilylene 2 (1.4390(12) Å) is much shorter than that of oxasilirane 1 (1.503(3) Å) and has a typical C–O single bond length (1.43 Å).^53^ The Ga–C bond length of 1.9662(10) Å is consistent with the sum of the covalence radii for Ga–C single bonds (1.967 Å).^50^

### Computational studies – reaction energies

Quantum chemical calculations (PBE0-D3BJ)^54,55^ were performed to investigate the energetics of the reactions in more detail (see ESI†). However, to simplify the calculations, *n*-propanal was used instead of iso-valeraldehyde.

**Fig. 1:**
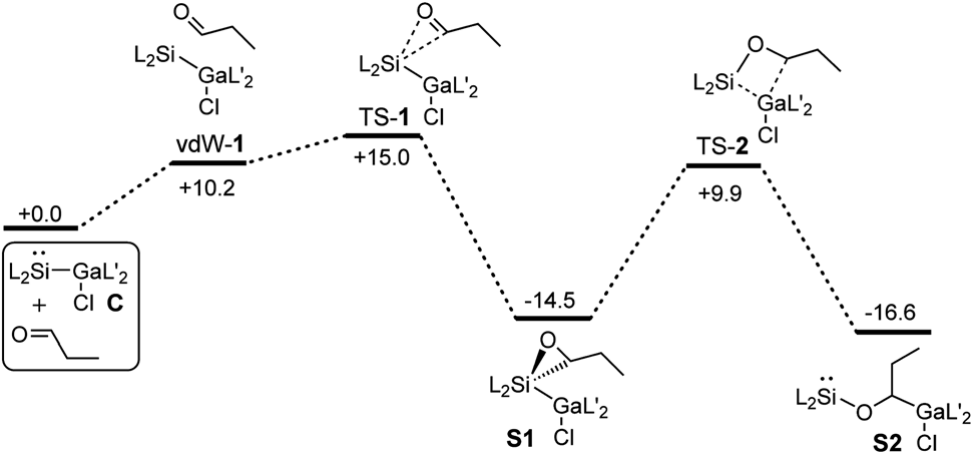
Gibbs energies for the formation of the catalyst S2 starting from C and *n*-propanal calculated by means of PBE0-D3BJ. The values are given in kcal mol^−1^.

The calculated Gibbs energies for the formation of the catalyst S2 starting from C and propanal are shown in Fig. 1. The first step is the formation of oxasilirane S1, which must pass an activation barrier of 15.0 kcal mol^−1^. The ring opening leads to the energetically more favorable silylene S2. The activation energy for this step (24.4 kcal mol^−1^) is much higher than that of the first step, allowing the isolation of S1 (or oxasilirane 1 if iso-valeraldehyde is used). Overall, this results in an insertion of the aldehyde into the Si–Ga bond of compound C. An alternative opening of the three-membered ring in S1, in which the oxygen is bound to the gallium atom, is also feasible. The activation energy for such an opening is even lower, but the product formed is destabilised compared to S1 and S2 (Fig. S36 and S37†).

**Fig. 2:**
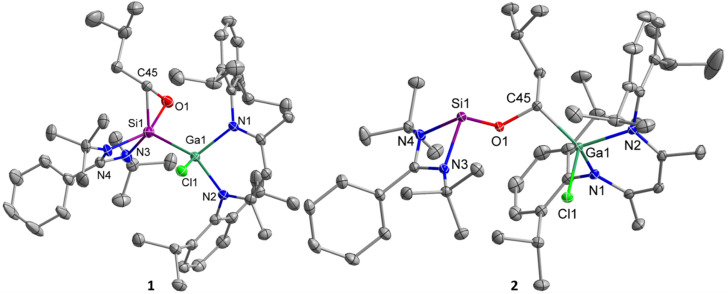
Structures of silylenes 1 and 2 in the solid state with thermal ellipsoids at 50% probability level. Solvent molecules hydrogen atoms and minor components of the disorder were omitted for clarity (RESI card were used to facilitate the refinement of 1. Residues 4 and 6 are displayed and their data given. For more details see ESI†). Selected bond lengths [Å] and angles [°]: 1: Ga(1)–N(2) 1.9614(14), Ga(1)–N(1) 1.9823(13), Ga(1)–Cl(1) 2.2845(4), Ga(1)–Si(1) 2.4054(5), Si(1)–C(45) 1.835(3), Si(1)–O(1) 1.7488(16), O(1)–C(45) 1.503(3), Si(1)–N(3) 1.818(7), Si(1)–N(4) 1.939(4), N(1)–Ga(1)–N(2) 94.20(6), O(1)–Si(1)–C(45) 49.52(11), Si(1)–O(1)–C(45) 68.23(12), O(1)–C(45)–Si(1) 62.25(12), N(3)–Si(1)–N(4) 69.17(18), O(1)–Si(1)–Ga(1) 103.62(6), C(45)–Si(1)–Ga(1) 114.48(9); 2: Ga(1)–N(1) 1.9444(9), Ga(1)–N(2) 1.9500(9), Ga(1)–C(45) 1.9662(10), Ga(1)–Cl(1) 2.2347(3), Si(1)–O(1) 1.6849(8), Si(1)–N(4) 1.8748(9), Si(1)–N(3) 1.9203(9), O(1)–C(45) 1.4390(12), N(1)–Ga(1)–N(2) 96.05(4), N(4)–Si(1)–N(3) 69.06(4), C(45)–O(1)–Si(1) 121.71(6).

### Catalytic studies

The isolated alkoxysilylene 2 was then used in the catalytic hydroboration of aldehydes and ketones with HBPin at room temperature and 1 mol% catalyst loading (Table 1). Moderate catalytic activities were observed with benzaldehyde (TOF 3.9 h^−1^), while electron withdrawing groups in the *para*-position were found to have a beneficial catalytic effect most likely due to an increase of the electrophilic nature of the carbonyl carbon atom of these aromatic aldehydes (TOF: 4-nitrobenzaldehyde 6.25 h^−1^; methyl-4-formylbenzoate 5.6 h^−1^). However, much higher catalytic activities were observed in the hydroboration of linear aliphatic aldehydes. The highest activities were found in the hydroboration of *n*-hexanal (TOF: 150 h^−1^) and *n*-propanal (TOF: 133 h^−1^), while the hydroboration of *n*-octanal is slower (TOF: 29 h^−1^). Studies with *n*-pentanal (TOF: 44 h^−1^) and iso-valeraldehyde (TOF: 11.1 h^−1^) show that the reaction time decreases with increasing steric demand of the aldehyde, however, the steady trend was not observed in the linear aliphatic aldehydes. In comparison, the hydroboration of ketones required longer reaction times under identical reaction conditions (TOF: 0.33–0.47 h^−1^), which is consistent with the lower reactivity of ketones in catalytic hydroboration reactions.^56^

**Table 1:** Aldehydes and ketones applied in catalytic hydroboration reactions with silylene 2 and HBPin

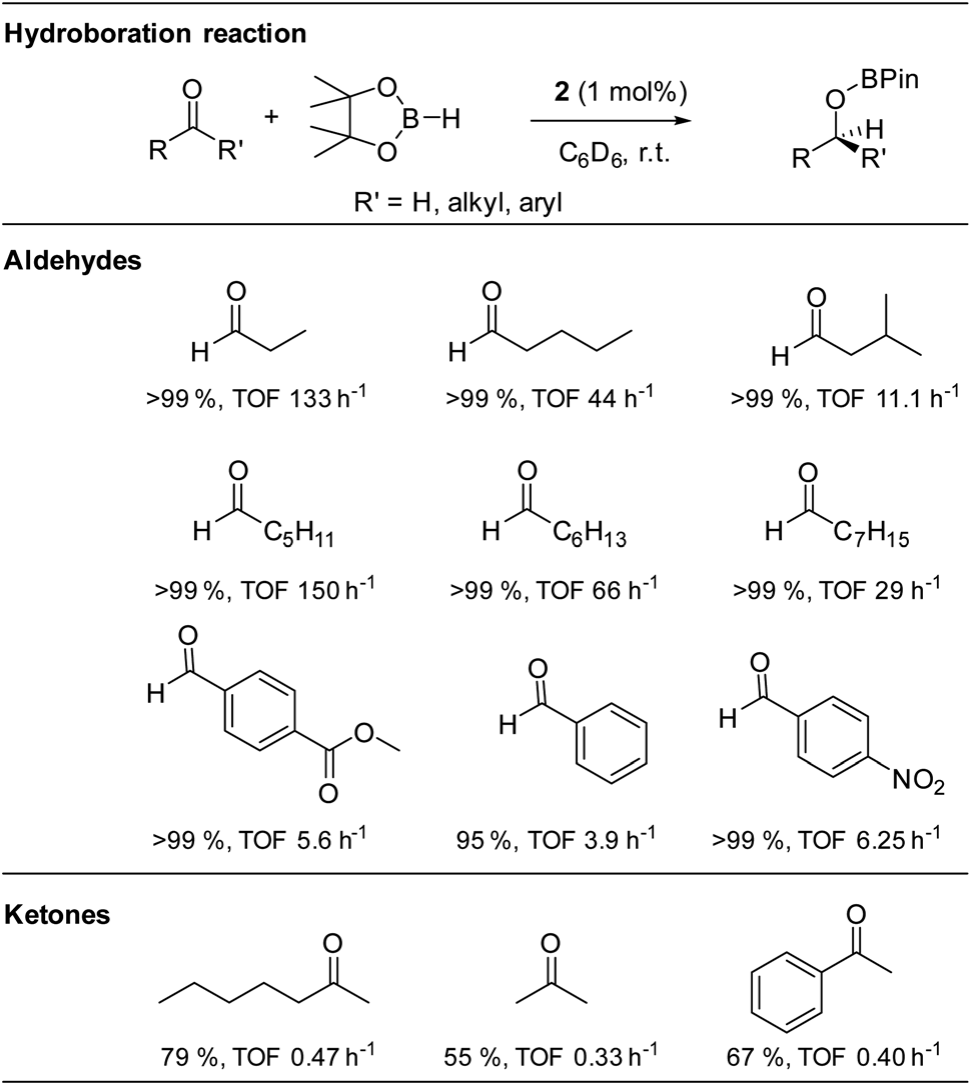

Compared to the silyliumylidene cation A, for which TOF-values in the range of 179 to 115.8 h^−1^ were reported, alkoxysilylene 2 was found to catalyse the hydroboration of aldehydes in a similar range of TOF values (TOF: 3.9–150 h^−1^), but with much lower catalyst loading (1 mol%) compared to A (10 mol%).^37^ In addition, the alkoxysilylene 2 showed a much higher catalytic activity in the hydroboration of aldehydes compared to the amidinate-substituted silylene B (TOF: 0.2–19.8 h^−1^) which was only active at high temperature (90 °C) and high catalyst loading (5 mol%).^40^ However, the alkoxysilylene 2 is catalytically less active compared to the heavier tetrylenes, *i.e.*, the hydridogermylene [Ar*(^i^Pr_3_Si)NGeH] (TOF: 179–6000 h^−1^, catalyst loading 0.05–1 mol%) and hydridostannylene Ar*(^i^Pr_3_Si)NSnH (TOF: 400–13 300 h^−1^, catalyst loading 0.05 mol%), respectively.^39^

To identify the active catalyst, the catalytic reaction of benzaldehyde, HBPin and the alkoxysilylene 2 (10 mol%) was performed at room temperature in the presence of two equivalents of TMEDA. Full conversion was observed after 16 h according to *in situ*^1^H NMR spectroscopic studies (Fig. S22†), which proved that the catalytic reaction occurred without the formation of borane BH_3_, proving that the alkoxysilylene 2 is the active catalyst in the reaction.^5^ To rule out a catalyst-free hydroboration reaction,^11^ we also reacted *n*-propanal with HBPin in the absence of catalyst 2. Only 76% (TOF: 1.58 h^−1^) of the aldehyde were converted into the corresponding borate ester after 48 h, whereas the reaction in the presence of alkoxysilylene 2 (1 mol%) was finished after 45 min (TOF: 133 h^−1^). Since we also did not observe any induction period in the catalysed reaction, we assume that 2 is the active catalyst.

We also became interested in its chemoselectivity and reacted 2 (1 mol%, r.t.) with a mixture of equimolar amounts of acetophenone, iso-valeraldehyde and HBPin. The resulting ^1^H NMR spectrum showed almost complete conversion of HBPin after 24 h. 94% conversion of the aldehyde and only 5% conversion of the ketone to their corresponding borane esters (Fig. S24†) clearly demonstrated the good chemoselectivity of catalyst 2 for aldehydes over ketones. In addition, alkoxysilylene 2 was shown to be a living hydroboration catalyst by adding an additional equivalent of both HBPin and benzaldehyde to a reaction mixture of 2, HBPin and iso-valeraldehyde (1 mol%, r.t.) after full conversion. The estimated signals of both products were detected in the ^1^H NMR spectrum after 24 h (Fig. S23†), proving full conversion of both aldehydes.

### Computational studies – reaction mechanism

**Fig. 3:**
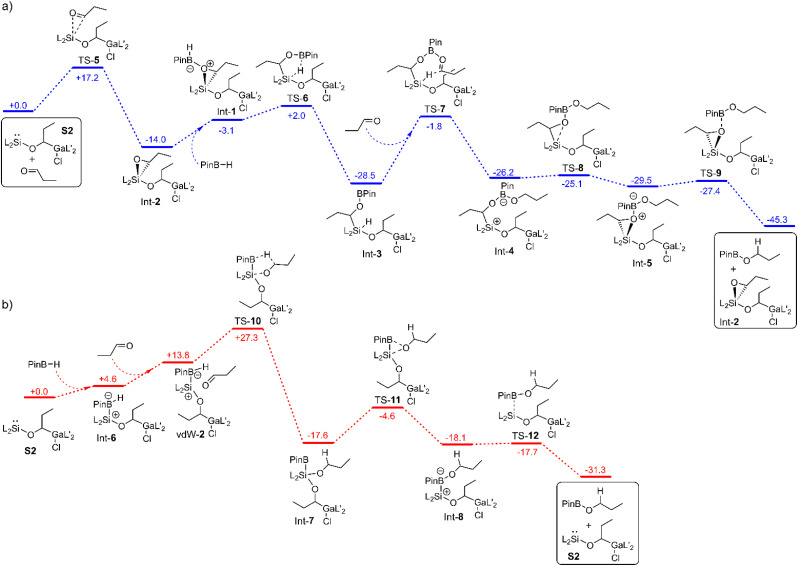
Mechanism for the hydroboration of *n*-propanal with Int-2 (a) and S2 (b) as catalytically active species calculated by means of PBE0-D3BJ. The values are given in kcal mol^−1^.

To find out the mechanism for the catalytic hydroboration reaction with S2 (or 2 if iso-valeraldehyde is used) we considered three different scenarios in our DFT studies. In the first case, the influence of the catalyst on the transition state of the uncatalysed reaction was calculated (Fig. S38†). In the other two cases, stepwise reactions of the components with the active species S2 were studied (Fig. 3a and b). In the first case, there is no lowering of the activation barrier. In fact, it is even higher (44.1 kcal mol^−1^) than that for the uncatalysed reaction (37.6 kcal mol; Fig. S38†) due to the entropy loss.

In the second case, the aldehyde is first added to the silylene center to form the oxasilirane Int-2 (Fig. 3a), which together with HBPin forms the complex Int-1, leading to the silane Int-3. The latter can then transfer the hydride to another aldehyde in the rate-determining step (26.7 kcal mol^−1^). In the corresponding seven-membered transition state TS-7, the oxygen atom of the carbonyl group is coordinated to the boron atom. The resulting intermediate Int-4 reacts to the product PinB-OPr and the catalytically active species Int-2 without any major activation barriers (Fig. 3a).

In the third case, the first step involves the addition of HBPin to form the complex Int-6 (Fig. 3b). The hydride on the boron atom now has a higher electron density and can therefore be transferred more efficiently to the carbon atom of the carbonyl group. In the corresponding five-membered transition state TS-10, the oxygen atom of the carbonyl group is bonded to the silicon atom. The activation barrier starting from the reactants amounts to 27.3 kcal mol^−1^. In the next step, the alkoxy group is transferred to the boron atom, forming the complex Int-8. The latter decomposes into the product PinB-OPr and the catalyst S2 (Fig. 3b).

A comparison of the latter cases reveals two completely different types of reaction. While in one case the hydride is transferred from the boron atom to the carbon atom, in the other case it is transferred from the silicon atom. While in one case S2 is the catalytically active species, in the other case Int-2 is the active catalyst. However, it is interesting to note, that in both cases the hydride transfer is the rate-determining step and the activation barriers are similar (27.3 kcal mol^−1^*versus* 26.7 kcal mol^−1^). Considering that the reaction takes place at room temperature, the calculated values are too high. This is due to the overestimation of the calculated entropies (for more details see calculation details). Therefore, the *G*_70%_ values were used for comparison with the experimental data (Fig. S40 and S41†). If the TOF values for the two mechanisms are estimated at room temperature, a TOF value of 0.9 h^−1^ is obtained for the first mechanism (*via* TS-7) and a value of 217 h^−1^ for the second one (*via* TS-10). The latter agrees well with the experimentally determined TOF values (133 h^−1^), which indicates that the second mechanism is dominant.

Unfortunately, we did not observe any reaction intermediate formed in these reactions by means of temperature-dependent *in situ*^1^H NMR spectroscopy. Even when the active silylene 2 was reacted with an equivalent of the aldehyde, we did not observe the formation of compound Int-2. In addition, we also did not observe the formation of Int-3 or Int-7 in reactions of 2 with an equivalent of the aldehyde and HBPin in the temperature range from –70 °C to room temperature.

## Experimental

### Materials and methods

All manipulations were performed in a purified argon atmosphere using standard Schlenk and glovebox techniques. Toluene was dried using an mBraun Solvent Purification System (SPS). Deuterated benzene was dried over activated molecular sieves (4 Å) and degassed prior to use. The anhydrous nature of the solvents was verified by Karl Fischer titration. L′(Cl)GaSiL C was prepared according to the literature.^42^ Microanalyses were performed at the Microanalysis Laboratory of the University of Duisburg-Essen. Melting points were measured using a Thermo Scientific 9300 apparatus.

#### Spectroscopic methods

^1^H (400 MHz), ^13^C{^1^H} (100 MHz) and ^29^Si{^1^H} (79 MHz) NMR spectra were recorded using a Bruker AscendTM 400 spectrometer, while ^1^H (600 MHz), ^13^C{^1^H} (151 MHz) and ^29^Si{^1^H} (119 MHz) NMR spectra were recorded using a BrukerAvanceIII HD 600 spectrometer. Catalytic reactions were monitored by ^1^H (300 MHz) and ^11^B (96 MHz) NMR spectroscopy using a Bruker DMX 300 spectrometer. The spectra were referenced to internal C_6_D_5_H (^1^H: *δ* = 7.16; ^13^C: *δ* = 128.06). ^11^B{^1^H} and ^29^Si{^1^H} NMR spectra were referenced using IUPAC recommendation of NMR nomenclature.^57^

IR spectra were recorded with an ALPHA-T FT-IR spectrometer equipped with a single reflection ATR sampling module. The IR spectrometer was placed in a glovebox to guarantee measurements under oxygen- and water-free conditions.

### Synthesis

#### General procedure for synthesis of L′Si[OCHCH_2_CHMe_2_]Ga(Cl)L (1)

A solution of iso-valeraldehyde (25.84 mg, 0.3 mmol) in 1 mL of toluene was added dropwise to a cooled (−80 °C) solution of L(Cl)GaSiL′ (234 mg, 0.3 mmol) in 2 mL of toluene. The solution was stirred at −80 °C for 5 minutes, concentrated to 1 mL and stored at −18 °C for 12 h to give oxasilirane 1 in the form of colorless crystals.

Yield 54 mg (0.06 mmol, 21%). Mp. 137 °C. Elemental analysis for C_49_H_74_ClGaN_4_OSi: anal. calcd. C 67.77, H 8.59, N 6.45%. Found C 67.1, H 8.38, N 6.41%. IR: *ν* 3061, 2964, 2952, 2931, 2868, 1537, 1518, 1464, 1435, 1382, 1361, 1318, 1260, 1204, 1177, 1078, 1022, 940, 892, 863, 796, 761, 732, 709, 621, 594, 544, 503, 466, 447. Oxasilirane 1 slowly converted in solution to compound 2 even at −5 °C. Therefore, the ^1^H, ^13^C and ^29^Si NMR spectra of compound 1 also contain small resonances due to compound 2. ^1^H NMR (600 MHz, C_6_D_6_): *δ* 7.46–6.75 (m, 11H, C_6_*H*_3_(^i^Pr)_2_ & C_6_*H*_5_), 5.17 (s, 1H, γ-C*H*), 4.24 (sept, ^3^*J*_HH_ = 6.7 Hz, 1H), 4.00 (sept, ^3^*J*_HH_ = 6.7 Hz, 1H, –C*H*(CH_3_)_2_), 3.49 (sept, ^3^*J*_HH_ = 6.6 Hz, 1H, –C*H*(CH_3_)_2_), 3.39 (sept, ^3^*J*_HH_ = 6.5 Hz, 1H, –C*H*(CH_3_)_2_), 2.18 (dd, ^3^*J*_HH_ = 11.3, 3.0 Hz, 1H, OC*H*), 2.13 (sept, ^3^*J*_HH_ = 6.4 Hz, 1H, –OCHCH_2_C*H*(CH_3_)_2_), 1.74 (dd, ^3^*J*_HH_ = 12.7, 3.9 Hz, 1H, –OCHC*H*_*2*_CH(CH_3_)_2_), 1.70 (d, ^3^*J*_HH_ = 6.7 Hz, 3H, –CH(C*H*_*3*_)_2_), 1.65 (s, 3H, ArNCC*H*_3_), 1.64 (s, 3H, ArNCC*H*_3_), 1.63 (d, ^3^*J*_HH_ = 6.6 Hz, 3H, –CH(C*H*_3_)_2_), 1.55 (d, ^3^*J*_HH_ = 3.2 Hz, 1H, OCHC*H*_2_CH(CH_3_)_2_), 1.53 (d, ^3^*J*_HH_ = 6.6 Hz, 3H, –CH(C*H*_*3*_)_2_), 1.48 (d, ^3^*J*_HH_ = 6.8 Hz, 3H, –CH(C*H*_*3*_)_2_), 1.32 (d, ^3^*J*_HH_ = 6.8 Hz, 3H, –CH(C*H*_*3*_)_2_), 1.26 (d, ^3^*J*_HH_ = 6.7 Hz, 3H, –CH(C*H*_*3*_)_2_), 1.18 (s, 9H, C(C*H*_3_)_3_), 1.16 (d, ^3^*J*_HH_ = 5.3 Hz, 6H, OCHCH_2_CH(C*H*_3_)_2_), 1.13 (d, ^3^*J*_HH_ = 6.7 Hz, 3H, –CH(C*H*_*3*_)_2_), 1.08 (d, ^3^*J*_HH_ = 6.6 Hz, 3H, –CH(C*H*_3_)_2_), 0.75 (s, 9H, C(C*H*_3_)_3_). ^13^C NMR (151 MHz, C_6_D_6_): *δ* 174.24 (N*C*N), 169.26, 168.54 (ArN*C*CH_3_), 146.14, 145.29, 144.67, 144.09 (NC*C*(CH(CH_3_)_2_)), 143.92, 143.65 (N*C*C(CH(CH_3_)_2_)), 134.12, 128.69, 127.69, 127.55, 127.46, 127.21 (*C*_6_H_5_), 129.63, 129.58, 124.79, 124.71, 124.59, 124.12 (*C*_6_H_3_), 99.89 (γ-*C*H), 59.20 (O*C*H), 53.64, 52.66 (*C*(CH_3_)_3_), 44.29 (OCH*C*H_2_CH(CH_3_)_2_), 31.51, 31.22 (C(*C*H_3_)_3_), 30.24, 29.17, 28.25, 28.15 (*C*H(CH_3_)_2_), 27.21 (OCHCH_2_*C*H(CH_3_)_2_), 24.89, 24.41 (ArNC*C*H_3_), 25.36, 24.33 (OCHCH_2_CH(*C*H_3_)_2_), 28.49, 26.37, 25.01, 24.26, 23.64, 22.61 (CH(*C*H_3_)_2_). ^29^Si NMR (119 MHz, toluene-d_8_): *δ* −112.79 (s).

#### General procedure for synthesis of L′SiOCH[Ga(Cl)L]CH2CHMe2 (2)

A solution of iso-valeraldehyde (25.84 mg, 0.3 mmol) in 1 mL of toluene was added dropwise to a solution of L(Cl)GaSiL′ (234 mg, 0.3 mmol) in 2 mL of toluene. The resulting solution was stirred for 12 h, after which the solvent was removed under vacuum and the remaining solid was dissolved in 4 mL of *n*-hexane, filtered and concentrated to 1 mL. Colorless crystals of 2 were formed within 12 h upon storage at room temperature.

Yield 172 mg (0.2 mmol, 66%). Mp. 142 °C. Elemental analysis for C_49_H_74_ClGaN_4_OSi: anal. calcd. C 67.77, H 8.59, N 6.45%. Found C 67.3, H 8.45, N 6.45%. IR: *ν* 3082, 2988, 2950, 2927, 2908, 2885, 2820,1547, 1518, 1484, 1438, 1419, 1384, 1381, 1318, 1285, 1258, 1210, 1175, 1108, 1051, 1018, 989, 933, 893, 798, 769, 757, 724, 707, 683, 610, 524, 490, 441, 434, 426. ^1^H NMR (400 MHz, C_6_D_6_): *δ* 7.41–6.78 (m, 11H, C_6_*H*_3_(^i^Pr)_2_ & C_6_*H*_5_), 5.05 (s, 1H, γ-C*H*), 4.33 (dd, ^3^*J*_HH_ = 9.7, 4.4 Hz, 1H, OC*H*), 4.12 (sept, ^3^*J*_HH_ = 6.7 Hz, 1H, –C*H*(CH_3_)_2_), 4.04 (sept, ^3^*J*_HH_ = 6.7 Hz, 1H, –C*H*(CH_3_)_2_), 3.52–3.45 (m, 1H, –C*H*(CH_3_)_2_), 3.44–3.37 (m, 1H, –C*H*(CH_3_)_2_), 2.42–2.22 (m, 1H, –OCHCH_2_C*H*(CH_3_)_2_), 1.68 (d, ^3^*J*_HH_ = 6.6 Hz, 3H, –CH(C*H*_*3*_)_2_), 1.65 (s, 4H, OCHC*H*_*2*_CH(CH_3_)_2_ & ArNCC*H*_3_), 1.63 (s, 3H, ArNCC*H*_3_), 1.58 (d, ^3^*J*_HH_ = 6.8 Hz, 3H, –CH(C*H*_3_)_2_), 1.55 (d, ^3^*J*_HH_ = 6.8 Hz, 3H, –CH(C*H*_3_)_2_), 1.31 (d, ^3^*J*_HH_ = 6.8 Hz, 3H, –CH(C*H*_3_)_2_), 1.26 (d, ^3^*J*_HH_ = 6.8 Hz, 3H, –CH(C*H*_3_)_2_), 1.24 (s, 10H, OCHC*H*_*2*_CH(CH_3_)_2_ & C(C*H*_3_)_3_), 1.12 (d, ^3^*J*_HH_ = 6.8 Hz, 3H, –CH(C*H*_3_)_2_), 1.10 (d, ^3^*J*_HH_ = 6.8 Hz, 3H, –CH(C*H*_3_)_2_), 1.00 (s, 9H, C(C*H*_3_)_3_), 0.95 (d, ^3^*J*_HH_ = 6.6 Hz, 3H, OCHCH_2_CH(C*H*_3_)_2_), 0.76 (d, ^3^*J*_HH_ = 6.5 Hz, 3H, OCHCH_2_CH(C*H*_3_)_2_). ^13^C NMR (101 MHz, C_6_D_6_): *δ* 169.87, 169.76 (ArN*C*CH_3_), 161.78 (N*C*N), 146.00, 145.75, 143.13, 142.92 (NC*C*(CH(CH_3_)_2_)), 142.48, 142.18 (N*C*C(CH(CH_3_)_2_)), 134.78, 130.10, 129.22, 127.55, 127.32, 127.29 (*C*_6_H_5_), 125.38, 125.29, 123.98, 123.75 (*C*_6_H_3_), 98.77 (γ-*C*H), 68.31 (O*C*H), 52.93, 52.68 (*C*(CH_3_)_3_), 47.18 (–OCH*C*H_2_CH(CH_3_)_2_), 32.09 (C(*C*H_3_)_3_), 29.50, 29.16, 28.16 (–*C*H(CH_3_)_2_), 27.51, 26.01 (ArNC*C*H_3_), 24.99 (OCHCH_2_*C*H(CH_3_)_2_), 25.26, 25.14, 24.57, 24.01, 23.82 (CH(*C*H_3_)_2_), 21.91(OCHCH_2_CH(*C*H_3_)_2_). ^29^Si NMR (79 MHz, C_6_D_6_): *δ* −12.59 (s).

### Catalytic studies

#### General procedure for the catalytic hydroboration of aldehydes and ketones

HBPin (28.2 mg, 0.22 mmol, 1.1 equiv.) and the substrate (aldehyde and ketone: 0.20 mmol) were placed in a *J*-Young NMR tube, dissolved in 0.3 mL of C_6_D_6_ and 0.2 mL of a 0.01 M solution of 2 (1.73 mg, catalytic loading: 1 mol%) was added. The solution was vortexed to form a homogeneous solution. The progress of the reaction was monitored by ^1^H NMR spectroscopy and a suitable internal standard was used to determine the yield. The values reported in the literature confirm the chemical shifts of the products.^37,58–60^

### Mechanistic studies

#### Determination of kinetics

HBPin (25.6 mg, 0.2 mmol, 1 equiv.) and iso-valeraldehyde (17.2 mg, 0.2 mmol) were added to a *J*-Young NMR tube and dissolved in 0.3 mL of C_6_D_6_. 0.2 mL of a 0.01 M solution of 2 (1.73 mg, catalytic loading: 1 mol%) was injected *via* syringe and the resulting solution was shaken to form a homogeneous solution. The progress of the reaction was monitored by ^1^H NMR spectroscopy at 80 °C by taking a spectrum every 30 s.

#### Living reaction

HBPin (28.2 mg, 0.22 mmol, 1.1 equiv.) and iso-valeraldehyde (17.2 mg, 0.2 mmol) were added to a *J*-Young NMR tube and dissolved in 0.3 mL of C_6_D_6_. 0.2 mL of a 0.01 M solution of 2 (1.73 mg, catalytic loading: 1 mol%) were added and the solution shaken to form a homogeneous solution. The progress of the reaction was monitored by ^1^H NMR spectroscopy. After complete conversion, a second fraction of HBPin (28.2 mg, 0.22 mmol, 1.1 equiv.) and benzaldehyde (21.2 mg, 0.2 mmol) were added. After 24 h, the progress of the reaction was monitored by ^1^H NMR spectroscopy, which again showed complete conversion of both substrates.

#### Chemoselectivity studies

HBPin (12.8 mg, 0.1 mmol, 1 equiv.), iso-valeraldehyde (8.6 mg, 0.1 mmol) and acetophenone (12.0 mg, 0.1 mmol) were added to a *J*-Young NMR tube and dissolved in 0.4 mL of C_6_D_6_. Then 0.1 mL of a 0.01 M solution of 2 (0.87 mg, catalytic loading: 1 mol%) was added and the mixture was shaken to form a homogeneous solution. The progress of the reaction was monitored by ^1^H NMR spectroscopy and a suitable internal standard was used to determine the yield.

#### TMEDA reaction

TMEDA (0.2 mmol, 23.2 mg), HBPin (0.1 mmol, 12.8 mg) and benzaldehyde (0.1 mmol, 10.2 mg) were added to a *J*-Young NMR tube and dissolved in 0.4 mL of C_6_D_6_. A solution of compound 2 (0.01 mmol, 8.68 mg) in 0.1 mL of C_6_D_6_ was added and the resulting solution was then shaken to form a homogeneous solution. The expected product was obtained (conversion >99%).

### Crystallography

The crystals were mounted on nylon loops in inert oil. The data of 1 were collected on a Bruker AXS D8 Venture diffractometer with Photon II detector (monochromated Cu_Ka_ radiation, *λ* = 1.54178 Å, microfocus source) at 100(2) K, while those of 2 were collected on a Bruker AXS D8 Kappa diffractometer with APEX2 detector (monochromated Mo_Ka_ radiation, *λ* = 0.71073 Å) at 100(2) K. The structures were solved by Direct Methods (SHELXS-2013)^61^ and anisotropically refined by full-matrix least-squares on *F*^2^ (SHELXL-2017).^62,63^ Absorption corrections were performed semi-empirically from equivalent reflections on basis of multi-scans (Bruker AXS APEX3). Hydrogen atoms were refined using a riding model or rigid methyl groups. In oxasilirane 1, the ligands of the silicon atom show a correlated disorder over two sites. All corresponding bond lengths were restrained to be equal (SADI) and the phenyl ring of the amidinate was restrained to be planar (FLAT). Global RIGU and SIMU restraints were applied to the displacement parameters of the disordered atoms. Additional specific SIMU restraints or common displacement parameters (EADP) were used for atoms in close proximity. The solvent molecules were restrained to a regular hexagon (SADI, FLAT) and RIGU and SIMU restraints were applied to their displacement parameters. One of the solvent molecules is disordered over a centre of inversion. The local symmetry was ignored in the refinement (negative PART). Due to the vast disorder and the consequent use of restraints, the quantitative results may be unreliable and biased and should be interpreted with caution. The solvent molecule in silylene 2 is disordered over two sites. Its bond lengths and angles were restrained to be equal (SADI) and RIGU restraints were applied to its atoms' displacement parameters. The displacement parameters of one isopropyl group suggested disorder but attempts to resolve it yielded a very low occupancy for the minor component and non-positive definites for the displacement parameter. Restraints did not succeed to overcome this, so the model was ultimately discarded.

### Computational details

All calculations were performed by using the program package Gaussian 16.^64^ The geometrical parameters of the stationary points were optimized by means of the density functional methods PBE0,^54^ PBE^65,66^ and M06-2X^67^ with the empirical dispersion D3 ^68^ and D3BJ.^55^ The basis sets def2-SVP^69,70^ and 6-31G(d)^71,72^ were employed. For all stationary points no symmetry restriction was applied. Frequency calculations were carried out at each of the structures to verify the nature of the stationary point. It turned out that all transition states have exactly one imaginary frequency, whereas all other structures have none. Furthermore, the energies of the stationary points were calculated using the density functionals PBE0-D3BJ, PBE-D3BJ and M06-2X-D3 and the basis sets def2-TZVP^69,70^ and 6-311++G(d,p).^73–76^ Solvent effects were taken into account by using the solvent model SMD^77^ (benzene as solvent) for single point calculations. Turnover frequencies (TOF) of the catalytic reaction were estimated using the AUTOF program.^78–80^

A well-known problem during the calculation of the Gibbs energies is the overestimation of the calculated entropies. The entropies are always computed on the assumption of an ideal gas. This leads to a large deviation for the translation and conformational term of the entropy if the reaction takes place in solution.^81,82^ This is particularly dramatic for bi- and trimolecular reactions. Some authors have estimated that the total entropy in solution should be about 50–70% of that in the gas phase.^82–85^ In our case, we have therefore used the upper limit, *i.e.* 70% (instead of 100%) of the calculated entropy. The thus obtained the Gibbs energies G70% were used for comparison with experimental data.

## Conclusions

Gallasilylene C serves as a precatalyst in the catalytic hydroboration of aldehydes with HBPin. The active catalyst is an alkoxysilylene as demonstrated by the reaction of C with iso-valeraldehyde, which occurs with oxidative addition to give the oxasilirane 1 followed by rearrangement to alkoxysilylene 2. The hydroboration catalysis using alkoxysilylene 2 shows a living character and a good chemoselectivity in the hydroboration of aldehydes over ketones. Quantum chemical calculations provided mechanistic insights into the energetics of the formation of oxasilirane 1 and alkoxysilylene 2 and also provided two possible reaction mechanism for the catalytic hydroboration.

## Data availability

CCDC-2270892 (1) and -2270893 (2) contain the supplementary crystallographic data for this paper. Other experimental data are available in the ESI.†

## Author contributions

L. K., conzeptualization, investigation, writing – original draft; C. W., sc-XRD data acquisition and processing, writing – original draft; H. S., quantum chemical calculations, writing – original draft; G. H. quantum chemical calculations, supervision, writing – review&editing; S. S., conzeptualization, supervision, writing – review&editing, funding acquisition, project administration.

## Conflicts of interest

There are no conflicts to declare.

## Supplementary Material

SC-015-D3SC06842K-s001

SC-015-D3SC06842K-s002
