# Supplementary material for: Catalytic hydroboration of aldehydes and ketones with an electron-rich acyclic metallasilylene
Source: Chem Sci. 2024 Feb 12;15(11):4161–70. doi: 10.1039/d3sc06842k (PMC10935726; doi:10.1039/d3sc06842k)
Supplement: SC-015-D3SC06842K-s001 [file SC-015-D3SC06842K-s001.pdf]

Electronic Supplementary Material (ESI) for Chemical Science.
This journal is © The Royal Society of Chemistry 2024

Supporting Information

Catalytic Hydroboration of Aldehydes and Ketones with an Electron-rich Acyclic Metallasilylene

Leon Kapp,[a] Christoph Wölper,[a] Hannah Siera,[b] Gebhard Haberhauer,[b] Stephan Schulz[a,c]**

[a] L. Kapp, Dr. C. Wölper, Prof. Dr. S. Schulz, Institute of Inorganic Chemistry, University of Duisburg-Essen, Universitätsstraße 5-7, 45141 Essen (Germany).

[b] Prof. Dr. G.Haberhauer Institute for Organic Chemistry, University of Duisburg-Essen, Universitätsstraße 5–7, 45117 Essen (Germany).

[c] Center for Nanointegration Duisburg-Essen (CENIDE), University of Duisburg-Essen, Carl-Benz-Straße 199, 47057 Duisburg (Germany).

Corresponding author: stephan.schulz@uni-due.de

Content

I. Spectroscopic Characterization

Fig. S1 – S5. ^{1}H, ^{13}C NMR, ^{29}Si NMR and IR spectra of L'Si[OCHCH_2CHMe_2]Ga(Cl)L (**1**).

Fig. S6 – S9. ^{1}H, ^{13}C NMR, and IR spectra of L'SiOCH[Ga(Cl)L]CH_2CHMe_2 (**2**).

Fig. S10 – S29. ^{1}H NMR spectra of catalytic reactions and mechanistic studies.

II. Catalytic Studies

Table S1. Catalytic Table.

Characterization of catalytic products.

Fig. S30 – S33. Investigations of reaction kinetics.

III. Single Crystal X-ray Diffraction

Table S2. Crystal data and structure refinement of **1** and **2**.

Figure S34, S35. Molecular structure of **1** and **2**.

IV. Quantum chemical calculations

Figure S36 – S42. Calculated Gibbs energies for the studied reactions.

VI. Absolute Energies and Cartesian Coordinates for the Calculated Compounds

Table S3 – S6. Absolute energies and entropies calculated by means of different methods.

V. References

I. Spectroscopic Characterization

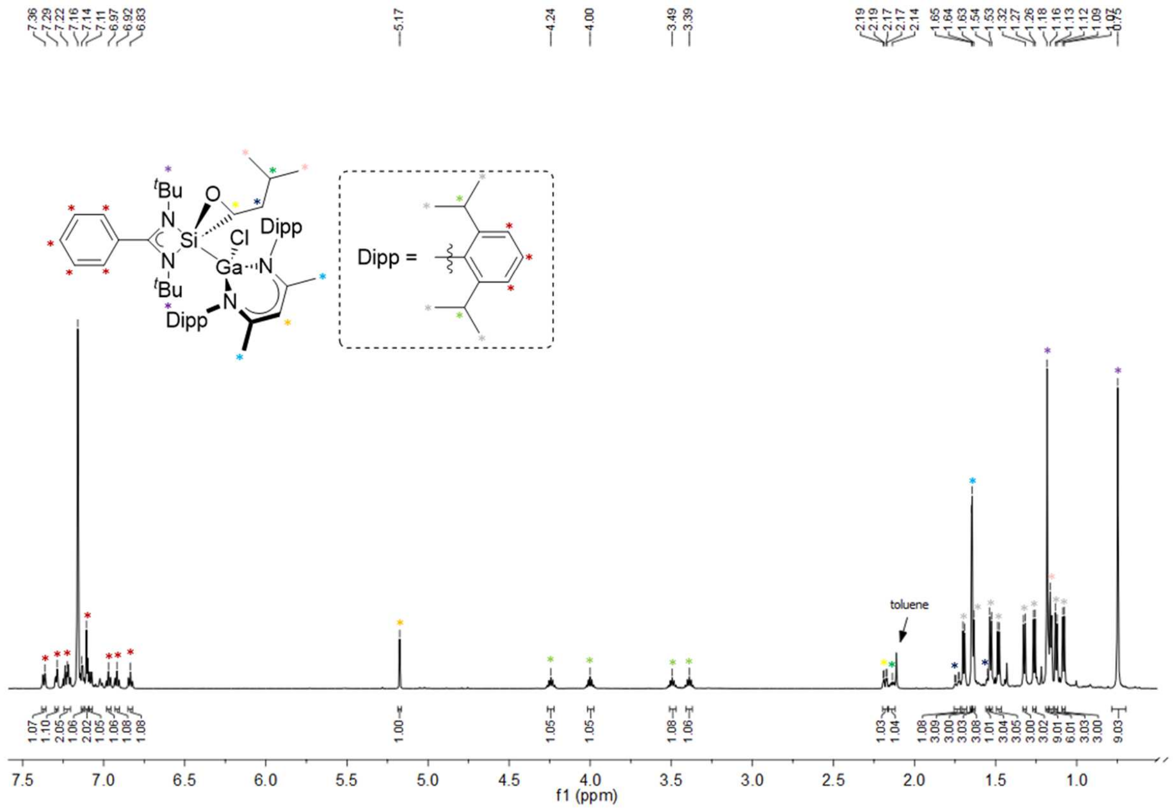

Figure S1. ^{1}H NMR spectrum of L'Si[OCHCH$_2$CHMe$_2$]Ga(Cl)L (**1**) in C_6D_6 at room temperature.

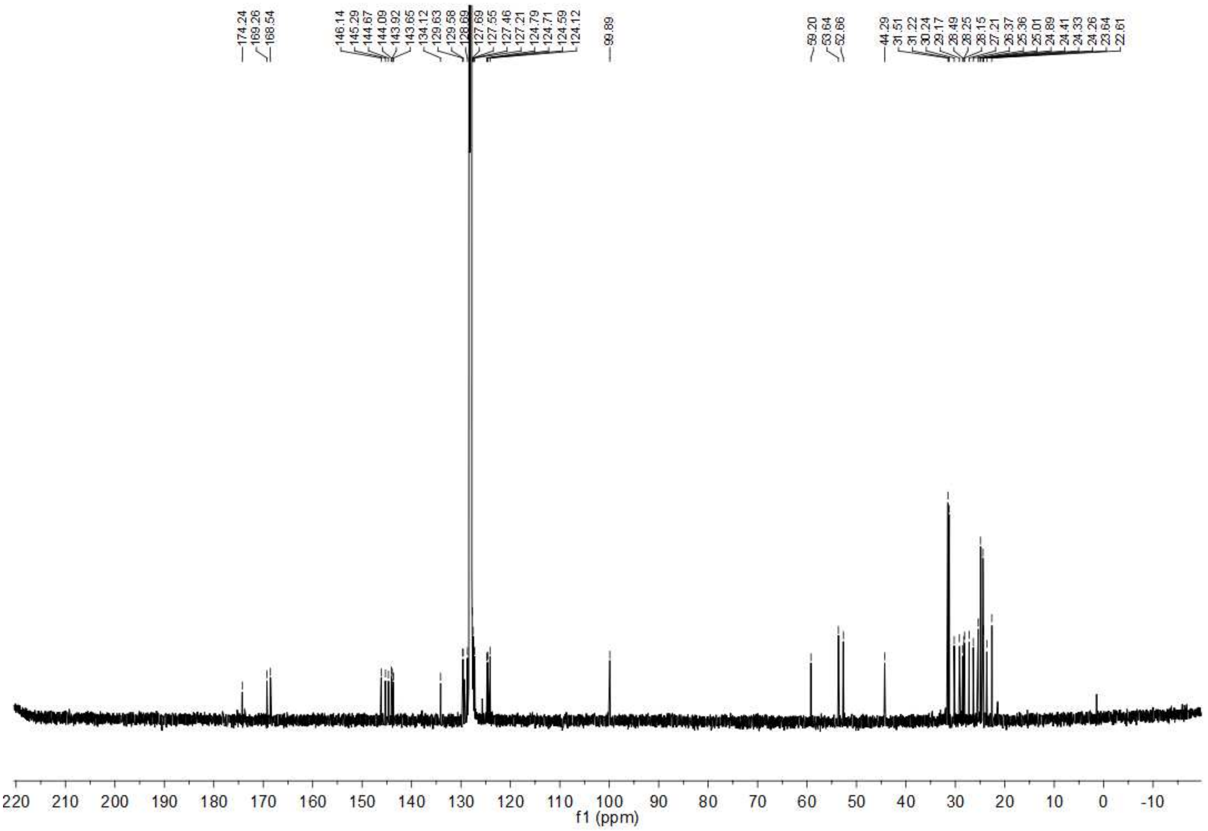

Figure S2. ^{13}C NMR spectrum of L'Si[OCHCH$_2$CHMe$_2$]Ga(Cl)L (**1**) in C_6D_6 at room temperature.

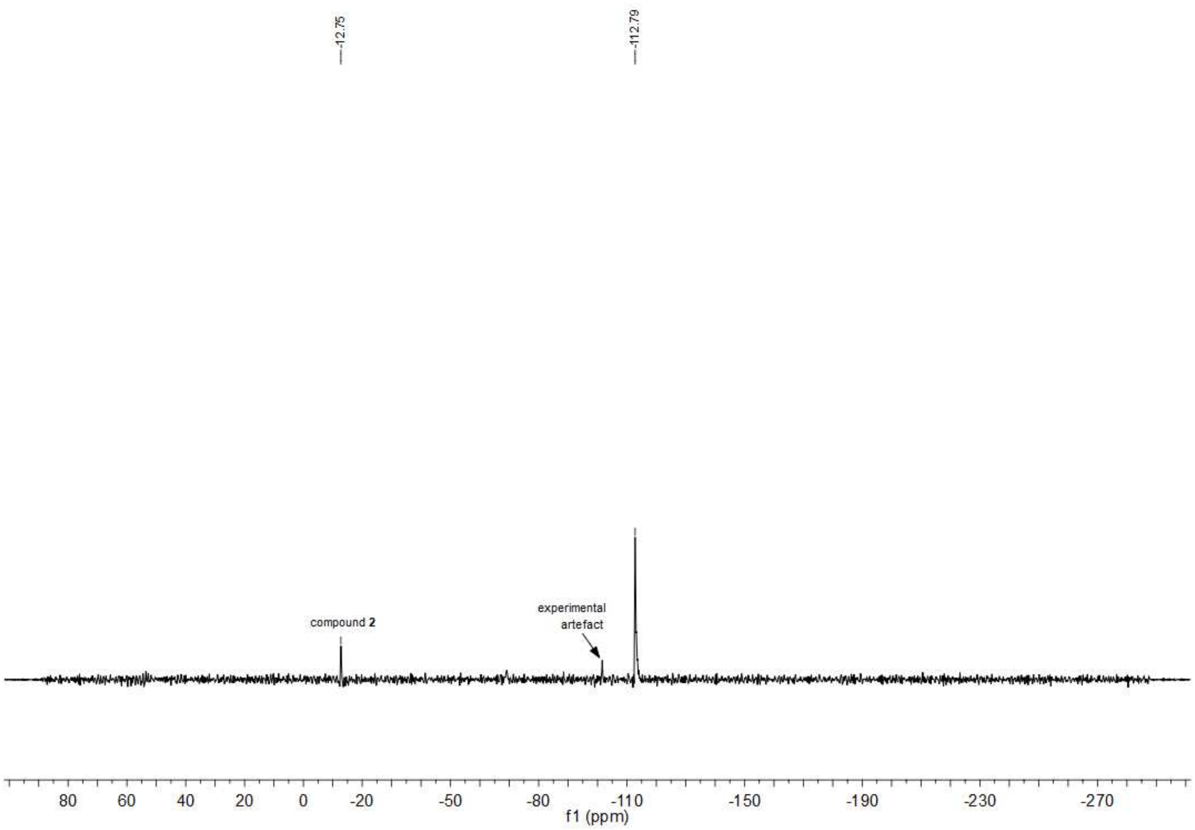

Figure S3. ^{29}Si NMR spectrum of L'Si[OCHCH$_2$CHMe$_2$]Ga(Cl)L (**1**) in C_6D_6 at room temperature

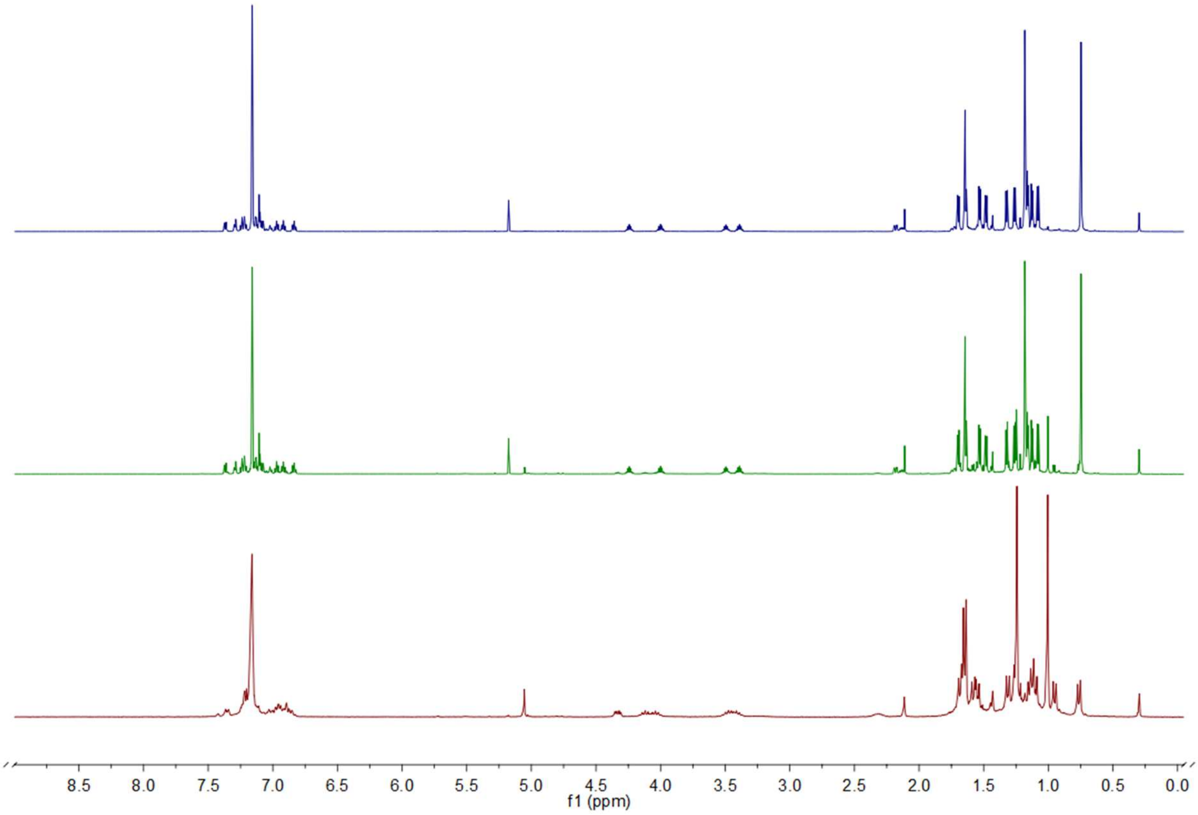

Figure S4. ^{1}H NMR spectra of L'Si[OCHCH$_2$CHMe$_2$]Ga(Cl)L (**1**) in C_6D_6 at room temperature after 3 h and 24 h, showing conversion of compound **1** to compound **2**.

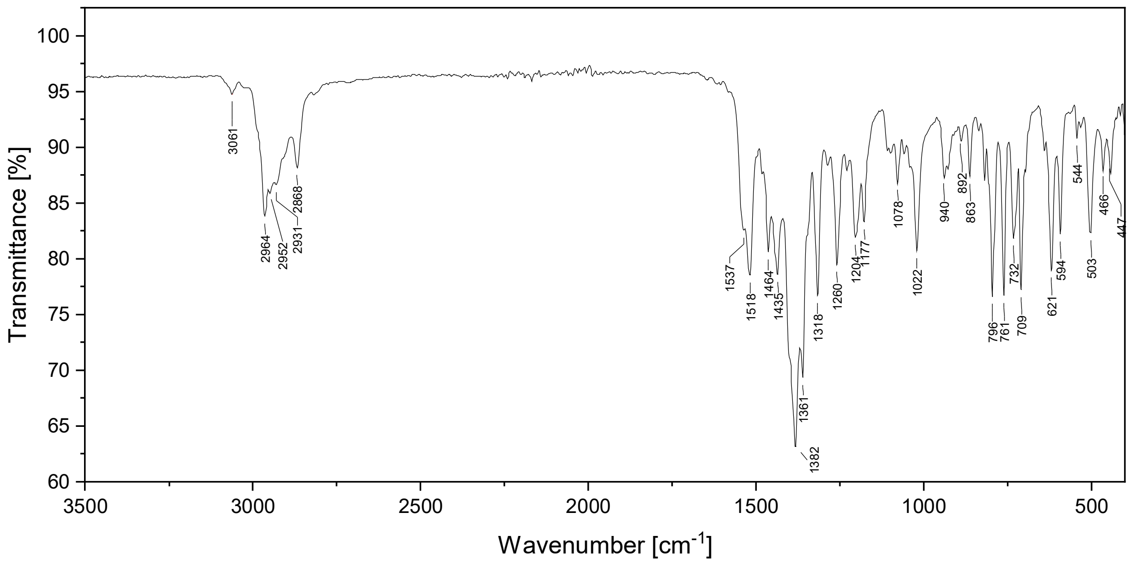

Figure S5. IR spectrum of L'Si[OCHCH$_2$CHMe$_2$]Ga(Cl)L (**1**).

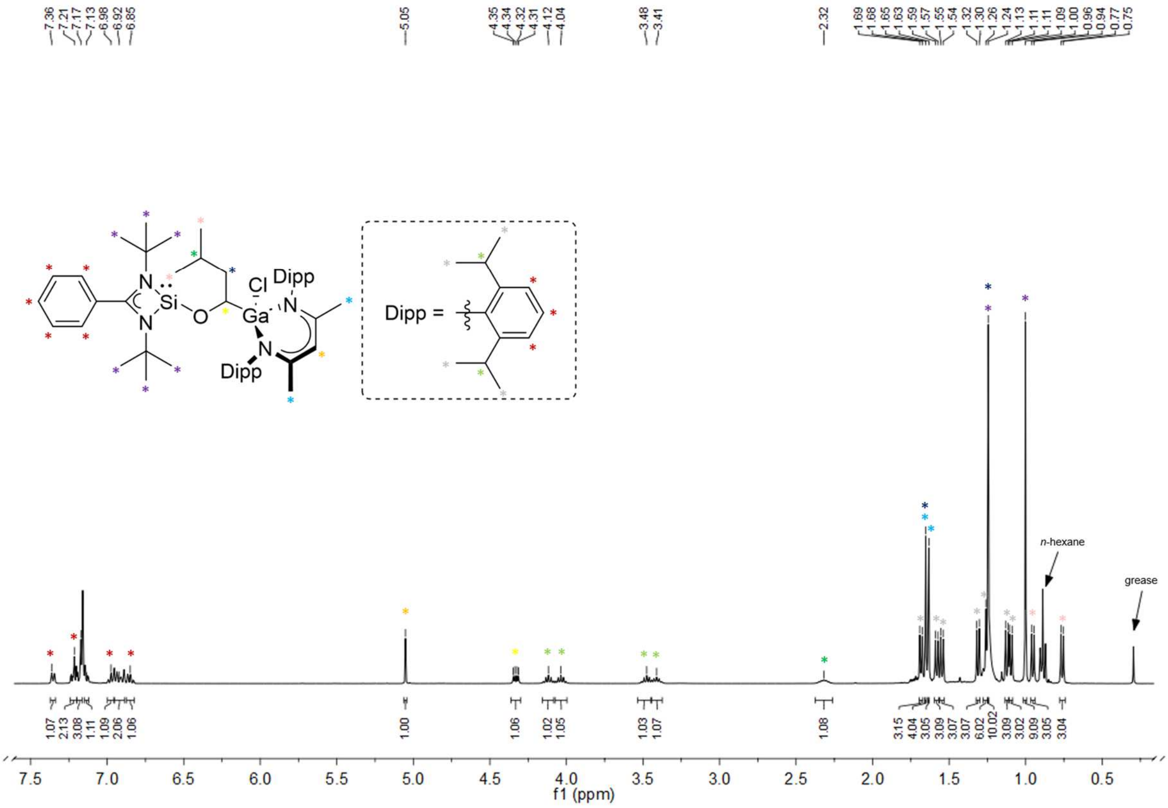

Figure S6. ^{1}H NMR spectrum of L'SiOCH[Ga(Cl)L]CH$_2$CHMe$_2$ (**2**) in C$_6$*D*$_6$ at room temperature.

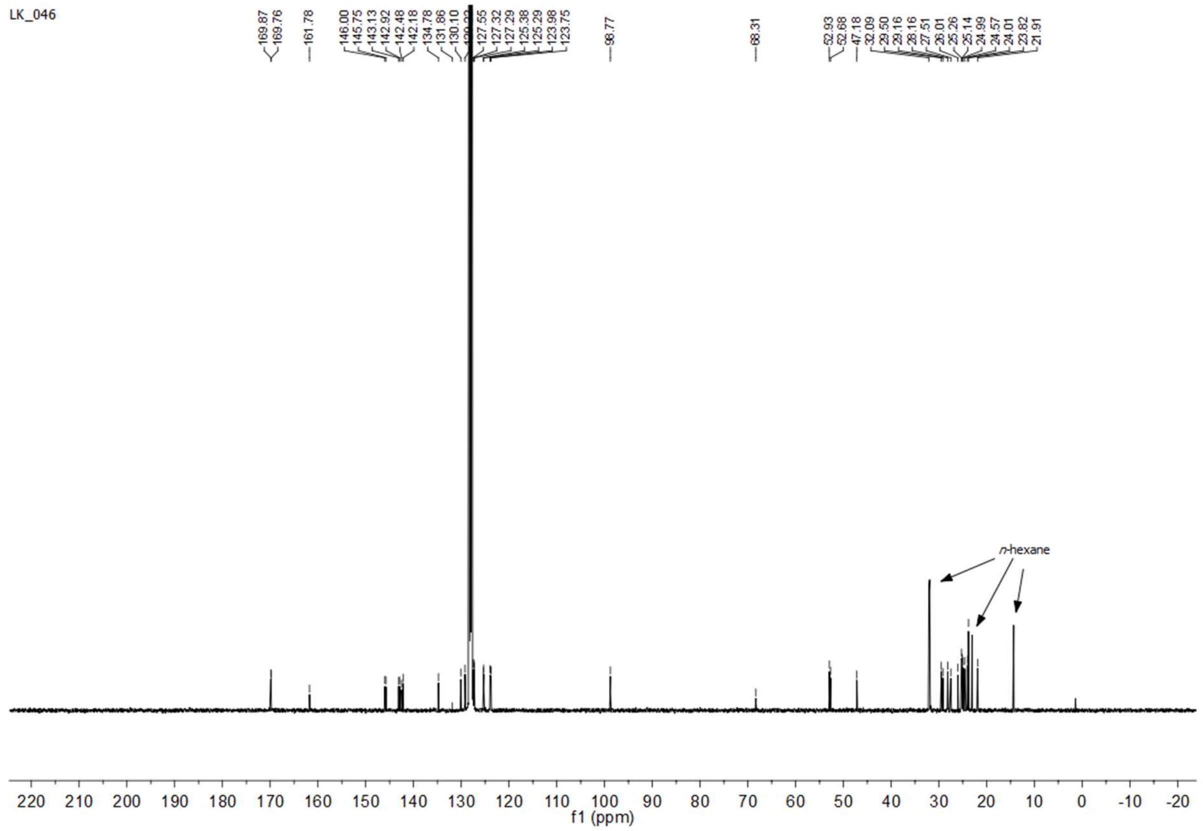

Figure S7. ^{13}C NMR spectrum of L'SiOCH[Ga(Cl)L]CH_2CHMe_2 (**2**) in C_6D_6 at room temperature.

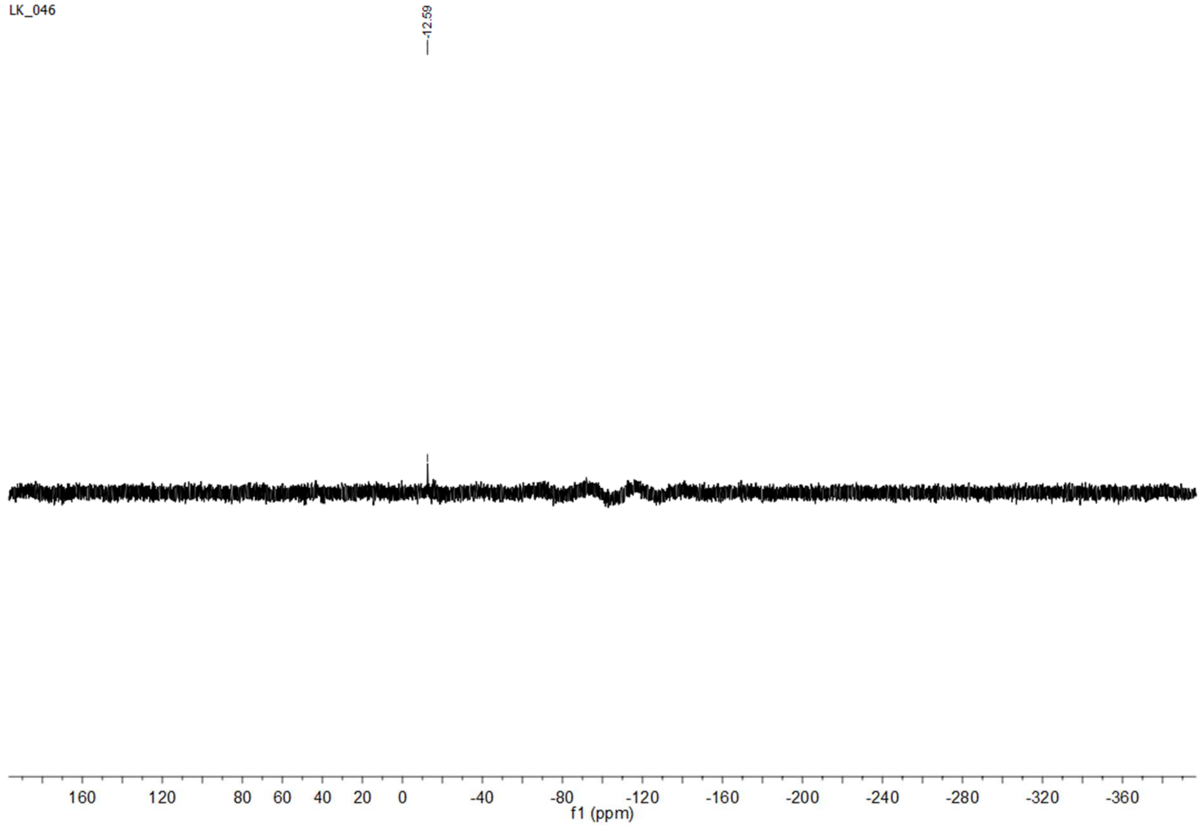

Figure S8. ^{29}Si NMR spectrum of L'SiOCH[Ga(Cl)L]CH_2CHMe_2 (**2**) in C_6D_6 at room temperature.

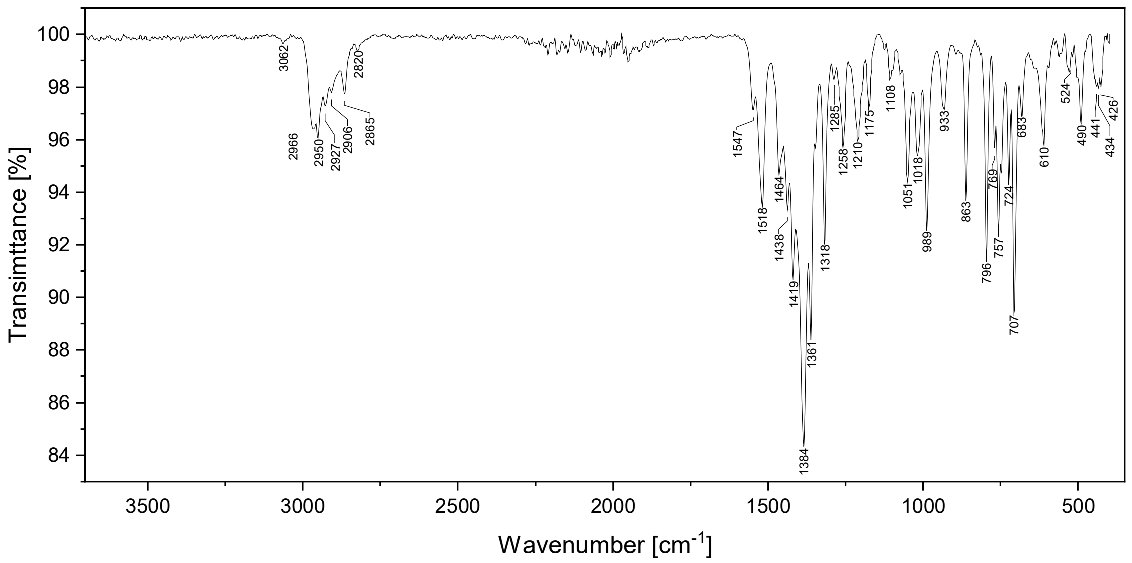

Figure S9. IR spectrum of L'SiOCH[Ga(Cl)L]CH_2CHMe_2 (**2**).

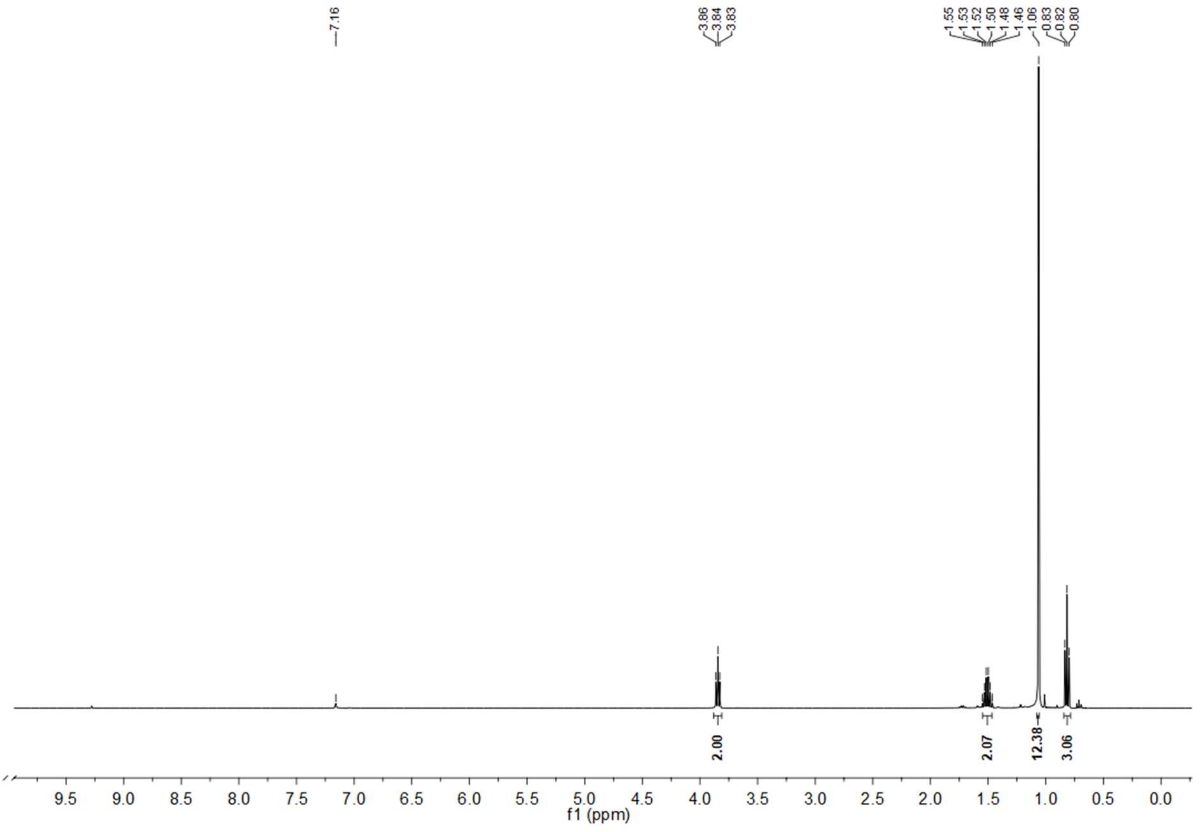

Figure S10. Crude ^{1}H NMR spectrum of the catalytic reaction (*n*-propanal and HBPin) with 1 mol% of **2** in C_6D_6 at room temperature.

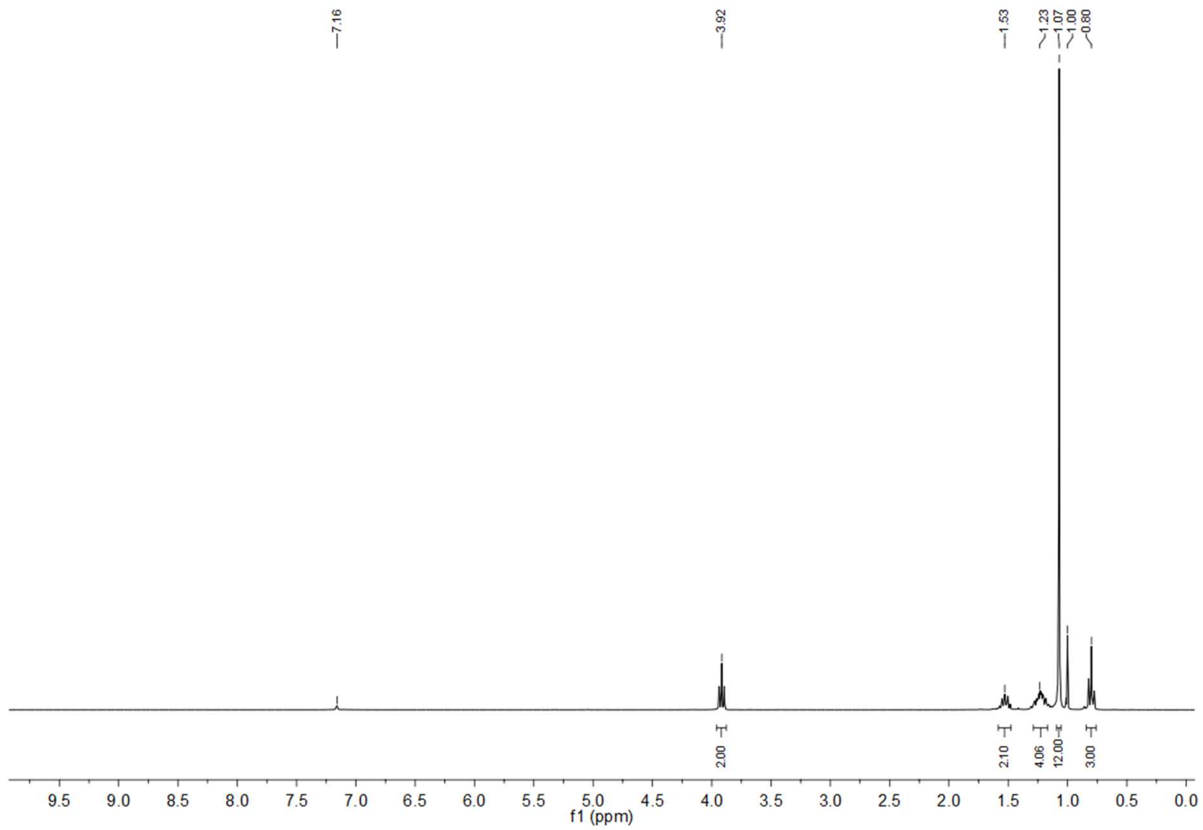

Figure S11. Crude ^{1}H NMR spectrum of the catalytic reaction (*n*-pentanal and HBPin) with 1 mol% of **2** in C_6D_6 at room temperature.

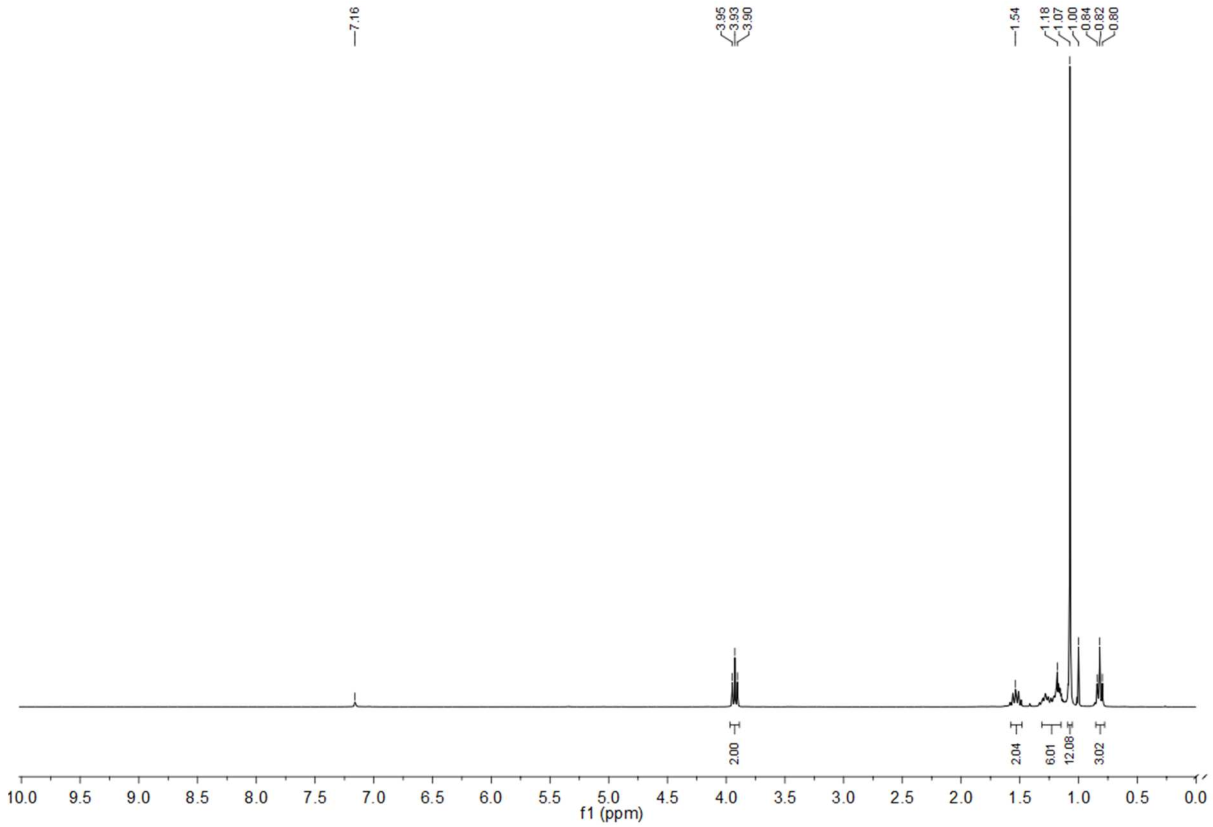

Figure S12. Crude ^{1}H NMR spectrum of the catalytic reaction (*n*-hexanal and HBPin) with 1 mol% of **2** in C_6D_6 at room temperature.

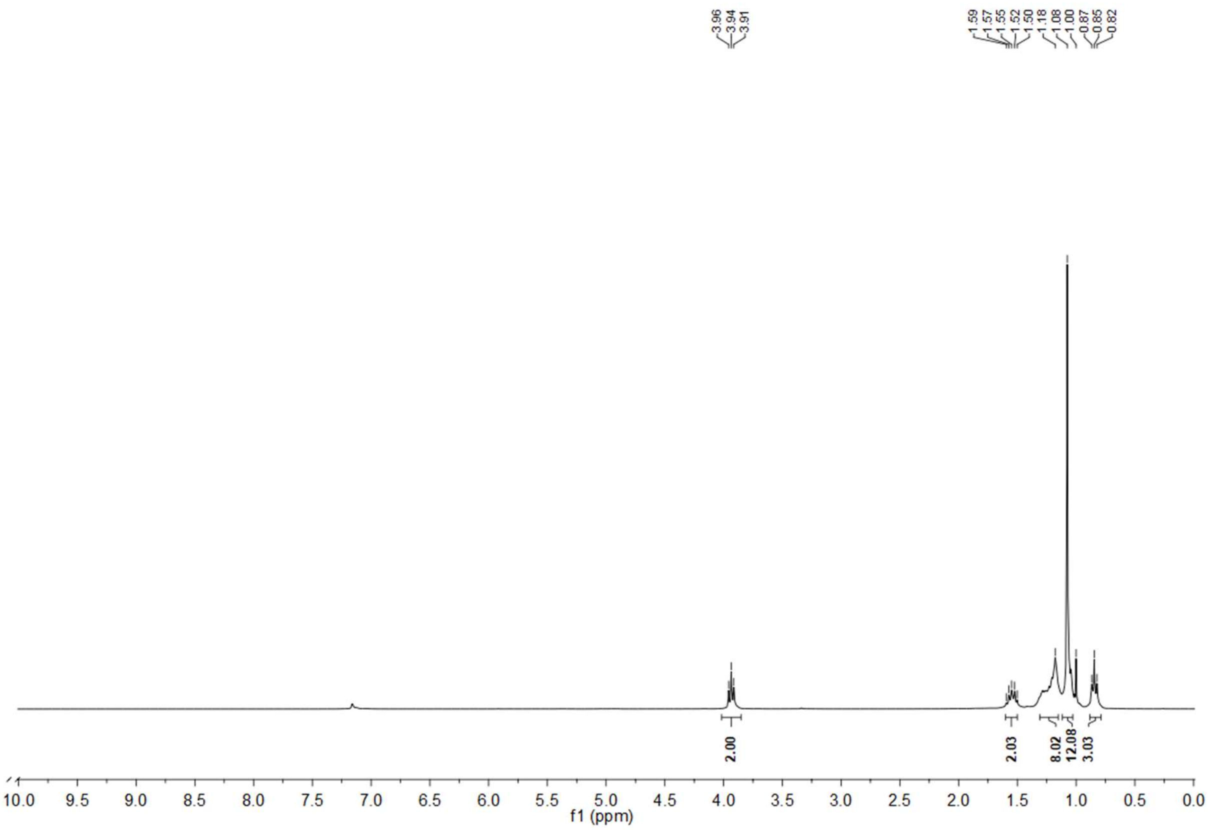

Figure S13. Crude ^{1}H NMR spectrum of the catalytic reaction (*n*-heptanal and HBPin) with 1 mol% of **2** in C_6D_6 at room temperature.

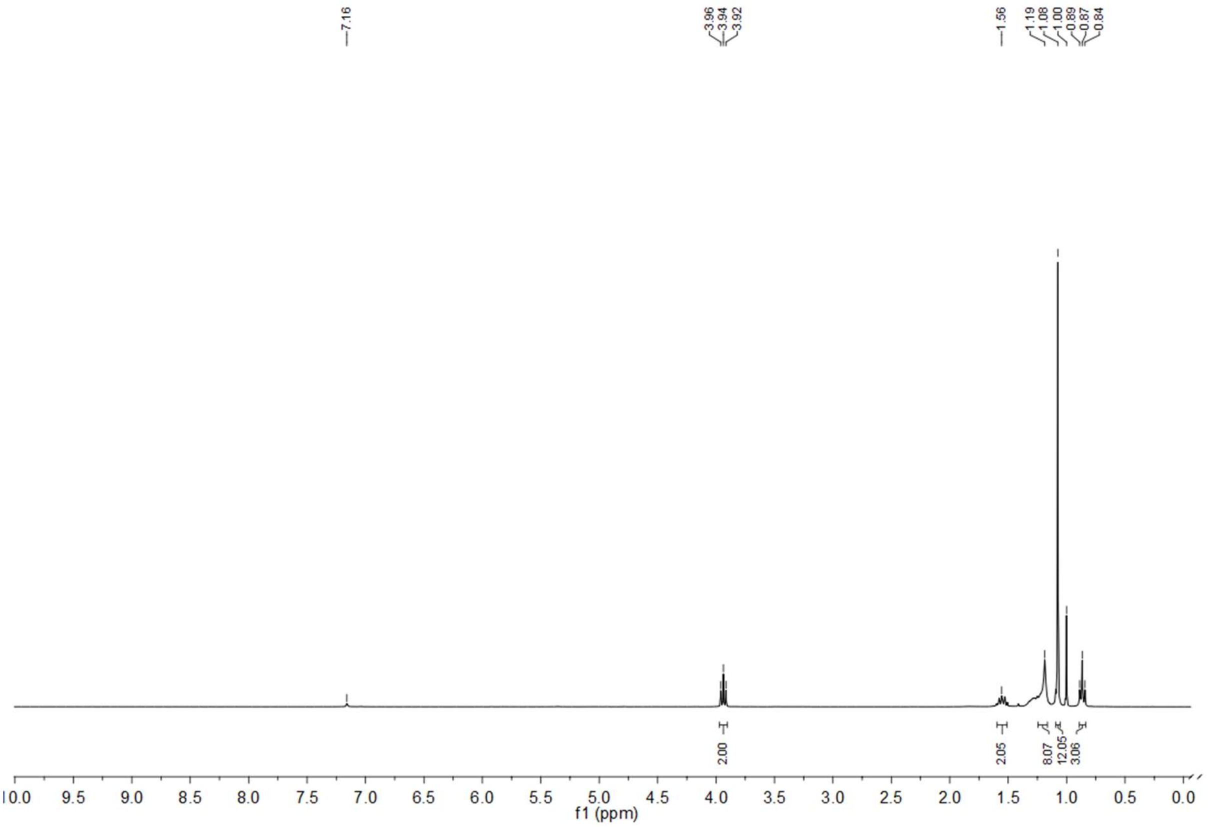

Figure S14. Crude ^{1}H NMR spectrum of the catalytic reaction (*n*-octanal and HBPin) with 1 mol% of **2** in C_6D_6 at room temperature.

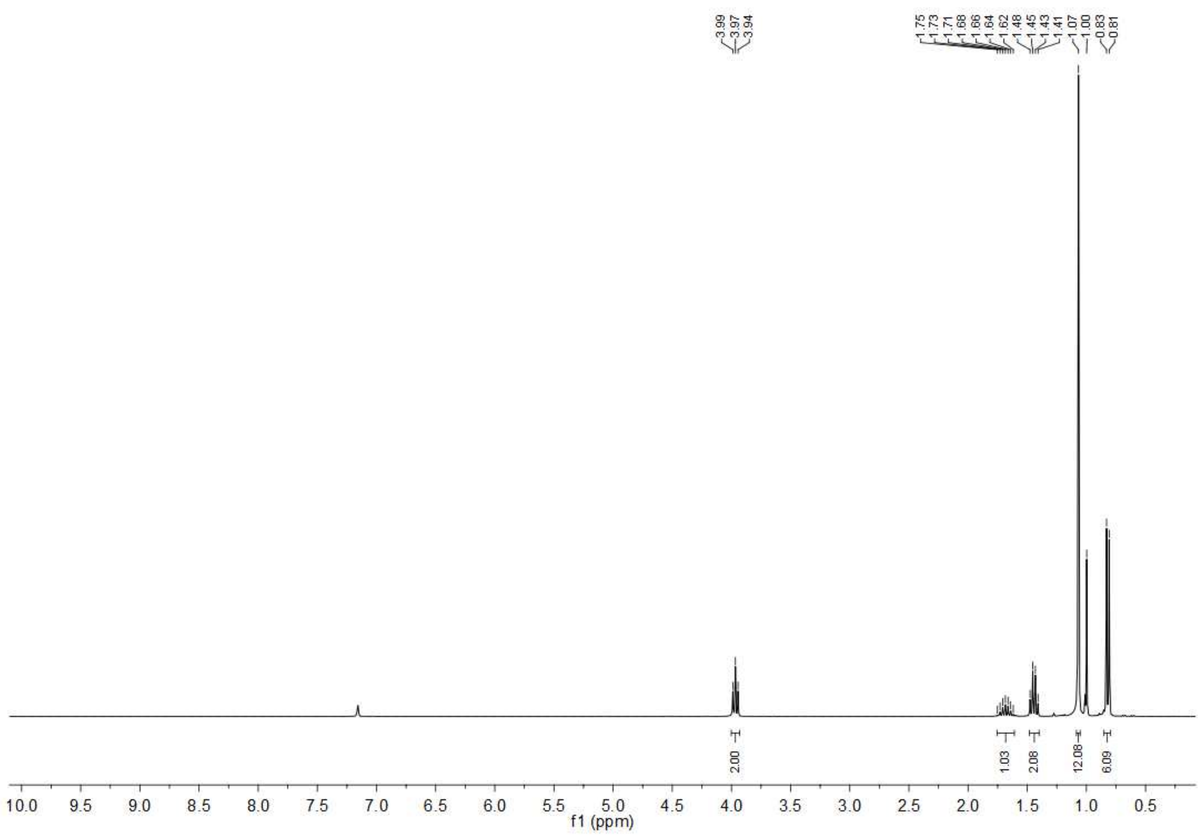

Figure S15. Crude ^{1}H NMR spectrum of the catalytic reaction (*iso*-valeraldehyde and HBPin) with 1 mol% of **2** in C_6D_6 at room temperature.

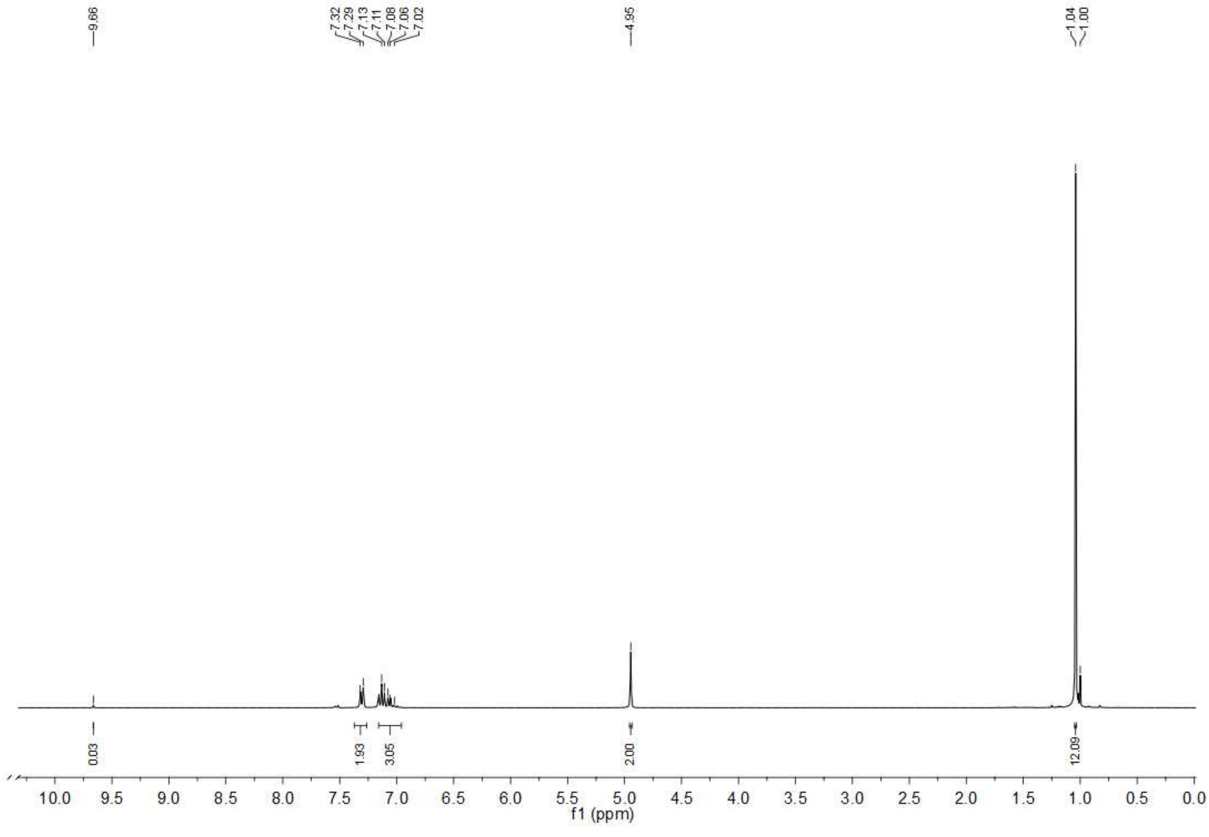

Figure S16. Crude ^{1}H NMR spectrum of the catalytic reaction (benzaldehyde and HBPin) with 1 mol% of **2** in C_6D_6 at room temperature.

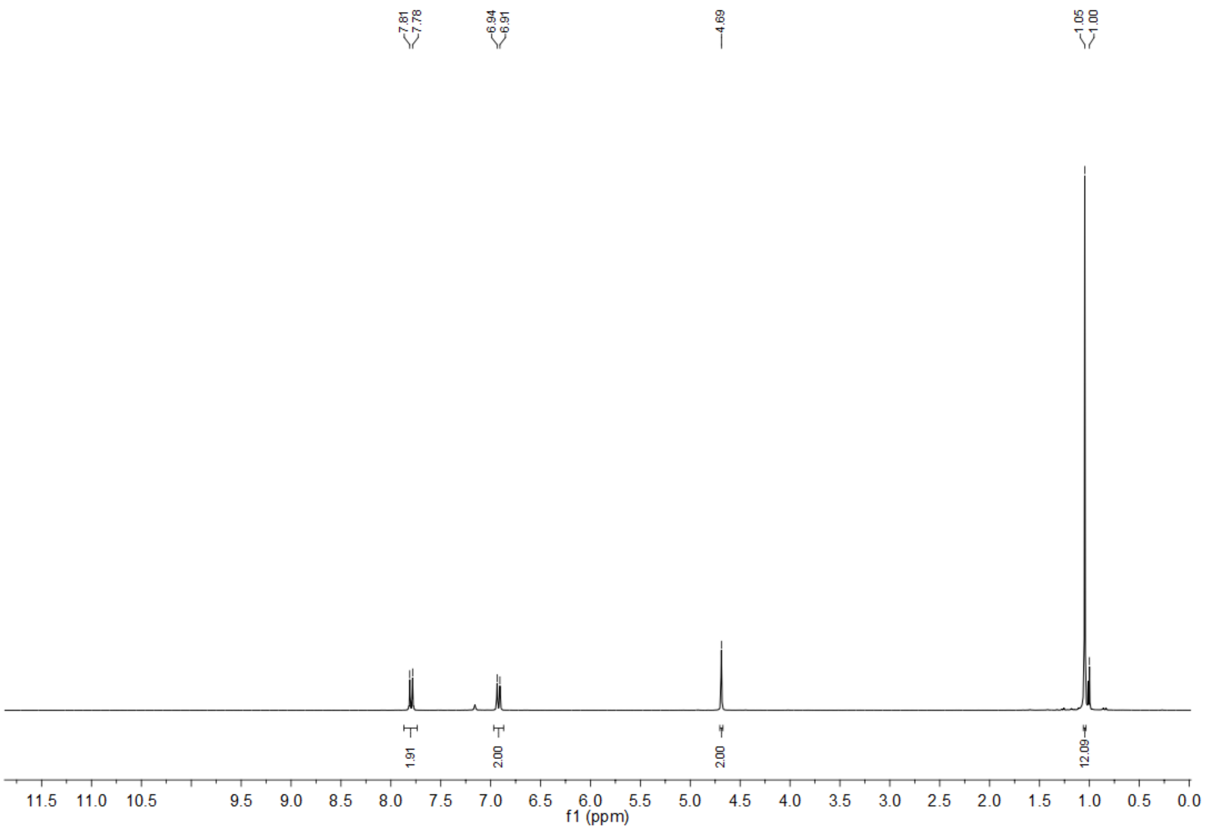

Figure S17. Crude ^{1}H NMR spectrum of the catalytic reaction (4-nitrobenzaldehyde and HBPin) with 1 mol% of **2** in C_6D_6 at room temperature.

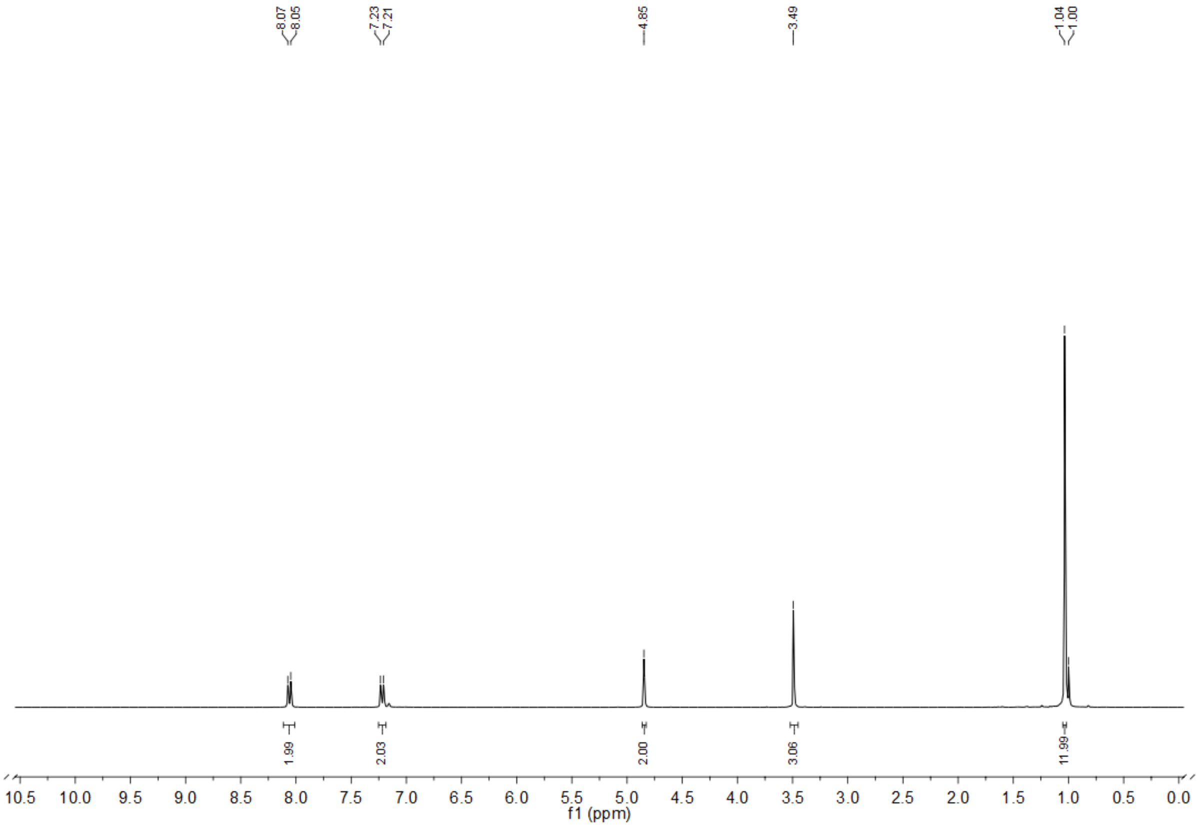

Figure S18. Crude ^{1}H NMR spectrum of the catalytic reaction (4-formylbenzoic acid and HBPin) with 1 mol% of **2** in C_6D_6 at room temperature.

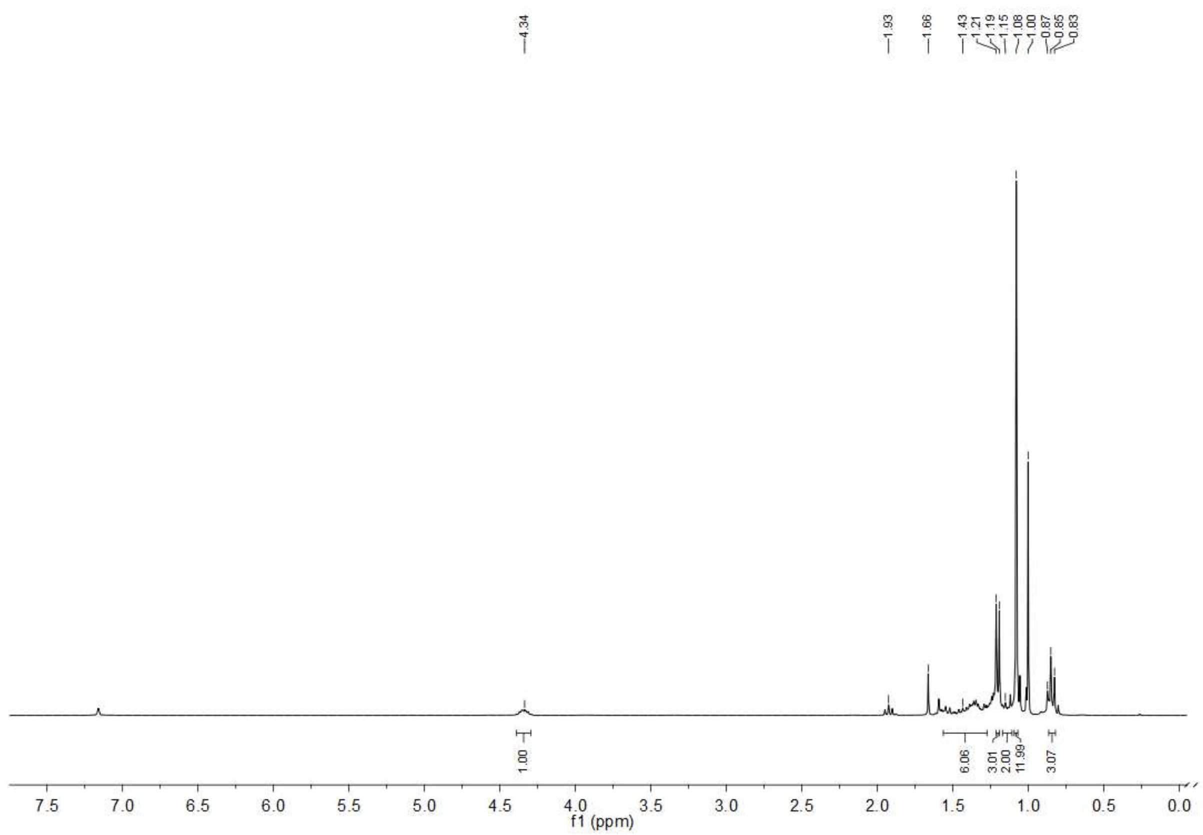

Figure S19. Crude ^{1}H NMR spectrum of the catalytic reaction (2-heptanone and HBPin) with 1 mol% of **2** in C_6D_6 at room temperature.

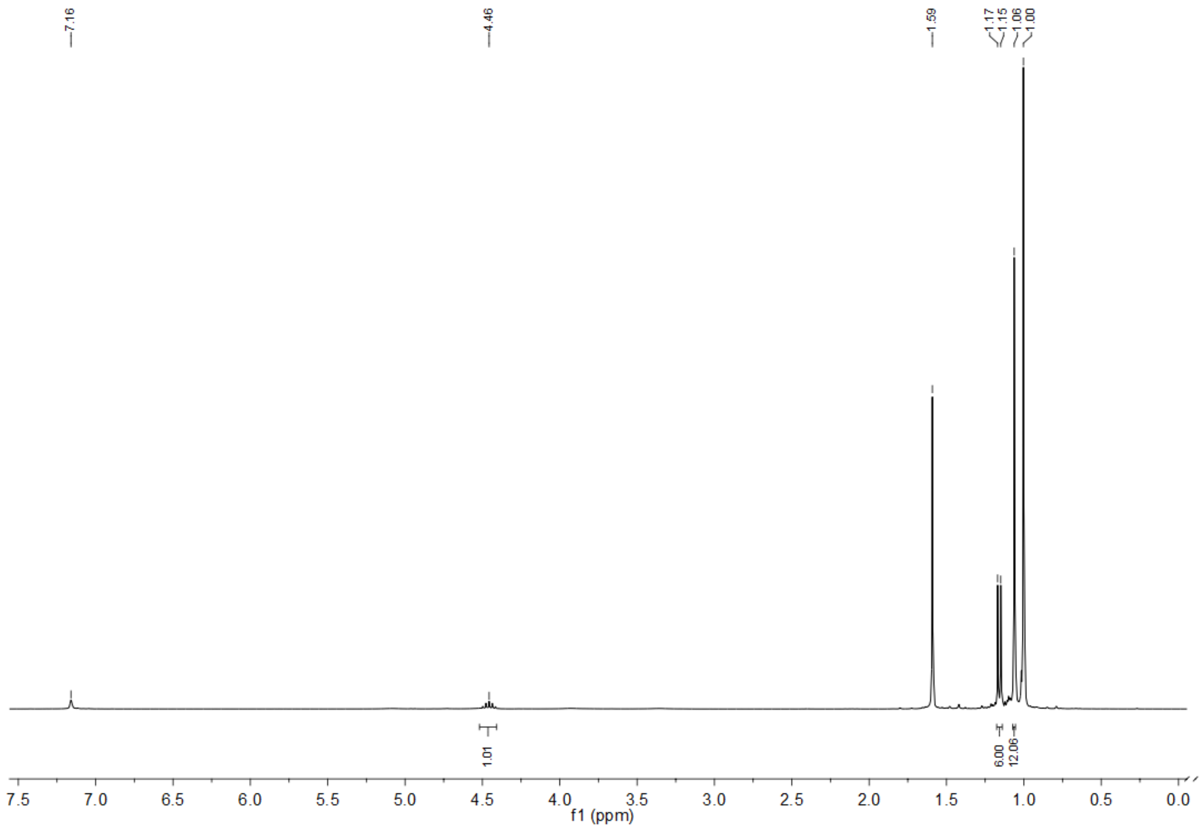

Figure S20. Crude ^{1}H NMR spectrum of the catalytic reaction (acetone and HBPin) with 1 mol% of **2** in C_6D_6 at room temperature.

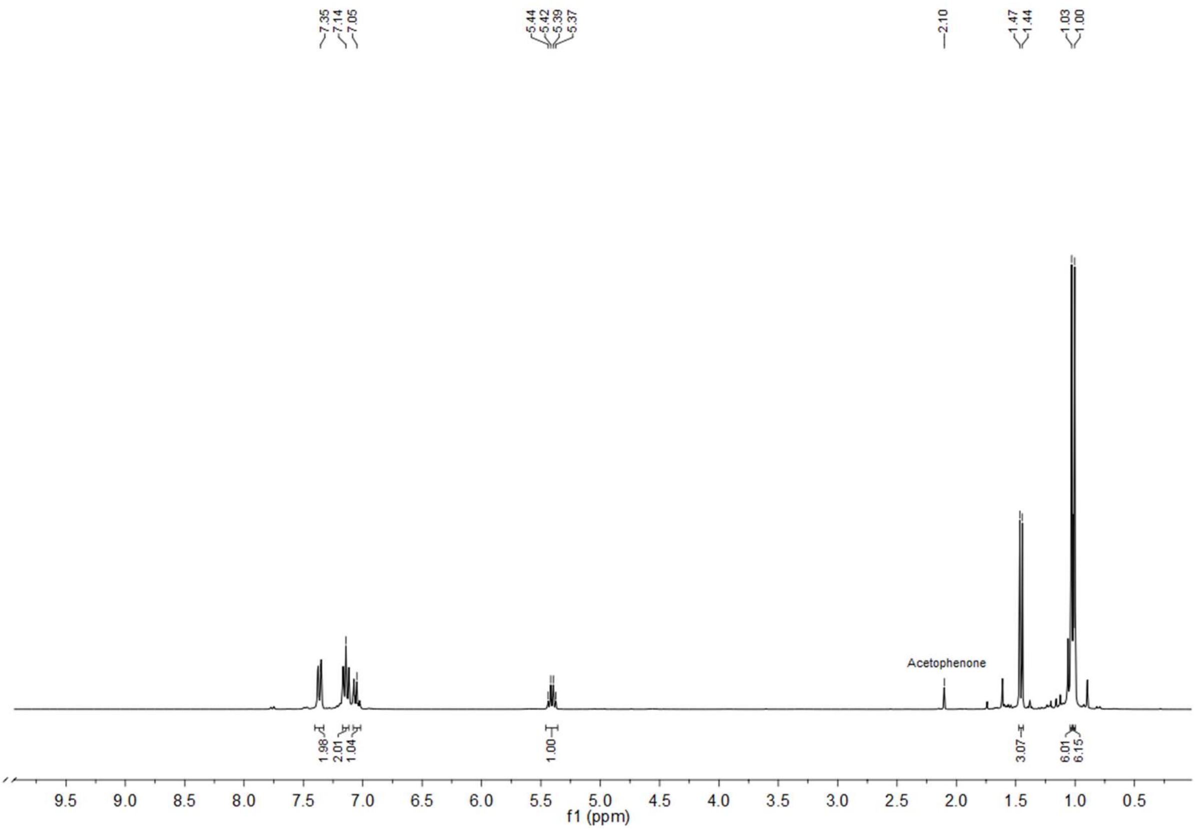

Figure S21. Crude ^{1}H NMR spectrum of the catalytic reaction (acetophenone and HBPin) with 1 mol% of **2** in C_6D_6 at room temperature.

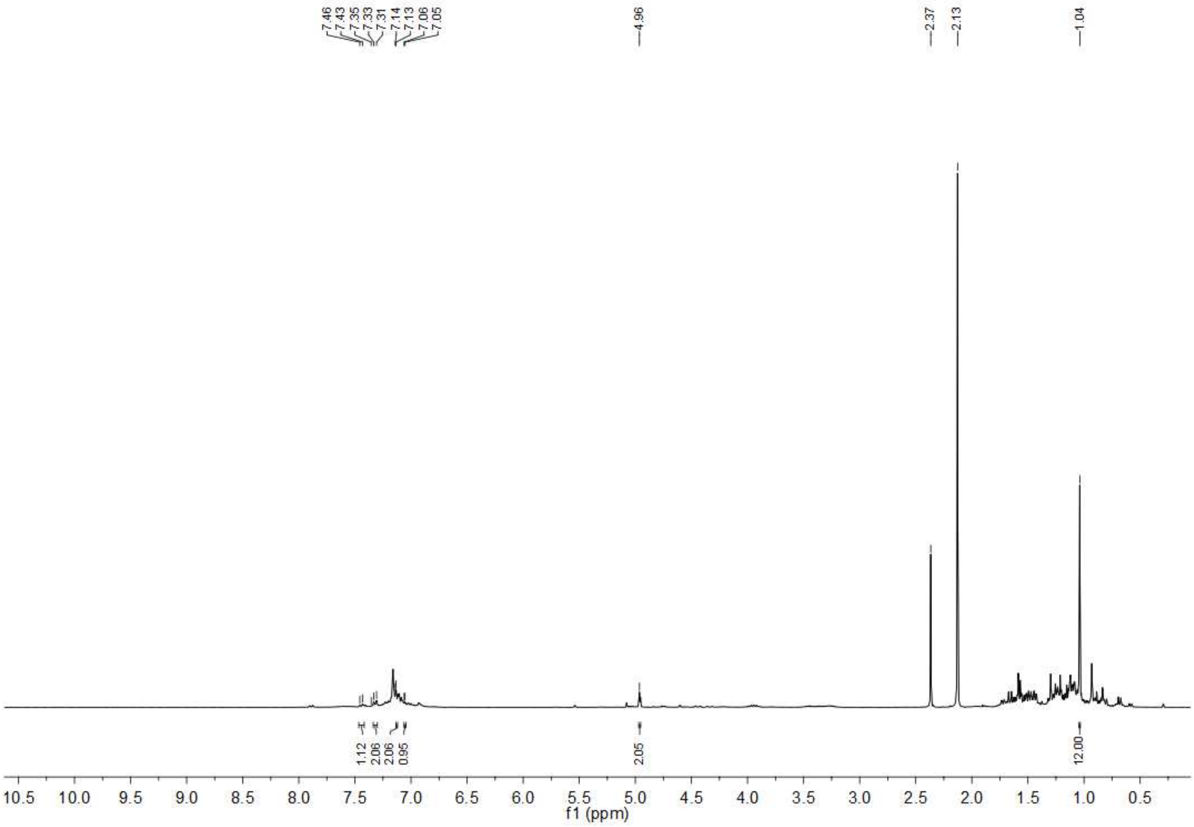

Figure S22. Crude ^{1}H NMR spectrum of the catalytic reaction (benzaldehyde + HBPin + 10 mol% **2**) with two equivalents of TMEDA in C_6D_6 at room temperature.

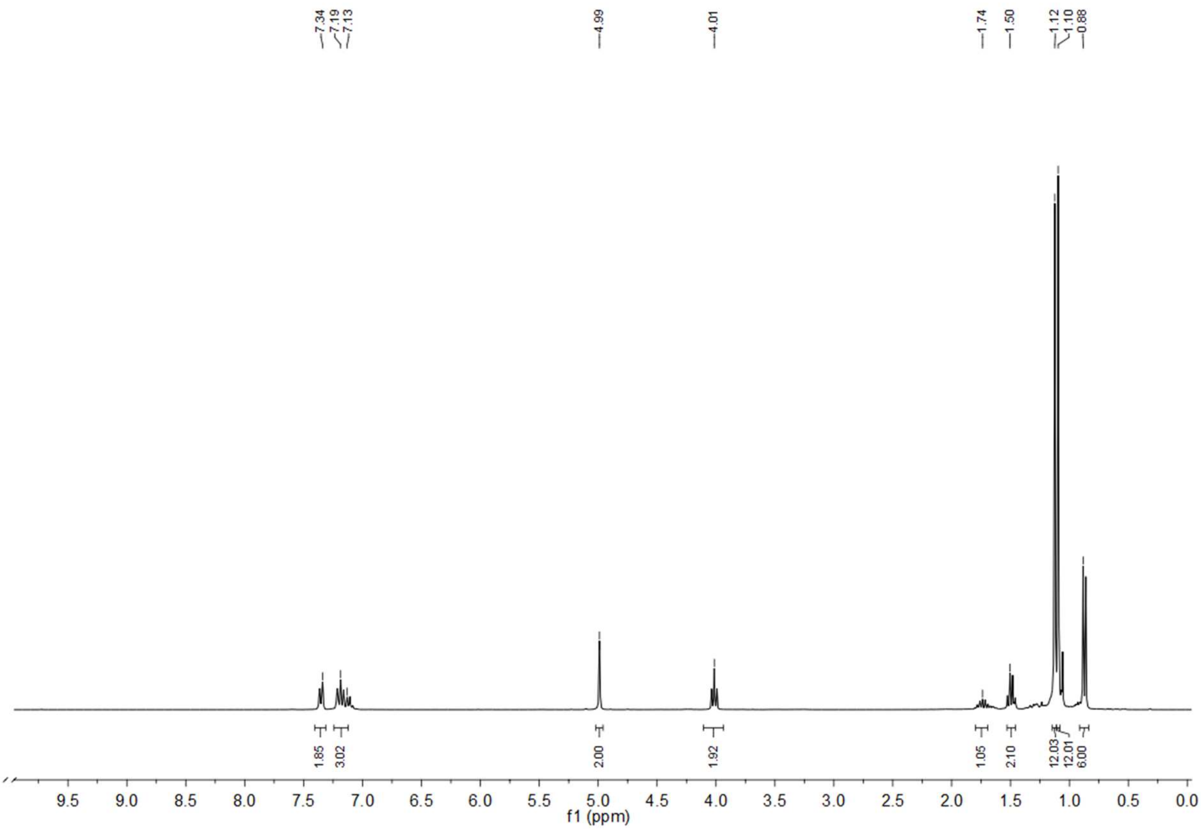

Figure S23. Crude ^{1}H NMR spectrum of the living catalytic reaction (*iso*-valeraldehyde, benzaldehyde and HBPin) with 1 mol% of **2** in C_6D_6 at room temperature.

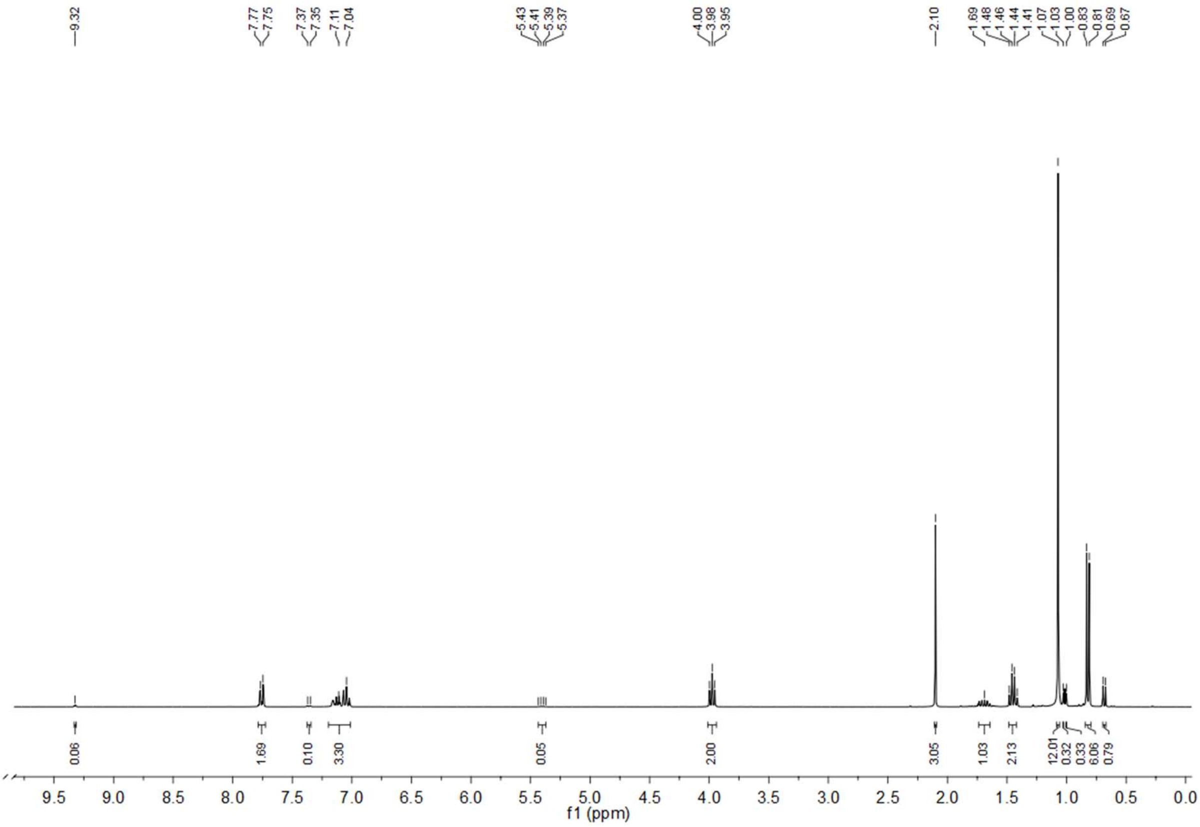

Figure S24. Crude ^{1}H NMR spectrum of the chemo selective catalytic reaction (*iso*-valeraldehyde, acetophenone and HBPin) with 1 mol% of **2** in C_6D_6 at room temperature.

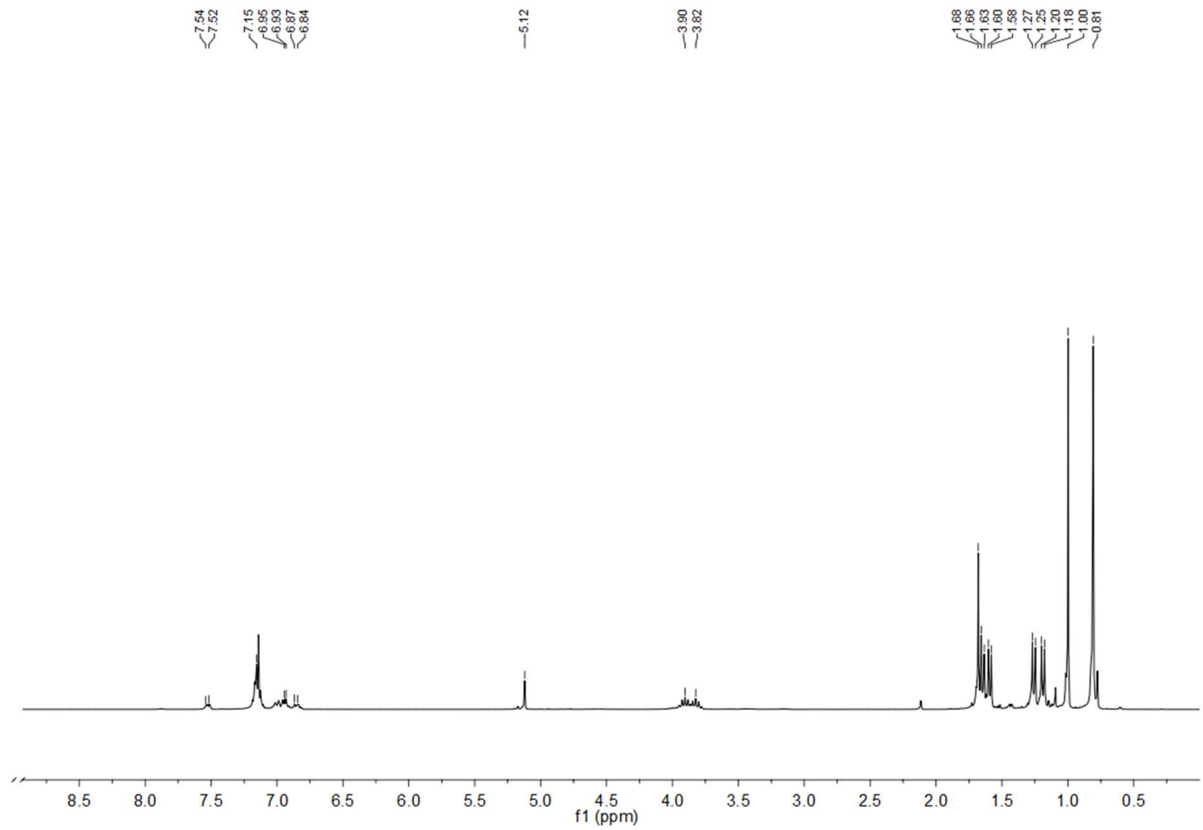

Figure S25. Crude ^{1}H NMR spectrum of **C** and HBPin showing no reaction in C_6D_6 at room temperature.

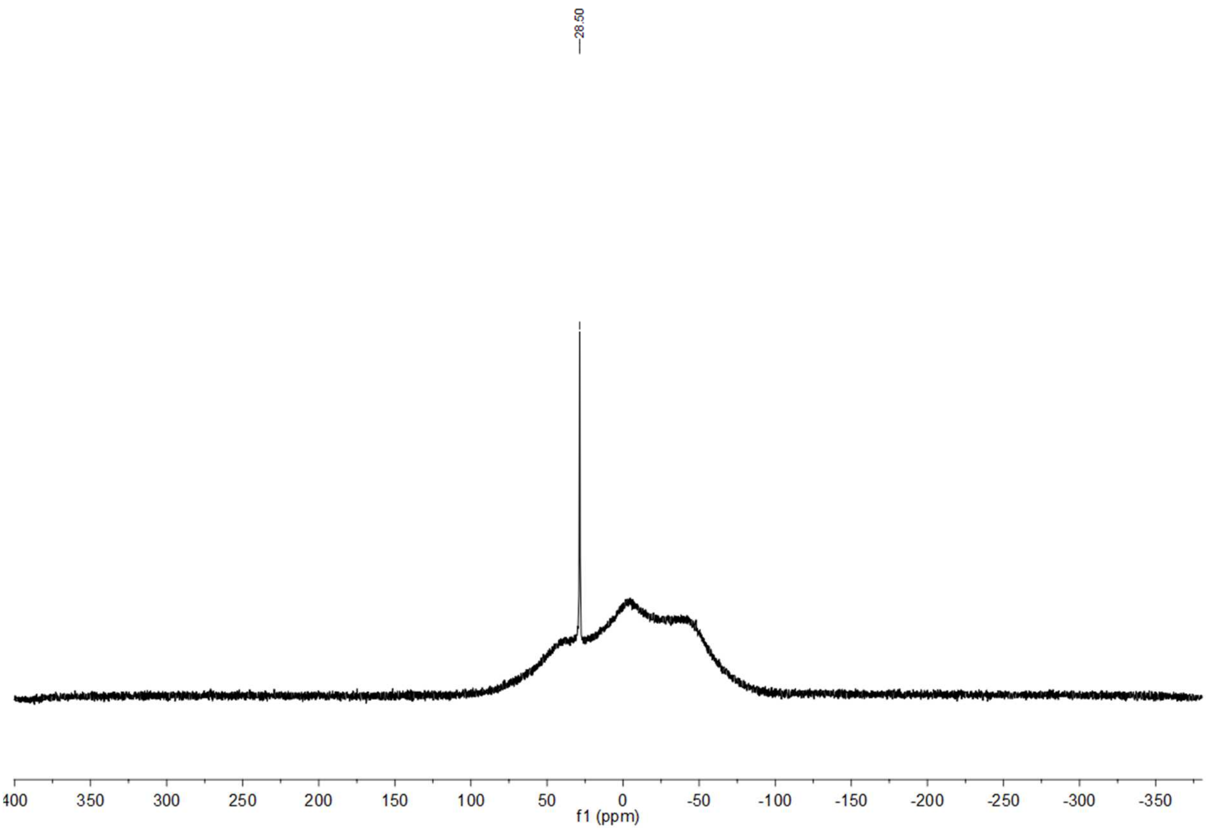

Figure S26. Crude ^{11}B NMR spectrum of **C** and HBPin showing no reaction in C_6D_6 at room temperature.

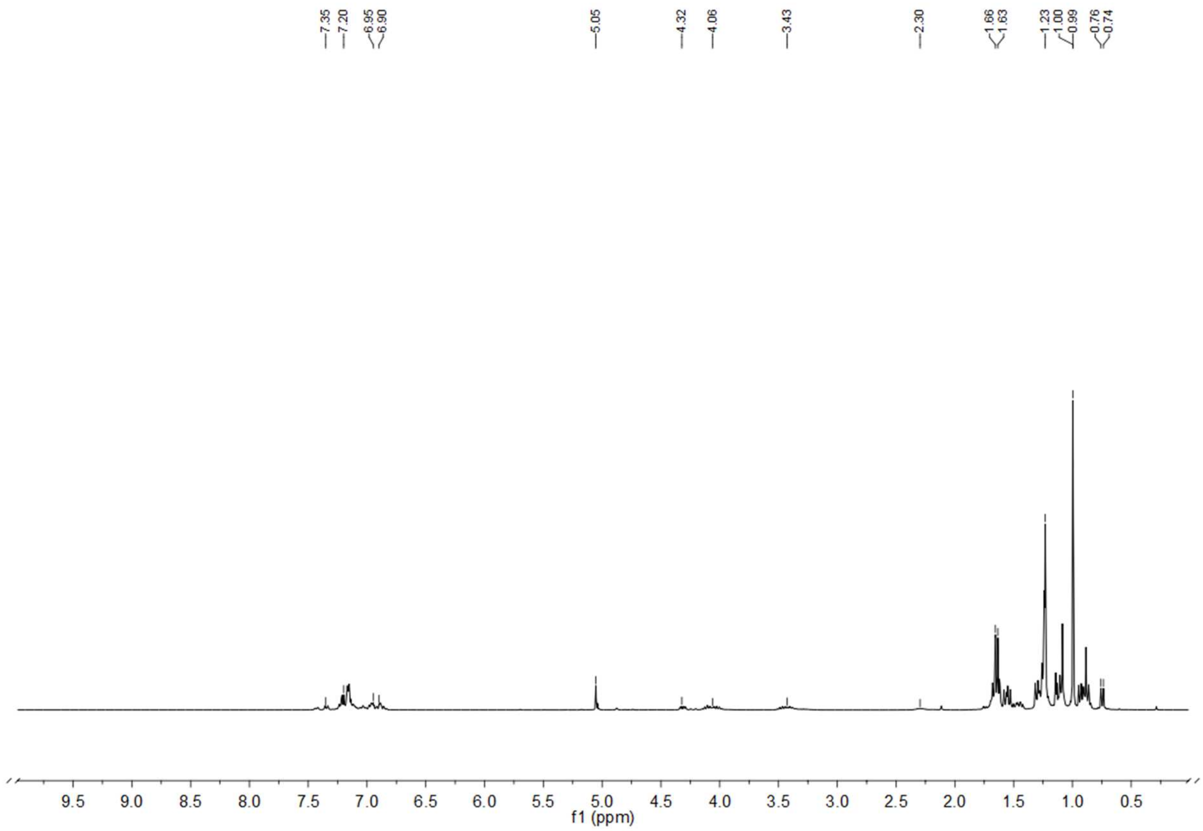

Figure S27. Crude ^{1}H NMR spectrum of **2** and HBPin showing no reaction in C_6D_6 at room temperature.

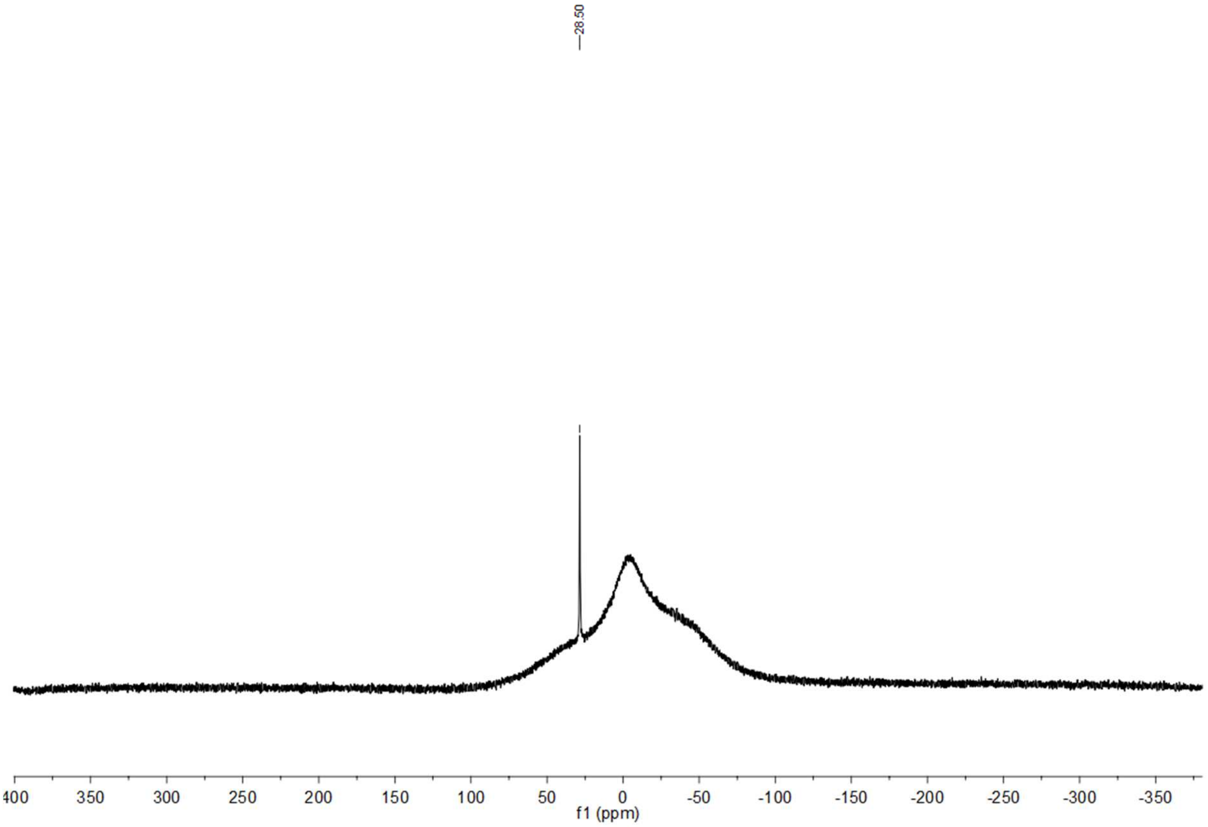

Figure S28. Crude ^{11}B NMR spectrum of **2** and HBPin showing no reaction in C_6D_6 at room temperature.

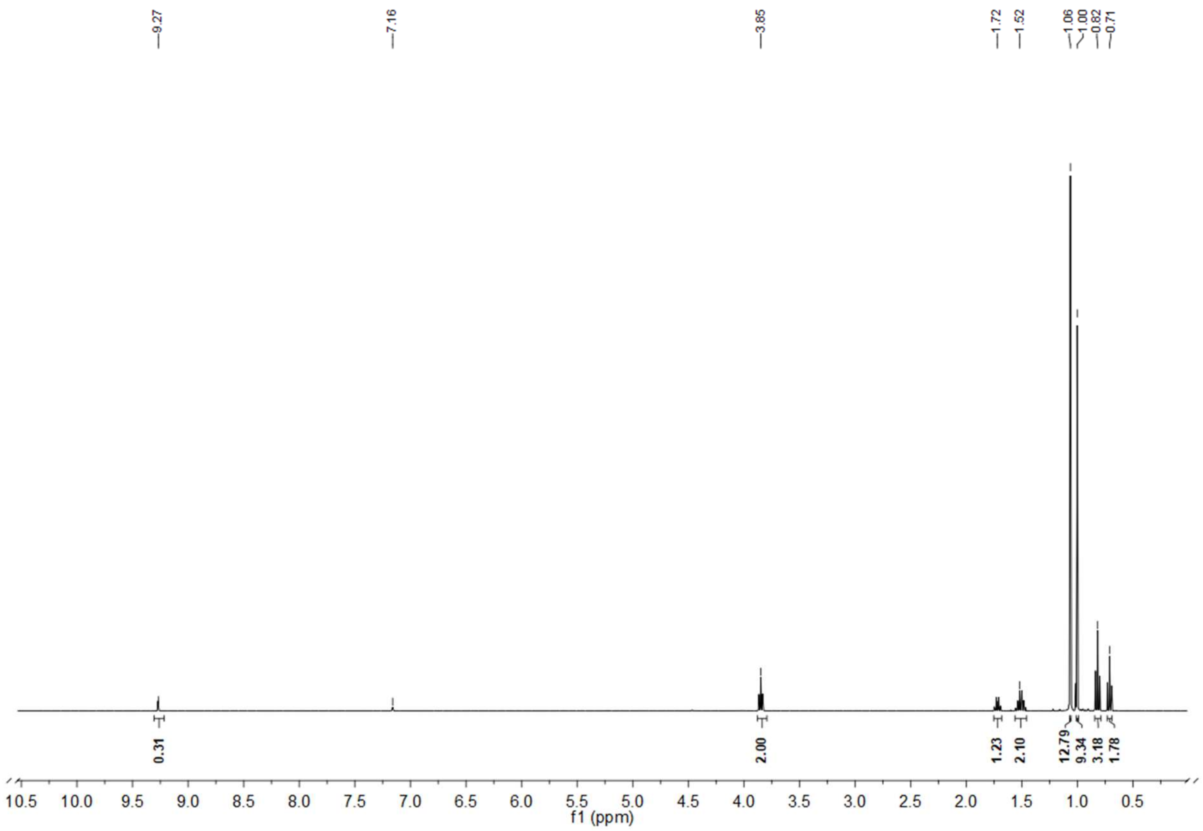

Figure S29. Crude ^{1}H NMR spectrum of the reaction (*n*-propanal and HBPin) without any catalyst in C_6D_6 at room temperature after 48 h.

II. Catalytic Studies

Table S1. Catalytic table for hydroboration of aldehyde and ketone derivatives using HBPin and 1 mol% of **2.**

Entry	Substrate	Product	t(h)	Yield (%)	TOF (h^{-1})
**1**	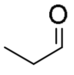	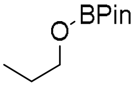	0.75	>99	133
**2**	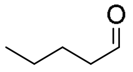	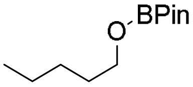	2.25	>99	44
**3**	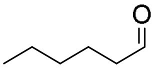	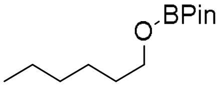	0.67	>99	150
**4**	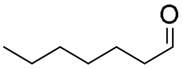	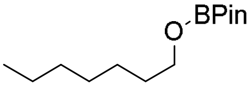	1.5	>99	66
**5**	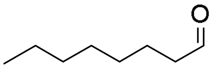	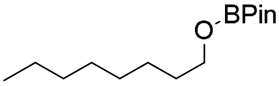	3.5	>99	29
**6**	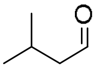	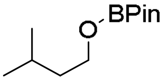	9	>99	11.1
**7**	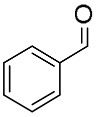	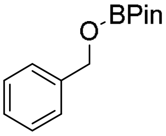	24	95	3.9
**8**	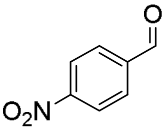	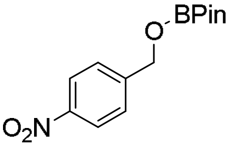	16	>99	6.25
**9**	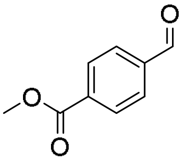	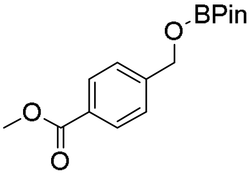	18	>99	5.6
**10**	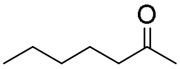	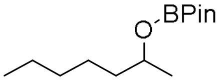	168	79	0.47
**11**	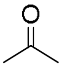	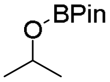	168	55	0.33
**12**	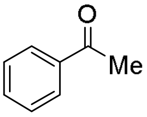	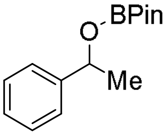	168	67	0.40

Characterization of products

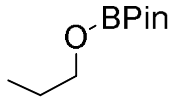

^{1}H NMR (400 MHz, C_6D_6): δ 3.84 (t, $^3J_{HH}$ = 6.6 Hz, 2H), 1.61 – 1.36 (m, 2H), 1.06 (s, 12H), 0.82 (t, $^3J_{HH}$ = 7.4 Hz, 3H).

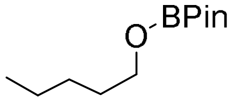

^{1}H NMR (300 MHz, C_6D_6): δ 3.92 (t, $^3J_{HH}$ = 6.5 Hz, 2H), 1.53 (m, 2H), 1.23 (m, 4H), 1.07 (s, 12H), 0.80 (t, $^3J_{HH}$ = 6.7 Hz, 3H).

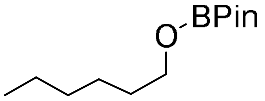

^{1}H NMR (300 MHz, C_6D_6): δ 3.93 (t, $^3J_{HH}$ = 6.5 Hz, 2H), 1.64 – 1.46 (m, 2H), 1.28 – 1.13 (m, 6H), 1.07 (s, 12H), 0.82 (t, $^3J_{HH}$ = 6.7 Hz, 3H).

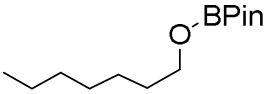

^{1}H NMR (300 MHz, C_6D_6): δ 3.94 (t, $^3J_{HH}$ = 6.5 Hz, 2H), 1.65 – 1.43 (m, 2H), 1.20 – 1.16 (m, 8H), 1.08 (s, 12H), 0.85 (t, $^3J_{HH}$ = 6.7 Hz, 3H).

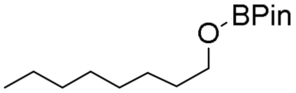

^{1}H NMR (300 MHz, C_6D_6): δ 3.94 (t, $^3J_{HH}$ = 6.5 Hz, 2H), 1.67 – 1.47 (m, 2H), 1.31 – 1.13 (m, 8H), 1.08 (s, 12H), 0.87 (t, $^3J_{HH}$ = 6.8 Hz, 3H).

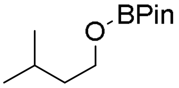

^{1}H NMR (300 MHz, C_6D_6): δ 3.97 (t, $^3J_{HH}$ = 6.6 Hz, 2H), 1.76 – 1.61 (m, 1H), 1.44 (q, $^3J_{HH}$ = 6.7 Hz, 2H), 1.07 (s, 12H), 0.82 (d, $^3J_{HH}$ = 6.6 Hz, 6H).

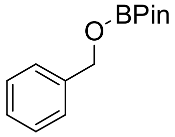

^{1}H NMR (300 MHz, C_6D_6): δ 7.60 – 6.76 (m, 5H), 4.95 (s, 2H), 1.04 (s, 12H).

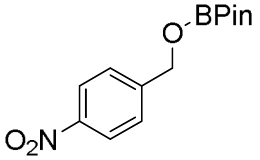

^{1}H NMR (300 MHz, C_6D_6): δ 7.80 (d, $^3J_{HH}$ = 8.7 Hz, 2H), 6.92 (d, $^3J_{HH}$ = 8.5 Hz, 2H), 4.69 (s, 2H), 1.05 (s, 12H).

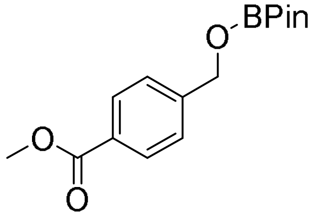

^{1}H NMR (300 MHz, C_6D_6) δ 8.06 (d, $^3J_{HH}$ = 8.2 Hz, 2H), 7.22 (d, $^3J_{HH}$ = 8.0 Hz, 2H), 4.85 (s, 2H), 3.49 (s, 3H), 1.04 (s, 12H).

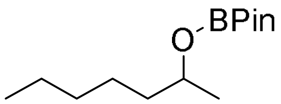

^{1}H NMR (300 MHz, C_6D_6): δ 4.35 – 4.31 (m, 1H), 1.47 – 1.35 (m, 6H), 1.20 (d, $^3J_{HH}$ = 6.2 Hz, 3H), 1.15 – 1.13 (m, 2H), 1.08 (s, 12H), 0.85 (t, $^3J_{HH}$ = 7.1 Hz, 3H).

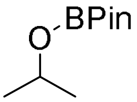

^{1}H NMR (300 MHz, C_6D_6): δ 4.61 – 4.26 (m, 1H), 1.16 (d, $^3J_{HH}$ = 6.2 Hz, 6H), 1.06 (s, 12H).

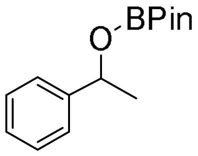

^{1}H NMR (300 MHz, C_6D_6): δ 7.43 – 7.30 (m, 2H), 7.17 – 7.12 (m, 2H), 7.06 – 7.00 (m, 1H), 5.41 (q, $^3J_{HH}$ = 6.5 Hz, 1H), 1.45 (d, $^3J_{HH}$= 6.5 Hz, 3H), 1.03 (s, 6H), 1.00 (s, 6H).

Kinetic Studies

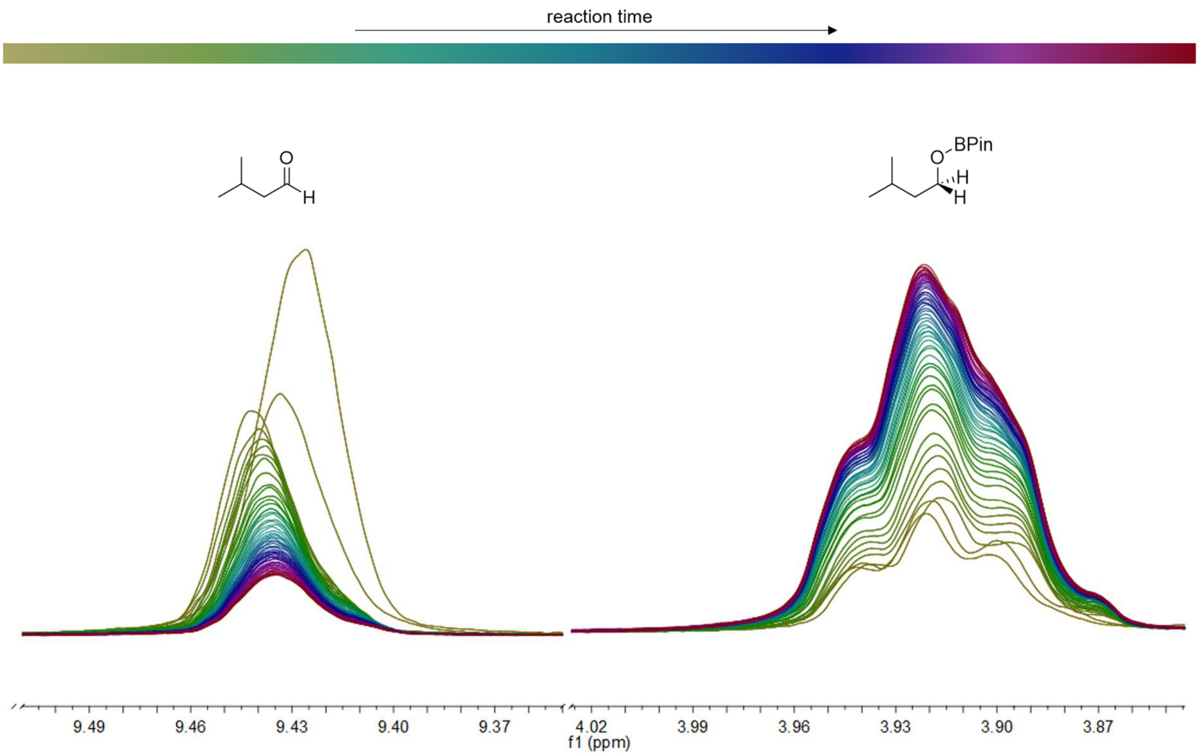

Figure S30. ^{1}H NMR spectra showing the conversion of *iso*-valeraldehyde in C_6D_6 at 80 °C.

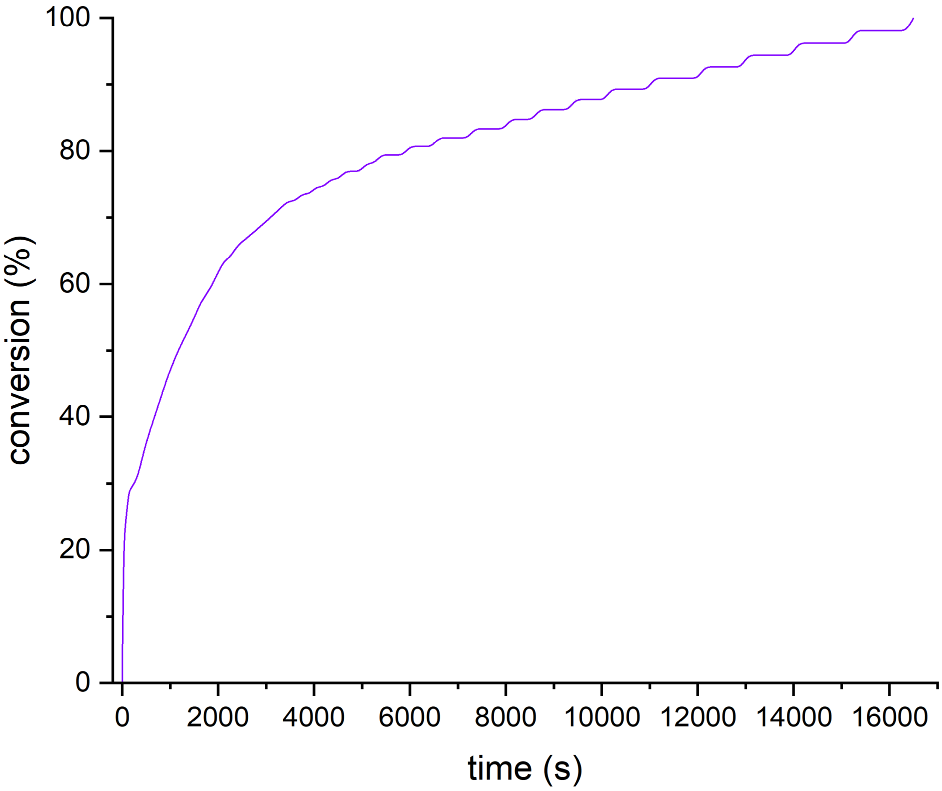

Figure S31. Plot of conversion against time during measurement of kinetics.

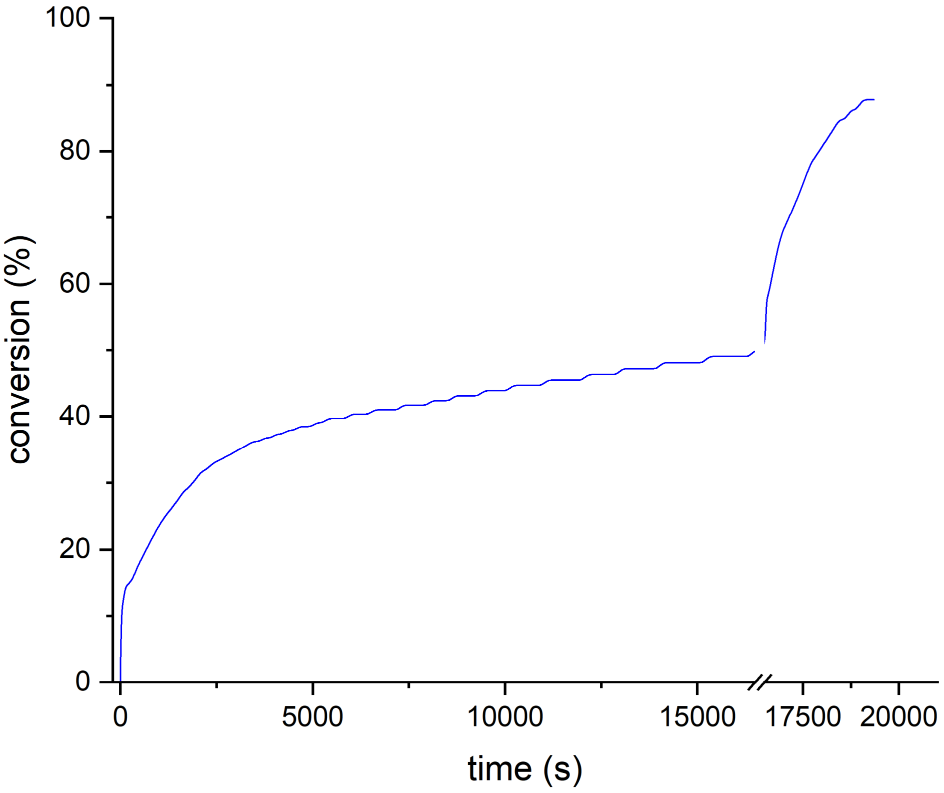

Figure S32. Plot of conversion against time during measurement of kinetics with addition of one equivalent *iso*-valeraldehyde and HBPin after 24 h.

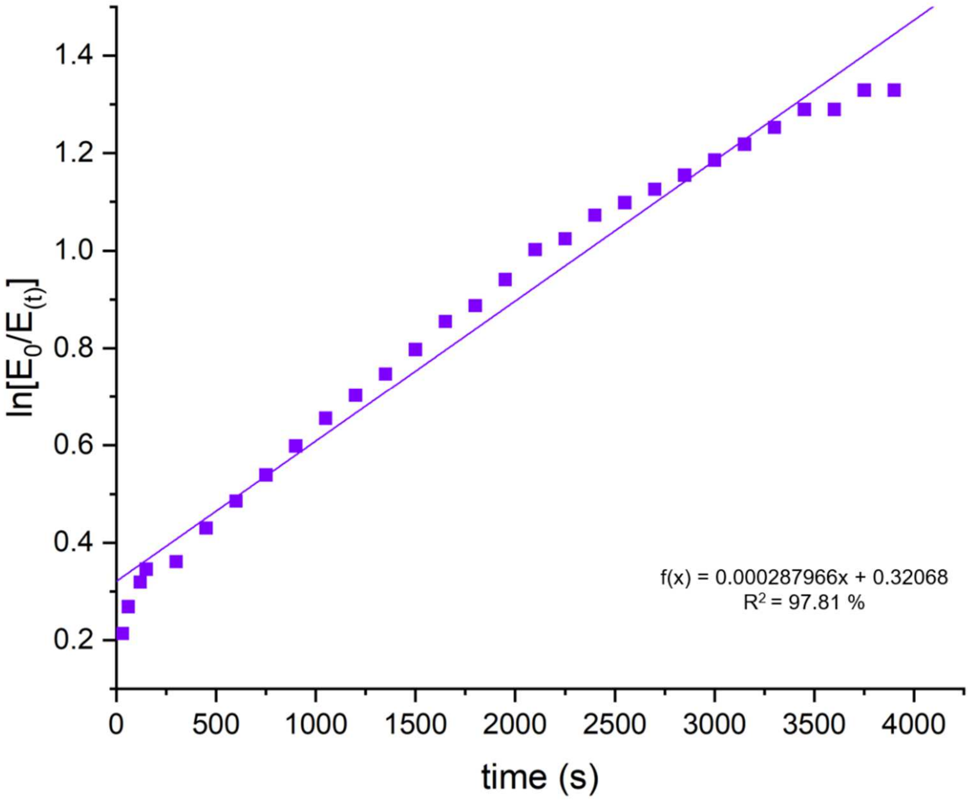

Figure S33. Plot of reaction kinetics (one equivalent *iso*-valeraldehyde and HBPin).

III. Single Crystal X-ray Diffraction

The crystals were mounted on nylon loops in inert oil. Data of **1** were collected on a Bruker AXS D8 Venture diffractometer with Photon II detector (monochromated $Cu_{K\alpha}$ radiation, λ = 1.54178 Å, microfocus source) at 100(2) K while those of **2** were collected on a Bruker AXS D8 Kappa diffractometer with APEX2 detector (monochromated $Mo_{K\alpha}$ radiation, λ = 0.71073 Å) at 100(2) K. The structures were solved by Direct Methods (SHELXS-2013)[1] and refined anisotropically by full-matrix least-squares on F^2 (SHELXL-2017).[2,3] Absorption corrections were performed semi-empirically from equivalent reflections on basis of multi-scans (Bruker AXS APEX3). Hydrogen atoms were refined using a riding model or rigid methyl groups.

Table S2. Crystal data and structure refinement of **1** and **2**.

Identification code	1 (lk_045m)	2 (lk_046m)
Empirical formula	$C_{58}H_{83}ClGaN_4OSi$	$C_{55}H_{88}ClGaN_4OSi$
M	985.54	954.55
Crystal size [mm]	0.174 × 0.161 × 0.077	0.300 × 0.234 × 0.056
T [K]	100(2)	100(2)
Crystal system	triclinic	triclinic
Space group	P-1	P-1
a [Å]	12.8260(8)	12.6656(7)
b [Å]	13.0023(8)	13.0233(7)
c [Å]	18.3120(10)	18.9626(11)
α [°]	83.087(2)	101.433(3)
β [°]	75.811(2)	102.306(3)
γ [°]	69.828(2)	111.151(2)
V [Å^3]	2776.9(3)	2717.1(3)
Z	2	2
D_{calc} [g·cm^{-3}]	1.179	1.167
$\mu(K_\alpha$ [mm^{-1}])	1.632 (Cu)	0.617 (Mo)
Transmissions	0.75/0.64	0.75/0.67
F(000)	1058	1032
Index ranges	$-16 \le h \le 16$	$-19 \le h \le 19$
	$-16 \le k \le 16$	$-20 \le k \le 20$
	$-23 \le l \le 20$	$-29 \le l \le 29$
θ_{max} [°]	80.466	33.267
Reflections collected	128716	229049
Independent reflections	12043	20905
R_{int}	0.0580	0.0495
Refined parameters	839	645
R_1 [$I > 2\sigma(I)$]	0.0393	0.0329
wR_2 [all data]	0.1094	0.0880
GooF	1.054	1.043
$\Delta\rho_{final}$ (max/min) [e·Å^{-3}]	0.975/-0.599	0.735/-0.603

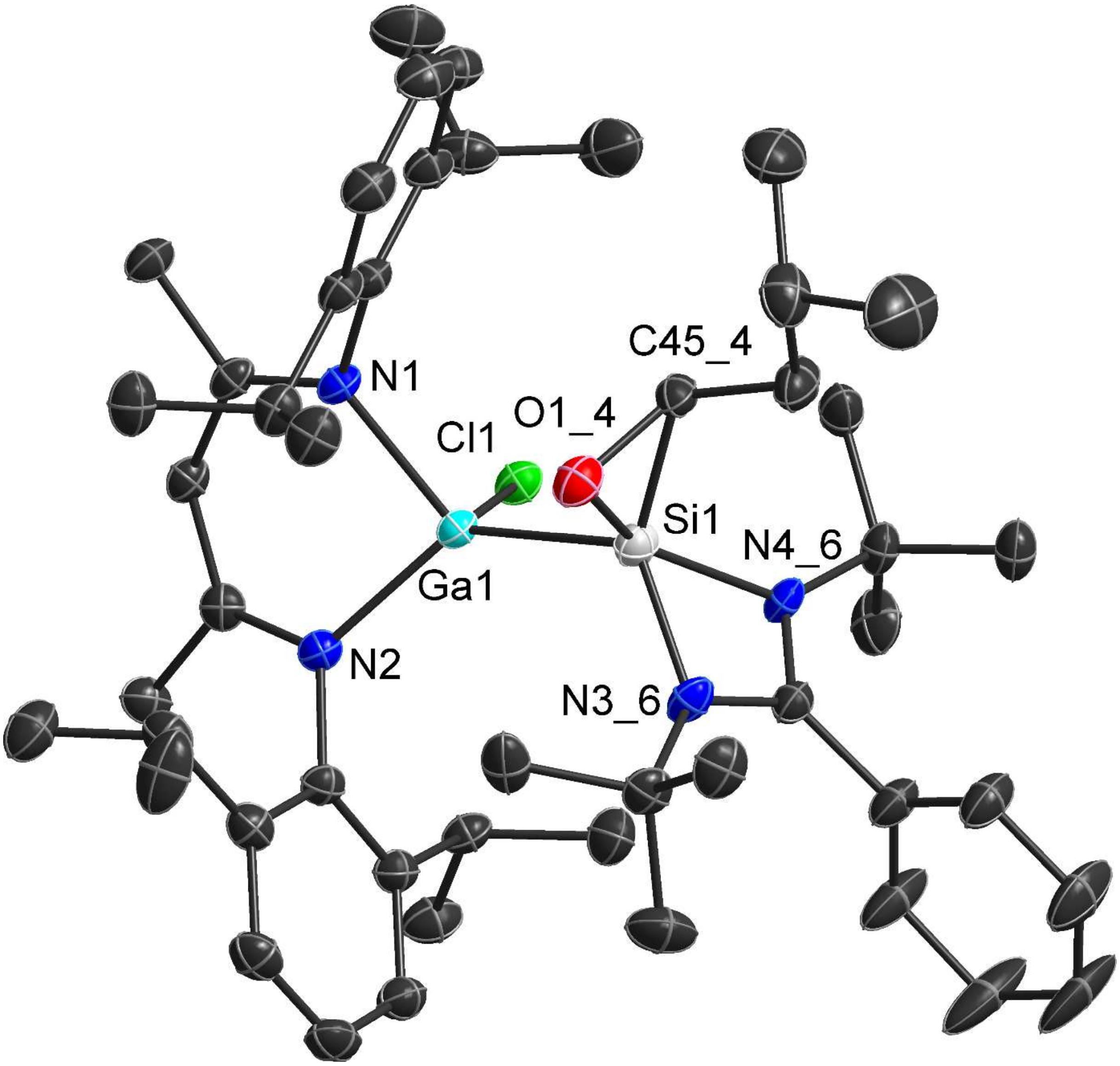

Figure S34. Molecular structure of **1** with thermal ellipsoids at 50% probability level. The hydrogen atoms, solvent molecules and the alternate position of the disordered parts are omitted for clarity. Selected bond length (Å) and angels (°): Ga(1)-N(2) 1.9614(14), Ga(1)-N(1) 1.9823(13), Ga(1)-Cl(1) 2.2845(4), Ga(1)-Si(1) 2.4054(5), Si(1)-C45_5 1.654(18), Si(1)-O1_5 1.730(8), Si(1)-O1_4 1.7488(16), Si(1)-N3_6 1.818(7), Si(1)-C45_4 1.835(3), Si(1)-N4_6 1.939(4), Si(1)-N3_7 1.94(3), Si(1)-N4_7 2.069(16), Si(1)-C30_6 2.340(4), N(2)-Ga(1)-N(1) 94.20(6), N(2)-Ga(1)-Cl(1) 99.43(4), N(1)-Ga(1)-Cl(1) 99.71(4), N(2)-Ga(1)-Si(1) 130.11(4), N(1)-Ga(1)-Si(1) 115.99(4), Cl(1)-Ga(1)-Si(1) 112.242(18), C45_5-Si(1)-O1_5 53.7(5), O1_4-Si(1)-N3_6 100.4(2), O1_4-Si(1)-C45_4 49.52(11), N3_6-Si(1)-C45_4 122.9(4), O1_4-Si(1)-N4_6 147.19(19), N3_6-Si(1)-N4_6 69.17(18), C45_4-Si(1)-N4_6 108.9(2), C45_5-Si(1)-N3_7 111.0(14), O1_5-Si(1)-N3_7 142.8(12), C45_5-Si(1)-N4_7 118.5(10), O1_5-Si(1)-N4_7 92.1(6), N3_7-Si(1)-N4_7 64.3(7), O1_4-Si(1)-C30_6 127.26(16), N3_6-Si(1)-C30_6 35.16(16), C45_4-Si(1)-C30_6 119.64(18), N4_6-Si(1)-C30_6 34.12(11), C45_5-Si(1)-Ga(1) 121.2(7), O1_5-Si(1)-Ga(1) 100.0(3), O1_4-Si(1)-Ga(1) 103.62(6), N3_6-Si(1)-Ga(1) 119.9(3), C45_4-Si(1)-Ga(1) 114.48(9), N4_6-Si(1)-Ga(1) 108.51(17), N3_7-Si(1)-Ga(1) 115.4(13), N4_7-Si(1)-Ga(1) 113.1(7), C30_6-Si(1)-Ga(1) 121.66(16).

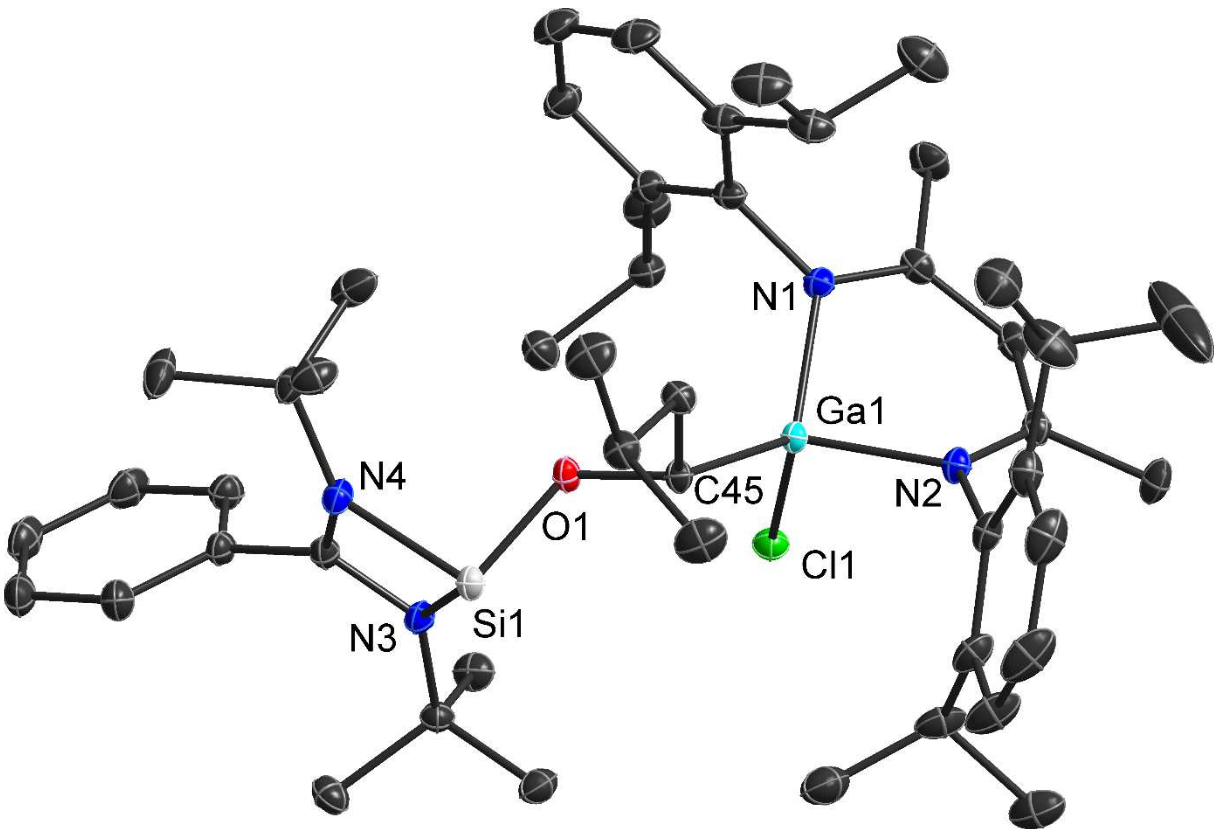

Figure S35. Molecular structure of **2** with thermal ellipsoids at 50% probability level. The hydrogen atoms and the disordered solvent molecule are omitted for clarity. Selected bond length (Å) and angels (º): Ga(1)-N(1) 1.9444(9), Ga(1)-N(2) 1.9500(9), Ga(1)-C(45) 1.9662(10), Ga(1)-Cl(1) 2.2347(3), Si(1)-O(1) 1.6849(8), Si(1)-N(4) 1.8748(9), Si(1)-N(3) 1.9203(9), Si(1)-C(30) 2.3467(11), O(1)-C(45) 1.4390(12), N(1)-Ga(1)-N(2) 96.05(4), N(1)-Ga(1)-C(45) 124.42(4), N(2)-Ga(1)-C(45) 116.18(4), N(1)-Ga(1)-Cl(1) 102.48(3), N(2)-Ga(1)-Cl(1) 103.15(3) ,C(45)-Ga(1)-Cl(1) 111.56(3), O(1)-Si(1)-N(4) 96.60(4), O(1)-Si(1)-N(3) 103.59(4), N(4)-Si(1)-N(3) 69.06(4), O(1)-Si(1)-C(30) 105.76(4), N(4)-Si(1)-C(30) 34.66(4), N(3)-Si(1)-C(30) 34.72(4), C(45)-O(1)-Si(1) 121.71(6).

IV. Computational Details

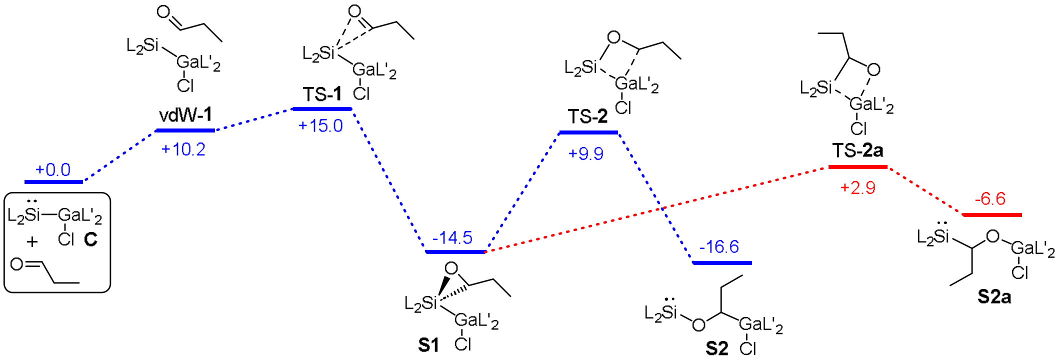

Figure S36. Gibbs energies (G) for the formation of the catalyst **S2** starting from **C** and propanal calculated by means of PBE0-D3BJ(SMD,benzene as solvent)/def2-TZVP//PBE0-D3BJ/def2-SVP. The values are given in kcal/mol. Half-life for **C** at -80 °C: 4.5 h. Half-life for **S1** at RT: 23.8 h.

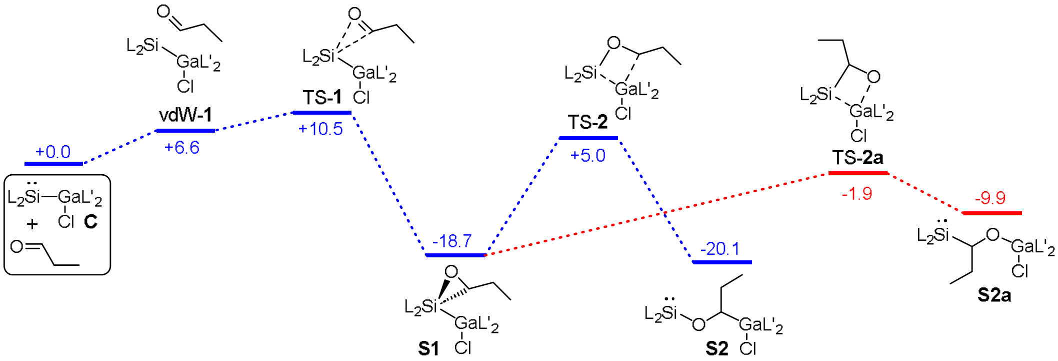

Figure S37. Gibbs energies ($G_{70\%}$) for the formation of the catalyst **S2** starting from **C** and propanal calculated by means of PBE0-D3BJ(SMD,benzene as solvent)/def2-TZVP//PBE0-D3BJ/def2-SVP. Herein $G_{70\%}$ means that 70% calculated gas-phase entropy contributes to solution-phase free energy. The values are given in kcal/mol. Half-life for **C** at -80 °C: 0.13 s. Half-life for **S1** at RT: 7.3 h.

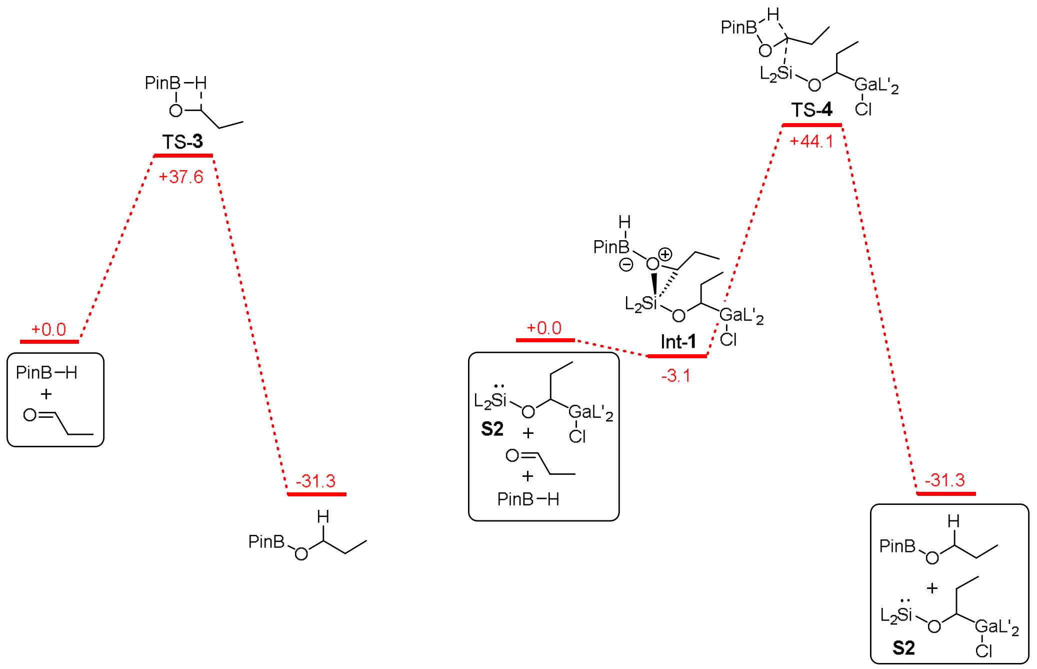

Figure S38. Gibbs energies (G) for the hydroboration of propanal without (left) and with **S2** as catalyst (right) calculated by means of PBE0-D3BJ(SMD,benzene as solvent)/def2-TZVP//PBE0-D3BJ/def2-SVP. The values are given in kcal/mol.

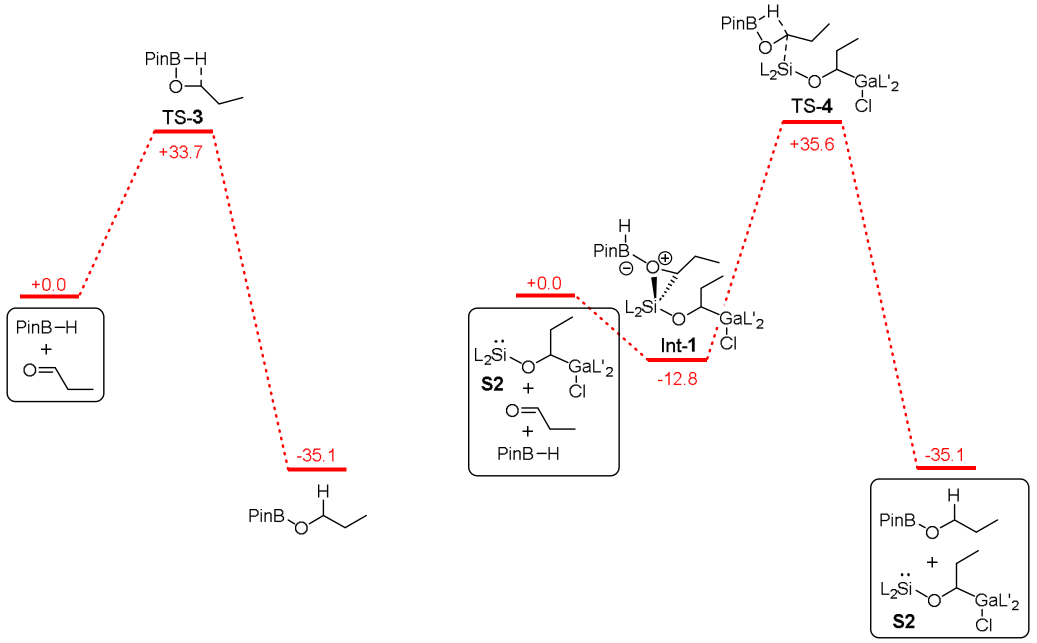

Figure S39. Gibbs energies ($G_{70\%}$) for the hydroboration of propanal without (left) and with **S2** as catalyst (right) calculated by means of PBE0-D3BJ(SMD,benzene as solvent)/def2-TZVP//PBE0-D3BJ/def2-SVP. Herein $G_{70\%}$ means that 70% calculated gas-phase entropy contributes to solution-phase free energy. The values are given in kcal/mol.

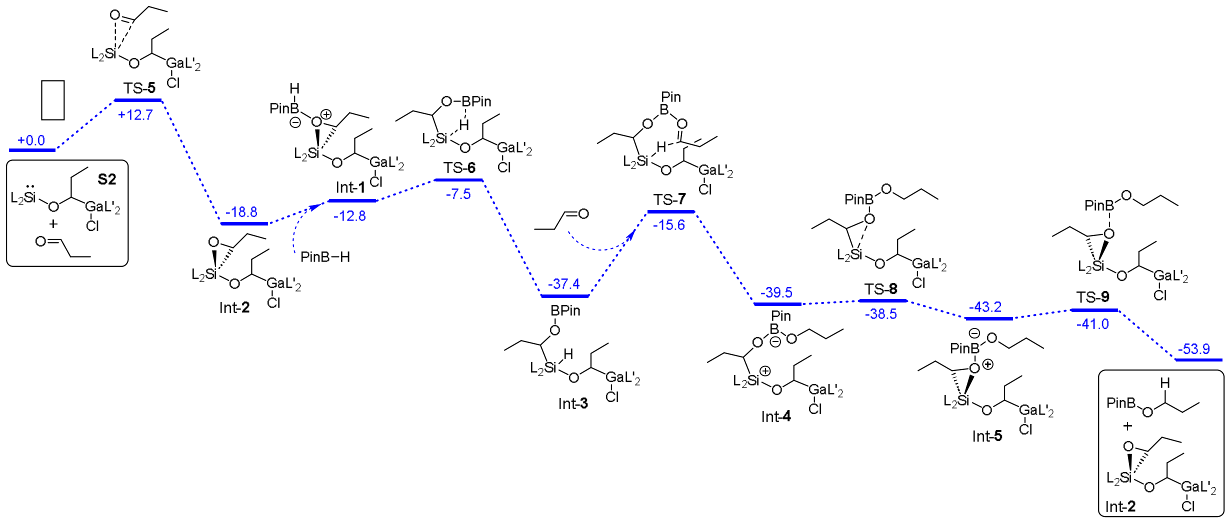

Figure S40. Gibbs energies ($G_{70\%}$) for the hydroboration of propanal with **Int-2** as catalytically active species calculated by means of PBE0-D3BJ(SMD,benzene as solvent)/def2-TZVP//PBE0-D3BJ/def2-SVP. Herein $G_{70\%}$ means that 70% calculated gas-phase entropy contributes to solution-phase free energy. The values are given in kcal/mol. The turnover frequencies (TOF) of the catalytic reaction was estimated to be 0.9 h^{-1}.

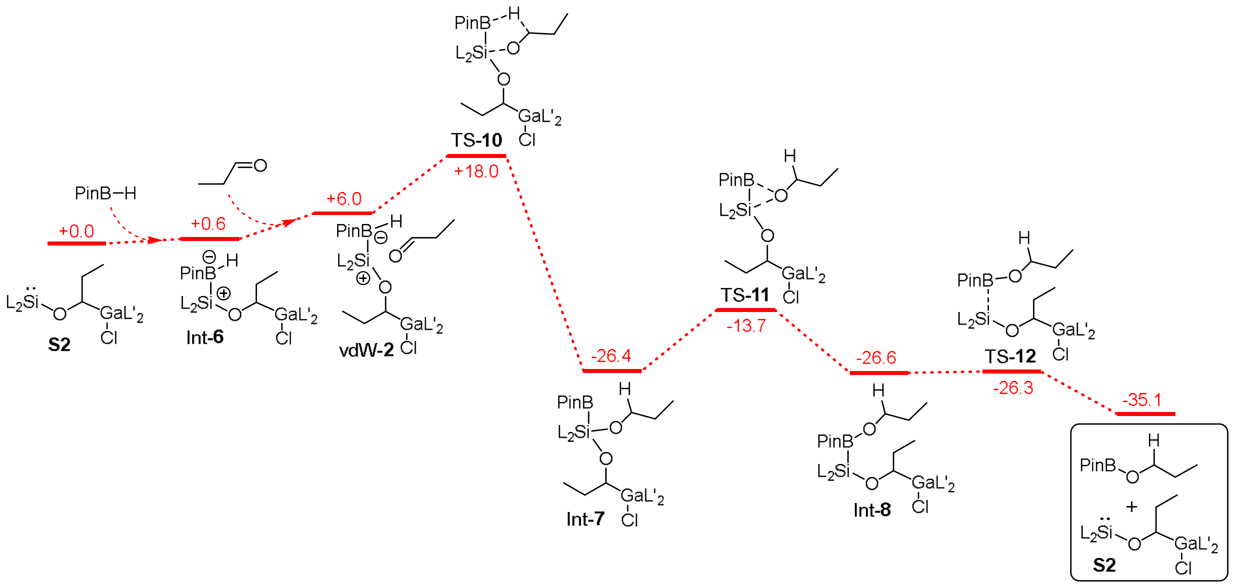

Figure S41. Gibbs energies ($G_{70\%}$) for the hydroboration of propanal with **S2** as catalytically active species calculated by means of PBE0-D3BJ(SMD,benzene as solvent)/def2-TZVP//PBE0-D3BJ/def2-SVP. Herein $G_{70\%}$ means that 70% calculated gas-phase entropy contributes to solution-phase free energy. The values are given in kcal/mol. The turnover frequencies (TOF) of the catalytic reaction was estimated to be 217 h^{-1}.

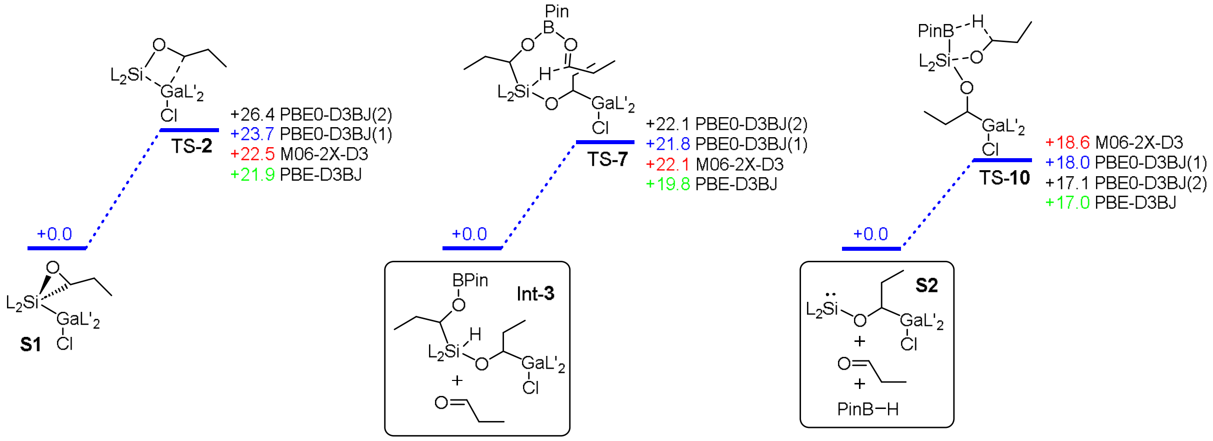

Figure S42. Gibbs energies ($G_{70\%}$) for the formation of the transition states TS-**2**, TS-**7** and TS-**10** using different methods. PBE0-D3BJ(1): PBE0-D3BJ(SMD,benzene as solvent)/def2-TZVP//PBE0-D3BJ/def2-SVP. PBE0-D3BJ(2): PBE0-D3BJ(SMD,benzene as solvent)/6-311++G**//PBE0-D3BJ/6-31G*. PBE-D3BJ: PBE-D3BJ(SMD,benzene as solvent)/def2-TZVP//PBE-D3BJ/def2-SVP. M06-2X-D3: M06-2X-D3(SMD,benzene as solvent)/def2-TZVP//M06-2X-D3/def2-SVP. Herein $G_{70\%}$ means that 70% calculated gas-phase entropy contributes to solution-phase free energy. The values are given in kcal/mol.

VI. Absolute Energies and Cartesian Coordinates for the Calculated Compounds

Table S3. Absolute energies [au] and entropies [cal/mol*K] calculated by means of different methods.

Compound	*E*[a]	*G*[a]	*S*[a]	*E*[b]
EtCHO	-192.778690	-192.721753	70.861	-193.001451
HBPin	-411.105949	-410.948011	91.247	-411.561149
TS-**3**	-603.852407	-603.616179	118.547	-604.524119
$EtCH_2OBPin$	-603.971179	-603.729839	119.732	-604.638998
**C**	-4604.456878	-4603.547267	311.816	-4606.992265
vdW-**1**	-4797.248939	-4796.260787	342.621	-4799.999132
TS-**1**	-4797.244634	-4796.252872	332.826	-4799.995088
**S1**	-4797.287210	-4796.295144	335.018	-4800.042288
TS-**2**	-4797.249612	-4796.254945	327.595	-4800.006015
**S2**	-4797.286449	-4796.298766	342.711	-4800.041229
TS-**2a**	-4797.265978	-4796.271915	328.514	-4800.016603
**S2a**	-4797.272827	-4796.286397	345.367	-4800.024117
Int-**1**	-5401.250230	-5399.989266	395.993	-5404.667150
TS-**4**	-5401.168024	-5399.918317	410.569	-5404.580783
TS-**5**	-4990.070519	-4989.000520	363.883	-4993.040722
Int-**2**	-4990.119669	-4989.045719	360.366	-4993.094326
Int-**1**	-5401.250230	-5399.989266	395.993	-5404.667150
TS-**6**	-5401.237735	-5399.979413	398.508	-5404.656359
Int-**3**	-5401.290720	-5400.033527	404.532	-5404.703831
TS-**7**	-5594.066780	-5592.726567	420.522	-5597.688790
Int-**4**	-5594.109317	-5592.766974	426.164	-5597.729813
TS-**8**	-5594.106712	-5592.765084	424.968	-5597.727335
Int-**5**	-5594.115290	-5592.771475	423.238	-5597.736653
TS-**9**	-5594.107205	-5592.764653	424.327	-5597.732014
Int-**6**	-5208.415676	-5207.247691	389.840	-5211.617456
vdW-**2**	-5401.217749	-5399.969237	417.067	-5404.627760
TS-**10**	-5401.205290	-5399.950093	400.653	-5404.612893
Int-**7**	-5401.273700	-5400.015291	406.107	-5404.687659
TS-**11**	-5401.256097	-5399.998541	402.854	-5404.666096
Int-**8**	-5401.276990	-5400.021508	410.258	-5404.685587
TS-**12**	-5401.275212	-5400.019628	408.351	-5404.685032

[a] PBE0-D3BJ/def2-SVP.
[b] PBE0-D3BJ(benzene as solvent)/def2-TZVP//PBE0-D3BJ/def2-SVP

Table S4. Absolute energies [au] and entropies [cal/mol*K] calculated by means of different methods.

Compound	*E*[a]	*G*[a]	*S*[a]	*E*[b]
**S1**	-4799.146483	-4798.151451	329.558	-4802.032553
TS-**2**	-4799.111755	-4798.112988	320.295	-4801.999197
**S2**	-4799.152481	-4798.158831	331.254	-4802.037580
EtCHO	-192.899670	-192.842530	70.967	-193.132904
HBPin	-411.378295	-411.219720	90.855	-411.856647
Int-**3**	-5403.550756	-5402.287631	393.647	-5407.130473
TS-**7**	-5596.446609	-5595.101878	412.872	-5600.245183
TS-**10**	-5403.454490	-5402.195437	393.283	-5407.032948

[a] M06-2X-D3/def2-SVP.
[b] M06-2X-D3(benzene as solvent)/def2-TZVP//M06-2X-D3/def2-SVP

Table S5. Absolute energies [au] and entropies [cal/mol*K] calculated by means of different methods.

Compound	*E*[a]	*G*[a]	*S*[a]	*E*[b]
**S1**	-4796.893165	-4795.939746	344.664	-4799.676775
TS-**2**	-4796.859521	-4795.903334	336.543	-4799.643448
**S2**	-4796.892733	-4795.943940	352.659	-4799.675916
EtCHO	-192.761707	-192.707778	71.213	-192.987181
HBPin	-411.053658	-410.902369	92.524	-411.513971

Int-**3**	-5400.818957	-5399.609518	414.273	-5404.266670
TS-**7**	-5593.580436	-5592.292948	434.313	-5597.239156
TS-**10**	-5400.745505	-5399.539397	412.691	-5404.187246

[a] PBE-D3BJ/def2-SVP.
[b] PBE-D3BJ(benzene as solvent)/def2-TZVP//PBE-D3BJ/def2-SVP

Table S6. Absolute energies [au] and entropies [cal/mol*K] calculated by means of different methods.

Compound	*E*[a]	*G*[a]	*S*[a]	*E*[b]
**S1**	-4797.300523	-4796.297623	332.883	-4799.788435
TS-**2**	-4797.267442	-4796.259107	320.439	-4799.750002
**S2**	-4797.297649	-4796.297480	337.527	-4799.789521
EtCHO	-192.918483	-192.860668	70.868	-192.982215
HBPin	-411.403217	-411.243289	91.160	-411.518100
Int-**3**	-5401.735365	-5400.464376	401.773	-5404.389315
TS-**7**	-5594.649671	-5593.294359	417.095	-5597.354841
TS-**10**	-5401.649643	-5400.379891	396.714	-5404.299852

[a] PBE0-D3BJ/6-31G*.
[b] PBE0-D3BJ(benzene as solvent)/6-311++G**//PBE0-D3BJ/6-31G*

Cartesian coordinates of the optimized geometry of EtCHO at PBE0-D3BJ/def2-SVP level of theory:

O	-1.83066500	-0.00493300	-0.26491500
C	-0.78014400	-0.27264400	0.25134400
H	-0.66585400	-1.20988600	0.86363600
C	0.46600500	0.56499600	0.16592900
H	0.59463800	1.03399000	1.15956400
H	0.28299300	1.37518800	-0.55593100
C	1.70465600	-0.25264900	-0.17614200
H	1.62643600	-0.69781500	-1.17976700
H	2.60799600	0.37345500	-0.15770300
H	1.85601100	-1.07368200	0.54273800

Cartesian coordinates of the optimized geometry of HBPin at PBE0-D3BJ/def2-SVP level of theory:

C	0.78343000	-0.18432800	0.05278800
C	-0.78345900	-0.18424500	-0.05281800
O	-1.06701400	1.18549000	-0.39617600
O	1.06710600	1.18545400	0.39610400
B	0.00006900	1.93122300	-0.00002800
H	0.00008200	3.13266800	-0.00010600
C	-1.47055400	-0.45411300	1.28063500
H	-2.54036000	-0.22220700	1.17979700
H	-1.36918300	-1.50514600	1.58664400
H	-1.05941600	0.18399400	2.07634000
C	-1.35088800	-1.09010400	-1.12866400
H	-1.08066800	-2.13967400	-0.93796800
H	-2.44780400	-1.01494500	-1.13268700
H	-0.99010700	-0.80833300	-2.12581200
C	1.35074600	-1.09010000	1.12873800
H	1.08043700	-2.13965900	0.93810400
H	2.44766800	-1.01503900	1.13279900
H	0.98993800	-0.80822900	2.12584800
C	1.47059100	-0.45429000	-1.28060700
H	2.54040900	-0.22246200	-1.17972800
H	1.36916800	-1.50533600	-1.58655400
H	1.05955000	0.18378600	-2.07638500

Cartesian coordinates of the optimized geometry of $EtCH_2OBPin$ at PBE0-D3BJ/def2-SVP level of theory:

O	-1.68618400	-0.20898700	-1.11855700
C	-2.64967200	0.76082400	-0.75743800
H	-3.41710400	0.75227500	-1.54896500
C	-3.28265800	0.45839700	0.58901100
H	-4.00598100	1.26247500	0.80913700
H	-2.49984300	0.53096500	1.36314900
C	-3.96499800	-0.89818800	0.64980400
H	-3.25330000	-1.70596100	0.42326000
H	-4.39055800	-1.08959300	1.64624400
H	-4.78461700	-0.96650700	-0.08368200
H	-2.19062800	1.76363000	-0.73987800
C	1.43120200	0.75286300	0.28876200
C	1.64761700	-0.76095300	-0.07140900
B	-0.42962900	-0.11727900	-0.63775600
O	0.00334000	0.87744500	0.20646000
O	0.54621500	-1.02490900	-0.95087900
C	1.48831700	-1.67810200	1.13621100
H	1.42279100	-2.71694600	0.78285000
H	2.33807700	-1.59762700	1.82916100
H	0.56417900	-1.44866100	1.68723200
C	2.94486400	-1.06304600	-0.79612500
H	3.81260800	-0.78588600	-0.17861000
H	3.00615900	-2.14022400	-1.00817000
H	3.00502400	-0.52825400	-1.75243100
C	1.88395500	1.14938900	1.68038900
H	2.96338800	0.97552800	1.80496300
H	1.68981600	2.21974400	1.84165400
H	1.34423900	0.58831100	2.45375800
C	2.02500500	1.69483100	-0.75210400
H	1.64640400	2.71029200	-0.56680400
H	3.12307300	1.71891900	-0.70436200
H	1.72565300	1.39944100	-1.76852900

Cartesian coordinates of the optimized geometry of **C** at PBE0-D3BJ/def2-SVP level of theory:

Ga	0.64267600	0.17658600	-0.35516700
Cl	-0.06738400	-0.65891000	-2.34510400
Si	-0.64225600	-0.56587800	1.66755500
N	2.60557100	-0.31001300	-0.45878600
N	0.99176200	2.08204100	-0.82912600
C	3.47533900	0.40662100	-1.15566000
C	3.16931500	1.63473500	-1.76917100
H	3.96768800	2.07305100	-2.36660500
C	2.05790600	2.44797500	-1.54665000
C	4.90251800	-0.04707700	-1.31490200
H	5.05001800	-0.43857300	-2.33278200
H	5.17797000	-0.83402100	-0.60359400
H	5.58190300	0.80720900	-1.19281700
C	2.11559100	3.84283800	-2.10589600
H	3.02311500	3.98727900	-2.70346400
H	2.09899500	4.58934300	-1.29850200
H	1.23599700	4.04695700	-2.73189200
C	3.01292800	-1.52548500	0.16362900
C	3.22676600	-2.69123800	-0.60499400
C	3.58925300	-3.86461800	0.06531000
H	3.75647000	-4.77601300	-0.51399600
C	3.72652200	-3.89819300	1.44575000
H	4.00174900	-4.82858700	1.94809100
C	3.51299800	-2.74155900	2.18857500
H	3.62719400	-2.77342600	3.27291600
C	3.16231600	-1.53964100	1.57024300
C	3.07732400	-2.73457700	-2.11670700
H	2.83801600	-1.71968800	-2.46637700
C	4.36898900	-3.19713100	-2.79601400
H	4.57394900	-4.25807600	-2.58172500
H	5.24591000	-2.62245000	-2.46590700
H	4.28387300	-3.09609900	-3.88916400

C	1.91439800	-3.62857300	-2.54698500
H	0.95904600	-3.24739500	-2.16838100
H	2.04978000	-4.66292700	-2.19174500
H	1.84446200	-3.65541500	-3.64549500
C	3.01419100	-0.26515200	2.38120100
H	2.20283400	0.31915700	1.91754700
C	4.28562200	0.58293700	2.30223100
H	5.14626300	0.03612500	2.71962900
H	4.16467100	1.51476400	2.87714000
H	4.53093700	0.86089900	1.26798600
C	2.61402500	-0.50805300	3.83075000
H	3.41532900	-1.00122900	4.40392000
H	1.70103600	-1.11824500	3.89377200
H	2.40492200	0.45069000	4.32713200
C	0.08865300	3.10919000	-0.42812700
C	-1.00916500	3.45701200	-1.24306100
C	-1.84229300	4.49891300	-0.82297800
H	-2.69866900	4.77908500	-1.44126100
C	-1.59767400	5.18709900	0.36033200
H	-2.25914300	6.00028900	0.66837500
C	-0.51216600	4.83193900	1.15309200
H	-0.32540500	5.37202100	2.08494700
C	0.34549800	3.79361700	0.77959400
C	-1.29585600	2.73487000	-2.54501300
H	-0.44566000	2.06836100	-2.75078100
C	-1.44719500	3.69242100	-3.72597900
H	-2.33607800	4.33478100	-3.62182200
H	-1.56051300	3.12533600	-4.66273400
H	-0.57414700	4.35380000	-3.83680700
C	-2.52536500	1.84000400	-2.40922000
H	-2.40987100	1.14685200	-1.56482800
H	-2.66694300	1.23944100	-3.32073300
H	-3.43683600	2.43857500	-2.24610700
C	1.53318300	3.44449900	1.65858800
H	2.08496800	2.63449000	1.15953100
C	2.49293800	4.62569600	1.80515700
H	3.38434300	4.32854300	2.37955600
H	2.02108000	5.46789800	2.33591800
H	2.83241000	4.99697500	0.82663100
C	1.09311700	2.92232500	3.02527900
H	1.97020200	2.71396400	3.65725700
H	0.51872900	1.98746600	2.93633300
H	0.46654800	3.65585200	3.55706800
N	-2.39961600	-0.05207900	1.08609100
N	-1.56256900	-2.00656800	0.81100200
C	-2.67545700	-1.29852000	0.67139200
C	-3.96325100	-1.75838500	0.09433300
C	-4.94004600	-2.41246400	0.85036100
H	-4.76235600	-2.61647600	1.90818300
C	-6.14220900	-2.79552400	0.25795800
H	-6.90358100	-3.30238400	0.85532400
C	-6.37230800	-2.53177200	-1.09125000
H	-7.31542600	-2.83244400	-1.55366400
C	-5.39571100	-1.88686500	-1.85065700
H	-5.56918200	-1.68420000	-2.90999400
C	-4.19540800	-1.50015300	-1.26234500
H	-3.41853200	-1.00493800	-1.84915000
C	-1.29557400	-3.41830400	0.53248800
C	0.19666400	-3.61561100	0.75982200
H	0.47881500	-3.34150000	1.78811000
H	0.77802300	-2.99463600	0.06862300
H	0.48320000	-4.66316700	0.59337900
C	-2.06669200	-4.29931200	1.52112900
H	-1.84431800	-3.99823100	2.55611500
H	-1.77304000	-5.35273700	1.39541500
H	-3.15103700	-4.23254900	1.35843000
C	-1.65527100	-3.78708400	-0.90847900
H	-2.74258800	-3.80657200	-1.06395400
H	-1.26408200	-4.78836600	-1.14403000

H	-1.21691900	-3.06120100	-1.60818900
C	-3.33493400	0.91207900	1.68361800
C	-3.86712400	0.35142000	3.00778000
H	-3.02951900	0.07405200	3.66624700
H	-4.48974400	-0.53946500	2.84028400
H	-4.48574300	1.10091600	3.52521700
C	-4.50151900	1.26449800	0.75912100
H	-5.08422400	2.08025800	1.21323200
H	-5.18150000	0.41793200	0.59839500
H	-4.13606100	1.60829900	-0.21661600
C	-2.52404000	2.16970000	1.96081800
H	-1.68142300	1.94491500	2.63243200
H	-3.14559200	2.93785100	2.44258700
H	-2.12497200	2.58294500	1.02753400

Cartesian coordinates of the optimized geometry of **S1** at PBE0-D3BJ/def2-SVP level of theory:

Ga	0.89242400	0.20118200	-0.80136900
Cl	0.74648400	-0.18100900	-3.02284500
Si	-0.69201500	-1.05620200	0.48377100
N	2.81397300	-0.23179300	-0.50053300
N	1.19915700	2.14644100	-0.79472100
C	3.72480700	0.54731400	-1.06394400
C	3.42696500	1.79905200	-1.64613100
H	4.24199200	2.27022900	-2.19460500
C	2.28815300	2.57806600	-1.44965200
C	5.16678500	0.12721400	-1.12101200
H	5.39082400	-0.23183100	-2.13769000
H	5.39489400	-0.68093700	-0.41645600
H	5.82377400	0.98441900	-0.92294100
C	2.28186500	3.96263800	-2.03421400
H	3.29257500	4.28816900	-2.30771200
H	1.83079600	4.69976000	-1.35783000
H	1.66374800	3.94620100	-2.94707800
C	3.17898800	-1.44826800	0.15903100
C	3.42528400	-2.61733300	-0.59087900
C	3.72489600	-3.79671600	0.10054800
H	3.91495000	-4.71118900	-0.46663800
C	3.77828000	-3.82566900	1.48667900
H	4.00516800	-4.75828000	2.00887800
C	3.54065500	-2.66173400	2.21058800
H	3.58004900	-2.69199700	3.29987200
C	3.24218200	-1.45756300	1.57082100
C	3.37857700	-2.65272500	-2.10659000
H	3.11968000	-1.64611300	-2.46607400
C	4.72609800	-3.05416100	-2.70956100
H	4.98403700	-4.09366700	-2.45158300
H	5.54841300	-2.41647500	-2.35556100
H	4.68967400	-2.98713400	-3.80817000
C	2.27924000	-3.59235100	-2.59587800
H	1.31852200	-3.34440300	-2.13130500
H	2.51161000	-4.64204200	-2.35429700
H	2.15802500	-3.51331500	-3.68704200
C	3.04143300	-0.18723000	2.37292200
H	2.24946000	0.38172300	1.86129200
C	4.31747700	0.65833100	2.37492400
H	5.15863300	0.08912300	2.80190400
H	4.18215500	1.56440100	2.98532200
H	4.60700800	0.98162600	1.36578200
C	2.55787900	-0.43733500	3.79560600
H	3.33224900	-0.91313700	4.41881400
H	1.65642300	-1.06436800	3.77782900
H	2.30327300	0.51894100	4.27596400
C	0.27970000	3.10083100	-0.26981300
C	-0.89577500	3.42715100	-0.97304900
C	-1.78895100	4.33737400	-0.39743700
H	-2.70989500	4.58997800	-0.92925200

C	-1.51429200	4.94267000	0.82191500
H	-2.21900900	5.65845800	1.25167500
C	-0.33426200	4.63667100	1.49122800
H	-0.12245400	5.11618400	2.44892000
C	0.57325800	3.70902100	0.97434800
C	-1.19872200	2.84527600	-2.33765700
H	-0.33248800	2.24494000	-2.65112200
C	-1.41022300	3.93628200	-3.38693700
H	-2.31688600	4.52899200	-3.18558100
H	-1.52326100	3.48759900	-4.38596400
H	-0.56054300	4.63479900	-3.42145900
C	-2.39517900	1.90195400	-2.27677900
H	-2.20465500	1.06487600	-1.59033500
H	-2.60568300	1.47404600	-3.26812100
H	-3.29816400	2.43298100	-1.93262300
C	1.84251100	3.37892900	1.73949900
H	2.18539300	2.39962600	1.37427600
C	2.95623700	4.38985600	1.45705600
H	3.86125700	4.13151500	2.02881900
H	2.64695600	5.40568700	1.75227800
H	3.23377500	4.41654100	0.39530700
C	1.61344000	3.26129500	3.24410900
H	2.53585600	2.92562300	3.74064800
H	0.82046800	2.53905400	3.47925900
H	1.34234800	4.22803000	3.69634900
N	-2.28290400	-0.35382200	1.10236200
N	-2.24752000	-1.49266400	-0.72376700
C	-3.04265900	-0.89343000	0.13341600
C	-4.52678800	-0.86055200	0.09689200
C	-5.25322000	-1.68268300	0.96527900
H	-4.72130100	-2.34520800	1.65084100
C	-6.64472000	-1.67428100	0.93655500
H	-7.20445400	-2.32485700	1.61228800
C	-7.32098300	-0.83977700	0.04693300
H	-8.41320400	-0.83062500	0.02759500
C	-6.59965200	-0.01956800	-0.81909900
H	-7.12431700	0.63682000	-1.51699900
C	-5.20694100	-0.03218100	-0.79959900
H	-4.64192800	0.61645000	-1.46948500
C	-2.55705700	-2.39950800	-1.83128100
C	-1.27345000	-3.17500900	-2.11363900
H	-0.96222200	-3.75822400	-1.23527300
H	-0.47001200	-2.47628300	-2.38103200
H	-1.42226800	-3.86336400	-2.95869900
C	-3.66678800	-3.39439000	-1.47871300
H	-3.44714200	-3.89788800	-0.52446000
H	-3.72688800	-4.16387100	-2.26306800
H	-4.65325300	-2.91853000	-1.40402200
C	-2.93075800	-1.60469100	-3.08509000
H	-3.85009000	-1.02199800	-2.93101300
H	-3.10329300	-2.29134700	-3.92813100
H	-2.11215800	-0.92316900	-3.35594400
C	-2.62875600	0.47814900	2.26147600
C	-2.98841600	-0.41820200	3.44952700
H	-2.15854900	-1.11069500	3.65108200
H	-3.90065400	-0.99920400	3.25247700
H	-3.16430300	0.19052700	4.34974500
C	-3.76194100	1.45856900	1.95100000
H	-3.85812000	2.16801100	2.78638900
H	-4.73292600	0.96400700	1.82082700
H	-3.53332400	2.03764900	1.04312300
C	-1.38193000	1.28720100	2.61077900
H	-0.54404500	0.61949700	2.85391300
H	-1.58708700	1.93273300	3.47733900
H	-1.09723200	1.93368300	1.76981000
O	0.15880400	-1.31936300	1.99922500
C	-0.03446900	-2.56950900	1.27558900
H	0.92312200	-3.01591000	0.95654100
C	-0.90105700	-3.57512100	1.99720500

H	-1.05114900	-4.44344300	1.33107100
H	-1.90434100	-3.15101800	2.18182000
C	-0.28612100	-4.02288200	3.31617300
H	-0.13101900	-3.15544100	3.97583900
H	-0.92347000	-4.74880700	3.84522900
H	0.69857500	-4.48888200	3.15042900

Cartesian coordinates of the optimized geometry of **S2** at PBE0-D3BJ/def2-SVP level of theory:

Ga	1.39532100	-0.24659300	-0.44764700
Cl	0.48995600	-1.46110500	-2.09741100
Si	-2.11341600	-1.11484100	1.87112900
O	-0.84206400	-0.28914800	1.07789400
N	1.49182800	1.54685200	-1.25316500
N	3.31436600	-0.62283900	-0.66056100
N	-3.17612800	-1.74117700	0.39235400
N	-3.51098500	0.09837500	1.43637100
C	2.33374800	1.71701100	-2.27231600
C	3.38313400	0.83070000	-2.57687800
H	3.94136800	1.05431000	-3.48484000
C	3.89972900	-0.20504000	-1.78162400
C	2.16619200	2.91051500	-3.16937300
H	1.30168500	2.74938500	-3.83183000
H	3.05578500	3.06705500	-3.79055000
H	1.95583800	3.82213300	-2.59421200
C	5.15679700	-0.88000000	-2.25073800
H	5.80203200	-1.17706300	-1.41408700
H	5.72052900	-0.24505000	-2.94477800
H	4.87469100	-1.80426500	-2.78160000
C	0.55321800	2.57102700	-0.91339500
C	-0.68476700	2.65412800	-1.58150300
C	-1.56831600	3.67636500	-1.21890200
H	-2.53067300	3.75300200	-1.73071300
C	-1.24975600	4.58780900	-0.22243700
H	-1.95539000	5.37690100	0.04774000
C	-0.03263000	4.48239300	0.44190100
H	0.20606700	5.19342600	1.23412700
C	0.88570200	3.48079000	0.11692200
C	-1.10670800	1.65871500	-2.64233400
H	-0.24848300	1.00934000	-2.86618400
C	-1.54323800	2.33407800	-3.94175700
H	-2.46510500	2.92213300	-3.80711000
H	-1.74894800	1.57570800	-4.71286000
H	-0.77420200	3.01705100	-4.33439000
C	-2.21106400	0.75388100	-2.09951000
H	-1.90241800	0.25108300	-1.17238900
H	-2.46948400	-0.02010900	-2.83816300
H	-3.11842300	1.34186100	-1.88470800
C	2.21298800	3.38980400	0.84751900
H	2.46264100	2.31964900	0.90636500
C	3.34546200	4.07155300	0.07682800
H	3.11180800	5.13258300	-0.10593600
H	3.53582500	3.59040300	-0.89176800
H	4.28259000	4.02703100	0.65398400
C	2.15028500	3.92567900	2.27452200
H	1.30576200	3.49916200	2.83557800
H	2.05338400	5.02259600	2.29663700
H	3.07491900	3.67336900	2.81361500
C	4.00306400	-1.45649400	0.27156900
C	3.89060100	-2.85946100	0.20466900
C	4.55287200	-3.62615300	1.16955100
H	4.47083100	-4.71518700	1.13032400
C	5.30907900	-3.03194300	2.17044500
H	5.81897500	-3.64888700	2.91411300
C	5.41135800	-1.64585700	2.22517000
H	6.00498200	-1.18366600	3.01625200
C	4.76350800	-0.83538100	1.29026000

C	3.07620300	-3.55548400	-0.86601200
H	2.70566200	-2.79004300	-1.56243300
C	3.91580300	-4.54851700	-1.66902900
H	4.26029000	-5.39042700	-1.04750900
H	4.80857500	-4.07075500	-2.10067600
H	3.32195300	-4.96994200	-2.49480900
C	1.84717500	-4.23449000	-0.26283800
H	2.13370600	-5.04452000	0.42725700
H	1.21528600	-4.66375800	-1.05485600
H	1.22979200	-3.51716600	0.29594800
C	4.90785000	0.67396500	1.35492300
H	4.02562100	1.09710700	0.85082600
C	6.13936300	1.15508300	0.58413000
H	7.05788200	0.71065000	1.00008500
H	6.23229400	2.25052300	0.64903200
H	6.08904300	0.89072700	-0.48030500
C	4.93737500	1.21368900	2.78192800
H	4.07940300	0.86041700	3.37275800
H	4.91333400	2.31373300	2.76950800
H	5.85652500	0.92131700	3.31300700
C	-4.06603300	-0.77073700	0.59488600
C	-5.46637200	-0.72666300	0.09579000
C	-6.51511500	-1.08547200	0.94965800
H	-6.29090600	-1.41657300	1.96611200
C	-7.83291800	-1.03500500	0.50162300
H	-8.64542300	-1.32253400	1.17294400
C	-8.11340500	-0.61928100	-0.79946300
H	-9.14748400	-0.57597600	-1.14926900
C	-7.07107900	-0.26086000	-1.65327300
H	-7.28576800	0.06617700	-2.67323000
C	-5.75165000	-0.31751900	-1.21054300
H	-4.93373200	-0.03124000	-1.87357800
C	-3.29880000	-3.05413700	-0.23913900
C	-4.38710400	-3.89756600	0.43131100
H	-4.20091200	-3.96607300	1.51419500
H	-4.39141900	-4.91736100	0.01632300
H	-5.38653000	-3.46654400	0.27477800
C	-3.57103000	-2.91407500	-1.73851300
H	-4.55597100	-2.46893600	-1.93457500
H	-3.55089200	-3.90604300	-2.21501500
H	-2.79390500	-2.29192900	-2.20499500
C	-1.93822100	-3.72869200	-0.05643000
H	-1.69909400	-3.83987900	1.01337300
H	-1.14970000	-3.12922900	-0.53475500
H	-1.94095300	-4.72956200	-0.51285500
C	-3.93700700	1.38970900	1.96401300
C	-4.85492200	1.19466000	3.17541100
H	-4.36732800	0.54850000	3.92081000
H	-5.80621700	0.72902700	2.87955400
H	-5.08374300	2.16221200	3.64862600
C	-2.65835800	2.11013800	2.40022300
H	-2.89869600	3.10398000	2.80607800
H	-1.96938800	2.22594400	1.55196100
H	-2.13795100	1.53872700	3.18446900
C	-4.63863600	2.23363200	0.89905200
H	-4.82987200	3.24132800	1.29773800
H	-5.60271900	1.80359600	0.59614200
H	-3.99985700	2.33424900	0.00992100
C	0.53448600	-0.51958900	1.31220900
H	0.71213700	-1.57587100	1.60579300
C	1.08274600	0.37892700	2.41277400
H	0.77841400	1.41199400	2.18243500
H	2.18461100	0.35786400	2.36524800
C	0.64251900	0.01497400	3.82257500
H	-0.45464800	0.01080100	3.91777500
H	0.98993500	-0.99365900	4.09702500
H	1.04385500	0.72598000	4.56249700

Cartesian coordinates of the optimized geometry of Int-**1** at PBE0-D3BJ/def2-SVP level of theory:

Ga	-1.38039800	-1.08121300	-0.71388300
Cl	-0.59511300	-1.16875000	-2.80544800
Si	2.12752700	0.88829700	0.76951700
O	0.81389300	-0.11081200	0.74042400
N	-1.41458700	-2.99962800	-0.23699600
N	-3.32593200	-0.95692600	-1.01439100
N	3.06089900	1.07157700	-0.78286500
N	3.51081500	-0.39328000	0.72941600
C	-2.28386900	-3.77934800	-0.88469800
C	-3.39553100	-3.29540300	-1.59049900
H	-3.98280400	-4.04524100	-2.11821300
C	-3.93518700	-1.99896200	-1.57640800
C	-2.07435400	-5.26785800	-0.89300500
H	-1.26887500	-5.51527400	-1.60204000
H	-2.98410700	-5.79187400	-1.20837200
H	-1.75632100	-5.64434600	0.08767800
C	-5.25861800	-1.80454700	-2.26007700
H	-5.07251500	-1.42864800	-3.27910500
H	-5.88151400	-1.05717500	-1.75285800
H	-5.80803000	-2.75018500	-2.34015600
C	-0.41797000	-3.60538000	0.59008400
C	0.79362600	-4.05915000	0.02871700
C	1.72374700	-4.67755300	0.86961900
H	2.65974900	-5.04881800	0.44553400
C	1.47885000	-4.83609900	2.22769700
H	2.22141000	-5.31874300	2.86748500
C	0.28378300	-4.37645500	2.76880200
H	0.09340200	-4.50479700	3.83608400
C	-0.68432100	-3.76350200	1.96834100
C	1.11701400	-3.88383000	-1.44126500
H	0.20914700	-3.53145100	-1.94979000
C	1.55888900	-5.18425900	-2.10997700
H	2.52409200	-5.54145600	-1.71678100
H	1.68419800	-5.03020200	-3.19279700
H	0.82671000	-5.99325500	-1.96506900
C	2.17047900	-2.79357500	-1.62712300
H	1.88073000	-1.86503000	-1.11659100
H	2.30753300	-2.56656200	-2.69542200
H	3.13555200	-3.12273600	-1.21074000
C	-2.00898200	-3.33583200	2.57099900
H	-2.42143100	-2.56048000	1.90856500
C	-3.02128500	-4.48312400	2.58523600
H	-2.64238000	-5.33520400	3.17213800
H	-3.24867700	-4.84301300	1.57248500
H	-3.96903800	-4.15299800	3.03929800
C	-1.86107300	-2.73466000	3.96628300
H	-1.08542300	-1.95549400	3.99536100
H	-1.60170700	-3.49858600	4.71575200
H	-2.81050500	-2.27929600	4.28600300
C	-4.02611000	0.27667300	-0.83545500
C	-4.03062500	1.25089800	-1.85381900
C	-4.70996200	2.45100000	-1.61738300
H	-4.71659600	3.21986100	-2.39347500
C	-5.37139900	2.68171800	-0.41919900
H	-5.89190200	3.62778500	-0.25338200
C	-5.36583700	1.70658900	0.57302700
H	-5.88676400	1.89693300	1.51287400
C	-4.69919300	0.49312200	0.39002200
C	-3.33879000	1.03383600	-3.18493000
H	-2.96045700	0.00216600	-3.20347900
C	-4.29869100	1.20391400	-4.36259700
H	-4.64898400	2.24419800	-4.45434700
H	-5.18952200	0.56500400	-4.26471100
H	-3.79371600	0.94381300	-5.30575900
C	-2.12584700	1.94907200	-3.34114700
H	-2.42997400	3.00291400	-3.43291100

H	-1.55454000	1.67557300	-4.24084900
H	-1.44485800	1.87335800	-2.48265200
C	-4.73278300	-0.57538600	1.46926000
H	-3.80454500	-1.15706700	1.36515100
C	-5.89472900	-1.55131500	1.26968800
H	-6.85952600	-1.01948000	1.28374100
H	-5.90850300	-2.30032300	2.07733200
H	-5.82104200	-2.09551900	0.31909700
C	-4.76897800	-0.00307100	2.88351500
H	-3.98609600	0.75232700	3.04344200
H	-4.62115000	-0.80782000	3.61959000
H	-5.74010600	0.46371100	3.11111400
C	3.96748800	0.16785400	-0.38247300
C	5.29159300	-0.05578700	-1.00931300
C	6.28390500	0.91541000	-0.83910800
H	6.06952800	1.80883500	-0.24859200
C	7.53669800	0.74029500	-1.42122700
H	8.30957000	1.49938600	-1.28207500
C	7.79921800	-0.39656800	-2.18450500
H	8.77963000	-0.52947000	-2.64758500
C	6.80835800	-1.36213800	-2.35858300
H	7.00861500	-2.25066700	-2.96136100
C	5.55814400	-1.19787600	-1.76844200
H	4.77647200	-1.94450200	-1.91226800
C	2.92096100	1.85342400	-2.02547500
C	3.47311300	3.26010400	-1.79027100
H	2.94953400	3.75145600	-0.95735800
H	3.32617900	3.87696200	-2.68944600
H	4.55160700	3.22882000	-1.57126800
C	3.62953300	1.18447900	-3.20281700
H	4.72377300	1.21145100	-3.12211000
H	3.35062700	1.72067700	-4.12123200
H	3.30578200	0.13851700	-3.31468000
C	1.42661000	1.91715300	-2.33560100
H	0.87354600	2.45045000	-1.54924400
H	1.01469900	0.90780700	-2.47660400
H	1.26897500	2.48161900	-3.26667400
C	4.18787800	-1.14711300	1.78922700
C	5.30163800	-0.30679100	2.42092500
H	4.90923100	0.65814700	2.77321900
H	6.11134900	-0.11341900	1.70241500
H	5.73534300	-0.83607700	3.28286400
C	3.10488800	-1.44837000	2.82712100
H	3.52027700	-2.04713200	3.65041300
H	2.27455400	-2.00455800	2.36968500
H	2.70305800	-0.51745800	3.25582200
C	4.76004300	-2.46545400	1.27034900
H	5.16255300	-3.04703100	2.11317300
H	5.57635300	-2.30470100	0.55381000
H	3.97168000	-3.06015900	0.79132800
C	-0.55708700	0.23771800	0.53824800
H	-0.61170000	1.24710700	0.09506900
C	-1.35450600	0.23078800	1.83604300
H	-1.57834500	-0.80247900	2.13673300
H	-2.32302000	0.69431100	1.59239100
C	-0.72947800	0.94421800	3.01946900
H	0.17801800	0.41441100	3.34544200
H	-0.45771900	1.98097700	2.77914900
H	-1.42523800	0.95352900	3.87327700
C	2.55019000	2.25507800	1.91103000
O	1.35173000	2.53615500	1.09946000
H	3.38069900	2.87521400	1.52509200
C	2.45376100	2.38568000	3.41081600
H	1.61296200	1.79174900	3.79322300
H	3.36632400	1.90436500	3.80814800
C	2.36398200	3.81376700	3.93104500
H	2.38256000	3.82595200	5.03217300
H	3.21127000	4.41880100	3.57107900
H	1.43877400	4.29854000	3.58825000

H	2.01473000	4.61895200	0.89890100
C	-0.80400300	5.08615100	-0.18466200
C	-1.20811500	4.87281200	1.31614600
O	0.01114000	4.46518000	1.90368700
O	0.18146400	4.08837200	-0.36580800
C	-1.70188200	6.12799200	2.02085400
H	-2.59390500	6.54313000	1.52527900
H	-1.97150700	5.88503500	3.05997800
H	-0.92047900	6.89830700	2.05007700
C	-2.25018100	3.76633300	1.46879800
H	-1.95824600	2.87227600	0.90203400
H	-2.32642600	3.49063900	2.53093600
H	-3.24577400	4.07978700	1.12211800
C	-0.16270000	6.45555600	-0.42163200
H	0.30298300	6.45223200	-1.41840000
H	-0.89868900	7.27250600	-0.38251700
H	0.62562900	6.65367000	0.31831200
C	-1.93167000	4.87626100	-1.17747000
H	-2.75022900	5.59144800	-1.00070700
H	-1.55745200	5.02989600	-2.20093800
H	-2.33654300	3.85982700	-1.11317200
B	0.92267300	4.02804100	0.86898500

Cartesian coordinates of the optimized geometry of Int-**2** at PBE0-D3BJ/def2-SVP level of theory:

Ga	1.31263500	0.17213800	-0.68937700
Cl	0.23473800	-0.02371200	-2.64100300
Si	-2.07898400	-1.54591600	0.86677200
O	-0.86351800	-0.41261600	1.07272300
N	1.85546500	2.06352800	-0.75617700
N	3.11669200	-0.52283600	-1.08862700
N	-3.23691300	-1.30517600	-0.55134200
N	-3.45110200	-0.28859200	1.33145100
C	2.72601300	2.40003300	-1.71030700
C	3.56014600	1.47619600	-2.35819800
H	4.17986000	1.87777900	-3.15982300
C	3.83840700	0.14897900	-1.98119500
C	2.81803500	3.83388400	-2.15007100
H	2.01110800	4.03436400	-2.87310200
H	3.77639600	4.03931600	-2.64188800
H	2.67735900	4.52990600	-1.31306000
C	5.01382300	-0.49925800	-2.65898200
H	4.80437600	-0.54911700	-3.73889600
H	5.20083000	-1.51489300	-2.29188700
H	5.92234600	0.10581000	-2.53442600
C	1.18186500	3.06032500	0.01423400
C	-0.02869000	3.61929900	-0.44294700
C	-0.68054100	4.55528200	0.36689100
H	-1.62337700	4.98928000	0.02499300
C	-0.15483700	4.93927900	1.59180000
H	-0.67951000	5.66981600	2.21201700
C	1.04521500	4.38769800	2.02747800
H	1.45352900	4.69529200	2.99119700
C	1.73352700	3.44536300	1.25910700
C	-0.65271000	3.23368300	-1.76792400
H	0.03972400	2.56040100	-2.29135400
C	-0.90244700	4.44669300	-2.66367200
H	-1.66511200	5.11873900	-2.23877200
H	-1.26343100	4.12276700	-3.65214600
H	0.01133900	5.04154100	-2.81284500
C	-1.94299500	2.44875000	-1.54538200
H	-1.77372000	1.56117500	-0.91866200
H	-2.35662900	2.10745000	-2.50648200
H	-2.69526900	3.08192500	-1.04750800
C	3.05599600	2.88073500	1.74685800
H	3.12598900	1.85447000	1.35528200
C	4.24627600	3.65773200	1.17921700

H	4.19438200	4.72017500	1.46637400
H	4.29010300	3.60357900	0.08337200
H	5.19255100	3.24837400	1.56693300
C	3.14892700	2.81316600	3.26823400
H	2.28139800	2.29930900	3.70784700
H	3.21377400	3.81582900	3.71896800
H	4.05448400	2.26909900	3.57038500
C	3.59851100	-1.70662300	-0.45417300
C	2.96091200	-2.94660700	-0.70478500
C	3.40463500	-4.07167800	-0.00642100
H	2.91666300	-5.03074400	-0.18030700
C	4.45359600	-3.99704300	0.90327400
H	4.78433600	-4.89291200	1.43389400
C	5.07110100	-2.77782500	1.13680600
H	5.89065400	-2.71919600	1.85721600
C	4.65473900	-1.61393400	0.48141200
C	1.85821500	-3.07553300	-1.73848200
H	1.19446200	-2.20350100	-1.63517800
C	2.42438600	-3.02097400	-3.15819800
H	3.11135000	-3.86393100	-3.33752100
H	2.96712600	-2.08645800	-3.34776700
H	1.60704000	-3.07714800	-3.89265900
C	0.97828200	-4.30541000	-1.55851600
H	1.53921600	-5.24218600	-1.70679500
H	0.17782200	-4.28921700	-2.31171400
H	0.49205300	-4.31259900	-0.57211700
C	5.35616000	-0.31036200	0.82327400
H	4.83881500	0.50611200	0.29686000
C	6.81797700	-0.31643900	0.36976400
H	7.39836000	-1.06714100	0.92938500
H	7.28388600	0.66526300	0.54963400
H	6.92055900	-0.55289300	-0.69733000
C	5.28517900	-0.01111600	2.32084900
H	4.24982000	-0.00367800	2.68820100
H	5.73402800	0.97039400	2.53777800
H	5.83903400	-0.76073800	2.90657800
C	-4.01608800	-0.46489700	0.15709300
C	-5.31793400	0.11285500	-0.26507700
C	-6.50321100	-0.54417100	0.08060900
H	-6.45643800	-1.48672200	0.63004900
C	-7.73424800	0.00935300	-0.26037000
H	-8.65571500	-0.50975800	0.01279100
C	-7.79045400	1.22460800	-0.94177100
H	-8.75701400	1.65967400	-1.20613600
C	-6.61032300	1.88283100	-1.28409900
H	-6.64790300	2.83473300	-1.81856700
C	-5.37653800	1.32974400	-0.94906600
H	-4.45233200	1.83875000	-1.22233600
C	-3.51892400	-1.98073200	-1.83008100
C	-4.68979600	-2.95510500	-1.67024900
H	-4.48600100	-3.66685600	-0.85586700
H	-4.83208900	-3.52980400	-2.59797800
H	-5.63158000	-2.43109800	-1.45537500
C	-3.81234000	-0.95651200	-2.92920000
H	-4.74868900	-0.40963700	-2.75718300
H	-3.90148400	-1.47425300	-3.89599100
H	-2.98201300	-0.23895100	-3.00179000
C	-2.27142600	-2.76452700	-2.22951300
H	-2.00517600	-3.50411500	-1.46504600
H	-1.41563700	-2.08652000	-2.35696500
H	-2.45624300	-3.27255100	-3.18763000
C	-3.67294400	0.66873500	2.41084000
C	-5.15127900	0.85014900	2.75967400
H	-5.62932100	-0.11980200	2.96334400
H	-5.71289400	1.35368800	1.96195300
H	-5.23291400	1.46771900	3.66683600
C	-2.94118000	0.10249700	3.62888200
H	-3.04256700	0.78280200	4.48689600
H	-1.87328000	-0.01715800	3.40119200

H	-3.34963100	-0.88044300	3.90742900
C	-3.04915900	2.01237600	2.01977500
H	-3.10062900	2.72344900	2.85868300
H	-3.58396800	2.45477600	1.16625700
H	-1.99655100	1.87312100	1.73628800
C	0.52823500	-0.65213100	0.93581700
H	0.70263300	-1.73872500	0.83217300
C	1.27763400	-0.14783300	2.16019300
H	1.01986000	0.91312200	2.31504500
H	2.35441400	-0.17378700	1.92235700
C	1.04949600	-0.92959100	3.44327400
H	-0.00380400	-0.89511800	3.75611100
H	1.32837100	-1.98716300	3.31478700
H	1.65164500	-0.51990600	4.26977400
C	-2.19864900	-3.17781300	1.67632500
O	-1.22762700	-3.01160800	0.57754700
H	-3.01675200	-3.86071700	1.37555500
C	-1.58205200	-3.62555500	2.97877600
H	-0.89715300	-2.85446800	3.36252300
H	-2.38971000	-3.71797200	3.72665700
C	-0.83034700	-4.94254600	2.84272800
H	-0.37013400	-5.25179600	3.79425700
H	-1.50002100	-5.75282100	2.51085400
H	-0.03234300	-4.84472900	2.09099100

Cartesian coordinates of the optimized geometry of Int-**3** at PBE0-D3BJ/def2-SVP level of theory:

Ga	-2.05399900	0.19525400	-0.50873600
Cl	-1.03368400	-0.05409600	-2.49232400
Si	1.86520500	-0.21696800	0.61275800
O	0.22417200	-0.42417100	1.01666800
N	-3.37502200	-1.26578400	-0.59281000
N	-3.35863500	1.63276700	-0.84003100
N	2.70057800	-1.16616100	-0.77297400
N	2.17066600	-2.14013600	1.08012100
C	-4.46953800	-1.10868500	-1.33866500
C	-4.86525200	0.10718700	-1.92223800
H	-5.73195400	0.05299100	-2.57919500
C	-4.39698400	1.39822800	-1.63728700
C	-5.36969800	-2.28877000	-1.57887200
H	-6.33090800	-1.96606500	-1.99519200
H	-5.54500400	-2.84993100	-0.65055100
H	-4.90176000	-2.98981500	-2.28438500
C	-5.11202600	2.55259000	-2.27935100
H	-5.16184900	3.42631300	-1.61689200
H	-6.12343600	2.27220000	-2.59732800
H	-4.54374100	2.85859300	-3.17278400
C	-3.11994900	-2.53904000	0.00333000
C	-2.60570700	-3.60319900	-0.76768300
C	-2.46743400	-4.85349800	-0.15645100
H	-2.07974200	-5.69102400	-0.74127600
C	-2.79654700	-5.04783300	1.17807800
H	-2.68169600	-6.03407700	1.63392800
C	-3.24474200	-3.97494600	1.94045500
H	-3.47362600	-4.12542500	2.99683800
C	-3.41101200	-2.70877100	1.37448800
C	-2.15032700	-3.42910600	-2.20439300
H	-2.45124500	-2.42712000	-2.54301200
C	-2.75228800	-4.46477400	-3.15389000
H	-2.38740800	-5.48040200	-2.93312200
H	-2.47023600	-4.23584700	-4.19298500
H	-3.85113900	-4.49567400	-3.10218000
C	-0.62358300	-3.47461800	-2.27633600
H	-0.16756200	-2.71436000	-1.62750100
H	-0.28042200	-3.28315300	-3.30458400
H	-0.25631900	-4.46754800	-1.96903700
C	-3.93202100	-1.55245000	2.20487500

H	-3.49250800	-0.63647500	1.77948300
C	-5.45122500	-1.41662400	2.08928200
H	-5.95187000	-2.33934200	2.42350200
H	-5.76698800	-1.21140000	1.05671100
H	-5.81502300	-0.58817800	2.71694100
C	-3.50057400	-1.62144200	3.66591900
H	-2.41644500	-1.78506300	3.75827800
H	-4.01520800	-2.42898300	4.21022700
H	-3.74716700	-0.67868400	4.17711700
C	-3.11054300	2.93789300	-0.31960300
C	-2.25192700	3.82962000	-0.98997600
C	-2.00137600	5.07457000	-0.40162500
H	-1.33231200	5.77604300	-0.90621600
C	-2.58397300	5.43246700	0.80619300
H	-2.37334200	6.40859800	1.24929900
C	-3.43176700	4.54070900	1.45661700
H	-3.87879600	4.82624700	2.41055800
C	-3.70620800	3.28240300	0.91747700
C	-1.60516600	3.48732000	-2.31730000
H	-1.95944600	2.49161200	-2.62057100
C	-2.00112200	4.48291800	-3.40857400
H	-1.60394800	5.48961800	-3.20180000
H	-3.09368100	4.57453300	-3.50381800
H	-1.59888100	4.16324100	-4.38240900
C	-0.08559700	3.39894700	-2.19042900
H	0.34606200	4.37077800	-1.90523700
H	0.36281100	3.09434300	-3.14841000
H	0.21788800	2.65746300	-1.43779100
C	-4.62836800	2.31418300	1.63953200
H	-4.30152900	1.30029900	1.35868400
C	-6.08051900	2.46211000	1.17906600
H	-6.44619000	3.48415100	1.36813000
H	-6.73093200	1.76185000	1.72630900
H	-6.20005100	2.25247800	0.10834000
C	-4.55213800	2.42358600	3.15998700
H	-3.51659600	2.37205800	3.52543000
H	-5.11761400	1.60143900	3.62478400
H	-4.99627500	3.36239200	3.52639600
C	2.77856600	-2.31041700	-0.07506600
C	3.50510700	-3.53958700	-0.49433000
C	4.81257700	-3.73148300	-0.03456500
H	5.27324100	-2.97444000	0.60395600
C	5.51469400	-4.88413500	-0.37579000
H	6.53522800	-5.02446600	-0.01234800
C	4.91402200	-5.85866000	-1.17194400
H	5.46306400	-6.76558200	-1.43561700
C	3.61126300	-5.67132500	-1.63114500
H	3.13597200	-6.42960000	-2.25759500
C	2.90971500	-4.51476900	-1.29843900
H	1.89594800	-4.36149000	-1.66931600
C	3.36854100	-0.78521400	-2.02949400
C	4.88524700	-0.69345400	-1.83186000
H	5.12214200	0.03887800	-1.04577200
H	5.36602100	-0.35741800	-2.76394100
H	5.31936700	-1.66784100	-1.56647700
C	3.02711300	-1.77260400	-3.14946400
H	3.47840300	-2.76009500	-2.99185200
H	3.40589600	-1.38424100	-4.10679900
H	1.93585900	-1.88447100	-3.23276200
C	2.84375400	0.58895100	-2.44890900
H	3.12356600	1.35796200	-1.71889700
H	1.74932700	0.57890900	-2.55168700
H	3.28731900	0.85502800	-3.42039500
C	1.63082300	-3.16011900	1.99121900
C	2.69983100	-4.14190000	2.48206300
H	3.56390100	-3.60430700	2.90200500
H	3.06010500	-4.80702700	1.68672500
H	2.27526600	-4.77302600	3.27761700
C	1.02167800	-2.45611600	3.20595700

H	0.50708700	-3.19895100	3.83336300
H	0.29629600	-1.70198700	2.87574100
H	1.78793000	-1.96819600	3.82075500
C	0.50367600	-3.91177100	1.28031500
H	0.02889500	-4.63200900	1.96252100
H	0.87617000	-4.46982700	0.41039200
H	-0.26460100	-3.20280800	0.94208600
C	-0.71416500	0.62485900	0.89507600
H	-0.21329600	1.50422100	0.43794000
C	-1.21854100	1.07652900	2.25586100
H	-1.81761400	0.28326600	2.73036900
H	-1.89810500	1.92571500	2.08463700
C	-0.10528100	1.50191700	3.20209900
H	0.52167500	0.64496600	3.48703000
H	0.55839700	2.24827000	2.73885600
H	-0.51501500	1.93468000	4.12815400
C	3.27336900	0.45310900	1.77005900
O	3.10806500	1.86686700	1.90521500
H	4.17427800	0.26875300	1.15102300
C	3.55123400	-0.11989600	3.14676600
H	2.67810000	0.04897900	3.79788700
H	3.66189200	-1.20908500	3.03960800
C	4.79268900	0.47113000	3.79843100
H	4.98555600	0.02019200	4.78425500
H	5.68566300	0.30724700	3.17229200
H	4.68095500	1.55623600	3.94050700
H	1.66151100	1.09225800	-0.11531400
C	4.24403400	4.62291000	0.00013300
C	5.28563800	3.50734600	-0.36730700
O	4.61795200	2.32085300	0.08288300
O	3.60440900	4.07327800	1.15750100
C	5.58974300	3.38057900	-1.84758800
H	6.02905400	4.31215600	-2.23530000
H	6.31376000	2.56848200	-2.00919000
H	4.68822500	3.14697600	-2.42709900
C	6.57811300	3.62360100	0.43284200
H	6.36752300	3.74100300	1.50616000
H	7.16071100	2.70065200	0.29932200
H	7.19179300	4.47337500	0.10101900
C	3.17140600	4.79151600	-1.06914700
H	2.35810200	5.40386300	-0.65417500
H	3.55937600	5.28712000	-1.97070900
H	2.74452600	3.81976900	-1.35611400
C	4.85133000	5.96517800	0.35956900
H	5.42930500	6.37295300	-0.48361900
H	4.04995300	6.67823500	0.60149700
H	5.50791500	5.88739600	1.23548100
B	3.74037200	2.71053300	1.07494600

Cartesian coordinates of the optimized geometry of Int-**4** at PBE0-D3BJ/def2-SVP level of theory:

Ga	-2.01529100	-0.35284100	-0.70177800
Cl	-1.05437400	-0.74990900	-2.67619400
Si	2.00158800	-0.68370800	0.69437600
O	0.38638200	-0.81925400	0.60859100
N	-3.25996300	-1.87585100	-0.57096300
N	-3.38414300	0.97109200	-1.15372400
N	2.84118800	-1.26703700	-0.82857600
N	2.64587000	-2.38331500	1.00549200
C	-4.26496300	-1.90932400	-1.44685200
C	-4.72937700	-0.77809400	-2.13607100
H	-5.53505400	-0.95767800	-2.84645700
C	-4.39803700	0.57235300	-1.92063400
C	-4.92938100	-3.22032200	-1.75858100
H	-5.96056700	-3.07534800	-2.10313800
H	-4.91909800	-3.90500000	-0.90127800
H	-4.36589000	-3.70749800	-2.57129900

C	-5.24209100	1.60751300	-2.60642200
H	-5.55358600	2.39650200	-1.90804700
H	-6.12873700	1.15594200	-3.06607800
H	-4.64914300	2.10374000	-3.39008000
C	-2.97549800	-2.98355400	0.28510500
C	-2.06521800	-3.98388100	-0.11063800
C	-1.74689400	-4.99503900	0.80323900
H	-1.03677600	-5.77251200	0.50979600
C	-2.32067100	-5.03393200	2.06644800
H	-2.05864200	-5.83120600	2.76604000
C	-3.23829300	-4.05532000	2.43535200
H	-3.69184300	-4.09414600	3.42720600
C	-3.58060800	-3.01878500	1.56422500
C	-1.43600200	-4.00884100	-1.48792400
H	-1.84285800	-3.16504000	-2.06175500
C	-1.77071700	-5.29748800	-2.23900800
H	-1.32213200	-6.17967200	-1.75449900
H	-1.38215200	-5.25204100	-3.26832400
H	-2.85624500	-5.46894200	-2.29217200
C	0.07350400	-3.79527500	-1.41098700
H	0.31700200	-2.87193600	-0.86825400
H	0.49761700	-3.71189100	-2.42351000
H	0.56329600	-4.64047900	-0.89984700
C	-4.59314200	-1.96836500	1.98088900
H	-4.33802100	-1.05071100	1.42966900
C	-6.01276100	-2.36137000	1.56519600
H	-6.30614100	-3.31485400	2.03297600
H	-6.10928700	-2.47510400	0.47777700
H	-6.73337400	-1.59141200	1.88218100
C	-4.55550900	-1.64650500	3.47154700
H	-3.54054900	-1.39813800	3.81381000
H	-4.92485800	-2.48526300	4.08207700
H	-5.20784200	-0.78695700	3.68668300
C	-3.23747500	2.35917700	-0.83351800
C	-2.54958300	3.22959400	-1.70198000
C	-2.47855700	4.58398600	-1.35890300
H	-1.94034800	5.26947700	-2.01713400
C	-3.05810300	5.06694500	-0.19421900
H	-2.98925300	6.12855500	0.05393300
C	-3.69910400	4.18836300	0.67248600
H	-4.12517800	4.56771200	1.60297000
C	-3.79116900	2.82634300	0.37919200
C	-1.82772700	2.74165200	-2.94007400
H	-2.11154300	1.69298500	-3.11142700
C	-2.19626000	3.53518200	-4.19238100
H	-1.85661600	4.58090100	-4.12932000
H	-3.28324200	3.55058800	-4.36960100
H	-1.71519100	3.09001500	-5.07672300
C	-0.31828900	2.76711800	-2.69756000
H	0.05365900	3.78959000	-2.53322100
H	0.21871400	2.34076400	-3.55787400
H	-0.03158500	2.18900900	-1.80946400
C	-4.47584900	1.87793500	1.34373300
H	-4.03651300	0.88488400	1.16316300
C	-5.97432600	1.75281800	1.06600700
H	-6.47027700	2.73317400	1.14785200
H	-6.44733500	1.07263500	1.79222500
H	-6.17345400	1.35353700	0.06161000
C	-4.22090600	2.23694300	2.80475800
H	-3.15050600	2.39639100	3.00070800
H	-4.57419200	1.42981100	3.46341600
H	-4.75549600	3.15257700	3.10177300
C	3.24208100	-2.36766600	-0.19114100
C	4.21877700	-3.36878000	-0.67880300
C	5.55094900	-3.28355400	-0.26442800
H	5.86677300	-2.46463800	0.38547000
C	6.47269600	-4.23666700	-0.69229900
H	7.51417400	-4.16399800	-0.37179800
C	6.06620800	-5.27630800	-1.52673100

H	6.78955100	-6.02435200	-1.85897600
C	4.73672700	-5.35907900	-1.94089400
H	4.41612400	-6.17102500	-2.59714900
C	3.81264800	-4.40655500	-1.52276800
H	2.77162300	-4.46594700	-1.84305500
C	3.32335800	-0.64228500	-2.07920000
C	4.74988300	-0.13183000	-1.87559000
H	4.76954000	0.57873400	-1.03639400
H	5.08571100	0.40002000	-2.77802200
H	5.45382900	-0.95717000	-1.69046100
C	3.24375400	-1.63243900	-3.24087300
H	3.97113400	-2.45019300	-3.15519600
H	3.45753200	-1.09503400	-4.17598000
H	2.22931300	-2.05167600	-3.31732000
C	2.38576200	0.52498800	-2.35868600
H	2.44935600	1.29711900	-1.57753600
H	1.35085400	0.17104900	-2.46017300
H	2.67402800	1.00848000	-3.30343200
C	2.66922400	-3.35071800	2.10903100
C	3.89441900	-3.09462700	2.99056000
H	3.91006800	-2.05362100	3.34642200
H	4.82474100	-3.28697000	2.43690100
H	3.87960100	-3.75715500	3.86900900
C	1.39019200	-3.11518700	2.91046600
H	1.33956500	-3.81692200	3.75505400
H	0.49851200	-3.25377500	2.28198200
H	1.36552900	-2.09696400	3.32318000
C	2.67081900	-4.79594800	1.61028200
H	2.52902900	-5.46721600	2.46976800
H	3.61327000	-5.07401300	1.12224300
H	1.84336300	-4.96545600	0.90646100
C	-0.77705500	-0.00910800	0.81595900
H	-0.46310700	1.04437200	0.79492000
C	-1.39940400	-0.34210100	2.16265100
H	-1.61008900	-1.42313300	2.19724100
H	-2.37941100	0.15891900	2.20992600
C	-0.58205800	0.06293200	3.37764300
H	0.42189200	-0.38023400	3.35609200
H	-0.44649600	1.15118700	3.42945700
H	-1.06616700	-0.26847800	4.30943100
C	3.15424200	0.60572200	1.47499900
O	3.31950200	1.65372200	0.60182000
H	4.08779200	-0.00806400	1.42988800
C	2.93981500	0.95453700	2.94501700
H	1.99344000	1.50414400	3.03548800
H	2.82745500	0.01443100	3.51787100
C	4.08156500	1.78681200	3.50749200
H	3.91405300	2.01476400	4.57149800
H	5.04522100	1.25658400	3.42285400
H	4.15043200	2.73486700	2.95421000
H	-0.70700800	3.11889200	0.38654000
C	3.45944800	4.35114800	-0.93829900
C	3.56900200	4.97448400	0.51265000
O	3.07627800	3.95727800	1.34713400
O	2.43674100	3.40049700	-0.79459400
C	4.99325000	5.32333200	0.93449500
H	5.44810800	6.06879500	0.26277300
H	4.97768900	5.74546900	1.95107000
H	5.63002600	4.42958400	0.95408100
C	2.70045400	6.22303200	0.68443700
H	1.66419800	6.03735100	0.36990700
H	2.68403700	6.49366300	1.75080600
H	3.08756300	7.08303500	0.11694500
C	4.74954800	3.64516600	-1.36714400
H	4.54901600	3.08836200	-2.29502000
H	5.57049100	4.35245500	-1.55930100
H	5.06579300	2.92296800	-0.60310100
C	3.05793400	5.34090700	-2.02388500
H	3.79319200	6.15445200	-2.12563800

H	2.99399500	4.81478900	-2.98878200
H	2.07269300	5.77928100	-1.81689500
B	2.46198300	2.88358000	0.56543300
C	0.14180000	3.47001200	1.00369400
H	0.51542500	4.37719600	0.50084800
O	1.13721700	2.48688100	1.03053000
C	-0.39892200	3.82443000	2.38215700
H	-1.21720000	4.55089200	2.23718600
H	-0.87891800	2.92692300	2.80930000
C	0.62394100	4.37163500	3.36180700
H	0.17601600	4.53983000	4.35432200
H	1.47613700	3.68665300	3.46989800
H	1.04208800	5.32445100	3.00721800

Cartesian coordinates of the optimized geometry of Int-**5** at PBE0-D3BJ/def2-SVP level of theory:

Ga	-1.82649500	-0.72898800	-0.69237300
Cl	-0.85060900	-1.10095300	-2.67346100
Si	2.15782300	-0.09271200	0.60077700
O	0.60807000	-0.62519200	0.71650100
N	-2.73170900	-2.46372000	-0.37770400
N	-3.47164900	0.21779300	-1.22008400
N	3.09606000	-0.57208800	-0.89038100
N	3.01093000	-1.77248900	0.89424800
C	-3.71149700	-2.79706500	-1.21617700
C	-4.39414700	-1.86826400	-2.01878100
H	-5.13746000	-2.28729600	-2.69533300
C	-4.36033400	-0.46631800	-1.94416600
C	-4.11276000	-4.23837000	-1.35935800
H	-5.15682800	-4.33008400	-1.68289600
H	-3.96223200	-4.80612900	-0.43280500
H	-3.47745300	-4.70099000	-2.13208500
C	-5.39305900	0.28643800	-2.73432700
H	-5.91284900	1.02973100	-2.11439500
H	-6.12836400	-0.39633400	-3.17535200
H	-4.89936500	0.84810100	-3.54212400
C	-2.21179200	-3.40999700	0.55890700
C	-1.15924600	-4.27239200	0.19446300
C	-0.63467100	-5.13331900	1.16481400
H	0.18706600	-5.80155600	0.89486600
C	-1.13889400	-5.15632900	2.45750300
H	-0.71332000	-5.83242600	3.20277200
C	-2.19446700	-4.31692900	2.79840500
H	-2.59316100	-4.34467200	3.81377800
C	-2.74985700	-3.43631800	1.86731000
C	-0.58142800	-4.29796000	-1.20465800
H	-1.16062600	-3.60155400	-1.82648900
C	-0.66992000	-5.68694700	-1.83640300
H	-0.03681100	-6.41611100	-1.30579700
H	-0.32790300	-5.65301500	-2.88254500
H	-1.69830100	-6.07794100	-1.82670000
C	0.85643000	-3.78972500	-1.20324700
H	0.92851300	-2.79819800	-0.73565600
H	1.23527500	-3.71244500	-2.23363200
H	1.51053700	-4.47881400	-0.64493100
C	-3.92653700	-2.55785400	2.24883600
H	-3.85322900	-1.65067800	1.63111700
C	-5.25905800	-3.22869600	1.90713800
H	-5.36009300	-4.18903500	2.43772100
H	-5.36042900	-3.42379900	0.83163200
H	-6.10126300	-2.58543200	2.20640700
C	-3.91556200	-2.12952900	3.71259300
H	-2.95602000	-1.67666800	4.00101100
H	-4.11115400	-2.97599200	4.38941800
H	-4.70879600	-1.38912000	3.89358400
C	-3.68434700	1.62092800	-1.02554500
C	-3.21291500	2.55821000	-1.96649000

C	-3.53599900	3.90611100	-1.77891800
H	-3.17551200	4.64272200	-2.50028500
C	-4.28694200	4.32561500	-0.68919100
H	-4.52772600	5.38360600	-0.56207900
C	-4.70606600	3.39595700	0.25560800
H	-5.27211100	3.73266200	1.12618800
C	-4.41242800	2.03783000	0.11105400
C	-2.32084800	2.16315900	-3.12335800
H	-2.32349400	1.06610400	-3.19882700
C	-2.79537900	2.72106400	-4.46353900
H	-2.72808700	3.81984100	-4.49915500
H	-3.83952400	2.44588000	-4.68035100
H	-2.16690100	2.33023000	-5.27835300
C	-0.88372000	2.59199400	-2.82655100
H	-0.79387600	3.68666200	-2.75082000
H	-0.20523100	2.24644400	-3.62011800
H	-0.51988100	2.18347800	-1.87420200
C	-4.89826500	1.04008100	1.14424200
H	-4.24578700	0.15874600	1.05586800
C	-6.32401700	0.56622200	0.85776900
H	-7.02418200	1.41686200	0.84296900
H	-6.66148600	-0.13610800	1.63632700
H	-6.39773600	0.04766800	-0.10831000
C	-4.77990100	1.56663500	2.57168700
H	-3.77355100	1.96041500	2.77617800
H	-4.98496700	0.76197300	3.29313000
H	-5.50223000	2.37269400	2.77364200
C	3.61418300	-1.65069200	-0.27100400
C	4.74920600	-2.47394300	-0.75989000
C	6.05111800	-2.03296200	-0.50262400
H	6.20759100	-1.08396600	0.01501200
C	7.14127500	-2.80031600	-0.90719200
H	8.15542000	-2.45086700	-0.70139300
C	6.93671000	-4.00663900	-1.57449100
H	7.79171500	-4.60708300	-1.89341400
C	5.63878200	-4.44495600	-1.83555900
H	5.47373300	-5.38897300	-2.35950200
C	4.54638200	-3.68450300	-1.42824300
H	3.53058700	-4.02881800	-1.62424100
C	3.27747900	-0.10474300	-2.27776300
C	4.68136600	0.47565400	-2.46083600
H	4.88585000	1.24402900	-1.70316200
H	4.76615400	0.94550600	-3.45209800
H	5.45365200	-0.30366800	-2.39245700
C	3.04024500	-1.24489200	-3.27077300
H	3.80458200	-2.03000800	-3.20176300
H	3.07255900	-0.84083800	-4.29343200
H	2.04427300	-1.68284700	-3.11357400
C	2.21581600	0.95991400	-2.52671400
H	2.28030600	1.80243900	-1.82934000
H	1.21551300	0.51358300	-2.44446300
H	2.32274600	1.35109400	-3.54914900
C	3.29972300	-2.58227800	2.07816100
C	4.46565900	-1.95892500	2.85336100
H	4.24780300	-0.91257500	3.11167500
H	5.39090100	-1.98655300	2.25870800
H	4.64581000	-2.51334800	3.78693400
C	2.02959400	-2.55075300	2.92953500
H	2.17371300	-3.13903600	3.84736400
H	1.17437000	-2.96001900	2.37320800
H	1.77710600	-1.52118100	3.22032800
C	3.62306200	-4.03621800	1.73345000
H	3.67951700	-4.61986700	2.66414300
H	4.58410500	-4.14150400	1.21418400
H	2.83235700	-4.47230800	1.10660600
C	-0.66580700	0.02222700	0.73352300
H	-0.52913300	1.10214600	0.55549900
C	-1.32561100	-0.16466300	2.09168000
H	-1.41717300	-1.24262700	2.30180200

H	-2.35467900	0.22210200	2.00942400
C	-0.63154400	0.52871400	3.25118800
H	0.34852700	0.07659500	3.45320300
H	-0.46801600	1.59207900	3.02668600
H	-1.22545800	0.44768000	4.17534600
C	2.97410000	1.22838500	1.58047000
O	2.03937900	1.73095100	0.55465200
H	4.00589100	1.48707600	1.28726500
C	2.68820100	1.59896500	3.02721700
H	1.77524200	2.20978000	3.04168700
H	2.45987500	0.69221500	3.61369600
C	3.82452100	2.35547400	3.69335300
H	3.59176500	2.57991700	4.74561600
H	4.76160900	1.77364900	3.67955400
H	3.99426500	3.30442700	3.16750000
H	-1.62093000	3.63444800	0.05783100
C	2.72846900	4.65765700	-0.81192000
C	2.86817400	5.09348800	0.69484400
O	2.32599800	3.99270000	1.39023900
O	1.60914800	3.79785600	-0.79025700
C	4.30351500	5.32181100	1.15197800
H	4.79083700	6.11476900	0.56350500
H	4.30749400	5.63461800	2.20717000
H	4.90294800	4.40581400	1.07140200
C	2.05390300	6.34420900	1.02537400
H	1.01885000	6.25429400	0.67025400
H	2.02393400	6.46256400	2.11825200
H	2.49473000	7.25248000	0.58789400
C	3.95590300	3.88555000	-1.29322200
H	3.75105200	3.46916700	-2.28907200
H	4.84653800	4.52659600	-1.36727500
H	4.17919200	3.05111300	-0.61348900
C	2.44841100	5.79761800	-1.77959100
H	3.25809500	6.54381200	-1.76987100
H	2.36373900	5.39696700	-2.80096700
H	1.50215000	6.29845600	-1.53788300
B	1.46586000	3.22654700	0.51831300
C	-0.86846500	3.98557100	0.78532400
H	-0.44787100	4.91601800	0.36261000
O	0.11772300	3.00981100	0.93919900
C	-1.56237100	4.27132700	2.10778300
H	-2.41623400	4.94411000	1.91620100
H	-2.00031900	3.32410100	2.46740800
C	-0.64679900	4.85328100	3.17143900
H	-1.14435900	4.90929600	4.15214200
H	0.26315200	4.24296000	3.27425300
H	-0.32006400	5.86978100	2.90269300

Cartesian coordinates of the optimized geometry of Int-**6** at PBE0-D3BJ/def2-SVP level of theory:

Ga	-1.28054100	-0.07070300	-0.84786400
Cl	-1.31708600	-0.18622200	-3.10277000
Si	1.02004000	0.20645500	-0.15092400
N	-2.46291300	1.48009200	-0.51832100
N	-2.66988900	-1.39876400	-0.39182100
C	-3.73028700	1.36952300	-0.89723400
C	-4.33341500	0.13780400	-1.22986500
H	-5.33247500	0.19871100	-1.65878600
C	-3.87725300	-1.14595200	-0.92147200
C	-4.58773200	2.59829000	-0.99229100
H	-4.30676900	3.17619000	-1.88578400
H	-4.43173700	3.26062200	-0.13011000
H	-5.64890400	2.33321200	-1.06630700
C	-4.78752100	-2.29855600	-1.24092600
H	-5.81975400	-1.96364000	-1.39810200
H	-4.76854900	-3.07514200	-0.46595500
H	-4.42992600	-2.76563000	-2.17351700

C	-1.96093400	2.74534100	-0.06915500
C	-1.56850000	3.72510100	-1.00159700
C	-1.13271200	4.96172700	-0.51959100
H	-0.81812900	5.72902800	-1.23088900
C	-1.06677300	5.22153700	0.84081800
H	-0.71312700	6.19143000	1.19803700
C	-1.42121000	4.23244300	1.74983900
H	-1.33949900	4.43427500	2.81876800
C	-1.86937300	2.98263700	1.31946400
C	-1.51684100	3.44945400	-2.49005400
H	-1.99536600	2.47726900	-2.67891500
C	-2.23771800	4.50255200	-3.32861100
H	-1.75605500	5.48944200	-3.24431200
H	-3.28982600	4.62533400	-3.02861500
H	-2.22074800	4.21978100	-4.39257200
C	-0.05638800	3.31434900	-2.91925700
H	0.48430400	2.64324400	-2.24044600
H	0.45673800	4.28879700	-2.88089000
H	0.02052000	2.91742300	-3.94294200
C	-2.28391700	1.92414300	2.32348800
H	-2.00601800	0.95015900	1.88622400
C	-3.79998200	1.92215100	2.53636000
H	-4.14347400	2.91867000	2.85639700
H	-4.08279100	1.20301100	3.32031600
H	-4.34923400	1.64811200	1.62530500
C	-1.56396100	2.04130800	3.66243300
H	-1.90796100	2.91636600	4.23630400
H	-0.47612100	2.12498400	3.52225200
H	-1.76609000	1.15134800	4.27728900
C	-2.43255000	-2.65559900	0.24246000
C	-1.84250700	-3.71881300	-0.46876200
C	-1.60846300	-4.92563800	0.19964700
H	-1.14610800	-5.75393800	-0.34296900
C	-1.96607100	-5.09306500	1.53009200
H	-1.77823700	-6.04312600	2.03573500
C	-2.56793900	-4.04391000	2.21683600
H	-2.85047300	-4.18285000	3.26166400
C	-2.81163400	-2.81474600	1.59883100
C	-1.47894700	-3.60126200	-1.93333700
H	-1.85509300	-2.63670100	-2.30264800
C	-2.12530800	-4.70105900	-2.77497200
H	-1.72348400	-5.69717700	-2.52997200
H	-1.93449200	-4.52248300	-3.84440800
H	-3.21496700	-4.73884500	-2.62538300
C	0.03459700	-3.58490800	-2.12060600
H	0.49433700	-2.74057500	-1.58721100
H	0.29010900	-3.48275800	-3.18552100
H	0.48967000	-4.51836400	-1.74939900
C	-3.49400500	-1.69779700	2.37079900
H	-3.12486900	-0.75180300	1.94624900
C	-5.01462200	-1.71592900	2.18753300
H	-5.48074400	-0.93727000	2.81090600
H	-5.43165100	-2.68902500	2.49305300
H	-5.31584800	-1.52421700	1.15083500
C	-3.17194300	-1.71008600	3.86309800
H	-3.54740200	-0.79039300	4.33603800
H	-2.09296700	-1.77337900	4.05382200
H	-3.65918000	-2.55298600	4.37802800
N	1.65174300	-1.23728000	0.82492600
N	2.22406200	-0.77286300	-1.19025200
C	2.52932700	-1.53344200	-0.13266300
C	3.68658600	-2.45053300	-0.00967000
C	4.85028500	-1.99336300	0.61964100
H	4.88100000	-0.97053700	1.00201000
C	5.95276100	-2.83540500	0.73424500
H	6.86142000	-2.47407800	1.22100400
C	5.89801200	-4.13412100	0.22778000
H	6.76413800	-4.79351200	0.32059400
C	4.73873500	-4.59054500	-0.39742300

H	4.69251200	-5.60766000	-0.79291200
C	3.63368600	-3.75055800	-0.51951300
H	2.72008600	-4.10408400	-1.00106000
C	2.97946600	-0.51253500	-2.42354100
C	2.29935800	0.67967300	-3.08831100
H	2.36738500	1.57126800	-2.44969100
H	1.23904000	0.46337600	-3.28485600
H	2.78854800	0.91154500	-4.04546000
C	4.43907100	-0.15998100	-2.12096100
H	4.49112000	0.58251200	-1.31089900
H	4.90978700	0.26893900	-3.01807700
H	5.02478700	-1.03989900	-1.82406200
C	2.90034300	-1.71605400	-3.36259200
H	3.33805500	-2.61508200	-2.90630600
H	3.45682500	-1.50205800	-4.28751500
H	1.85495900	-1.92247400	-3.62842400
C	1.50339500	-1.74938900	2.19063600
C	2.67840200	-1.30956600	3.06972100
H	2.87227600	-0.23556400	2.92833700
H	3.59230300	-1.86810200	2.82633200
H	2.44374600	-1.50112900	4.12778100
C	1.35672500	-3.27090900	2.17360100
H	1.14606600	-3.63336400	3.19094100
H	2.27417900	-3.76542800	1.82565900
H	0.52075900	-3.57273300	1.52509200
C	0.22177500	-1.12766500	2.73335400
H	0.29857400	-0.03143700	2.75112200
H	0.04397900	-1.47791600	3.75958900
H	-0.63961000	-1.41921400	2.11572100
H	0.81094100	1.68492200	1.81526900
C	3.61655300	2.91873300	1.51751400
C	2.88801500	3.77919400	0.42313200
B	1.67301400	1.87226400	0.92379100
O	3.05035400	1.63418000	1.31897100
O	1.60644800	3.18908900	0.36085100
C	3.26821000	3.38405700	2.93220600
H	3.62369400	2.62879500	3.64914200
H	3.73424800	4.34828700	3.18386200
H	2.17850300	3.47794500	3.05018400
C	5.12438500	2.82546400	1.35979000
H	5.54368900	2.20876300	2.16953900
H	5.39790600	2.35916700	0.40375300
H	5.59414700	3.81995600	1.41212700
C	3.56034700	3.65970500	-0.94338200
H	3.75206800	2.60789800	-1.19252400
H	2.88316500	4.07255900	-1.70524400
H	4.51363200	4.20746800	-0.99193000
C	2.73035900	5.25039700	0.77896600
H	3.70741800	5.73532900	0.93266600
H	2.21370900	5.77035700	-0.04175000
H	2.12218100	5.37437500	1.68399500

Cartesian coordinates of the optimized geometry of Int-**7** at PBE0-D3BJ/def2-SVP level of theory:

Ga	-1.81454900	-0.69431200	-0.52666600
Cl	-0.95990400	-0.74543700	-2.59676100
Si	2.07308200	0.75907700	0.52483600
O	0.67902500	-0.17501200	0.58005100
N	-2.41488400	-2.55055400	-0.27478800
N	-3.62375600	0.02995600	-0.88531300
N	3.19364600	0.52809800	-0.93268800
N	3.13911200	-1.03864400	0.55170600
C	-3.47301800	-2.99123000	-0.95248500
C	-4.41121200	-2.14734500	-1.56651800
H	-5.21375100	-2.64942000	-2.10530100
C	-4.53435400	-0.75174000	-1.45959700
C	-3.69422500	-4.47071200	-1.09828300

H	-3.08878600	-4.83751800	-1.94226100
H	-4.74634600	-4.69458500	-1.31197000
H	-3.37271700	-5.02577600	-0.20784800
C	-5.77362800	-0.14295100	-2.05472900
H	-6.07714000	0.77340300	-1.53318300
H	-6.60342800	-0.86037700	-2.05130800
H	-5.56874000	0.13073600	-3.10175100
C	-1.60695600	-3.45092000	0.48480300
C	-0.55171700	-4.15705500	-0.12688300
C	0.22185400	-5.00819900	0.66944400
H	1.04229100	-5.56591300	0.21154100
C	-0.02834400	-5.15230600	2.02722000
H	0.59498300	-5.81452100	2.63256300
C	-1.07123500	-4.44525800	2.61745200
H	-1.25895800	-4.56132900	3.68613900
C	-1.87892500	-3.58883100	1.86576200
C	-0.23064900	-4.01405800	-1.60159600
H	-0.99546800	-3.36530800	-2.05043100
C	-0.25475800	-5.35698900	-2.33106300
H	0.54619400	-6.02519000	-1.97623600
H	-0.10277700	-5.20732200	-3.41134000
H	-1.20858500	-5.88771400	-2.19150500
C	1.11143900	-3.31176600	-1.80978000
H	1.18571600	-2.39345400	-1.20943800
H	1.23664100	-3.03530000	-2.86775300
H	1.94288600	-3.97841200	-1.53000300
C	-3.04150800	-2.85281900	2.50848200
H	-3.14283700	-1.89757400	1.97072200
C	-4.36161400	-3.60661200	2.32916200
H	-4.30134500	-4.61178200	2.77628700
H	-4.63549200	-3.72001900	1.27227800
H	-5.18238700	-3.06385200	2.82443700
C	-2.82150300	-2.53526700	3.98406500
H	-1.85174200	-2.04911900	4.16060900
H	-2.86802700	-3.44164500	4.60833100
H	-3.61098100	-1.85791200	4.34404000
C	-3.87650400	1.42100400	-0.68321800
C	-3.65795300	2.35420400	-1.71845100
C	-3.90929700	3.70527100	-1.45422100
H	-3.75251100	4.43865500	-2.24916900
C	-4.35136600	4.13072600	-0.20852500
H	-4.53904200	5.19138500	-0.02482000
C	-4.55528700	3.19986400	0.80497000
H	-4.90355600	3.53826300	1.78246000
C	-4.32833300	1.83865500	0.58972000
C	-3.16198200	1.95169200	-3.09373400
H	-3.08227500	0.85562900	-3.11696500
C	-4.12059600	2.39041100	-4.20132600
H	-4.16484100	3.48788200	-4.28710400
H	-5.14705100	2.03481900	-4.02750200
H	-3.78394600	1.99948600	-5.17400900
C	-1.75821600	2.49662100	-3.35395400
H	-1.76582100	3.59538500	-3.43584800
H	-1.35761700	2.08709900	-4.29350400
H	-1.06606100	2.21451700	-2.54987600
C	-4.61296000	0.82764800	1.68365300
H	-3.93689500	-0.02345200	1.50853600
C	-6.03884500	0.28147000	1.58525000
H	-6.77656300	1.09340700	1.68768700
H	-6.22623800	-0.45189100	2.38556800
H	-6.22065600	-0.22220400	0.62626100
C	-4.34031500	1.36152900	3.08631400
H	-3.34679200	1.82714600	3.16092700
H	-4.38526300	0.54091800	3.81842100
H	-5.08998700	2.10779200	3.39315700
C	3.76684000	-0.63069600	-0.50905100
C	4.98348300	-1.23208700	-1.11653700
C	6.22209600	-0.61409200	-0.91576000
H	6.27616400	0.29557500	-0.31354700

C	7.37631200	-1.15666700	-1.47664500
H	8.34037700	-0.67068200	-1.30987600
C	7.29950900	-2.31319500	-2.25098400
H	8.20347200	-2.73509000	-2.69645900
C	6.06523300	-2.92993400	-2.45618400
H	5.99920400	-3.83387000	-3.06606200
C	4.91241800	-2.39631800	-1.88762100
H	3.94376100	-2.86943000	-2.05603500
C	3.31705500	1.21840400	-2.22936400
C	3.72464200	0.27388800	-3.36381700
H	4.76506200	-0.06425900	-3.28914200
H	3.61881400	0.80838600	-4.31947400
H	3.06554200	-0.60692000	-3.39655600
C	1.94081300	1.77892700	-2.60236500
H	1.18296500	0.98194500	-2.62330800
H	1.98586600	2.24632300	-3.59749100
H	1.61394500	2.55277300	-1.89708400
C	4.33191400	2.35866400	-2.10787100
H	5.33291500	1.96287400	-1.87930000
H	4.03176500	3.05036500	-1.30790200
H	4.39825300	2.92214500	-3.05215300
C	3.48580200	-2.05149700	1.55059300
C	4.77637700	-1.67732700	2.28466600
H	4.66851700	-0.70105100	2.77163300
H	5.63301700	-1.64039400	1.59535200
H	4.99988300	-2.42643400	3.06001400
C	2.32199000	-2.08935200	2.54347800
H	2.51751200	-2.83768300	3.32635700
H	1.38857100	-2.35226900	2.02721900
H	2.19252100	-1.10513500	3.01224900
C	3.64408700	-3.44775300	0.94348400
H	3.74116000	-4.18614700	1.75395900
H	4.53732300	-3.52805000	0.31056800
H	2.75848200	-3.70927700	0.35266400
C	-0.66163600	0.22988600	0.79866900
H	-0.78891600	1.30223300	0.57258500
C	-1.02535600	-0.00174900	2.25859800
H	-0.74085900	-1.03414900	2.51648900
H	-2.11689500	0.05667700	2.37807000
C	-0.36863800	0.97628600	3.22246200
H	0.72768400	0.95087400	3.13668700
H	-0.68913700	2.00845600	3.00840300
H	-0.63230400	0.74540600	4.26707000
C	0.15584100	4.61722600	0.34672700
C	1.66452600	4.97850500	0.56802500
B	1.43226400	2.70173900	0.35183200
O	0.15606200	3.18871900	0.50962200
O	2.32960900	3.74656000	0.24213300
H	3.92995200	2.80723800	1.72498800
C	4.02548700	1.80276700	2.17785800
O	2.83722300	1.07834900	2.02775800
H	4.85769200	1.29571800	1.64726900
C	4.38529600	1.92520400	3.64883900
H	4.56192000	0.91482400	4.05530200
H	5.34880100	2.45938800	3.72087300
C	3.32892400	2.62659500	4.48535500
H	2.35708200	2.12185000	4.38260200
H	3.59925100	2.63197100	5.55221200
H	3.19782200	3.67273500	4.16941800
C	-0.80918000	5.21006600	1.35780500
H	-0.56602600	4.89760100	2.38120300
H	-0.80202900	6.30979100	1.31220500
H	-1.82692300	4.86128300	1.12956400
C	-0.33510600	4.91017400	-1.06333400
H	0.32954200	4.47272400	-1.82144000
H	-1.32856300	4.45963800	-1.18535200
H	-0.41501800	5.99122000	-1.24813100
C	2.19910400	6.07323600	-0.33651400
H	1.66338700	7.01970900	-0.16799000

H	3.26431000	6.24060300	-0.12023800
H	2.10844800	5.80096600	-1.39564500
C	1.99103800	5.29345300	2.02197900
H	1.62134300	4.50260800	2.68918700
H	3.08270000	5.35231500	2.13962000
H	1.55710300	6.25314200	2.33782100

Cartesian coordinates of the optimized geometry of Int-**8** at PBE0-D3BJ/def2-SVP level of theory:

Ga	-1.99521900	-0.56402700	-0.59332500
Cl	-1.26516900	-0.95301500	-2.67062400
Si	2.10661400	0.31740700	0.21958200
O	0.60245800	-0.39794100	0.28437500
N	-2.87740300	-2.24832000	-0.12324900
N	-3.65669600	0.42894200	-0.95097800
N	2.92747300	-0.31150100	-1.33852700
N	3.24925800	-1.09763700	0.63346200
C	-3.98269000	-2.57710000	-0.79329400
C	-4.78365500	-1.64264200	-1.46619500
H	-5.65394300	-2.04998600	-1.97962700
C	-4.69811500	-0.23661500	-1.44055500
C	-4.38982800	-4.02097700	-0.86930900
H	-3.82544700	-4.49316200	-1.69040400
H	-5.45972200	-4.12861000	-1.08520200
H	-4.14023600	-4.56913400	0.04798800
C	-5.85749900	0.52011200	-2.02386500
H	-5.98079000	1.50548900	-1.55671600
H	-6.78815100	-0.05274800	-1.92863100
H	-5.67260300	0.68838000	-3.09662700
C	-2.26206900	-3.14985000	0.79832100
C	-1.20047000	-3.98175300	0.39181300
C	-0.57984600	-4.78521600	1.35496700
H	0.24943700	-5.43056800	1.05418300
C	-0.99888800	-4.78049800	2.67826600
H	-0.50038700	-5.41451600	3.41528400
C	-2.05667400	-3.96266800	3.06337800
H	-2.37944800	-3.96104500	4.10604600
C	-2.70115100	-3.13285300	2.14368800
C	-0.71922300	-4.03930100	-1.04325900
H	-1.35205800	-3.36617900	-1.63823800
C	-0.85510500	-5.44576300	-1.62651900
H	-0.19417600	-6.16368000	-1.11468500
H	-0.58081900	-5.44567100	-2.69301900
H	-1.88406900	-5.82663100	-1.53952700
C	0.71356000	-3.52301300	-1.16961600
H	0.81386800	-2.51724800	-0.73761400
H	1.00468200	-3.46843500	-2.23022600
H	1.42086800	-4.19411800	-0.65438700
C	-3.84884200	-2.24283000	2.58614900
H	-3.86763100	-1.38718200	1.89359400
C	-5.19974900	-2.95100100	2.46030900
H	-5.22347700	-3.85850100	3.08482500
H	-5.41819000	-3.24748100	1.42638300
H	-6.01384900	-2.28812100	2.79340900
C	-3.67731100	-1.69714800	4.00108700
H	-2.69618100	-1.22159500	4.14215500
H	-3.78392200	-2.48729200	4.76065300
H	-4.45525100	-0.94758900	4.21203800
C	-3.65518900	1.85390000	-0.83716300
C	-3.34160900	2.66889700	-1.94475300
C	-3.32066300	4.05600000	-1.76021500
H	-3.07789900	4.69933600	-2.60938600
C	-3.58588300	4.62795700	-0.52432400
H	-3.55504900	5.71308900	-0.40228700
C	-3.87911200	3.81154600	0.56282600
H	-4.07915800	4.26443200	1.53524600
C	-3.92000100	2.42198300	0.43034900

C	-3.00373100	2.10832900	-3.31222900
H	-3.10881600	1.01452300	-3.26924900
C	-3.93863200	2.64158200	-4.39840600
H	-3.80499400	3.72435300	-4.55059600
H	-4.99780600	2.47479900	-4.15199000
H	-3.72938100	2.14711100	-5.35961400
C	-1.54529900	2.39576700	-3.66665800
H	-1.36177000	3.47790600	-3.76458100
H	-1.27768500	1.91476700	-4.61971300
H	-0.86895900	2.00322000	-2.89750200
C	-4.27842000	1.54539100	1.61327200
H	-3.75726800	0.58804200	1.46056700
C	-5.77480700	1.22969900	1.64402100
H	-6.36706800	2.15382800	1.73817500
H	-6.01593800	0.58231100	2.50214400
H	-6.10258600	0.70903100	0.73355600
C	-3.81587100	2.11498400	2.94978000
H	-2.75449200	2.40118600	2.92461300
H	-3.94366900	1.36652700	3.74552500
H	-4.40100500	3.00060500	3.24312500
C	3.68848300	-1.13784400	-0.62128800
C	4.83203400	-1.93536200	-1.12960400
C	6.13530400	-1.43761900	-1.04417300
H	6.31106300	-0.45654900	-0.59804500
C	7.20055400	-2.19262300	-1.53026900
H	8.21716900	-1.79874500	-1.46407000
C	6.96985000	-3.44471100	-2.09783200
H	7.80700100	-4.03538500	-2.47692300
C	5.66975000	-3.94214500	-2.18259100
H	5.48487500	-4.92222700	-2.62786700
C	4.60097400	-3.18997300	-1.70269000
H	3.57982300	-3.57025700	-1.77044100
C	3.08481200	0.21564500	-2.69747900
C	3.09932600	-0.91246300	-3.72867500
H	3.99914700	-1.53704400	-3.64851000
H	3.08002200	-0.48198400	-4.74106100
H	2.20881000	-1.54763600	-3.60927600
C	1.85630900	1.08817200	-2.92920700
H	0.93495900	0.49460600	-2.84575400
H	1.89003100	1.54857600	-3.92714400
H	1.84054600	1.89713100	-2.18507000
C	4.34535600	1.07976700	-2.79014500
H	5.25599100	0.47448100	-2.67391300
H	4.32144500	1.85690600	-2.01010400
H	4.39434100	1.57014600	-3.77461500
C	3.69312500	-1.79221500	1.84474000
C	5.09337600	-1.32245100	2.24725000
H	5.10793800	-0.22415800	2.28767000
H	5.85625300	-1.66932600	1.53638500
H	5.35492500	-1.71871800	3.24014900
C	2.70249500	-1.40209800	2.93837900
H	2.97011700	-1.89308100	3.88507300
H	1.68136000	-1.70447400	2.66398400
H	2.73049300	-0.31288600	3.08680800
C	3.65547500	-3.30837000	1.64750100
H	3.90084900	-3.80891600	2.59624200
H	4.38121100	-3.64488500	0.89446100
H	2.64910700	-3.62853000	1.33933900
C	-0.64465500	0.18166200	0.65414000
H	-0.61265500	1.27227400	0.49495500
C	-0.94291500	-0.07971900	2.12483500
H	-0.74833000	-1.14581200	2.32833000
H	-2.02080200	0.06161800	2.29701900
C	-0.17378000	0.80629700	3.09187100
H	0.91161300	0.73600600	2.94299300
H	-0.42631900	1.86657900	2.94690000
H	-0.38723000	0.53342800	4.13761200
C	1.20984000	4.07954100	0.51864800
C	2.59676300	4.32142500	-0.18742000

B	2.62524700	2.26779900	0.95758100
O	1.49693500	3.03912800	1.42612200
O	3.22584200	3.05407800	-0.10192500
H	4.47327200	3.55545500	2.26913000
C	4.38580800	2.55077300	2.71553700
O	3.49564900	1.74981000	1.98842700
H	5.39433600	2.09727900	2.65142000
C	3.99495600	2.65730600	4.18228800
H	3.89059400	1.63190500	4.57911600
H	4.83773700	3.11385400	4.73192200
C	2.71801900	3.44246000	4.43238700
H	1.88984100	3.04724200	3.82749500
H	2.42971000	3.41602400	5.49482100
H	2.84402200	4.50050300	4.14876000
C	0.68130100	5.27701900	1.29677700
H	1.36326700	5.56234000	2.10738100
H	0.52635200	6.14575400	0.63809600
H	-0.28767000	5.01697300	1.74897400
C	0.12394500	3.62279600	-0.45513300
H	0.43862400	2.73735200	-1.02321700
H	-0.78368900	3.36115400	0.10830300
H	-0.15194200	4.40763800	-1.17368300
C	2.49796700	4.73846800	-1.64844400
H	1.95760500	5.69212600	-1.75297000
H	3.50932400	4.87474800	-2.06060000
H	1.98789800	3.98272700	-2.25814500
C	3.45250200	5.35197600	0.54966800
H	3.47799200	5.15534500	1.62951100
H	4.48216300	5.28950000	0.16748300
H	3.08596300	6.37810000	0.39644700

Cartesian coordinates of the optimized geometry of TS-**1** at PBE0-D3BJ/def2-SVP level of theory:

Ga	0.93009400	0.24526600	-0.77402900
Cl	0.84652500	-0.01866800	-3.02334300
Si	-0.72085300	-1.24718800	0.23038400
N	2.80575300	-0.33820300	-0.44784300
N	1.36902000	2.16331800	-0.62461400
C	3.79083700	0.40778100	-0.92108200
C	3.59537100	1.70731800	-1.43880200
H	4.45798100	2.15402700	-1.93219800
C	2.50260500	2.54873200	-1.23424900
C	5.20441800	-0.10131000	-0.93427000
H	5.46080000	-0.41879100	-1.95703800
H	5.34331000	-0.95944200	-0.26617700
H	5.90175300	0.69921000	-0.65378100
C	2.60548600	3.95785300	-1.74803600
H	3.63798600	4.21082100	-2.01669900
H	2.22609700	4.69311600	-1.02677100
H	1.97964100	4.04462300	-2.65117900
C	3.04296600	-1.61814800	0.14234200
C	3.26545000	-2.74958600	-0.67000000
C	3.43701300	-3.98882200	-0.04270700
H	3.60863300	-4.87570900	-0.65777100
C	3.38755500	-4.11278200	1.33852700
H	3.51397400	-5.09143900	1.80736200
C	3.16848400	-2.98721000	2.12564400
H	3.11561600	-3.09177200	3.20940400
C	2.99576500	-1.72671900	1.55116600
C	3.32401800	-2.68127300	-2.18439400
H	3.17063500	-1.63513000	-2.48717600
C	4.67516100	-3.15311600	-2.72502700
H	4.82936900	-4.22646500	-2.53110800
H	5.51779200	-2.61479300	-2.26845700
H	4.72499600	-3.00573100	-3.81521700
C	2.19136400	-3.49000400	-2.81207800
H	1.22017100	-3.17505100	-2.41448500

H	2.30788900	-4.56720500	-2.61165500
H	2.17089100	-3.34697100	-3.90332000
C	2.80639800	-0.49987200	2.42263700
H	2.05901700	0.13539900	1.91532700
C	4.10648600	0.30289100	2.52152500
H	4.91212700	-0.32566100	2.93290000
H	3.97738400	1.16412500	3.19458800
H	4.44014100	0.69048600	1.54956600
C	2.26673000	-0.81407600	3.81288500
H	3.02775800	-1.31118000	4.43640000
H	1.37106200	-1.45276600	3.75295100
H	1.99610000	0.12231900	4.32416200
C	0.48773500	3.15971600	-0.10852000
C	-0.59348100	3.62849800	-0.87924400
C	-1.43942000	4.59723800	-0.32841700
H	-2.28473500	4.96410200	-0.91582800
C	-1.21799900	5.10512300	0.94429900
H	-1.88807400	5.86190900	1.35888500
C	-0.13794300	4.64555400	1.69079800
H	0.03048000	5.04953400	2.69048100
C	0.73078600	3.67318500	1.18910000
C	-0.86049000	3.11237700	-2.27706700
H	-0.00669200	2.48981400	-2.58058100
C	-0.99842500	4.23955100	-3.29879800
H	-1.89652100	4.85182500	-3.11943000
H	-1.08179700	3.82343300	-4.31458800
H	-0.12932600	4.91455700	-3.27775700
C	-2.08922800	2.20871000	-2.28480700
H	-1.95397400	1.35172100	-1.60809800
H	-2.27556300	1.81039200	-3.29284700
H	-2.98419900	2.76621700	-1.96240700
C	1.91702400	3.20735400	2.01661600
H	2.11656100	2.16640400	1.71968900
C	3.18276600	4.01530700	1.71504500
H	4.00444600	3.69081400	2.37227400
H	3.01088900	5.08913000	1.89291500
H	3.52567900	3.88936200	0.68091800
C	1.65306500	3.23086500	3.51977500
H	2.47407500	2.72870600	4.05294200
H	0.71761100	2.72086400	3.78303200
H	1.60350100	4.26000800	3.90914000
N	-2.11290200	-0.27353200	0.96950500
N	-2.22828500	-1.33050000	-0.88870400
C	-2.96107700	-0.67592900	0.02168000
C	-4.43477500	-0.50189000	0.01912200
C	-5.23866300	-1.34967200	0.79026200
H	-4.77361900	-2.13764700	1.38555700
C	-6.62275700	-1.20246600	0.78058100
H	-7.24199100	-1.87227400	1.38134800
C	-7.21538300	-0.20624800	0.00522200
H	-8.30172100	-0.09089700	-0.00030000
C	-6.41886600	0.64134100	-0.76248400
H	-6.87733700	1.42535600	-1.36930500
C	-5.03351800	0.49405000	-0.75846100
H	-4.40770600	1.16309500	-1.34975400
C	-2.63318500	-2.10942700	-2.06441800
C	-1.41267600	-2.92992600	-2.46903800
H	-1.11173100	-3.61947800	-1.66563400
H	-0.57421000	-2.25876400	-2.70350200
H	-1.63201500	-3.52485800	-3.36742200
C	-3.80060100	-3.05190300	-1.76010200
H	-3.58677200	-3.66497900	-0.87219600
H	-3.95409100	-3.72827400	-2.61426700
H	-4.74034300	-2.50964900	-1.59234500
C	-2.99130100	-1.16991000	-3.21759800
H	-3.85079700	-0.53374000	-2.96322100
H	-3.25739700	-1.75619400	-4.11027300
H	-2.13075100	-0.53238900	-3.46315300
C	-2.31052800	0.50325500	2.19716500

C	-3.05839700	-0.32102300	3.24764700
H	-2.51294800	-1.26210100	3.41769200
H	-4.09262200	-0.52711700	2.93954500
H	-3.09808300	0.23722100	4.19550100
C	-3.03375000	1.81317400	1.88704700
H	-3.06955000	2.43715300	2.79244600
H	-4.06801800	1.64348100	1.55588000
H	-2.49794900	2.37787200	1.10923800
C	-0.90876900	0.79497600	2.72028600
H	-0.38712400	-0.15368200	2.92325600
H	-0.97184600	1.36129100	3.66022800
H	-0.34359200	1.39678800	1.99355000
O	-0.45056400	-2.32267100	2.94367200
C	-0.46645600	-2.90862800	1.82841300
H	0.48829300	-3.31753200	1.41809600
C	-1.66224700	-3.79586600	1.48717000
H	-1.55620500	-4.18796400	0.46226700
H	-2.58851000	-3.19532000	1.51985400
C	-1.76704500	-4.93652700	2.49317700
H	-1.85816800	-4.53161200	3.51128300
H	-2.63698100	-5.57997700	2.28872500
H	-0.86519800	-5.56919900	2.46592100

Cartesian coordinates of the optimized geometry of TS-**2** at PBE0-D3BJ/def2-SVP level of theory:

Ga	0.93454100	-0.02527800	-0.69791500
Cl	0.82465300	-0.80388600	-2.81312200
Si	-1.03683500	-0.41950400	0.52458300
N	2.93348100	-0.19969600	-0.46086100
N	1.08170300	1.96257200	-1.04607100
C	3.72877100	0.56362600	-1.21543400
C	3.31385000	1.71367200	-1.90135400
H	4.06972100	2.19275100	-2.52145000
C	2.12684500	2.43563300	-1.71955300
C	5.18468100	0.20724100	-1.35082200
H	5.29340300	-0.70980400	-1.94683600
H	5.64319500	0.00037500	-0.37440500
H	5.73789400	1.01326700	-1.84648700
C	2.10698000	3.83848000	-2.26406300
H	2.41602700	4.55025500	-1.48336200
H	1.10454300	4.13913600	-2.59344500
H	2.81300000	3.93071700	-3.09946100
C	3.56071300	-1.22968500	0.31090900
C	3.86934000	-2.48097800	-0.26119900
C	4.51061900	-3.43741300	0.53452500
H	4.74967700	-4.41205500	0.10216800
C	4.85057900	-3.17331200	1.85324700
H	5.35525200	-3.93268900	2.45518000
C	4.53676900	-1.93705600	2.40722700
H	4.79494400	-1.73644000	3.44827700
C	3.88592900	-0.95362300	1.65966200
C	3.51698100	-2.84371200	-1.69038300
H	3.11342800	-1.94672100	-2.18142400
C	4.72219100	-3.33221100	-2.49455300
H	5.10921200	-4.28822200	-2.10769600
H	5.55633400	-2.61458000	-2.48250900
H	4.43483700	-3.49626300	-3.54460500
C	2.40974700	-3.89532200	-1.70908700
H	1.55656500	-3.57124000	-1.10210700
H	2.76688700	-4.85675200	-1.30521700
H	2.04958900	-4.06418300	-2.73532000
C	3.53747900	0.38029500	2.29651800
H	2.66388300	0.77214300	1.75194200
C	4.66347300	1.40178800	2.12597200
H	5.59709600	1.03796800	2.58434900
H	4.39840800	2.35458400	2.61117800
H	4.86481800	1.61594800	1.06697100

C	3.14064800	0.24082300	3.76332400
H	3.98967200	-0.06534700	4.39389700
H	2.33346600	-0.49577400	3.88971500
H	2.78161600	1.20259200	4.15531100
C	0.06311200	2.88474800	-0.67354500
C	-1.16594500	2.91845300	-1.37179900
C	-2.09326600	3.90816500	-1.03070400
H	-3.04079700	3.96047600	-1.56890400
C	-1.83260400	4.83584400	-0.02781600
H	-2.57065500	5.60430000	0.21376300
C	-0.64010200	4.76347800	0.67869600
H	-0.45044100	5.46976600	1.49035300
C	0.31776400	3.78995000	0.37992100
C	-1.46694500	1.93732300	-2.48788000
H	-1.02127300	0.97550400	-2.19294200
C	-0.81139400	2.33393500	-3.81022000
H	-1.14851500	3.33105400	-4.13787400
H	-1.07230800	1.60591600	-4.59325900
H	0.28341800	2.33846800	-3.73389000
C	-2.95911800	1.69556000	-2.67999900
H	-3.44204800	1.43927600	-1.72467800
H	-3.11601600	0.86321700	-3.38108900
H	-3.47508100	2.57367600	-3.10012200
C	1.56196200	3.69162600	1.24210600
H	2.26720700	3.00734600	0.74798900
C	2.27284500	5.03063500	1.42865800
H	3.22261200	4.88578200	1.96640500
H	1.66900200	5.73727300	2.01938900
H	2.50040100	5.51375400	0.46644200
C	1.19847000	3.07602500	2.59367400
H	2.08269800	3.02496300	3.24615000
H	0.79610500	2.05910700	2.47906900
H	0.43549400	3.68092900	3.10870700
N	-2.80605600	0.14085200	0.98895500
N	-2.30603500	-1.67552200	-0.07976300
C	-3.31427900	-0.92841100	0.40812400
C	-4.76154500	-1.25399500	0.33200200
C	-5.31351200	-2.16481000	1.23906300
H	-4.67003500	-2.64190400	1.98112100
C	-6.67091700	-2.46647400	1.18634900
H	-7.09553400	-3.17798100	1.89805300
C	-7.48456900	-1.86736100	0.22474200
H	-8.54896000	-2.10921600	0.18110900
C	-6.93751700	-0.95826600	-0.67905500
H	-7.57044100	-0.48504000	-1.43302200
C	-5.58090900	-0.64580000	-0.62301300
H	-5.15310500	0.07239100	-1.32483800
C	-2.40405200	-2.68653900	-1.15555900
C	-1.05851900	-3.38777300	-1.26378800
H	-0.83721500	-3.96810100	-0.35971500
H	-0.25450200	-2.66505600	-1.43680400
H	-1.07076100	-4.07509500	-2.12192700
C	-3.46347200	-3.75170000	-0.85530500
H	-3.33757500	-4.15475300	0.16058500
H	-3.33353200	-4.58111600	-1.56618000
H	-4.49071400	-3.38332500	-0.96382300
C	-2.71692900	-1.99417000	-2.48441800
H	-3.67776200	-1.46164600	-2.43840800
H	-2.78309300	-2.73831600	-3.29260200
H	-1.91810800	-1.28505700	-2.74484500
C	-3.36725800	1.06747000	1.97679600
C	-3.86643400	0.29635900	3.20162300
H	-3.07056900	-0.36455600	3.57665300
H	-4.75496100	-0.30712000	2.96979800
H	-4.13913400	1.00037500	4.00234200
C	-4.48601300	1.91671300	1.37202000
H	-4.81824100	2.66770600	2.10469300
H	-5.35819700	1.30965000	1.09356900
H	-4.12050400	2.44903100	0.48157700

C	-2.21310200	1.97233400	2.39902900
H	-1.42041900	1.37772200	2.87435400
H	-2.56495800	2.73057900	3.11350200
H	-1.78970400	2.49182900	1.52895100
O	-0.43278900	-0.69973100	2.06107500
C	0.56575300	-1.51835800	1.39882500
H	1.55804300	-1.14735600	1.68645500
C	0.44797900	-2.97162500	1.78338100
H	1.11957400	-3.56621900	1.14136000
H	-0.57795400	-3.32104500	1.57954900
C	0.78403700	-3.22089300	3.25081700
H	0.63652600	-4.27597500	3.53398500
H	1.83431600	-2.96213000	3.45983500
H	0.14605500	-2.59975400	3.89836800

Cartesian coordinates of the optimized geometry of TS-**3** at PBE0-D3BJ/def2-SVP level of theory:

O	-1.59707400	-0.96737900	0.46106300
C	-2.35849000	-0.62835000	-0.48016800
H	-2.68583700	-1.39367600	-1.22249100
C	-3.23049700	0.59277600	-0.38969000
H	-3.54005600	0.90207700	-1.40024000
H	-2.64237900	1.40231000	0.06640300
C	-4.44594600	0.25976800	0.47336500
H	-4.13212800	-0.03460700	1.48524500
H	-5.10433600	1.13592000	0.55882600
H	-5.03932800	-0.56382100	0.04578600
H	-1.24745400	-0.05548200	-1.62191000
C	1.41480800	0.86390900	0.12404100
C	1.64995400	-0.68708800	-0.00696600
B	-0.41923500	-0.18121200	-0.67017300
O	-0.00418000	0.98564900	-0.04521800
O	0.60803000	-1.07102700	-0.91793600
C	1.43617800	-1.42795300	1.30963700
H	1.39132200	-2.50629600	1.09991300
H	2.25992000	-1.24427300	2.01474400
H	0.48838100	-1.13924200	1.78280300
C	2.98440500	-1.07597000	-0.61446900
H	3.81826600	-0.70552900	0.00104700
H	3.05902300	-2.17194900	-0.66625900
H	3.09243000	-0.68168600	-1.63281900
C	1.80121400	1.44784000	1.47014400
H	2.87283400	1.29721600	1.67168200
H	1.60186900	2.52955300	1.47447600
H	1.22011100	0.99348200	2.28238200
C	2.07287400	1.65174800	-1.00366900
H	1.69013800	2.68237100	-0.98196600
H	3.16731600	1.68508200	-0.90066800
H	1.82488300	1.21659000	-1.98270500

Cartesian coordinates of the optimized geometry of TS-**4** at PBE0-D3BJ/def2-SVP level of theory:

Ga	-0.95710500	-1.46182200	-0.65873900
Cl	-0.00840800	-1.27575200	-2.68025100
Si	2.00522500	1.38551000	0.63120400
O	1.00505600	0.04625700	0.52362000
N	-0.62390700	-3.35982700	-0.23923100
N	-2.85435600	-1.70783400	-1.11076700
N	3.02771200	1.38812400	-0.95276000
N	3.67687200	0.58086000	0.92375800
C	-1.24771500	-4.26965700	-0.98460200
C	-2.38637800	-3.98823100	-1.75936200
H	-2.76226900	-4.81501600	-2.36018300
C	-3.18954100	-2.83570400	-1.74057200
C	-0.71408900	-5.67305400	-1.04302100

H	-1.50069300	-6.38736900	-1.31487600
H	-0.24926700	-5.97790800	-0.09689300
H	0.06873800	-5.71749900	-1.81749800
C	-4.49495900	-2.89507100	-2.48187300
H	-4.46027800	-2.20743600	-3.34091500
H	-5.33106900	-2.56049600	-1.85246900
H	-4.69803000	-3.90851400	-2.84665200
C	0.40130800	-3.73293300	0.68368200
C	1.74272000	-3.83248900	0.26420300
C	2.71565100	-4.15184900	1.21775800
H	3.75991400	-4.23111600	0.90520100
C	2.38131900	-4.36908600	2.54705200
H	3.15704000	-4.61256800	3.27689700
C	1.05222500	-4.27401500	2.94662200
H	0.79457300	-4.44678900	3.99274300
C	0.04311800	-3.95832600	2.03384300
C	2.16503300	-3.60030400	-1.17169200
H	1.25900100	-3.43408800	-1.77089300
C	2.90635300	-4.80361800	-1.75366300
H	3.87315700	-4.96815400	-1.25136100
H	3.11467400	-4.64245400	-2.82280500
H	2.32443500	-5.73256000	-1.65623300
C	3.00378600	-2.33014200	-1.28976600
H	2.47402300	-1.46202600	-0.87435700
H	3.22868500	-2.11915400	-2.34660100
H	3.95856200	-2.44302800	-0.75065800
C	-1.40588000	-3.89302100	2.48016200
H	-1.89577600	-3.14146500	1.84295000
C	-2.12821500	-5.22172600	2.24495000
H	-1.63160000	-6.03817600	2.79330200
H	-2.15782800	-5.49514000	1.18222900
H	-3.16960600	-5.16048100	2.59770200
C	-1.57656100	-3.45544400	3.93123400
H	-1.03308200	-2.52418000	4.14672400
H	-1.22691200	-4.22616200	4.63603700
H	-2.64134000	-3.28476300	4.14916100
C	-3.84027400	-0.68066300	-0.96000100
C	-4.02002900	0.29743400	-1.95915400
C	-5.06752700	1.21221400	-1.81079800
H	-5.22459100	1.96784400	-2.58362500
C	-5.90549100	1.17984200	-0.70446900
H	-6.72108700	1.90085200	-0.61215100
C	-5.68242200	0.24495200	0.29951900
H	-6.32155800	0.24421500	1.18461800
C	-4.64870000	-0.68920400	0.19847200
C	-3.09167600	0.41626500	-3.14846100
H	-2.45702000	-0.48084700	-3.17716700
C	-3.83305600	0.50056600	-4.48092200
H	-4.42205600	1.42728800	-4.56764800
H	-4.52337300	-0.34568100	-4.62289500
H	-3.11367800	0.49270300	-5.31404100
C	-2.16359200	1.61730100	-2.95839300
H	-2.72388800	2.56507800	-2.96144000
H	-1.41375500	1.65511700	-3.76259800
H	-1.62576800	1.57257700	-2.00102800
C	-4.42120600	-1.69406000	1.31074500
H	-3.38053800	-2.03734500	1.21175200
C	-5.31203900	-2.92844300	1.16478400
H	-6.37712000	-2.64606200	1.16753000
H	-5.14536900	-3.62711600	1.99984200
H	-5.10695700	-3.47337900	0.23263100
C	-4.57704500	-1.07220400	2.69649500
H	-3.99113700	-0.14592300	2.79338600
H	-4.23543200	-1.77527800	3.47091400
H	-5.62721900	-0.82968300	2.92168200
C	4.09678900	0.95709900	-0.28486800
C	5.50836600	0.99365800	-0.74679500
C	6.32383700	2.07581300	-0.40004000
H	5.90986800	2.89744400	0.18876000

C	7.65383200	2.10960600	-0.81299900
H	8.28420800	2.95942700	-0.54158400
C	8.17694700	1.06299200	-1.57093000
H	9.22056300	1.08929700	-1.89270100
C	7.36541100	-0.01605900	-1.91935600
H	7.77104300	-0.83781600	-2.51371300
C	6.03438400	-0.05205700	-1.51165900
H	5.39690600	-0.89695400	-1.77709500
C	2.87751700	2.02309100	-2.26418300
C	3.61369100	3.36450800	-2.31233800
H	3.26902500	4.02631400	-1.50265700
H	3.41608700	3.87092100	-3.26904200
H	4.70135900	3.23410300	-2.21761500
C	3.37857100	1.09428800	-3.37170600
H	4.46445600	0.93674800	-3.31726200
H	3.15266700	1.53671400	-4.35345600
H	2.86576300	0.12382500	-3.30761100
C	1.37695100	2.23922400	-2.45353300
H	0.94188200	2.86267000	-1.65813500
H	0.85378600	1.27291600	-2.45406200
H	1.18308500	2.73769200	-3.41423000
C	4.36306600	0.06780600	2.10637600
C	5.04204200	1.21140500	2.86638700
H	4.31149300	1.99946700	3.10473800
H	5.85393600	1.65488500	2.27193700
H	5.47419600	0.84544000	3.81033000
C	3.27405400	-0.55709900	2.98057300
H	3.71321200	-0.97896300	3.89632500
H	2.74690100	-1.35441400	2.43769900
H	2.53232200	0.19971000	3.27970300
C	5.38751600	-1.00774600	1.74436000
H	5.78924000	-1.45340900	2.66649300
H	6.23216000	-0.60201500	1.17190500
H	4.91294000	-1.80667300	1.15711300
C	-0.40779500	-0.07727100	0.64446400
H	-0.88867400	0.87076500	0.35309500
C	-0.80756000	-0.40402600	2.07530000
H	-0.24786700	-1.29737100	2.39690000
H	-1.87235200	-0.68874200	2.07564600
C	-0.59836400	0.72374100	3.07324400
H	0.46495500	1.00474700	3.13033700
H	-1.15516800	1.62847400	2.78726400
H	-0.91071400	0.41997200	4.08507100
C	1.04036800	3.62457500	1.01691000
O	-0.10335700	2.94576900	0.65917100
H	1.69361800	4.00214200	0.22024600
C	1.44451300	3.88197400	2.46342500
H	0.94946600	3.12000300	3.08053800
H	2.52775800	3.69463400	2.53468300
C	1.12712000	5.26856500	3.01492600
H	1.52164900	5.36339500	4.03905700
H	1.58857300	6.05921500	2.40263800
H	0.04174300	5.43870800	3.03805900
H	0.17815900	4.94782000	0.82062900
C	-2.83081400	5.12560800	-0.08554200
C	-3.08115100	4.56679800	1.35997900
O	-1.76118200	4.34077200	1.82740300
O	-1.68033000	4.39741400	-0.48811800
C	-3.78903100	5.52814600	2.29899400
H	-4.78600700	5.79749300	1.91665100
H	-3.91786500	5.05398900	3.28358200
H	-3.20503600	6.44662700	2.44287800
C	-3.81104400	3.22479500	1.33722200
H	-3.34793300	2.53304100	0.61823000
H	-3.74337800	2.77170000	2.33769700
H	-4.87430800	3.32920600	1.07677000
C	-2.48397000	6.61334900	-0.07717300
H	-2.09314900	6.88678700	-1.06832700
H	-3.35845800	7.24468000	0.13965100

H	-1.70243300	6.82687100	0.66687000
C	-3.95524100	4.85636600	-1.06938100
H	-4.89138900	5.33426200	-0.74094600
H	-3.68993000	5.26518900	-2.05605300
H	-4.12841000	3.77896200	-1.18257800
B	-0.93417800	4.13351500	0.68268500

Cartesian coordinates of the optimized geometry of TS-**5** at PBE0-D3BJ/def2-SVP level of theory:

Ga	1.46758400	0.15398200	-0.74425500
Cl	0.54502800	-0.00223500	-2.78328400
Si	-2.19476600	-1.24525600	1.04091800
O	-0.80943500	-0.36982100	0.71755400
N	1.82599800	2.08226800	-0.65117800
N	3.34032400	-0.35667500	-1.05783200
N	-3.21128400	-1.19796300	-0.54403100
N	-3.47595100	0.11618600	1.12911400
C	2.72093000	2.55925100	-1.51794300
C	3.67049200	1.75571600	-2.16996400
H	4.29480000	2.26295900	-2.90547200
C	4.04294600	0.43219500	-1.86669700
C	2.71389300	4.02461300	-1.84904100
H	1.92656200	4.21617800	-2.59562400
H	3.67370100	4.34253000	-2.27349000
H	2.47779600	4.64039800	-0.97133500
C	5.29673300	-0.07408000	-2.52392900
H	5.13975800	-0.06488900	-3.61388400
H	5.54684100	-1.09539500	-2.21454200
H	6.14862000	0.58733600	-2.31387900
C	1.01133000	2.95066700	0.13760000
C	-0.21107000	3.43935700	-0.36632500
C	-0.99660500	4.25111100	0.45889000
H	-1.94559200	4.63622700	0.07736900
C	-0.59573800	4.57352200	1.74739600
H	-1.22504900	5.20542800	2.37858400
C	0.61180300	4.08380900	2.23336100
H	0.91995900	4.33896800	3.24815700
C	1.43413800	3.27037000	1.44930200
C	-0.71435900	3.09773400	-1.75299100
H	0.06922200	2.52829800	-2.27170300
C	-1.02301100	4.34384900	-2.58193800
H	-1.86851000	4.91377600	-2.16436700
H	-1.29458900	4.05957300	-3.61042500
H	-0.16167000	5.02706100	-2.63401300
C	-1.93346600	2.18202300	-1.66520400
H	-1.71764600	1.28196500	-1.07262400
H	-2.24494400	1.86231200	-2.67140700
H	-2.77899400	2.70837800	-1.19266100
C	2.76180300	2.77318800	1.99318700
H	2.93742400	1.78200400	1.54548300
C	3.92083800	3.67365300	1.55829300
H	3.76138000	4.70889900	1.89997300
H	4.04443100	3.69044400	0.46741500
H	4.86859800	3.31695700	1.99099700
C	2.76170300	2.61288600	3.51041400
H	1.90815300	2.01222400	3.85819900
H	2.72373900	3.58625600	4.02410800
H	3.68362500	2.11383900	3.83987400
C	3.89274600	-1.52919800	-0.46368500
C	3.35120000	-2.79871200	-0.77951200
C	3.87223200	-3.92227600	-0.13501600
H	3.45730700	-4.90436800	-0.36339800
C	4.90541100	-3.81579900	0.78896000
H	5.30033000	-4.71008500	1.27652700
C	5.42033300	-2.56557200	1.09762400
H	6.22035100	-2.48106400	1.83737000
C	4.92098300	-1.40423100	0.49861200

C	2.27466600	-2.95852200	-1.83378000
H	1.59724800	-2.09547100	-1.76353000
C	2.88022800	-2.90214100	-3.23685300
H	3.57137300	-3.74524000	-3.39847800
H	3.43382400	-1.96757200	-3.40136500
H	2.08594200	-2.95115900	-3.99689800
C	1.40546900	-4.19759200	-1.65581500
H	1.97255900	-5.12804900	-1.82060500
H	0.59797500	-4.18193400	-2.40276000
H	0.93470700	-4.23444600	-0.66124000
C	5.48581700	-0.06755700	0.94702100
H	4.94423200	0.73120900	0.41840300
C	6.97262000	0.07014800	0.61472300
H	7.57373200	-0.65659400	1.18386600
H	7.33637200	1.07655300	0.87530000
H	7.17495600	-0.10084400	-0.45126800
C	5.26123000	0.14225700	2.44471900
H	4.19837800	0.05441900	2.70938900
H	5.61223700	1.13925400	2.75293100
H	5.81410700	-0.60052300	3.04021900
C	-4.03956400	-0.30455400	-0.00561700
C	-5.39198000	0.06611200	-0.49673500
C	-6.51070200	-0.61857700	-0.01112200
H	-6.37712100	-1.42879000	0.70928500
C	-7.78691600	-0.27316000	-0.45050500
H	-8.65609000	-0.81391000	-0.06924100
C	-7.95263800	0.75759300	-1.37412000
H	-8.95369500	1.02864700	-1.71732600
C	-6.83843700	1.44050300	-1.86084100
H	-6.96360300	2.24771500	-2.58597700
C	-5.56102500	1.09686200	-1.42634500
H	-4.68643300	1.62889500	-1.80375800
C	-3.31856800	-2.05030900	-1.73169900
C	-4.41455100	-3.10278400	-1.54315400
H	-4.22520600	-3.70157100	-0.63908800
H	-4.43710900	-3.78649200	-2.40514200
H	-5.40702900	-2.63702600	-1.45518100
C	-3.59408600	-1.21182300	-2.98113400
H	-4.58880000	-0.74659600	-2.95898600
H	-3.54635000	-1.85742100	-3.87072400
H	-2.82859200	-0.42986200	-3.08739300
C	-1.95579000	-2.72543000	-1.89504100
H	-1.65214800	-3.29108900	-1.00096400
H	-1.18145400	-1.97190000	-2.10024400
H	-1.98613900	-3.42373200	-2.74451100
C	-3.89898900	1.04022200	2.17754200
C	-4.94762700	0.38112500	3.07855900
H	-4.56576600	-0.57291400	3.47327300
H	-5.87728300	0.18381500	2.52516300
H	-5.19231600	1.03509900	3.92960600
C	-2.63917900	1.34952000	2.98929200
H	-2.87218200	2.04865300	3.80556100
H	-1.86203900	1.79274800	2.35014500
H	-2.23002300	0.43002400	3.43743300
C	-4.44306800	2.34278900	1.59051400
H	-4.63602800	3.05811000	2.40391000
H	-5.38483700	2.19078500	1.04649600
H	-3.70624800	2.79078500	0.90859500
C	0.55242600	-0.79187800	0.72883200
H	0.60347300	-1.87500400	0.52736300
C	1.20836700	-0.52978200	2.07531500
H	1.12731300	0.54358700	2.31248300
H	2.28448200	-0.75147600	1.96552000
C	0.64048100	-1.35843000	3.21523300
H	-0.41465400	-1.09994400	3.40073600
H	0.67610300	-2.42962100	2.96682200
H	1.19030700	-1.18570000	4.15365000
C	-1.81789800	-3.49292800	1.55386700
O	-0.65348000	-3.68751800	1.13641900

H	-2.68255700	-3.78147800	0.89450200
C	-2.16994300	-3.70908200	3.01924400
H	-1.50235100	-3.11006400	3.65512100
H	-3.20153100	-3.36016100	3.19763100
C	-2.02208700	-5.18573200	3.36792900
H	-2.26599100	-5.37725400	4.42430500
H	-2.68712300	-5.81180500	2.75044000
H	-0.98947700	-5.51626700	3.18337600

Cartesian coordinates of the optimized geometry of TS-**6** at PBE0-D3BJ/def2-SVP level of theory:

Ga	-1.55606900	-0.97257400	-0.70837800
Cl	-0.76492200	-1.05108400	-2.80585300
Si	2.18386900	0.63688400	0.71001900
O	0.77491600	-0.15624400	0.47528700
N	-1.58054100	-2.90106500	-0.27847100
N	-3.48813400	-0.84363800	-1.01643200
N	3.18765600	0.75583800	-0.82633100
N	3.54581800	-0.58596700	0.82421900
C	-2.46167200	-3.66615700	-0.92674900
C	-3.55083000	-3.16341300	-1.65689800
H	-4.13181800	-3.90197800	-2.20686200
C	-4.08336000	-1.86581500	-1.62979600
C	-2.30583100	-5.16098800	-0.89140500
H	-3.20413200	-5.65647400	-1.27721800
H	-2.10167400	-5.52029100	0.12645400
H	-1.44725700	-5.46435700	-1.50868000
C	-5.37882600	-1.63741600	-2.35383200
H	-6.01780500	-0.90697500	-1.84275000
H	-5.92979100	-2.57422300	-2.49918300
H	-5.14340700	-1.21806800	-3.34609200
C	-0.58317500	-3.53127200	0.52633200
C	0.57593500	-4.07211600	-0.06921400
C	1.47880300	-4.76142600	0.74634900
H	2.36970500	-5.20612800	0.29644800
C	1.26261200	-4.89810700	2.11171000
H	1.97909300	-5.44450900	2.72970100
C	0.13827500	-4.32112200	2.69113900
H	-0.01843800	-4.41446800	3.76718000
C	-0.79918100	-3.63037200	1.91788700
C	0.88286700	-3.90220300	-1.54401200
H	-0.01565600	-3.50669800	-2.03841100
C	1.27003500	-5.21004500	-2.23166600
H	2.23300500	-5.59921900	-1.86413500
H	1.37550600	-5.05074000	-3.31567900
H	0.51834800	-5.99931800	-2.07807300
C	1.97525200	-2.85045700	-1.73326600
H	1.69903200	-1.90067600	-1.25603100
H	2.14049900	-2.65740100	-2.80407600
H	2.92238700	-3.19947100	-1.29065500
C	-2.04186800	-3.04488600	2.55986000
H	-2.34475100	-2.18804100	1.93814900
C	-3.20484100	-4.03896500	2.54360800
H	-2.93525000	-4.96651300	3.07357100
H	-3.50250400	-4.30469200	1.51973500
H	-4.08758500	-3.60901900	3.04179000
C	-1.79205900	-2.52335300	3.97149600
H	-0.91431100	-1.86123100	4.00740900
H	-1.63230200	-3.34191200	4.69081300
H	-2.66222700	-1.94953300	4.32202200
C	-4.22786700	0.34077300	-0.71505800
C	-4.12811200	1.47697000	-1.54073100
C	-4.83795300	2.62468900	-1.17349600
H	-4.76064500	3.51911300	-1.79622400
C	-5.64011600	2.64537900	-0.03987100
H	-6.18758900	3.55202700	0.22766100
C	-5.73683900	1.51071400	0.75885200

H	-6.36136900	1.53628100	1.65464200
C	-5.02637400	0.34838400	0.45126000
C	-3.29972100	1.47786600	-2.80803400
H	-2.83512800	0.48711000	-2.90549500
C	-4.17325100	1.69321700	-4.04468200
H	-4.63965800	2.69153100	-4.03984400
H	-4.98329600	0.95078500	-4.10879000
H	-3.56586300	1.61465500	-4.95975700
C	-2.16930500	2.50458300	-2.75417300
H	-2.56119700	3.53182000	-2.82812400
H	-1.47763000	2.35143900	-3.59663900
H	-1.58644300	2.45277100	-1.82308900
C	-5.11583500	-0.86080000	1.36596400
H	-4.28878500	-1.53336200	1.09312900
C	-6.41974500	-1.63329000	1.15858400
H	-7.29168000	-1.00069300	1.39068400
H	-6.45694700	-2.51495400	1.81785700
H	-6.52889400	-1.98569500	0.12359700
C	-4.94001000	-0.49104800	2.83735100
H	-4.00440600	0.06007800	3.00509100
H	-4.92315100	-1.39971300	3.45859900
H	-5.76859800	0.13372700	3.20514900
C	4.06097400	-0.11469500	-0.32070600
C	5.40405800	-0.42121800	-0.86610500
C	6.43643300	0.49462000	-0.64102700
H	6.23859000	1.40565600	-0.07199600
C	7.71135000	0.24099400	-1.14234400
H	8.51569800	0.95733300	-0.96189500
C	7.95535900	-0.91863300	-1.87553700
H	8.95383400	-1.11332500	-2.27341300
C	6.92328300	-1.82870400	-2.10493000
H	7.10971800	-2.73458400	-2.68569900
C	5.65003400	-1.58570900	-1.59927900
H	4.83699500	-2.28776400	-1.78846500
C	3.12677600	1.41615700	-2.15250600
C	3.94325700	2.70908100	-2.12788700
H	3.56647900	3.38471600	-1.34711300
H	3.85481300	3.21850800	-3.09906500
H	5.00948300	2.50065000	-1.95236000
C	3.64955700	0.48567100	-3.24853400
H	4.73698000	0.34300200	-3.20681800
H	3.41230700	0.93470100	-4.22349100
H	3.15253300	-0.49448400	-3.20282500
C	1.65297500	1.70571800	-2.43794100
H	1.21659500	2.36808000	-1.67772000
H	1.07219500	0.77459500	-2.49753900
H	1.56851800	2.21850200	-3.40728700
C	4.11142800	-1.43803000	1.87986000
C	5.24332600	-0.71098600	2.60893800
H	4.90238900	0.25602300	3.00549200
H	6.09426600	-0.52942200	1.93611500
H	5.60359500	-1.31851800	3.45256100
C	2.95108500	-1.73673600	2.82905500
H	3.28428200	-2.40382500	3.63659500
H	2.12916900	-2.22928700	2.29088700
H	2.56488000	-0.81880000	3.29185600
C	4.62482100	-2.76220400	1.31379300
H	4.89042200	-3.42989800	2.14651000
H	5.52072900	-2.62982500	0.69433900
H	3.84205400	-3.25460500	0.72170000
C	-0.58737100	0.33286900	0.44411200
H	-0.58803800	1.30387600	-0.07716800
C	-1.13033300	0.57277100	1.84981400
H	-0.27038000	0.82480100	2.48695900
H	-1.58204800	-0.33606400	2.27779100
C	-2.09374300	1.74658700	1.89271700
H	-1.57556900	2.64561700	1.53040400
H	-2.97630400	1.58804600	1.25812800
H	-2.44984900	1.92897300	2.91825800

C	2.19838100	2.07413500	1.95048400
O	1.00890700	2.72350900	1.66531400
H	3.08485900	2.69601800	1.70019100
C	2.27397300	1.74273000	3.43795200
H	1.47185200	1.03459000	3.70876400
H	3.22984800	1.24088600	3.65970000
C	2.13717000	3.00272800	4.27896100
H	2.17625500	2.77091000	5.35422100
H	2.94157800	3.71867400	4.04865000
H	1.18582700	3.50287900	4.05420600
H	1.99046600	3.27826100	-0.20104000
C	-0.57376300	5.16616200	-0.17192500
C	0.24602000	5.81580800	0.99812200
O	1.43175200	5.05808500	0.98514200
O	-0.22769600	3.80230700	-0.05798800
C	0.58863100	7.28335600	0.79085400
H	-0.31858700	7.89897600	0.68228000
H	1.15187600	7.65589200	1.66007800
H	1.21917500	7.41856600	-0.09779100
C	-0.45277700	5.63966100	2.35013100
H	-0.75480800	4.59311900	2.49658700
H	0.26484600	5.89657900	3.14376800
H	-1.33673700	6.28742100	2.45764200
C	-0.10962400	5.67316400	-1.53875700
H	-0.54128700	5.02885300	-2.31907800
H	-0.42177700	6.71074200	-1.73339500
H	0.98602100	5.61327600	-1.61340500
C	-2.08153000	5.32484000	-0.04611100
H	-2.36764600	6.38841700	-0.02458300
H	-2.58178700	4.85424300	-0.90479700
H	-2.46022700	4.83927000	0.86267800
B	1.09380000	3.71647700	0.55408800

Cartesian coordinates of the optimized geometry of TS-**7** at PBE0-D3BJ/def2-SVP level of theory:

Ga	2.32547900	-0.25885600	-0.49209800
Cl	1.42334300	0.29871800	-2.45940400
Si	-1.58454400	0.67713400	0.50075100
O	0.06598000	0.68443700	0.86341100
N	3.87501100	0.94483800	-0.34268100
N	3.38513700	-1.85284400	-0.97769100
N	-2.17622600	1.55489600	-1.01254900
N	-1.58398900	2.65673300	0.75043500
C	4.92365100	0.74572700	-1.13760400
C	5.16124800	-0.45559600	-1.82623700
H	6.04208500	-0.46291000	-2.46666400
C	4.50682800	-1.68821100	-1.67963000
C	5.92072700	1.85008700	-1.34882800
H	6.91632500	1.44621300	-1.57045700
H	5.98009800	2.52694700	-0.48763300
H	5.60033700	2.44967600	-2.21581900
C	5.12486000	-2.87249100	-2.36786600
H	5.10025200	-3.77027100	-1.73595300
H	6.15954600	-2.66213900	-2.66262200
H	4.54768300	-3.10821400	-3.27523300
C	3.77095400	2.11721900	0.46841800
C	3.22474100	3.30335300	-0.06019400
C	3.08784400	4.40597200	0.79021500
H	2.65952000	5.33138400	0.39740800
C	3.48078000	4.34432000	2.11988000
H	3.35758100	5.21385700	2.76958600
C	4.03155500	3.17021600	2.62427600
H	4.34219700	3.13155800	3.66955100
C	4.19035900	2.04146100	1.81707500
C	2.78750300	3.41939800	-1.50624600
H	3.01369700	2.46774300	-2.00683400
C	3.53027900	4.53689700	-2.23926200

H	3.27395500	5.52697900	-1.82966300
H	3.25754600	4.54152900	-3.30605000
H	4.62226900	4.42595200	-2.16651000
C	1.27810800	3.61997000	-1.60991100
H	0.72892600	2.86337000	-1.03235600
H	0.95467400	3.54225200	-2.65918200
H	0.99421100	4.61676600	-1.23674500
C	4.83639700	0.78086300	2.36346500
H	4.36218200	-0.06573700	1.84360000
C	6.33042200	0.72592500	2.03497900
H	6.85560600	1.59505300	2.46207700
H	6.51784100	0.71423400	0.95363800
H	6.78297600	-0.18503300	2.45742600
C	4.62749000	0.58550000	3.86159600
H	3.56988500	0.67646100	4.14711200
H	5.20363100	1.31471100	4.45260300
H	4.97557700	-0.41428800	4.16235000
C	2.89422400	-3.16916200	-0.71979600
C	2.09397400	-3.84433800	-1.66565100
C	1.66804000	-5.14308600	-1.36524700
H	1.05093200	-5.68015900	-2.08945900
C	2.01071600	-5.76012300	-0.16972000
H	1.67349700	-6.77818100	0.03930900
C	2.77899400	-5.07369100	0.76439800
H	3.03787900	-5.55907700	1.70712500
C	3.23233700	-3.77680200	0.51155300
C	1.66482500	-3.21565500	-2.97704100
H	2.18463100	-2.25201800	-3.07984600
C	2.02001200	-4.08495900	-4.18336400
H	1.44132100	-5.02201000	-4.19585600
H	3.08589600	-4.35867200	-4.19828200
H	1.79316200	-3.54886400	-5.11751900
C	0.16877800	-2.90513100	-2.95959600
H	-0.43137100	-3.82279500	-2.84585500
H	-0.13608500	-2.41054700	-3.89386800
H	-0.08146400	-2.22015600	-2.13886700
C	4.10943100	-3.06132400	1.52079200
H	3.94859800	-1.98338900	1.36665500
C	5.59534300	-3.32269500	1.26674500
H	5.81906400	-4.39990800	1.32458800
H	6.21080700	-2.80708300	2.02070800
H	5.91315700	-2.96142500	0.27927900
C	3.74798800	-3.38704700	2.96689000
H	2.66961200	-3.27744400	3.15454000
H	4.28123900	-2.71089900	3.65197500
H	4.03567500	-4.41418400	3.24042900
C	-2.15838900	2.76943900	-0.42279700
C	-2.79829100	4.00312700	-0.95442300
C	-4.09268900	4.31578400	-0.52511100
H	-4.62008500	3.62671300	0.13824400
C	-4.71090100	5.48655400	-0.95674100
H	-5.72320800	5.71984100	-0.61920500
C	-4.04115100	6.35381500	-1.81877900
H	-4.52548500	7.27314500	-2.15603900
C	-2.75294200	6.04263700	-2.25145800
H	-2.22327400	6.71797700	-2.92721100
C	-2.13277000	4.87155700	-1.82313900
H	-1.12052900	4.63536100	-2.15113700
C	-2.77980600	1.17490700	-2.30432200
C	-4.28744200	1.44513600	-2.31025700
H	-4.74364700	0.97682600	-1.42525400
H	-4.73784600	0.99935900	-3.21032400
H	-4.51826100	2.51865100	-2.32155200
C	-2.07170100	1.90719100	-3.44750700
H	-2.25229400	2.98933900	-3.41889600
H	-2.44824200	1.53632500	-4.41274300
H	-0.98840100	1.72022600	-3.40113000
C	-2.57355500	-0.32381500	-2.49835200
H	-3.19960200	-0.89221300	-1.79772800

H	-1.51376300	-0.58717800	-2.38148500
H	-2.88440500	-0.59824800	-3.51714700
C	-1.32776200	3.63767400	1.80783100
C	-2.56767800	3.81204000	2.69339700
H	-2.87138800	2.85967800	3.14917400
H	-3.41520800	4.20787300	2.11595900
H	-2.35183800	4.52248100	3.50605600
C	-0.17762100	3.08920500	2.65531900
H	0.04258900	3.78459200	3.47895300
H	0.72712500	2.95603500	2.04780200
H	-0.43488100	2.11195900	3.08531200
C	-0.90596700	5.00586800	1.26517700
H	-0.55961700	5.62995100	2.10257500
H	-1.72885600	5.53785100	0.77142700
H	-0.07535300	4.90065600	0.55498500
C	0.96974800	-0.39180900	0.96096400
H	0.44716000	-1.34433500	0.76600900
C	1.54916100	-0.42930300	2.36775400
H	1.96000500	0.56886500	2.58604400
H	2.39990200	-1.12579600	2.39234600
C	0.55131000	-0.82047100	3.44509400
H	-0.32714700	-0.16127800	3.43371300
H	0.18797800	-1.85115200	3.30300000
H	0.99883500	-0.76289200	4.44951300
C	-3.21600800	0.46360000	1.52752800
O	-4.10115400	-0.25397800	0.70888500
H	-3.61389200	1.49497000	1.54566500
C	-3.11646400	0.02796900	2.98599900
H	-2.72998100	-1.00008100	3.05327400
H	-2.35615300	0.67010700	3.46651000
C	-4.43323200	0.14206500	3.74161900
H	-4.31029900	-0.14106700	4.79888400
H	-4.81418900	1.17695800	3.71312400
H	-5.19084700	-0.51650900	3.29418900
H	-1.52954600	-0.86051100	0.03783800
C	-6.09497300	-2.63569900	-0.50032100
C	-6.22754800	-3.04273700	1.00952500
O	-5.43162700	-2.06535700	1.64990900
O	-4.76494900	-2.15625200	-0.56036900
C	-7.64084900	-2.97114200	1.56353400
H	-8.32060500	-3.64025500	1.01314200
H	-7.63972400	-3.27879900	2.62008000
H	-8.03178100	-1.94674800	1.51299400
C	-5.63638100	-4.42603900	1.28629300
H	-4.64106200	-4.52471500	0.83105200
H	-5.52429000	-4.54540500	2.37391000
H	-6.27726200	-5.23690300	0.90859900
C	-7.03025700	-1.48433200	-0.86780900
H	-6.73219300	-1.09298000	-1.85173900
H	-8.08363100	-1.79827800	-0.92072300
H	-6.93547200	-0.66778500	-0.13761800
C	-6.26239900	-3.77830100	-1.48720400
H	-7.25961900	-4.23716900	-1.40137600
H	-6.14893200	-3.39905200	-2.51407600
H	-5.50214300	-4.55495300	-1.32993700
B	-4.42419200	-1.63727800	0.73500100
C	-2.17321500	-2.52599600	0.28625200
H	-2.46246300	-2.40921900	-0.76958700
O	-3.04969100	-2.38339600	1.16654000
C	-0.98708200	-3.37088600	0.61537900
H	-0.16978700	-3.19788100	-0.09620600
H	-0.63602200	-3.12203300	1.62653000
C	-1.42688000	-4.83555800	0.56058800
H	-0.57636500	-5.48907600	0.79599400
H	-2.23375300	-5.02663000	1.28235900
H	-1.79354600	-5.10647600	-0.44173500

Cartesian coordinates of the optimized geometry of TS-**8** at PBE0-D3BJ/def2-SVP level of theory:

Ga	-1.80423300	-0.84023900	-0.74916800
Cl	-0.85085500	-1.02569300	-2.75815400
Si	2.19453200	-0.10190300	0.69628500
O	0.65434900	-0.57128600	0.53932000
N	-2.57673100	-2.64672500	-0.53401200
N	-3.51312700	0.02963000	-1.15828500
N	3.20116800	-0.44931000	-0.78677700
N	3.17075800	-1.65414500	0.99847900
C	-3.55664400	-2.98444300	-1.37327700
C	-4.33600100	-2.04849800	-2.07126200
H	-5.07495200	-2.46553000	-2.75387400
C	-4.39058500	-0.65448300	-1.89140700
C	-3.83933400	-4.43734700	-1.63255900
H	-4.87212900	-4.59059200	-1.96838100
H	-3.64077300	-5.06240100	-0.75288400
H	-3.16643400	-4.78279000	-2.43455100
C	-5.49985100	0.08781500	-2.57937100
H	-6.00798500	0.77803500	-1.89214800
H	-6.23294600	-0.60344900	-3.01081000
H	-5.07828900	0.70796300	-3.38567900
C	-1.97431300	-3.61275400	0.32858600
C	-0.84360900	-4.34331900	-0.08781600
C	-0.24239800	-5.21932900	0.82320800
H	0.63768700	-5.78819700	0.51223200
C	-0.74586800	-5.38520700	2.10595200
H	-0.26151200	-6.07302700	2.80312900
C	-1.87580000	-4.67442000	2.49792300
H	-2.27161500	-4.81218800	3.50556600
C	-2.50750600	-3.78202100	1.62886400
C	-0.26642800	-4.21768100	-1.48216700
H	-0.91084100	-3.53872600	-2.05705400
C	-0.22990500	-5.56263000	-2.20722400
H	0.46738800	-6.26735600	-1.72618700
H	0.10364800	-5.42653600	-3.24774400
H	-1.21975800	-6.04283000	-2.22718700
C	1.11805600	-3.57634100	-1.44473200
H	1.09474100	-2.60681800	-0.92906600
H	1.48837500	-3.40552100	-2.46735100
H	1.83382800	-4.22988500	-0.91966100
C	-3.75003400	-3.03104200	2.06954000
H	-3.76512600	-2.09000700	1.49975500
C	-5.02624800	-3.79467200	1.70830500
H	-5.04057400	-4.78346900	2.19426200
H	-5.12429000	-3.94837400	0.62588500
H	-5.91497300	-3.23801400	2.04442400
C	-3.75216900	-2.68068700	3.55414000
H	-2.83024000	-2.16313200	3.85600200
H	-3.86401600	-3.57495100	4.18703500
H	-4.60309100	-2.02199400	3.78328700
C	-3.75858900	1.40961300	-0.86504100
C	-3.34224300	2.41649000	-1.75830500
C	-3.64189000	3.74557700	-1.44187000
H	-3.31513100	4.53800700	-2.11870300
C	-4.32987600	4.07565500	-0.28233900
H	-4.55349100	5.12055000	-0.05523900
C	-4.70740800	3.07331100	0.60435700
H	-5.22264500	3.34047700	1.52876700
C	-4.42131800	1.73203100	0.33987000
C	-2.53373700	2.11057600	-3.00086300
H	-2.54656200	1.02228000	-3.15803100
C	-3.10401200	2.76716300	-4.25653300
H	-3.03649000	3.86553500	-4.21200200
H	-4.16217800	2.50635900	-4.41680800
H	-2.53697000	2.44259200	-5.14245400
C	-1.07803700	2.51743800	-2.77678600
H	-0.97744900	3.60307800	-2.63275900
H	-0.45484000	2.21923700	-3.63341700

H	-0.65118800	2.05292200	-1.87837700
C	-4.82145500	0.65442900	1.32844600
H	-4.14121000	-0.19278300	1.15306900
C	-6.23967800	0.13898800	1.08064000
H	-6.97278100	0.95792000	1.15694100
H	-6.50882100	-0.62658900	1.82579400
H	-6.34232200	-0.31775200	0.08633100
C	-4.64956500	1.09563000	2.77924100
H	-3.65519500	1.53152200	2.95464800
H	-4.77287400	0.23768300	3.45668300
H	-5.39964000	1.84702800	3.07101600
C	3.79946000	-1.46785900	-0.16172300
C	4.99521300	-2.20834500	-0.62922000
C	6.26261100	-1.73835500	-0.27407000
H	6.35731100	-0.82953500	0.32427300
C	7.40049100	-2.42750000	-0.68943500
H	8.38926300	-2.05606800	-0.41174500
C	7.27536600	-3.58304000	-1.45790000
H	8.16800400	-4.12168900	-1.78381300
C	6.00953200	-4.04971200	-1.81348000
H	5.90826600	-4.95293800	-2.41903100
C	4.86984800	-3.36644600	-1.40196600
H	3.87846400	-3.72436200	-1.68174200
C	3.50846700	0.23055700	-2.06141000
C	4.67396400	1.19250800	-1.83197100
H	4.42275100	1.88635500	-1.01863400
H	4.86368400	1.78440800	-2.73942100
H	5.59676600	0.64688300	-1.58223100
C	3.84470600	-0.77629500	-3.16139300
H	4.81082300	-1.27416900	-3.00914600
H	3.89630400	-0.23886800	-4.11915500
H	3.05456300	-1.53732500	-3.24610600
C	2.24951100	0.99167000	-2.46881100
H	1.97920100	1.75665700	-1.72869000
H	1.40579300	0.29959000	-2.59985000
H	2.42740200	1.49899700	-3.42841200
C	3.43178100	-2.54258900	2.13452800
C	4.55414500	-1.95591400	2.99531000
H	4.30690000	-0.93157400	3.31280800
H	5.50285600	-1.92904200	2.43932000
H	4.70702300	-2.56705400	3.89759600
C	2.13101700	-2.59495400	2.93420700
H	2.24819600	-3.25332600	3.80672500
H	1.30365100	-2.97284800	2.31622400
H	1.85635600	-1.59692800	3.30452200
C	3.79077800	-3.96079500	1.69155100
H	3.82369600	-4.61302900	2.57649500
H	4.77089500	-4.01350300	1.20119700
H	3.02830400	-4.35719500	1.00572900
C	-0.68746400	-0.10590100	0.72048200
H	-0.67268500	0.99382400	0.66479100
C	-1.21229800	-0.53777200	2.08075600
H	-1.14235600	-1.63461600	2.16281900
H	-2.28781300	-0.29964200	2.10503300
C	-0.54153900	0.11534000	3.27628000
H	0.52552600	-0.13788700	3.32991500
H	-0.60588900	1.20908000	3.21624000
H	-1.00198600	-0.21675200	4.21994100
C	2.85638800	1.40909800	1.53352500
O	2.34923000	2.19279600	0.46883400
H	3.96226800	1.33677800	1.42138500
C	2.56961100	1.80616800	2.97322500
H	1.51420000	2.10103500	3.04466200
H	2.69543300	0.90614600	3.60277200
C	3.46919400	2.91971400	3.48914800
H	3.23383200	3.15810100	4.53820500
H	4.53217300	2.62812000	3.44229600
H	3.31857400	3.82509200	2.88499600
H	-1.77446900	2.94942200	0.65466000

C	1.89438700	4.95869100	-1.02747900
C	2.11688100	5.58268300	0.40779900
O	1.96774700	4.48195700	1.26927800
O	1.10546500	3.82449800	-0.76621700
C	3.49829800	6.19587300	0.61703400
H	3.69251600	7.01626600	-0.09184000
H	3.56285500	6.60603700	1.63651300
H	4.28893600	5.44214500	0.50998400
C	1.06968900	6.64111400	0.76015300
H	0.05000900	6.26585700	0.60043200
H	1.17112400	6.89149900	1.82637300
H	1.19753100	7.56372200	0.17390500
C	3.21033500	4.52478700	-1.67369300
H	2.98408500	3.93964500	-2.57690100
H	3.83636100	5.38197500	-1.96352200
H	3.77914700	3.88382500	-0.98917800
C	1.14567500	5.86104800	-1.99973600
H	1.68223900	6.80730000	-2.17102700
H	1.04269500	5.34740600	-2.96784700
H	0.13627400	6.08896800	-1.63260800
B	1.36624000	3.34710600	0.57518000
C	-1.00463200	3.52808200	1.20174500
H	-0.87971500	4.46345200	0.62958200
O	0.18743700	2.80212300	1.23098700
C	-1.53708000	3.83164300	2.59546900
H	-2.52487900	4.31280400	2.48504900
H	-1.73027900	2.87398300	3.10950200
C	-0.61761200	4.68995900	3.44754800
H	-0.97417800	4.75688000	4.48754500
H	0.40426700	4.28352700	3.44724400
H	-0.54742400	5.71291900	3.04924800

Cartesian coordinates of the optimized geometry of TS-**9** at PBE0-D3BJ/def2-SVP level of theory:

Ga	-1.71233600	-0.91714000	-0.71656000
Cl	-0.75580000	-1.25902400	-2.71355800
Si	2.17614400	0.08988100	0.57759300
O	0.68101500	-0.62388000	0.71403200
N	-2.44280400	-2.72191400	-0.33492300
N	-3.45457800	-0.16081100	-1.24286300
N	3.14971900	-0.37150100	-0.90440300
N	3.15989400	-1.54130400	0.89841700
C	-3.39326900	-3.17554900	-1.14940300
C	-4.16397100	-2.34603300	-1.98307600
H	-4.85953200	-2.85890100	-2.64564800
C	-4.26523200	-0.94752600	-1.95558000
C	-3.66361600	-4.65167100	-1.23211500
H	-4.69326300	-4.84845300	-1.55484300
H	-3.47040200	-5.16346200	-0.28121600
H	-2.98408800	-5.08999900	-1.98083300
C	-5.34614800	-0.31695300	-2.78710200
H	-5.94447400	0.39595200	-2.20372000
H	-6.00747500	-1.07627300	-3.22017500
H	-4.88477600	0.26019100	-3.60364300
C	-1.81386000	-3.59303600	0.60868500
C	-0.70178400	-4.36990500	0.22819100
C	-0.08022300	-5.16428300	1.19755800
H	0.78790900	-5.76549400	0.91594700
C	-0.54445300	-5.20323700	2.50472200
H	-0.04187200	-5.82515800	3.24914700
C	-1.65620100	-4.44776400	2.86220100
H	-2.02136400	-4.48787600	3.88957800
C	-2.31112700	-3.63664900	1.93230100
C	-0.15888700	-4.36909500	-1.18529700
H	-0.82485200	-3.75567100	-1.80747500
C	-0.10956800	-5.77422200	-1.78461400
H	0.61578900	-6.41588500	-1.25920200

H	0.19786400	-5.72730600	-2.84091500
H	-1.08705500	-6.27731300	-1.73618400
C	1.21442600	-3.70733100	-1.23259000
H	1.19170100	-2.70402600	-0.78586200
H	1.55734800	-3.61284600	-2.27426500
H	1.95134300	-4.30946600	-0.67759500
C	-3.54795500	-2.85381300	2.33369200
H	-3.55918000	-1.94596700	1.71260800
C	-4.83090500	-3.62752700	2.02148400
H	-4.84357700	-4.59395200	2.55037300
H	-4.94357800	-3.82661000	0.94772100
H	-5.71342100	-3.05307600	2.34419800
C	-3.54029600	-2.41832800	3.79556000
H	-2.60998500	-1.89701200	4.06398700
H	-3.66173200	-3.27342300	4.47887000
H	-4.37972200	-1.73448300	3.98947400
C	-3.81723600	1.21013300	-1.05950000
C	-3.38788300	2.19842700	-1.96722400
C	-3.82863500	3.51222700	-1.77624500
H	-3.49761300	4.28871900	-2.46955900
C	-4.67660100	3.84542300	-0.72919700
H	-5.01848100	4.87568300	-0.60511900
C	-5.07040700	2.86525000	0.17530900
H	-5.71857500	3.13503100	1.01158100
C	-4.63989800	1.54354900	0.04060700
C	-2.44037400	1.89177700	-3.10665000
H	-2.34982800	0.79919900	-3.19127700
C	-2.94114100	2.41823800	-4.45029300
H	-2.96382000	3.51910800	-4.47942500
H	-3.95669100	2.05960500	-4.67997400
H	-2.27324800	2.08419700	-5.25902300
C	-1.04916000	2.43606900	-2.78582800
H	-1.05577700	3.53070100	-2.66922500
H	-0.34000300	2.17782400	-3.58585100
H	-0.65455200	2.02177700	-1.84815700
C	-5.06639600	0.49882600	1.05387400
H	-4.34196700	-0.32538400	0.97494300
C	-6.44401400	-0.08489500	0.73636700
H	-7.21047200	0.70657300	0.71576900
H	-6.73757500	-0.82000400	1.50216600
H	-6.45859400	-0.59785400	-0.23519000
C	-5.02127500	1.02537000	2.48617000
H	-4.05248100	1.49240300	2.71739400
H	-5.18312200	0.20342600	3.19929100
H	-5.80689200	1.77366700	2.67375300
C	3.75495200	-1.39076100	-0.26462700
C	4.96224700	-2.11608300	-0.73818000
C	6.21943800	-1.56750500	-0.46549600
H	6.28830100	-0.61408900	0.06308400
C	7.37581700	-2.23466600	-0.86474800
H	8.35411400	-1.80072300	-0.64615500
C	7.28286100	-3.44949000	-1.54115900
H	8.18943400	-3.97196400	-1.85504900
C	6.02969200	-3.99601100	-1.81742300
H	5.95149200	-4.94696200	-2.34910200
C	4.87227300	-3.33462900	-1.41713400
H	3.89164400	-3.76054900	-1.63045500
C	3.34810000	0.15683200	-2.26116100
C	4.63068700	0.99114500	-2.31535600
H	4.61913900	1.76851900	-1.53881300
H	4.72378500	1.48669000	-3.29363300
H	5.52128300	0.36284300	-2.16933900
C	3.40224500	-0.96584900	-3.29944900
H	4.30765400	-1.58036400	-3.21296400
H	3.39636100	-0.52263600	-4.30644000
H	2.51448900	-1.60857100	-3.20897000
C	2.12849900	1.02055100	-2.56836300
H	1.98082800	1.79352100	-1.80575700
H	1.22700400	0.39439600	-2.60993500

H	2.25317900	1.50803400	-3.54661000
C	3.50312900	-2.31597000	2.08866500
C	4.63760000	-1.61851600	2.84781800
H	4.36505000	-0.57824200	3.07717400
H	5.56358000	-1.61430800	2.25386400
H	4.84444800	-2.14038700	3.79476600
C	2.24225100	-2.34149900	2.95397200
H	2.42268800	-2.91971200	3.87200600
H	1.40103600	-2.78930200	2.40664300
H	1.94923300	-1.32227300	3.24328900
C	3.90229900	-3.75507100	1.76003500
H	4.01578700	-4.32036500	2.69705600
H	4.85463900	-3.81261800	1.21741600
H	3.12266600	-4.24543300	1.15990500
C	-0.60492300	-0.02031300	0.67425200
H	-0.50100700	1.05040500	0.43231800
C	-1.29826200	-0.15611500	2.02290300
H	-1.39511400	-1.22756500	2.25747300
H	-2.32475100	0.23128100	1.90456300
C	-0.62343900	0.55321700	3.18386300
H	0.35044800	0.09689100	3.40606500
H	-0.45019600	1.61345200	2.95417800
H	-1.23505400	0.48087300	4.09737100
C	2.89399000	1.48282400	1.51591400
O	1.85365300	1.81459500	0.51610500
H	3.87279800	1.86856400	1.17758300
C	2.62266300	1.87244500	2.96147000
H	1.74597500	2.53461300	2.98049900
H	2.35040900	0.98935500	3.56497900
C	3.80429500	2.57505900	3.60877700
H	3.60096400	2.82182000	4.66234500
H	4.71269600	1.94934400	3.58632500
H	4.02320100	3.51200100	3.07552500
H	-1.94816900	2.71129300	0.56226600
C	2.02755200	5.09373400	-0.81240500
C	2.47922300	5.45265500	0.64999800
O	2.03524700	4.32678500	1.39601900
O	0.88006100	4.27513100	-0.58603900
C	3.97856600	5.61405800	0.83080000
H	4.37149800	6.42071200	0.19308800
H	4.19647100	5.87185700	1.87784700
H	4.51125900	4.68406800	0.59488000
C	1.75854600	6.68215900	1.19860300
H	0.67211300	6.59909700	1.05338000
H	1.94737000	6.74442000	2.28008100
H	2.10690400	7.61327300	0.72790900
C	3.07502200	4.27099200	-1.54664700
H	2.64623000	3.89837300	-2.48660100
H	3.96903800	4.86513200	-1.78500900
H	3.36460600	3.40439900	-0.93901200
C	1.61281700	6.28274400	-1.66141700
H	2.44684600	6.99023400	-1.78597100
H	1.31081300	5.93210500	-2.65948000
H	0.76105600	6.81509700	-1.21900900
B	0.96150800	3.76490700	0.70265200
C	-1.45888000	3.61911100	0.95819200
H	-1.44215200	4.35324000	0.13409900
O	-0.15106900	3.31641400	1.37237600
C	-2.25429700	4.15440100	2.13526000
H	-3.29391500	4.30578300	1.80385600
H	-2.28639700	3.37461700	2.91583200
C	-1.68073000	5.43682500	2.71282700
H	-2.25280300	5.78142000	3.58779100
H	-0.63615400	5.28784100	3.02442100
H	-1.69297700	6.24808500	1.96604400

Cartesian coordinates of the optimized geometry of TS-**10** at PBE0-D3BJ/def2-SVP level of theory:

Ga	1.91536800	-0.62521300	0.57589800
Cl	1.20051600	-0.81825000	2.67964600
Si	-2.16238700	0.55306000	-0.33873100
O	-0.66057000	-0.16632800	-0.32409400
N	2.61043400	-2.42558500	0.20444800
N	3.68870200	0.18934100	0.87909000
N	-2.98847000	0.13002000	1.24288100
N	-3.26624100	-0.89419700	-0.62744700
C	3.68169400	-2.83613000	0.88635100
C	4.59564900	-1.96232200	1.48952600
H	5.42815500	-2.43401700	2.01029100
C	4.66341400	-0.56021500	1.38343900
C	3.92573500	-4.30969500	1.04792000
H	3.28044500	-4.67918100	1.86172900
H	4.96844200	-4.51755800	1.31629000
H	3.65676600	-4.87554400	0.14692500
C	5.91999200	0.08680900	1.89458200
H	6.11563400	1.05111900	1.40926100
H	6.78251100	-0.57708300	1.75605200
H	5.81366900	0.27669300	2.97413400
C	1.89212700	-3.32937000	-0.63623700
C	0.77733800	-4.03626800	-0.14502100
C	0.07320700	-4.86062200	-1.02992600
H	-0.79607700	-5.41290300	-0.66451400
C	0.46041400	-4.99281300	-2.35666800
H	-0.10478600	-5.63976400	-3.03170100
C	1.57214400	-4.29848400	-2.82430200
H	1.87073300	-4.40717400	-3.86862700
C	2.30421600	-3.45730400	-1.98385800
C	0.32844400	-3.93426300	1.29847100
H	1.05760300	-3.30998100	1.83336900
C	0.29301500	-5.30125000	1.98113700
H	-0.47484300	-5.95723700	1.54064400
H	0.05446000	-5.18709600	3.04998600
H	1.25800200	-5.82478300	1.90126900
C	-1.02143600	-3.22726500	1.41134700
H	-1.01039000	-2.25223900	0.90388600
H	-1.27222900	-3.05303500	2.46908100
H	-1.82106300	-3.83805900	0.96237100
C	3.51700700	-2.70936800	-2.50952600
H	3.62449200	-1.80800900	-1.88622300
C	4.80379300	-3.52135300	-2.34397400
H	4.73466100	-4.47738100	-2.88738200
H	5.02018100	-3.74549000	-1.29150500
H	5.66413100	-2.96360400	-2.74641200
C	3.36756800	-2.26196100	-3.96122800
H	2.42298700	-1.72615700	-4.13222100
H	3.40414700	-3.11396500	-4.65798500
H	4.19617800	-1.59104300	-4.23564900
C	3.84063300	1.59971500	0.71745800
C	3.67271600	2.47623100	1.81020600
C	3.81239700	3.84944700	1.57897500
H	3.68581000	4.54222500	2.41444300
C	4.09847400	4.34815800	0.31559100
H	4.19954100	5.42483700	0.15981700
C	4.24943000	3.47179300	-0.75425300
H	4.46897300	3.86845200	-1.74700200
C	4.12428700	2.09266100	-0.57666700
C	3.34888400	1.99624700	3.21253200
H	3.33908600	0.89671500	3.20292700
C	4.39322600	2.46339600	4.22808600
H	4.36677700	3.55672800	4.35985200
H	5.41649000	2.19571300	3.92586500
H	4.19756400	2.01087900	5.21248300
C	1.95110200	2.44048700	3.64184100
H	1.87642600	3.53891500	3.68678000
H	1.71433300	2.04138200	4.63991800

H	1.18528600	2.06795400	2.95032400
C	4.33873300	1.13869700	-1.73456500
H	3.72279600	0.25090400	-1.52551700
C	5.78952900	0.65846400	-1.80008300
H	6.47495000	1.50647000	-1.95734500
H	5.92553200	-0.04849100	-2.63396100
H	6.09280400	0.14534500	-0.87690800
C	3.89148100	1.70351900	-3.07786000
H	2.87025800	2.10741400	-3.02870400
H	3.90469300	0.91424800	-3.84434900
H	4.55885100	2.50622200	-3.42885900
C	-3.75162800	-0.75989000	0.60491500
C	-4.96602800	-1.41284100	1.14159200
C	-6.18760000	-0.74049000	1.02194500
H	-6.19648800	0.22251200	0.50275600
C	-7.34165400	-1.32803500	1.53340800
H	-8.29809800	-0.80829800	1.44063400
C	-7.28160100	-2.57563000	2.15536900
H	-8.19079000	-3.03148900	2.55473200
C	-6.06240500	-3.24298200	2.26628900
H	-6.01293900	-4.21919400	2.75392100
C	-4.90094400	-2.66326600	1.76034800
H	-3.93932500	-3.17052600	1.85973500
C	-3.12659500	0.78238400	2.54937400
C	-4.18137500	1.88899100	2.47847200
H	-3.89992500	2.63410700	1.72155000
H	-4.25582500	2.39404400	3.45411300
H	-5.16934100	1.48370000	2.22191900
C	-3.47135200	-0.23132900	3.64024200
H	-4.49183600	-0.62400900	3.54243700
H	-3.39377800	0.25967500	4.62153600
H	-2.76293100	-1.07338400	3.62339500
C	-1.76043300	1.39490200	2.85153000
H	-1.50034500	2.13068000	2.07722100
H	-0.98177400	0.62074900	2.89228400
H	-1.78804500	1.92017700	3.81780300
C	-3.72429500	-1.69889100	-1.76471900
C	-5.21200300	-1.48905700	-2.05667600
H	-5.43726400	-0.41356600	-2.01954500
H	-5.84659400	-2.00682900	-1.32465500
H	-5.45175200	-1.89363900	-3.05181900
C	-2.89972500	-1.22770100	-2.95853000
H	-3.18076400	-1.78610800	-3.86264000
H	-1.82843000	-1.38856800	-2.77350600
H	-3.07628100	-0.15838600	-3.14853100
C	-3.42323300	-3.17635000	-1.50693300
H	-3.72305100	-3.77610600	-2.37997700
H	-3.98215000	-3.54889800	-0.63647200
H	-2.34883700	-3.32901100	-1.33190000
C	0.64761500	0.18793900	-0.73768000
H	0.77125200	1.28543700	-0.71443600
C	0.90851700	-0.30344400	-2.15802800
H	0.59339800	-1.35857200	-2.21005300
H	1.99523600	-0.31318100	-2.33092100
C	0.25355400	0.50000500	-3.27001400
H	-0.82962000	0.60139100	-3.12774300
H	0.64807900	1.52396600	-3.31159100
H	0.42061000	0.01977600	-4.24755700
C	-0.65266600	4.23625600	-1.57804000
C	-0.91528900	4.39092600	-0.03776600
B	-2.04689700	2.56655900	-0.90108800
O	-1.02124500	2.87962400	-1.81542700
O	-1.96619700	3.45554400	0.20127900
H	-3.28113000	2.76143000	-1.46826200
C	-4.60326200	2.59164800	-1.20942600
O	-4.79129300	1.38813700	-0.86144600
H	-4.64994800	3.39023700	-0.42525800
C	-5.12472700	3.09231800	-2.55360700
H	-6.22557300	3.14302700	-2.46248200

H	-4.77766000	4.12824000	-2.70941200
C	-4.73491500	2.21168300	-3.72545500
H	-5.10868100	1.18732900	-3.58732500
H	-5.13641700	2.59585800	-4.67536300
H	-3.63740600	2.15966100	-3.81948500
C	-1.38844300	5.77270700	0.38360400
H	-0.63642800	6.53963500	0.14156200
H	-2.33544200	6.03888100	-0.10265900
H	-1.55317600	5.78722500	1.47094200
C	-1.56160600	5.12425700	-2.42488700
H	-1.28353000	6.18586400	-2.35584400
H	-1.47935200	4.80899300	-3.47526700
H	-2.61339900	5.01815200	-2.12326800
C	0.79452000	4.42963100	-1.99442300
H	0.88839600	4.28888000	-3.08166600
H	1.14203900	5.44558500	-1.75171500
H	1.45892300	3.71170700	-1.49710300
C	0.28347600	3.98378100	0.80888800
H	1.12061900	4.68875600	0.71178300
H	-0.01847700	3.95467200	1.86456700
H	0.64977300	2.98356200	0.53774300

Cartesian coordinates of the optimized geometry of TS-**11** at PBE0-D3BJ/def2-SVP level of theory:

Ga	-1.86207500	-0.60775200	-0.60597000
Cl	-1.07772000	-0.86534000	-2.68573800
Si	2.06416600	0.54993300	0.36871000
O	0.67674700	-0.41037600	0.58644700
N	-2.68517400	-2.36323600	-0.26415000
N	-3.57498600	0.31507300	-0.94624000
N	3.04922500	-0.00860900	-1.12106100
N	3.21170800	-0.92894900	0.81522700
C	-3.75225400	-2.70166600	-0.98623500
C	-4.55757100	-1.77067800	-1.66239200
H	-5.37525700	-2.19069000	-2.24629100
C	-4.53824700	-0.36876700	-1.56240700
C	-4.12079200	-4.15200400	-1.12127700
H	-3.49477600	-4.60122800	-1.90916800
H	-5.17186200	-4.27145100	-1.41030000
H	-3.92542100	-4.71431300	-0.19923800
C	-5.66971400	0.37755100	-2.21086600
H	-6.01419400	1.21588400	-1.59120300
H	-6.51265200	-0.28916800	-2.42732700
H	-5.31698700	0.81098100	-3.16005400
C	-2.03173900	-3.31747200	0.57431800
C	-1.00583700	-4.13619900	0.06236500
C	-0.36818900	-5.02482400	0.93462500
H	0.43309000	-5.66206900	0.55244300
C	-0.72870100	-5.10701700	2.27242600
H	-0.21480400	-5.80436700	2.93825300
C	-1.74546800	-4.29453000	2.76372300
H	-2.02307500	-4.36283300	3.81692000
C	-2.41325400	-3.39052500	1.93439100
C	-0.56554100	-4.07141000	-1.38585100
H	-1.24308900	-3.38898700	-1.91727800
C	-0.63450000	-5.43499100	-2.07220700
H	0.08829600	-6.14626700	-1.64140500
H	-0.39860000	-5.33489500	-3.14303300
H	-1.63329200	-5.88907300	-1.98437400
C	0.83549800	-3.47250100	-1.49873300
H	0.89849600	-2.49655900	-0.99513900
H	1.10501200	-3.32450200	-2.55585100
H	1.57676800	-4.14639800	-1.03976400
C	-3.53957200	-2.53097900	2.47870800
H	-3.53427400	-1.60114900	1.89054300
C	-4.90672000	-3.18560500	2.26730700
H	-4.95185300	-4.16689500	2.76637000

H	-5.13493700	-3.33460600	1.20387700
H	-5.70403000	-2.55350500	2.68917700
C	-3.36083100	-2.15516200	3.94612100
H	-2.36996800	-1.72049200	4.14082100
H	-3.48958700	-3.02395100	4.61068800
H	-4.12000800	-1.41480700	4.23982400
C	-3.72536900	1.71881000	-0.71811200
C	-3.34820100	2.65418800	-1.70362700
C	-3.53157700	4.01411500	-1.43267100
H	-3.24011400	4.74898800	-2.18685400
C	-4.06625200	4.44688000	-0.22738000
H	-4.19892900	5.51430100	-0.03627200
C	-4.41851900	3.51498800	0.74232300
H	-4.82939400	3.85925400	1.69323400
C	-4.25453500	2.14541000	0.52142500
C	-2.73059200	2.24224500	-3.02434100
H	-2.77539800	1.14609500	-3.09536100
C	-3.47541500	2.82474700	-4.22490200
H	-3.38680300	3.92184800	-4.26891600
H	-4.54912600	2.58306700	-4.20123700
H	-3.05759500	2.42496300	-5.16166200
C	-1.25167500	2.62397500	-3.06451600
H	-1.12071100	3.71769400	-3.02780300
H	-0.77952000	2.25185400	-3.98587700
H	-0.70193400	2.18774900	-2.21972000
C	-4.67381900	1.14651600	1.58236300
H	-4.08032100	0.23681600	1.40614200
C	-6.14366900	0.74740900	1.43940700
H	-6.79941000	1.62840200	1.52849500
H	-6.42736800	0.03209500	2.22763300
H	-6.34717000	0.26956200	0.47125700
C	-4.38305900	1.62933700	3.00064400
H	-3.34662000	1.98200600	3.10722500
H	-4.53841400	0.81113300	3.71964100
H	-5.05272300	2.45096900	3.29906500
C	3.69905400	-0.93807600	-0.41157700
C	4.80642000	-1.79457200	-0.90928900
C	6.12718700	-1.35009900	-0.78995400
H	6.33203200	-0.38610700	-0.31981200
C	7.17503600	-2.13603900	-1.26270800
H	8.20366700	-1.78138400	-1.16671900
C	6.91140300	-3.37079600	-1.85411600
H	7.73387100	-3.98688000	-2.22479200
C	5.59561900	-3.81610700	-1.97272100
H	5.38286000	-4.78055200	-2.43936900
C	4.54481400	-3.03139400	-1.50469500
H	3.51372300	-3.36802800	-1.61601400
C	3.31754000	0.55663600	-2.44943100
C	3.65447400	-0.52622900	-3.47619800
H	4.63880900	-0.98275700	-3.31021000
H	3.66288500	-0.07350100	-4.47879500
H	2.88972100	-1.31747900	-3.46781200
C	2.03179200	1.25559900	-2.88778000
H	1.19731200	0.54106100	-2.92826400
H	2.16882100	1.69144300	-3.88894600
H	1.78036100	2.07073000	-2.19570300
C	4.45274100	1.58018500	-2.34971000
H	5.38796800	1.10328700	-2.01887900
H	4.17635800	2.37553000	-1.64235700
H	4.63861200	2.04086000	-3.33242500
C	3.39753400	-1.84969700	1.93772300
C	4.87226400	-1.97093000	2.33081000
H	5.30620400	-0.97998500	2.53181800
H	5.46862500	-2.45984100	1.54891000
H	4.96182200	-2.57428000	3.24679300
C	2.60692300	-1.26873800	3.10914900
H	2.75039000	-1.88591100	4.00771200
H	1.53733200	-1.24649300	2.86374600
H	2.93166500	-0.24246400	3.33082500

C	2.81931500	-3.22621700	1.60221800
H	2.86603200	-3.88251900	2.48487000
H	3.38143200	-3.71176400	0.79185200
H	1.76800400	-3.12901700	1.29952500
C	-0.60826400	0.17752000	0.71429700
H	-0.54868900	1.25812500	0.47157100
C	-1.10522100	0.03410400	2.14754700
H	-0.98783300	-1.02461300	2.43416900
H	-2.18707800	0.23500900	2.17187500
C	-0.42120800	0.94046900	3.15936400
H	0.66710300	0.78966800	3.17633400
H	-0.57570100	2.00030900	2.91246200
H	-0.80053400	0.75342800	4.17691300
C	0.84216400	4.52883500	1.00964900
C	1.96591000	4.74828900	-0.06352600
B	1.87663900	2.52703000	0.55936100
O	1.16171200	3.24891500	1.53755300
O	2.25866900	3.41374200	-0.46965200
H	4.87833800	2.85529400	1.05231600
C	4.56728100	1.94208600	1.59117800
O	3.21054200	1.69573700	1.30547200
H	5.17316600	1.10753700	1.19247200
C	4.81152400	2.09020100	3.08339600
H	4.55953300	1.13385700	3.57165600
H	5.89374300	2.23968400	3.24585700
C	4.01366800	3.21606500	3.72060100
H	2.94790800	3.13015400	3.45807500
H	4.10766400	3.20546400	4.81693900
H	4.36480300	4.20000800	3.37012500
C	0.83808000	5.54476700	2.13915000
H	1.78121500	5.52612200	2.70016600
H	0.67249600	6.56370500	1.75607200
H	0.02469900	5.30849700	2.84117700
C	-0.54882000	4.43992600	0.38836200
H	-0.56417600	3.72590000	-0.44609100
H	-1.25973500	4.07899100	1.14560700
H	-0.90889100	5.41130400	0.01897600
C	1.53230900	5.54071400	-1.28523200
H	1.20158700	6.55286400	-1.00541200
H	2.37850300	5.63811500	-1.98164900
H	0.71582500	5.03584900	-1.81718800
C	3.23290100	5.36044100	0.52443300
H	3.53905300	4.82756300	1.43362500
H	4.04335100	5.27297400	-0.21443300
H	3.10301800	6.42486200	0.77044000

Cartesian coordinates of the optimized geometry of TS-**12** at PBE0-D3BJ/def2-SVP level of theory:

Ga	-2.08693900	-0.47667500	-0.53839800
Cl	-1.44583200	-0.76610200	-2.66244400
Si	2.03483500	0.29732500	0.26788300
O	0.52639400	-0.47453900	0.33837500
N	-3.00448400	-2.16112100	-0.14105700
N	-3.73369600	0.58068200	-0.78718500
N	2.79715500	-0.41123200	-1.32724200
N	3.14470900	-1.19437500	0.64134400
C	-4.14274500	-2.41904800	-0.78473900
C	-4.93268600	-1.42701600	-1.38626900
H	-5.82844100	-1.78171400	-1.89472600
C	-4.80113700	-0.02803600	-1.29574900
C	-4.60703200	-3.84238100	-0.90736300
H	-4.05850300	-4.31626800	-1.73777800
H	-5.67960000	-3.89808700	-1.12889300
H	-4.38532400	-4.42652100	-0.00494200
C	-5.93938100	0.79251400	-1.83338100
H	-6.03410600	1.75469400	-1.31423000
H	-6.88661600	0.24316300	-1.76820000

H	-5.74863800	1.01301400	-2.89576200
C	-2.37117300	-3.14383000	0.67973500
C	-1.39593400	-4.00840300	0.14479600
C	-0.76714400	-4.91291500	1.00750800
H	-0.00485500	-5.58597700	0.60737800
C	-1.08882400	-4.96798700	2.35656900
H	-0.58303400	-5.67954500	3.01352800
C	-2.05497000	-4.10903800	2.87084300
H	-2.29984300	-4.15382500	3.93348900
C	-2.70858800	-3.18463800	2.05318100
C	-1.00440100	-3.98455900	-1.31865500
H	-1.65715600	-3.26440200	-1.83156600
C	-1.19719400	-5.34781500	-1.98237300
H	-0.51284300	-6.10455700	-1.56635200
H	-0.99376400	-5.27837400	-3.06230800
H	-2.22284000	-5.72591000	-1.85247600
C	0.42831900	-3.48172600	-1.49553000
H	0.57143800	-2.49834200	-1.02438200
H	0.66673400	-3.38193800	-2.56579400
H	1.14642600	-4.18752300	-1.04652000
C	-3.76683700	-2.26053600	2.62811300
H	-3.75470500	-1.34993800	2.00968100
C	-5.17041900	-2.85974700	2.51322200
H	-5.23044500	-3.81886900	3.05227900
H	-5.46106400	-3.03890300	1.47001700
H	-5.91630600	-2.17734700	2.95053300
C	-3.49187400	-1.84982800	4.07147300
H	-2.47391600	-1.45514800	4.19938700
H	-3.62112500	-2.69233300	4.76885100
H	-4.20266200	-1.06905600	4.38206000
C	-3.69658900	1.99595400	-0.59045700
C	-3.35452600	2.86621300	-1.64669600
C	-3.29910100	4.23927600	-1.38205400
H	-3.03516900	4.92392300	-2.19181200
C	-3.55952800	4.74584700	-0.11688100
H	-3.50243700	5.82105700	0.06808300
C	-3.88594900	3.87588000	0.91801100
H	-4.08646400	4.27703800	1.91286200
C	-3.96260400	2.49768800	0.70493200
C	-3.03044700	2.37776700	-3.04472700
H	-3.16846000	1.28726100	-3.06629600
C	-3.94931500	3.00053800	-4.09633600
H	-3.78534000	4.08620300	-4.18596700
H	-5.01266200	2.84918000	-3.85776700
H	-3.75450000	2.55665600	-5.08488700
C	-1.56386300	2.64006400	-3.38474500
H	-1.34624900	3.71999800	-3.41813400
H	-1.31286900	2.20940900	-4.36589000
H	-0.89851100	2.17910600	-2.64405100
C	-4.36426600	1.56703400	1.83178000
H	-3.87835900	0.60108000	1.62625800
C	-5.87181900	1.30720300	1.83559800
H	-6.42984600	2.24650400	1.97744100
H	-6.14313900	0.62310600	2.65535200
H	-6.21216500	0.85002000	0.89642800
C	-3.89610900	2.04217100	3.20319400
H	-2.82600700	2.29508700	3.20448600
H	-4.05691100	1.25317900	3.95264500
H	-4.45576600	2.92760800	3.54313700
C	3.53598900	-1.26932900	-0.62801200
C	4.64777200	-2.10576300	-1.14875200
C	5.97280800	-1.67749800	-1.02014400
H	6.18614700	-0.72345400	-0.53286400
C	7.01142100	-2.46881000	-1.50326100
H	8.04436600	-2.12797000	-1.40289500
C	6.73383800	-3.69437800	-2.10854000
H	7.55016800	-4.31581300	-2.48394900
C	5.41391700	-4.12494600	-2.23450600
H	5.19247300	-5.08343700	-2.70932500

C	4.37116000	-3.33198000	-1.76030400
H	3.33418600	-3.65649300	-1.86788800
C	3.01550000	0.18692000	-2.64633000
C	3.05032300	-0.88325300	-3.73743600
H	3.93686100	-1.52684900	-3.65780700
H	3.07344300	-0.40068400	-4.72612000
H	2.14815600	-1.51051400	-3.68187400
C	1.81373700	1.09954300	-2.87848200
H	0.87641800	0.52569700	-2.85256000
H	1.89328900	1.60522800	-3.85196000
H	1.78323900	1.87366400	-2.09653400
C	4.29540500	1.02821200	-2.65111200
H	5.18784800	0.40088400	-2.51161700
H	4.25224400	1.78247900	-1.84980200
H	4.40228800	1.55131200	-3.61419600
C	3.37480600	-2.07190300	1.78763800
C	4.86315900	-2.30138600	2.06241700
H	5.39479200	-1.34429400	2.16812800
H	5.34663000	-2.88381500	1.26712200
H	4.97901100	-2.86015700	3.00336200
C	2.74320600	-1.37026700	2.98891600
H	2.92525500	-1.94588300	3.90786000
H	1.65705600	-1.28373600	2.84819900
H	3.15544300	-0.35747500	3.11580600
C	2.66601900	-3.40891200	1.55548800
H	2.75156000	-4.04918000	2.44711800
H	3.11124900	-3.94915200	0.70646000
H	1.60006900	-3.24307100	1.34575800
C	-0.68139200	0.18369300	0.69152000
H	-0.59197200	1.26796600	0.49812800
C	-0.98342400	-0.00519600	2.17339300
H	-0.81949000	-1.06959800	2.41327400
H	-2.05285000	0.17923000	2.35520000
C	-0.17106700	0.89377400	3.09353700
H	0.91052300	0.79942300	2.92053100
H	-0.40559100	1.95515300	2.92380000
H	-0.36331200	0.66187800	4.15330200
C	1.25041500	4.29952700	0.67335700
C	2.58705000	4.53720100	-0.11983400
B	2.77761500	2.68070800	1.17520500
O	1.63478100	3.32564300	1.63885100
O	3.23516800	3.26924800	-0.01669500
H	5.34943200	2.46217700	0.98546800
C	4.91884900	1.72720900	1.69011900
O	3.64789200	2.14221300	2.10648500
H	4.83859800	0.76639600	1.14311600
C	5.82262100	1.56164200	2.89555200
H	5.39216200	0.78990600	3.55681000
H	6.79307100	1.16669600	2.54757000
C	6.01864600	2.85325000	3.67176100
H	5.04803600	3.24272900	4.01205800
H	6.65959800	2.70551100	4.55415900
H	6.48563700	3.62782000	3.04162100
C	0.72728000	5.52432900	1.40579400
H	1.44248300	5.87668100	2.15971900
H	0.51454900	6.34452500	0.70280100
H	-0.20966700	5.26764500	1.92186900
C	0.14280800	3.72811800	-0.20258200
H	0.48557500	2.83591600	-0.74300800
H	-0.71052600	3.44113500	0.42846800
H	-0.22070900	4.46119300	-0.93578700
C	2.40554900	4.89412500	-1.58363400
H	1.84991500	5.83830200	-1.69109500
H	3.39036300	5.02248100	-2.05696500
H	1.86936400	4.10825000	-2.13000300
C	3.49195300	5.56405300	0.55829900
H	3.62075100	5.32766500	1.62477200
H	4.48225900	5.52724300	0.08121400
H	3.10118100	6.58835800	0.46860100

Cartesian coordinates of the optimized geometry of vdW-**1** at PBE0-D3BJ/def2-SVP level of theory:

Ga	1.00054100	0.24244600	-0.73263500
Cl	1.07334700	0.10082600	-2.99996800
Si	-0.73991300	-1.18714200	0.25906500
N	2.82186200	-0.52640500	-0.35775500
N	1.62410200	2.09924500	-0.44708800
C	3.89964100	0.15638200	-0.69999300
C	3.85533100	1.49760400	-1.14250700
H	4.78866800	1.89545500	-1.53998900
C	2.82478700	2.41745000	-0.95864700
C	5.26808500	-0.46094900	-0.62145000
H	5.64284300	-0.64554300	-1.63981300
H	5.26552900	-1.41170000	-0.07577100
H	5.96766000	0.23458300	-0.13754700
C	3.08397100	3.84248000	-1.36328800
H	4.13917400	3.99811000	-1.61734300
H	2.79057900	4.55511000	-0.58104800
H	2.47160500	4.07589700	-2.24903300
C	2.88399800	-1.86954500	0.11728600
C	3.09959400	-2.93967900	-0.77718800
C	3.05422200	-4.24343900	-0.26866200
H	3.21553800	-5.08321300	-0.94916300
C	2.81044600	-4.48980000	1.07448700
H	2.77703100	-5.51617300	1.44777800
C	2.61427000	-3.42340000	1.94717300
H	2.43061700	-3.62159400	3.00394700
C	2.64526600	-2.10456300	1.49227500
C	3.39403500	-2.74382300	-2.25392400
H	3.36866100	-1.66545600	-2.46839400
C	4.77482000	-3.29004000	-2.62675200
H	4.80549800	-4.38706700	-2.53065900
H	5.57308800	-2.88432300	-1.98999300
H	5.01414900	-3.04553300	-3.67353500
C	2.32584700	-3.38794200	-3.13430100
H	1.33153200	-2.99545800	-2.89717500
H	2.30464700	-4.48232400	-3.00770600
H	2.52520500	-3.17475300	-4.19568500
C	2.48028500	-0.94606900	2.45721700
H	1.84269000	-0.19927300	1.95080500
C	3.83038700	-0.28235100	2.74376600
H	4.53256000	-1.01505200	3.17196300
H	3.71239900	0.53498500	3.47140600
H	4.28898800	0.14194700	1.84133800
C	1.79887800	-1.31487700	3.76940400
H	2.44614100	-1.95311700	4.39251500
H	0.83871000	-1.83226000	3.62824200
H	1.59990900	-0.40107000	4.34951000
C	0.80356200	3.14677600	0.06410300
C	-0.15685000	3.76897300	-0.75725700
C	-0.92303200	4.80976200	-0.22196200
H	-1.67235700	5.29886100	-0.84886600
C	-0.74539900	5.23221000	1.08875600
H	-1.35187300	6.04736300	1.49047300
C	0.20331400	4.60783300	1.89210400
H	0.33214800	4.94102800	2.92336400
C	0.99179300	3.56202400	1.40486500
C	-0.38854600	3.31784700	-2.18366700
H	0.44612000	2.66360200	-2.47417400
C	-0.43385300	4.47761800	-3.17527400
H	-1.31231700	5.12377100	-3.01883300
H	-0.49034000	4.09274500	-4.20500500
H	0.46228100	5.11242900	-3.09822600
C	-1.65695600	2.47215500	-2.26395400
H	-1.59335600	1.59585500	-1.60035100
H	-1.81805600	2.10270500	-3.28746800

H	-2.53548200	3.06733400	-1.96440200
C	2.04121400	2.91321600	2.29263800
H	2.12966800	1.86792800	1.95744900
C	3.42087600	3.55783300	2.12749400
H	4.13659800	3.10525400	2.83149300
H	3.37377100	4.63785500	2.34114500
H	3.82977000	3.42314300	1.11877100
C	1.66514100	2.91107100	3.77209000
H	2.36593600	2.27796500	4.33710800
H	0.65079600	2.52791600	3.94162100
H	1.72603700	3.92042400	4.20885200
N	-2.13856100	-0.06151800	0.83395600
N	-2.15819600	-1.15445200	-0.99877500
C	-2.92568300	-0.42184600	-0.18295200
C	-4.37933600	-0.14852000	-0.32976400
C	-5.32203900	-0.96862600	0.30279600
H	-4.98214400	-1.81144600	0.90747600
C	-6.68351900	-0.72129100	0.15450600
H	-7.40863800	-1.37014900	0.65085500
C	-7.11932700	0.34937300	-0.62600800
H	-8.18822900	0.54321500	-0.74172700
C	-6.18729600	1.17015400	-1.25724800
H	-6.52137900	2.01133700	-1.86881600
C	-4.82351700	0.92271100	-1.11114300
H	-4.09308400	1.56768500	-1.60041200
C	-2.49157200	-1.89464100	-2.21769400
C	-1.27618400	-2.76349100	-2.52404100
H	-1.06999500	-3.46492400	-1.70048400
H	-0.39812700	-2.12029600	-2.67649600
H	-1.43689200	-3.34367400	-3.44424600
C	-3.72107600	-2.78912500	-2.03613200
H	-3.60846800	-3.43007600	-1.14894700
H	-3.83428500	-3.44022000	-2.91605900
H	-4.64628700	-2.20737000	-1.93128400
C	-2.70582200	-0.92147500	-3.37956900
H	-3.56005300	-0.25603900	-3.18934200
H	-2.91196500	-1.47926200	-4.30595200
H	-1.80201100	-0.31481100	-3.53193900
C	-2.40757600	0.72653400	2.03671300
C	-3.47165200	0.07435300	2.92397200
H	-3.17840800	-0.95633600	3.17419200
H	-4.45923800	0.07605100	2.44396000
H	-3.55985700	0.63866800	3.86499200
C	-2.81503900	2.14706200	1.64636300
H	-2.93148900	2.76931700	2.54636700
H	-3.77216800	2.15449800	1.10499100
H	-2.04645400	2.60899100	1.01065700
C	-1.09111100	0.75776200	2.80457600
H	-0.79563900	-0.25948400	3.10039000
H	-1.19905900	1.35904500	3.71812500
H	-0.30043600	1.20997900	2.18753600
O	-1.39665900	-2.62558100	3.45024200
C	-1.25172400	-3.27272300	2.43239000
H	-0.23968400	-3.61495400	2.11389800
C	-2.42478800	-3.80401300	1.64217600
H	-2.06858300	-4.20525200	0.68167700
H	-3.09357200	-2.95663800	1.42037200
C	-3.18288300	-4.86085800	2.44175300
H	-3.52507400	-4.44207600	3.39958200
H	-4.06117800	-5.22216100	1.88611100
H	-2.54635700	-5.73183900	2.66370300

Cartesian coordinates of the optimized geometry of vdW-**2** at PBE0-D3BJ/def2-SVP level of theory:

Ga	-2.01548800	-0.45694300	-0.56114500
Cl	-1.15162900	-0.75324100	-2.59952000
Si	2.05567900	0.23364100	0.40335800

O	0.54816600	-0.47117100	0.50160100
N	-3.03111900	-2.10754600	-0.25694000
N	-3.59407100	0.64391300	-1.00753800
N	2.89357000	-0.40884300	-1.13424300
N	3.21421700	-1.15181900	0.85194900
C	-4.08664700	-2.34274300	-1.04040700
C	-4.78141400	-1.33462200	-1.71927700
H	-5.62856800	-1.66464100	-2.31958000
C	-4.63219100	0.06126200	-1.59730400
C	-4.55242100	-3.75777200	-1.23377900
H	-3.90548200	-4.23142000	-1.99047900
H	-5.58708400	-3.79651600	-1.59505900
H	-4.45905200	-4.35322600	-0.31673200
C	-5.72875600	0.90002400	-2.19126000
H	-5.79683500	1.88720800	-1.71759100
H	-6.69497500	0.38602400	-2.11215300
H	-5.52166900	1.05979300	-3.26089500
C	-2.58244900	-3.08937000	0.67804000
C	-1.53223500	-3.96837300	0.35086900
C	-1.08814400	-4.86617700	1.32826600
H	-0.26921000	-5.54949100	1.08956200
C	-1.67081100	-4.90850000	2.58737100
H	-1.31076900	-5.61778200	3.33637100
C	-2.71529300	-4.04140200	2.89327000
H	-3.16616100	-4.07680400	3.88681700
C	-3.18502000	-3.11694900	1.95848100
C	-0.88162400	-3.97867100	-1.01684200
H	-1.39398600	-3.23203000	-1.64009800
C	-1.02971000	-5.33785700	-1.70092000
H	-0.47966500	-6.12573000	-1.16163100
H	-0.62811300	-5.29580400	-2.72536700
H	-2.08232500	-5.65372300	-1.76032000
C	0.58502700	-3.55850200	-0.93660600
H	0.69304200	-2.57699100	-0.45355600
H	1.01340900	-3.48847900	-1.94822000
H	1.17633500	-4.29275600	-0.36432600
C	-4.31711200	-2.17162800	2.32052300
H	-4.21468300	-1.29139700	1.66715400
C	-5.68879900	-2.78595900	2.03205100
H	-5.83039400	-3.71312300	2.61040400
H	-5.81904400	-3.02795600	0.96934700
H	-6.49073300	-2.08497600	2.31248800
C	-4.25529100	-1.69050900	3.76819300
H	-3.26447500	-1.28926300	4.02587000
H	-4.48794000	-2.49868000	4.47894500
H	-5.00010100	-0.89726700	3.93449300
C	-3.52818200	2.06150000	-0.84046800
C	-3.15093900	2.90047400	-1.90999800
C	-3.07928300	4.27796400	-1.67276000
H	-2.78620500	4.94112800	-2.49018400
C	-3.36204000	4.81591900	-0.42518100
H	-3.29387800	5.89414200	-0.26311300
C	-3.72385000	3.97488800	0.62187000
H	-3.93726000	4.40097200	1.60343500
C	-3.81191600	2.59402000	0.43775300
C	-2.81257900	2.37807700	-3.29324700
H	-2.96830600	1.28939800	-3.29542900
C	-3.70151700	2.99920000	-4.37211100
H	-3.50263700	4.07663700	-4.48565600
H	-4.77204600	2.88726800	-4.14589000
H	-3.50634200	2.52621300	-5.34701800
C	-1.33824200	2.61039300	-3.61810900
H	-1.10153500	3.68556100	-3.66095400
H	-1.08403400	2.16432900	-4.59181900
H	-0.69127900	2.14807000	-2.86373500
C	-4.25129900	1.68866200	1.57047700
H	-3.75741200	0.71883800	1.40596900
C	-5.75815600	1.43037200	1.51921100
H	-6.31834200	2.37354400	1.61998000

H	-6.06596100	0.76294200	2.33955200
H	-6.06032700	0.95688000	0.57463000
C	-3.82969000	2.18877400	2.94709400
H	-2.75956500	2.43777600	2.97681100
H	-4.01703600	1.41415400	3.70524100
H	-4.39666800	3.08278100	3.25080200
C	3.64268600	-1.23548400	-0.40683500
C	4.73554200	-2.10275600	-0.91234400
C	6.06814900	-1.68121100	-0.86344100
H	6.30004500	-0.69011400	-0.46864100
C	7.07018600	-2.51992600	-1.34587900
H	8.11058000	-2.18869200	-1.31137100
C	6.75161800	-3.77080100	-1.87385700
H	7.54283200	-4.42368000	-2.25006100
C	5.42207000	-4.18677100	-1.92430000
H	5.16664100	-5.16491900	-2.33795900
C	4.41252800	-3.35456600	-1.44610900
H	3.36984300	-3.67623500	-1.47691400
C	2.99011100	0.00614700	-2.53821500
C	4.42542800	0.36431300	-2.93110800
H	4.89060400	1.00880600	-2.17242700
H	4.41716000	0.89512900	-3.89483100
H	5.05348300	-0.52895100	-3.04905800
C	2.44830500	-1.09636200	-3.44913300
H	3.06838700	-2.00238700	-3.38341700
H	2.46128400	-0.75534900	-4.49568400
H	1.41241400	-1.34380300	-3.17925100
C	2.10930800	1.24776700	-2.63935600
H	2.47412500	2.03752500	-1.96462000
H	1.07433100	0.99300400	-2.36682000
H	2.09835800	1.63193800	-3.66965800
C	3.69039500	-1.78703800	2.08208200
C	5.12150600	-1.34965400	2.40493400
H	5.20869700	-0.25440000	2.35891700
H	5.84303100	-1.77945500	1.69731300
H	5.39870700	-1.68698200	3.41523100
C	2.75226700	-1.30506300	3.18490000
H	3.04419500	-1.73910100	4.15184600
H	1.71666100	-1.60274600	2.96764900
H	2.79437900	-0.20831800	3.27673100
C	3.59538200	-3.31013600	1.97238600
H	3.86461500	-3.77036300	2.93506500
H	4.27778700	-3.70650400	1.20797900
H	2.56801700	-3.61206100	1.71872900
C	-0.71222500	0.10555700	0.83785100
H	-0.63258700	1.20716600	0.83207800
C	-1.14157400	-0.34642700	2.22992000
H	-0.96390800	-1.43236500	2.29750800
H	-2.23265600	-0.22413500	2.31721600
C	-0.48075900	0.37727000	3.39184400
H	0.61270400	0.33353100	3.33657400
H	-0.73467800	1.44586100	3.39327300
H	-0.79518100	-0.05974700	4.35336300
C	1.15650900	3.92057400	1.81024800
C	1.63877500	4.12718700	0.33351500
B	2.47611100	2.12869700	1.19988600
O	1.31699500	2.52970100	1.98295000
O	2.69884800	3.19421700	0.22503800
H	3.50969200	1.87645800	1.84635600
C	5.59653100	2.54723600	0.04495000
O	5.93067500	1.38669500	0.10067500
H	5.08637100	2.94646000	-0.86337500
C	5.80972700	3.54824400	1.13252100
H	6.40180900	4.37239000	0.69098700
H	4.80947500	3.97828700	1.31305400
C	6.44076500	2.99097200	2.39013800
H	7.40905800	2.51466600	2.17593200
H	6.60103100	3.78268500	3.13616300
H	5.78627000	2.22836900	2.83707700

C	2.17606300	5.51754700	0.03220900
H	1.40251100	6.28599800	0.18732600
H	3.04098000	5.75866600	0.66433700
H	2.50091800	5.56962400	-1.01808900
C	2.04877300	4.64755900	2.81803500
H	1.91312400	5.73928000	2.79284800
H	1.79764800	4.28708700	3.82636100
H	3.10945100	4.41893600	2.63871200
C	-0.29768800	4.28844800	2.05588900
H	-0.54911200	4.12156500	3.11453900
H	-0.48695700	5.34884900	1.82607500
H	-0.97569900	3.67837300	1.44534900
C	0.54494100	3.77779800	-0.67333500
H	-0.29296600	4.48903600	-0.64981400
H	0.96900800	3.78448200	-1.68698500
H	0.13853200	2.77342200	-0.48373600

Cartesian coordinates of the optimized geometry of EtCHO at M06-2X-D3/def2-SVP level of theory:

O	-1.82367500	-0.01135600	-0.26962200
C	-0.77954500	-0.26724500	0.26095600
H	-0.67136000	-1.19102300	0.88787500
C	0.46697200	0.57572300	0.16296000
H	0.60505600	1.05737200	1.14650600
H	0.28077000	1.36890600	-0.57407300
C	1.69915200	-0.26073400	-0.17641000
H	1.60307700	-0.72504200	-1.16788000
H	2.60497100	0.35897400	-0.18020200
H	1.84740800	-1.06479800	0.55971500

Cartesian coordinates of the optimized geometry of HBPin at M06-2X-D3/def2-SVP level of theory:

C	0.78224600	-0.18346500	0.05464400
C	-0.78224600	-0.18346500	-0.05464200
O	-1.06206500	1.18460500	-0.40678400
O	1.06206400	1.18460900	0.40678300
B	0.00000000	1.93686900	-0.00000100
H	-0.00000400	3.13318100	-0.00000300
C	-1.46771200	-0.44630700	1.28393800
H	-2.53739800	-0.21936600	1.18102900
H	-1.35684600	-1.49414900	1.59547100
H	-1.05352700	0.20127200	2.06982600
C	-1.34589900	-1.10016800	-1.12696200
H	-1.06482800	-2.14508600	-0.92845700
H	-2.44228300	-1.03103600	-1.12708700
H	-0.98617900	-0.81763300	-2.12345900
C	1.34590600	-1.10016400	1.12696300
H	1.06483800	-2.14508400	0.92846200
H	2.44228900	-1.03102900	1.12708300
H	0.98619000	-0.81762900	2.12346100
C	1.46770700	-0.44630700	-1.28393800
H	2.53739200	-0.21936200	-1.18103400
H	1.35684300	-1.49415000	-1.59546800
H	1.05351500	0.20126900	-2.06982500

Cartesian coordinates of the optimized geometry of **S1** at M06-2X-D3/def2-SVP level of theory:

Ga	0.89626900	0.20712800	-0.83564300
Cl	0.71828800	-0.14889300	-3.07556500
Si	-0.67972200	-1.11052900	0.43101500
N	2.81110800	-0.24041600	-0.51044900
N	1.24866900	2.15479000	-0.78135000
C	3.74174200	0.51765000	-1.06876200

C	3.47253200	1.78129300	-1.64682200
H	4.29255700	2.24449100	-2.19020000
C	2.34369000	2.57729300	-1.42875000
C	5.17305400	0.04404600	-1.08884300
H	5.27196400	-0.81905000	-1.76232000
H	5.48049000	-0.29550700	-0.09022300
H	5.84286000	0.84103800	-1.42803700
C	2.36211700	3.98705400	-1.96752600
H	3.33760300	4.23039400	-2.40213700
H	2.11289500	4.72449300	-1.19385400
H	1.58977300	4.07075600	-2.74846600
C	3.17267100	-1.45984800	0.15787200
C	3.45699600	-2.61780900	-0.59237500
C	3.77991400	-3.79246700	0.09662600
H	3.99700800	-4.70013100	-0.46988000
C	3.80355000	-3.82668100	1.48467000
H	4.04690400	-4.75389500	2.00596900
C	3.50280700	-2.67758400	2.21129800
H	3.50586200	-2.71881300	3.30015500
C	3.18922600	-1.47748300	1.56981300
C	3.31946900	-2.66284400	-2.10497100
H	3.19704000	-1.63650900	-2.48007000
C	4.52533900	-3.29496300	-2.80457100
H	4.62611500	-4.35952500	-2.54585900
H	5.47016100	-2.79899700	-2.53738500
H	4.40232300	-3.23301300	-3.89558300
C	2.03257600	-3.41520600	-2.45167000
H	1.19423800	-3.00766800	-1.87328000
H	2.12696200	-4.48382400	-2.20006100
H	1.79101800	-3.32085100	-3.52079400
C	2.90849000	-0.21730200	2.37044600
H	2.06672800	0.28724600	1.87001100
C	4.12716400	0.71570600	2.36005200
H	5.02168300	0.17928500	2.71367200
H	3.96307200	1.57094100	3.03223000
H	4.33801600	1.12021600	1.35935500
C	2.46115800	-0.49650100	3.80318900
H	3.28514700	-0.88995600	4.41904200
H	1.62328600	-1.20582100	3.80135900
H	2.12286500	0.43994300	4.27119400
C	0.34296300	3.12995200	-0.25341500
C	-0.81056300	3.49570700	-0.97107300
C	-1.69167500	4.42048900	-0.39745800
H	-2.59738800	4.70126300	-0.93919200
C	-1.42326500	4.99838000	0.83655700
H	-2.11582400	5.72404100	1.26552600
C	-0.26440500	4.64813000	1.52391300
H	-0.06357300	5.10301400	2.49433800
C	0.62895300	3.70666700	1.00727600
C	-1.12351300	2.91459200	-2.33760700
H	-0.23960200	2.36847000	-2.69489900
C	-1.44924000	4.00013400	-3.36772900
H	-2.39483600	4.51233300	-3.13365600
H	-1.55430200	3.55176500	-4.36617200
H	-0.65832600	4.76305800	-3.41425700
C	-2.27051800	1.90716000	-2.23512300
H	-2.01432900	1.06598600	-1.57007000
H	-2.51011100	1.48865900	-3.22329400
H	-3.17141100	2.40225900	-1.83570500
C	1.86415000	3.30944200	1.80473600
H	2.11958800	2.28309500	1.50420100
C	3.07484700	4.19689900	1.48386800
H	3.91397600	3.94424700	2.14940700
H	2.82611000	5.25904300	1.63718300
H	3.42526100	4.06684800	0.45189400
C	1.61312800	3.31427300	3.31459700
H	2.47339000	2.87649000	3.84075500
H	0.71806200	2.73534500	3.57932300
H	1.49135800	4.33723700	3.70154700

N	-2.23745300	-0.41231100	1.11070300
N	-2.28513100	-1.47118300	-0.76571200
C	-3.03442100	-0.88404200	0.13580700
C	-4.52115300	-0.79802900	0.14487000
C	-5.24336900	-1.62632400	1.00977000
H	-4.71025700	-2.32917800	1.65293600
C	-6.63431200	-1.56608000	1.03011400
H	-7.19583500	-2.21878800	1.69972400
C	-7.30719000	-0.67301000	0.19480100
H	-8.39675500	-0.62336200	0.21422200
C	-6.58654700	0.15411000	-0.66593300
H	-7.11000800	0.85407800	-1.31839000
C	-5.19414000	0.08986700	-0.69621800
H	-4.62301800	0.73871600	-1.36140500
C	-2.66866200	-2.30360100	-1.91433000
C	-1.43608600	-3.13872300	-2.25817400
H	-1.13062900	-3.76091900	-1.40390400
H	-0.61015400	-2.46856200	-2.52850800
H	-1.64766600	-3.79084400	-3.11795700
C	-3.83732800	-3.24530700	-1.59413200
H	-3.65317600	-3.77942900	-0.64899700
H	-3.92589300	-3.99081000	-2.39768800
H	-4.79724000	-2.71783500	-1.52056800
C	-3.00362600	-1.42379700	-3.12336500
H	-3.86725600	-0.77504400	-2.91693900
H	-3.25038500	-2.05714800	-3.98873900
H	-2.13358000	-0.80259600	-3.38001900
C	-2.51207500	0.38011800	2.31933200
C	-2.71857900	-0.56737600	3.50602100
H	-1.83761900	-1.21866000	3.60172600
H	-3.61439600	-1.18902500	3.36000700
H	-2.84666100	0.00476100	4.43707800
C	-3.70948300	1.32240700	2.14674200
H	-3.72444400	2.02648400	2.99120400
H	-4.67256900	0.79706100	2.13063400
H	-3.60653800	1.90645900	1.21860600
C	-1.27173600	1.24295900	2.57050300
H	-0.37946200	0.61428200	2.71259000
H	-1.42336300	1.85089700	3.47476300
H	-1.11250700	1.92534000	1.72216800
O	0.18483100	-1.45606200	1.91393000
C	-0.02618800	-2.67363300	1.13936400
H	0.92657100	-3.12328800	0.80852600
C	-0.91566200	-3.68106000	1.83893300
H	-1.07921800	-4.53773300	1.16358900
H	-1.90909600	-3.23640500	2.03157000
C	-0.30014500	-4.15015600	3.15497000
H	-0.12896700	-3.28704700	3.81489300
H	-0.94408200	-4.86991500	3.68104800
H	0.67551400	-4.62696500	2.97383400

Cartesian coordinates of the optimized geometry of **S2** at M06-2X-D3/def2-SVP level of theory:

Ga	1.38118200	-0.26932700	-0.38439800
Cl	0.39344300	-1.60314900	-1.91648200
Si	-2.07636000	-1.10403100	1.93601000
O	-0.80678700	-0.26968600	1.15358600
N	1.40933800	1.48986200	-1.28445700
N	3.29875000	-0.60706000	-0.68921700
N	-3.10030000	-1.76605000	0.44541300
N	-3.46537700	0.09265100	1.45180600
C	2.20103700	1.63331600	-2.34541100
C	3.25048600	0.75012500	-2.67474900
H	3.76038100	0.94632300	-3.61468600
C	3.82435400	-0.23702000	-1.85339500
C	1.99002600	2.81581800	-3.25765700
H	1.00900100	2.73783400	-3.74794500

H	2.77186400	2.86504900	-4.02238400
H	1.98096900	3.75172000	-2.68159600
C	5.08538800	-0.91144400	-2.33135000
H	5.84238700	-0.96119800	-1.53791400
H	5.50200300	-0.40199300	-3.20682500
H	4.84207900	-1.95057400	-2.60460900
C	0.45621300	2.51594200	-0.96631200
C	-0.77893600	2.56885900	-1.64169900
C	-1.66247500	3.60871400	-1.33008300
H	-2.62299500	3.66352500	-1.84664000
C	-1.34407400	4.56339500	-0.37310500
H	-2.04610100	5.36674200	-0.14436400
C	-0.13486100	4.47777400	0.31066500
H	0.09665500	5.21676400	1.07767900
C	0.78185700	3.45921200	0.03403300
C	-1.20367000	1.50629500	-2.63994600
H	-0.32846400	0.89288400	-2.89621100
C	-1.77194300	2.09458400	-3.93438400
H	-2.72795200	2.61045800	-3.75827900
H	-1.96348200	1.28928400	-4.65812200
H	-1.08545900	2.81717000	-4.40056100
C	-2.22203100	0.57396300	-1.97671100
H	-1.82562500	0.13067600	-1.05072400
H	-2.49371200	-0.24501300	-2.66054300
H	-3.13486500	1.13910000	-1.72367100
C	2.10193100	3.37894700	0.78697400
H	2.30365900	2.31076100	0.95077600
C	3.27489500	3.94175100	-0.02475600
H	3.07017800	4.97804600	-0.33613400
H	3.48535800	3.34097100	-0.92093200
H	4.18896700	3.94765400	0.58931300
C	2.04630600	4.04565100	2.16184800
H	1.16584000	3.72236600	2.73615800
H	2.01658400	5.14280100	2.07790800
H	2.94466100	3.78625000	2.73979800
C	4.05365000	-1.38002100	0.25022200
C	4.00520500	-2.78669300	0.22767300
C	4.72855600	-3.48985100	1.19786200
H	4.69696200	-4.58124800	1.19784400
C	5.47753100	-2.82533900	2.16074900
H	6.03266100	-3.39158600	2.90996500
C	5.51251700	-1.43342600	2.17217500
H	6.09840500	-0.91934500	2.93513500
C	4.80529300	-0.68699900	1.22662900
C	3.16674300	-3.54764000	-0.78462800
H	2.81923900	-2.83794100	-1.54857600
C	3.96280500	-4.64890000	-1.49043200
H	4.25488900	-5.44822800	-0.79275200
H	4.88142000	-4.25555300	-1.95068200
H	3.35115800	-5.10861300	-2.28012600
C	1.91759700	-4.12556600	-0.11074900
H	2.19490700	-4.87914300	0.64337100
H	1.25899300	-4.59552800	-0.85573900
H	1.33747200	-3.33785400	0.39122100
C	4.87925200	0.83327900	1.22715500
H	3.94255000	1.20295200	0.78342800
C	6.02656000	1.33617300	0.34016400
H	6.98170700	0.88929100	0.65764700
H	6.11926600	2.42991700	0.41545200
H	5.86535400	1.09083200	-0.71809200
C	5.00560000	1.42926200	2.62972700
H	4.23807000	1.03800000	3.31344900
H	4.89960400	2.52315800	2.57974200
H	5.99266200	1.22316700	3.07045100
C	-3.99291400	-0.79186000	0.61202500
C	-5.39513900	-0.76009700	0.10304500
C	-6.43950200	-0.99171800	1.00538500
H	-6.20513600	-1.22079500	2.04708100
C	-7.76179100	-0.94756500	0.57037700

H	-8.57224700	-1.13683500	1.27561600
C	-8.04784000	-0.66114700	-0.76533100
H	-9.08372600	-0.62095800	-1.10511500
C	-7.00866700	-0.42831100	-1.66524600
H	-7.22932400	-0.20198400	-2.70924200
C	-5.68320700	-0.48152000	-1.23523800
H	-4.86671800	-0.29035800	-1.93253300
C	-3.21957700	-3.09710500	-0.16002800
C	-4.44811200	-3.85767200	0.35319200
H	-4.45844900	-3.86234300	1.45402200
H	-4.41521400	-4.89996000	0.00246100
H	-5.38495700	-3.40969800	-0.00709100
C	-3.25087500	-2.98427200	-1.68802700
H	-4.14348500	-2.44279000	-2.03075100
H	-3.27346400	-3.98952600	-2.13488600
H	-2.34790700	-2.46382300	-2.03835400
C	-1.94403100	-3.84791400	0.23690100
H	-1.89722500	-3.99477500	1.32633500
H	-1.06393100	-3.27057200	-0.08747200
H	-1.91459000	-4.83244600	-0.25179300
C	-3.85359200	1.43082100	1.89693200
C	-4.64850600	1.33896400	3.20539200
H	-4.08290800	0.76148500	3.95119900
H	-5.61672800	0.84457000	3.03951500
H	-4.84061100	2.34420600	3.60948700
C	-2.54008200	2.18609400	2.14157300
H	-2.74558200	3.21829500	2.46180600
H	-1.93190200	2.20140100	1.22453000
H	-1.94936300	1.68945500	2.92713000
C	-4.66601600	2.18482100	0.83988300
H	-4.79902100	3.22629500	1.16786300
H	-5.66175400	1.74799400	0.68581100
H	-4.12782100	2.19508300	-0.11922800
C	0.57541800	-0.46320900	1.42554600
H	0.75874100	-1.49403800	1.79532100
C	1.07189500	0.51799200	2.48538100
H	0.72166100	1.52149700	2.19313900
H	2.17632100	0.54320800	2.45794900
C	0.61327300	0.20742000	3.90710400
H	-0.48418600	0.19073100	3.98270900
H	0.97345800	-0.78216800	4.22634600
H	0.99084100	0.95643500	4.61952500

Cartesian coordinates of the optimized geometry of Int-**3** at M06-2X-D3/def2-SVP level of theory:

Ga	1.92669100	0.06342000	-0.44470000
Cl	0.81436200	0.48671600	-2.36723900
Si	-2.03724900	0.10730500	0.59044500
O	-0.43667600	0.44097000	1.03780500
N	3.26817000	1.51009600	-0.43930700
N	3.22889700	-1.34117000	-0.93567100
N	-2.87340100	0.99222600	-0.82071300
N	-2.45323700	2.03649700	1.02621100
C	4.36075800	1.41867400	-1.19373200
C	4.75931500	0.25562100	-1.87937700
H	5.63191800	0.35715700	-2.51977500
C	4.27340900	-1.05180700	-1.70216400
C	5.26964600	2.61683800	-1.31108700
H	6.19530700	2.35216000	-1.83214300
H	5.51076200	3.01189100	-0.31398200
H	4.76848300	3.42483800	-1.85932400
C	4.98861600	-2.16388000	-2.42676300
H	5.10671200	-3.05219100	-1.79307200
H	5.96903800	-1.83422100	-2.78749300
H	4.37771400	-2.46183900	-3.29309000
C	2.99349000	2.72631500	0.26707500
C	2.48094100	3.85070300	-0.41289500

C	2.27172600	5.02361400	0.32086900
H	1.87822100	5.90637900	-0.18752200
C	2.53755700	5.08104200	1.68274700
H	2.36566300	6.00593900	2.23552700
C	2.99822400	3.94813100	2.34776400
H	3.17819200	3.99601800	3.42172000
C	3.23299200	2.75518000	1.65925900
C	2.10082100	3.82291300	-1.88673200
H	2.44799200	2.87426900	-2.32089400
C	2.71118300	4.98462000	-2.67970600
H	2.28050400	5.94783100	-2.36727200
H	2.49612500	4.86208500	-3.75107300
H	3.80111500	5.05796200	-2.55657300
C	0.57541300	3.84942000	-2.03611300
H	0.10503800	3.02763900	-1.47816900
H	0.29256900	3.74175800	-3.09456600
H	0.17780100	4.80833400	-1.66382500
C	3.75252800	1.52018700	2.38239700
H	3.21790400	0.65509800	1.95989800
C	5.24992200	1.28957900	2.14137500
H	5.83106900	2.17330500	2.44795500
H	5.47347800	1.07117100	1.08766300
H	5.59924200	0.43146500	2.73668600
C	3.46041200	1.53375200	3.88232100
H	2.40925900	1.78044300	4.09038700
H	4.09556900	2.26165200	4.41025300
H	3.66909300	0.54226000	4.31018200
C	2.92615000	-2.69300300	-0.57371300
C	2.10909200	-3.48982800	-1.39781100
C	1.78462300	-4.77832300	-0.95736300
H	1.14418300	-5.40755800	-1.57966100
C	2.25187000	-5.26350700	0.25699000
H	1.97997800	-6.26747800	0.58680400
C	3.06594900	-4.46479100	1.05729000
H	3.42533300	-4.85641700	2.00914500
C	3.42119500	-3.17299300	0.66189100
C	1.56840600	-2.99908700	-2.73083600
H	1.98375400	-2.00032400	-2.92583800
C	1.97434100	-3.92711000	-3.88153700
H	1.50024700	-4.91521100	-3.77926100
H	3.06178600	-4.08592200	-3.92095100
H	1.65091100	-3.50297400	-4.84332600
C	0.04472600	-2.84952800	-2.68675900
H	-0.43463300	-3.83688800	-2.59130800
H	-0.31987000	-2.37637600	-3.61067900
H	-0.27885100	-2.22063100	-1.84317500
C	4.34690300	-2.32046100	1.52266800
H	4.01650100	-1.27353000	1.42043600
C	5.79859900	-2.38083000	1.02666600
H	6.14598600	-3.42482800	0.98291200
H	6.45750700	-1.83085500	1.71543200
H	5.91792600	-1.93320700	0.03166400
C	4.30345100	-2.68961100	3.00683900
H	3.27513500	-2.76193800	3.38894500
H	4.83728600	-1.92875500	3.59545300
H	4.80404000	-3.65188400	3.19342500
C	-3.00448700	2.15669800	-0.15635900
C	-3.69780700	3.37392900	-0.66988100
C	-5.03208500	3.59293600	-0.31733400
H	-5.54783500	2.86694600	0.31488000
C	-5.69074500	4.73939300	-0.75782400
H	-6.73257900	4.90640000	-0.48119700
C	-5.01607500	5.67397000	-1.54301800
H	-5.53048100	6.57369000	-1.88359700
C	-3.68299200	5.45651900	-1.89317800
H	-3.15299500	6.18382400	-2.50972400
C	-3.02448000	4.30727300	-1.46178000
H	-1.98812000	4.12300300	-1.74576900
C	-3.54084000	0.53932500	-2.05725100

C	-5.00986700	0.19993000	-1.77458100
H	-5.07416200	-0.62445300	-1.04827100
H	-5.50832500	-0.12178500	-2.70174600
H	-5.55169600	1.07437000	-1.38427200
C	-3.43874900	1.58653900	-3.17447600
H	-4.09808500	2.44823900	-3.01238600
H	-3.73033100	1.11896800	-4.12600100
H	-2.39960600	1.93731900	-3.26727600
C	-2.82144100	-0.72618000	-2.53251300
H	-2.93865000	-1.53982800	-1.80513400
H	-1.74996200	-0.52203300	-2.67069400
H	-3.25442700	-1.04806100	-3.49165400
C	-1.96167400	3.08952900	1.93064300
C	-3.01605700	4.16180200	2.24128500
H	-3.96502500	3.69781900	2.55123000
H	-3.21183600	4.82453300	1.38834500
H	-2.65316300	4.78368300	3.07314400
C	-1.54095100	2.43290500	3.25088400
H	-0.96873600	3.15895700	3.84691300
H	-0.90535400	1.55936100	3.05305100
H	-2.41345300	2.12563500	3.84035400
C	-0.72236600	3.73835200	1.30417400
H	-0.30481900	4.49788700	1.98138600
H	-0.97684200	4.23158800	0.35506200
H	0.04562600	2.97254100	1.11395700
C	0.61959700	-0.50084000	0.96320800
H	0.24170600	-1.44777300	0.51762100
C	1.09078400	-0.84278000	2.37146100
H	1.35511200	0.09121100	2.89258300
H	2.01130700	-1.44206300	2.30464700
C	0.03625900	-1.60135400	3.17068700
H	-0.89476900	-1.02144700	3.25187500
H	-0.22416500	-2.55206800	2.68003700
H	0.38428100	-1.82427700	4.19025500
C	-3.42603400	-0.62724600	1.73131700
O	-3.05880000	-1.95674500	2.11453100
H	-4.28315900	-0.69514600	1.03089000
C	-3.91416400	0.07657500	2.98799200
H	-3.09439300	0.09288900	3.72329500
H	-4.13961200	1.12337000	2.73506300
C	-5.13590700	-0.60068000	3.60123200
H	-5.47198000	-0.08116100	4.51009900
H	-5.97521300	-0.61390000	2.88804800
H	-4.90379000	-1.64079100	3.86811600
H	-1.72107100	-1.20361600	-0.09079900
C	-2.96748300	-5.06301200	0.42977400
C	-4.02827400	-4.27107700	-0.41640100
O	-3.83650200	-2.92515300	0.04157100
O	-2.88387300	-4.28202200	1.62584500
C	-3.80360800	-4.32527500	-1.91817800
H	-3.86486200	-5.36243800	-2.28098000
H	-4.57798600	-3.73379100	-2.42753000
H	-2.82528000	-3.90861400	-2.18964400
C	-5.46278500	-4.66505600	-0.07349500
H	-5.62064700	-4.64447100	1.01453700
H	-6.14660500	-3.93922600	-0.53484100
H	-5.70906000	-5.66858000	-0.44746700
C	-1.58041000	-5.04124100	-0.20592500
H	-0.85315900	-5.42042700	0.52518000
H	-1.53240700	-5.66225700	-1.11294400
H	-1.29043300	-4.01138800	-0.46437500
C	-3.37307600	-6.48369600	0.78314400
H	-3.53401200	-7.07959800	-0.12771700
H	-2.57197800	-6.95539600	1.36869100
H	-4.28907300	-6.49762100	1.38606700
B	-3.24388100	-2.99869400	1.28780600

Cartesian coordinates of the optimized geometry of TS-**2** at M06-2X-D3/def2-SVP level of theory:

Ga	0.95073300	-0.03088300	-0.70726500
Cl	0.85132000	-0.79496000	-2.84726900
Si	-1.03674600	-0.44598400	0.52809300
N	2.94912700	-0.18579200	-0.44946800
N	1.08810100	1.96211100	-1.06217100
C	3.74176000	0.57244600	-1.21090400
C	3.32839600	1.72044200	-1.90686000
H	4.08472400	2.20108600	-2.52207700
C	2.13754300	2.44151300	-1.72019200
C	5.20592600	0.21773000	-1.33470600
H	5.31732900	-0.75575100	-1.83172000
H	5.67193500	0.12038100	-0.34453300
H	5.73947400	0.97810900	-1.91399200
C	2.12184100	3.85967100	-2.24031800
H	2.34613000	4.55644700	-1.41826300
H	1.13636500	4.13485900	-2.63681000
H	2.88496500	3.98715900	-3.01665600
C	3.58712000	-1.21837800	0.32210000
C	3.92259500	-2.45274200	-0.26728700
C	4.57033700	-3.41306800	0.51892100
H	4.82857800	-4.37650200	0.07538500
C	4.88725500	-3.16378600	1.84724200
H	5.39518500	-3.92350500	2.44309500
C	4.54254000	-1.94247500	2.41938300
H	4.77769700	-1.76002500	3.46799300
C	3.88677900	-0.95731400	1.67752600
C	3.55867500	-2.79519900	-1.70150200
H	3.25266500	-1.87221000	-2.21295000
C	4.71633000	-3.41704600	-2.48729600
H	4.98662400	-4.40866800	-2.09455900
H	5.62092500	-2.79141100	-2.46153200
H	4.42403100	-3.55048300	-3.53893000
C	2.34516100	-3.72797400	-1.70046700
H	1.54176600	-3.30190400	-1.08616800
H	2.61201600	-4.71013000	-1.27733800
H	1.95742000	-3.87169100	-2.71985700
C	3.48525800	0.35921500	2.32687000
H	2.55849800	0.68996500	1.83140300
C	4.53536000	1.45195600	2.09229600
H	5.51548400	1.13784900	2.48429000
H	4.24593700	2.38110800	2.60781500
H	4.64851000	1.68645900	1.02381300
C	3.17855100	0.20219500	3.81634400
H	4.08750700	-0.00910400	4.39923200
H	2.45673500	-0.61035800	3.98710100
H	2.74623400	1.12874200	4.21706000
C	0.06851300	2.88852000	-0.67989800
C	-1.17042700	2.91311800	-1.35865800
C	-2.09811700	3.89849300	-1.00236900
H	-3.05676900	3.94463600	-1.51924800
C	-1.82220500	4.83203600	-0.00733700
H	-2.55773900	5.59795500	0.24320800
C	-0.61725000	4.76677800	0.68044100
H	-0.41878300	5.47420800	1.48765200
C	0.33612400	3.79191200	0.37031900
C	-1.48043500	1.92448000	-2.47216100
H	-1.07927700	0.95027700	-2.16079800
C	-0.78122500	2.28664900	-3.78610900
H	-1.07484000	3.29448500	-4.12104100
H	-1.05702500	1.56392000	-4.56795300
H	0.31125400	2.24440100	-3.68393700
C	-2.97949100	1.73193100	-2.69230500
H	-3.48411800	1.50062300	-1.74015000
H	-3.14927800	0.90014700	-3.39037000
H	-3.45263900	2.62576700	-3.12702500
C	1.57651100	3.66471400	1.24070500
H	2.31393300	3.03444400	0.72074000

C	2.24464300	5.00644600	1.54929900
H	3.19593700	4.84106300	2.07599600
H	1.61661200	5.63248800	2.20052200
H	2.45648100	5.57913000	0.63447600
C	1.18780500	2.94380800	2.53680200
H	2.05836400	2.84857000	3.20155300
H	0.78970300	1.93667100	2.33532100
H	0.40884400	3.51015700	3.07157000
N	-2.79737500	0.15620800	0.97927300
N	-2.32477700	-1.67689700	-0.07681600
C	-3.31986500	-0.90938000	0.40919300
C	-4.77370800	-1.23377000	0.34859700
C	-5.30007500	-2.17350900	1.24062200
H	-4.64001200	-2.65349700	1.96583800
C	-6.65361800	-2.49530200	1.19265600
H	-7.06181100	-3.22760200	1.89029400
C	-7.48360100	-1.88779600	0.24924200
H	-8.54255700	-2.14634400	0.20768700
C	-6.96039000	-0.94804800	-0.63786500
H	-7.60718300	-0.46954400	-1.37416500
C	-5.60751100	-0.61449100	-0.58560600
H	-5.19455200	0.12567900	-1.27349300
C	-2.44326000	-2.65293000	-1.18796600
C	-1.10501100	-3.36961500	-1.32091100
H	-0.87762000	-3.96342000	-0.42633900
H	-0.30143000	-2.64786600	-1.49377900
H	-1.13521600	-4.04234600	-2.19012100
C	-3.52577500	-3.71046300	-0.92917300
H	-3.42651900	-4.12912400	0.08310100
H	-3.38626600	-4.52776800	-1.65117400
H	-4.54483400	-3.32565100	-1.05790500
C	-2.74013400	-1.90092700	-2.49045900
H	-3.68673300	-1.34519500	-2.41391100
H	-2.82813900	-2.61067500	-3.32640700
H	-1.92119400	-1.20264400	-2.72193900
C	-3.34035300	1.08925900	1.97784700
C	-3.84566200	0.31933300	3.20308200
H	-3.05928900	-0.36173600	3.56104900
H	-4.75079500	-0.25919700	2.97265300
H	-4.09155200	1.02556700	4.00962700
C	-4.44775700	1.96156200	1.38077000
H	-4.75553300	2.71869100	2.11708100
H	-5.33356300	1.36977300	1.11292800
H	-4.07440300	2.48358900	0.48700800
C	-2.16059900	1.96870100	2.39250300
H	-1.38751300	1.35225600	2.87393900
H	-2.49141500	2.74606900	3.09644200
H	-1.72616500	2.45952400	1.50905100
O	-0.44910400	-0.76797600	2.05586300
C	0.54850800	-1.58766600	1.37207800
H	1.53744400	-1.23029200	1.68714100
C	0.40600900	-3.04469300	1.75497500
H	1.04928800	-3.65921700	1.10200400
H	-0.63504000	-3.36114500	1.57445800
C	0.76748400	-3.28962800	3.22105300
H	0.59049700	-4.33283100	3.52418300
H	1.83118500	-3.06232300	3.39520200
H	0.16678200	-2.63247200	3.86698800

Cartesian coordinates of the optimized geometry of TS-**7** at M06-2X-D3/def2-SVP level of theory:

Ga	2.30009100	-0.36144700	-0.39549900
Cl	1.23176900	0.25837500	-2.28192500
Si	-1.50463600	0.70639200	0.47254100
O	0.10386100	0.63026400	0.94984400
N	3.87751900	0.81521900	-0.31947600
N	3.29829900	-1.96208500	-0.97776300

N	-2.02128100	1.59975500	-1.06099700
N	-1.42513400	2.67527300	0.71780000
C	4.91525800	0.58843200	-1.11884700
C	5.12142700	-0.61720300	-1.81652000
H	6.00225600	-0.64879300	-2.45294200
C	4.42024800	-1.82919600	-1.68048200
C	5.94451800	1.67232800	-1.31867800
H	6.88074300	1.25477600	-1.70472800
H	6.14000700	2.22477800	-0.39104000
H	5.55665100	2.39381100	-2.05352100
C	4.99892100	-3.04045500	-2.36768100
H	5.08552600	-3.88421500	-1.66908900
H	5.98179900	-2.81684500	-2.79483900
H	4.32362200	-3.36428100	-3.17261300
C	3.79093800	2.01787900	0.45903300
C	3.30788500	3.20768700	-0.11916200
C	3.19912800	4.34248900	0.69415900
H	2.82002200	5.27186000	0.26309200
C	3.55344400	4.30365700	2.03604400
H	3.45326500	5.19626700	2.65531500
C	4.03104500	3.11944400	2.59299400
H	4.30438400	3.09895600	3.64808800
C	4.16345800	1.96178800	1.82219800
C	2.88077500	3.29544000	-1.57444900
H	3.09317500	2.33261700	-2.05904700
C	3.62592800	4.40216000	-2.32959500
H	3.35723800	5.39563800	-1.93970700
H	3.35533400	4.38141600	-3.39540500
H	4.71764500	4.30310300	-2.24837700
C	1.36989400	3.52537700	-1.67405200
H	0.80802300	2.81888200	-1.04353400
H	1.03251600	3.39273700	-2.71379900
H	1.12868100	4.55310600	-1.35826000
C	4.73946100	0.68586800	2.42075400
H	4.19441900	-0.15607400	1.96693300
C	6.22130100	0.51478000	2.05813300
H	6.79773000	1.39877100	2.37217900
H	6.37134800	0.36797700	0.98036700
H	6.63963900	-0.36329500	2.57248000
C	4.56762200	0.59219100	3.93679600
H	3.53374700	0.79570100	4.24896400
H	5.23002000	1.29927000	4.45909400
H	4.83783500	-0.41700500	4.28047000
C	2.74760200	-3.26540200	-0.74872700
C	1.95841900	-3.90417800	-1.72686500
C	1.48407900	-5.19273800	-1.45366700
H	0.87565200	-5.70489300	-2.20188700
C	1.76115500	-5.82519300	-0.24806900
H	1.38507400	-6.83201100	-0.05906500
C	2.50127300	-5.16255300	0.72704200
H	2.69527700	-5.65504200	1.68051000
C	3.00567100	-3.87994400	0.49701700
C	1.55145600	-3.22848700	-3.02610800
H	2.11845100	-2.29132300	-3.12416000
C	1.82512200	-4.09827700	-4.25719100
H	1.18422000	-4.99252600	-4.26710500
H	2.86979100	-4.43912100	-4.30365100
H	1.60875000	-3.53045700	-5.17333500
C	0.06768700	-2.84758600	-2.96473500
H	-0.56418300	-3.74670600	-2.87505600
H	-0.23066300	-2.30093900	-3.87144500
H	-0.13193600	-2.18814700	-2.10858200
C	3.85045200	-3.17935100	1.54867700
H	3.67773000	-2.09974800	1.42882700
C	5.34818100	-3.41439500	1.31838800
H	5.57352800	-4.49215300	1.31623600
H	5.93613200	-2.94391000	2.12085100
H	5.68667100	-2.98708700	0.36403900
C	3.46052400	-3.55528400	2.97812400

H	2.37407000	-3.48269000	3.13412300
H	3.95427500	-2.87904600	3.69154900
H	3.77564900	-4.57926900	3.22904000
C	-1.96813300	2.81186200	-0.46595400
C	-2.55777700	4.07904200	-0.99265500
C	-3.81179000	4.46463600	-0.50656800
H	-4.34963700	3.80264000	0.17561400
C	-4.37690400	5.67223000	-0.90966500
H	-5.35730300	5.96415300	-0.53121900
C	-3.69036600	6.50259200	-1.79606500
H	-4.13057400	7.45058000	-2.10849700
C	-2.44375400	6.11595500	-2.28662400
H	-1.90524400	6.76054900	-2.98251500
C	-1.87811400	4.90561200	-1.88903300
H	-0.90064400	4.60467700	-2.26440900
C	-2.67634700	1.24737600	-2.34163400
C	-4.16174000	1.63314600	-2.33548000
H	-4.63442200	1.23101600	-1.42597800
H	-4.65401600	1.18208400	-3.21005900
H	-4.31306900	2.71901500	-2.39290800
C	-1.92972100	1.90536800	-3.50766700
H	-2.02524400	2.99847700	-3.48938700
H	-2.35299100	1.55217700	-4.45950100
H	-0.86519400	1.63096800	-3.47063000
C	-2.59350800	-0.26872900	-2.50611800
H	-3.25641400	-0.76273600	-1.78234300
H	-1.55457300	-0.60672400	-2.38865500
H	-2.93667900	-0.53717300	-3.51569400
C	-1.11226900	3.64888700	1.77307200
C	-2.32627100	3.84500400	2.69171300
H	-2.64714100	2.89039400	3.13147400
H	-3.17182800	4.27829900	2.13874300
H	-2.06707400	4.53130600	3.51158700
C	0.04807000	3.06328700	2.58702300
H	0.31962600	3.75782400	3.39563800
H	0.92432200	2.89290800	1.94382700
H	-0.23271300	2.09668700	3.02797200
C	-0.66745400	5.00982900	1.22243900
H	-0.24836900	5.60293500	2.04852400
H	-1.49469100	5.58224500	0.78361000
H	0.11727100	4.87516100	0.46546700
C	0.98459000	-0.46772800	1.11274800
H	0.43807400	-1.41408200	0.96747900
C	1.50918700	-0.43624500	2.54651800
H	1.91583300	0.57204600	2.72896700
H	2.34566700	-1.14177600	2.66335200
C	0.42963800	-0.76018600	3.57283800
H	-0.45183800	-0.12177600	3.42424600
H	0.09664300	-1.80606400	3.47924800
H	0.78606300	-0.61246300	4.60281000
C	-3.18466600	0.56514300	1.42385900
O	-4.07057300	-0.14284200	0.59158800
H	-3.53332400	1.61491400	1.38884600
C	-3.19306000	0.17960500	2.90441200
H	-2.92648400	-0.88079800	3.02490000
H	-2.39572700	0.75817600	3.40454100
C	-4.53366600	0.46870500	3.57541300
H	-4.51327800	0.19421900	4.64015600
H	-4.77770800	1.54143100	3.50538700
H	-5.32923200	-0.10633300	3.08475100
H	-1.48059800	-0.85234500	-0.01034300
C	-6.13351100	-2.53470800	-0.45185800
C	-6.22410600	-2.88761600	1.07274100
O	-5.39122600	-1.90259100	1.65496200
O	-4.79084300	-2.09989700	-0.57336400
C	-7.62123900	-2.77616600	1.66588900
H	-8.32552700	-3.44655400	1.14955200
H	-7.59198700	-3.05716700	2.72856000
H	-7.99115600	-1.74557700	1.59710000

C	-5.63835700	-4.27132800	1.36848600
H	-4.66168900	-4.38265100	0.87651100
H	-5.48962800	-4.36079900	2.45387100
H	-6.30044100	-5.08225100	1.03107700
C	-7.04841100	-1.36394400	-0.81878300
H	-6.77716100	-1.01424500	-1.82519700
H	-8.11072000	-1.64924400	-0.81694700
H	-6.89528100	-0.53285900	-0.11520800
C	-6.37477800	-3.70676500	-1.39173900
H	-7.37638300	-4.13684400	-1.23811500
H	-6.30332600	-3.36216300	-2.43355100
H	-5.62304200	-4.49221400	-1.24151700
B	-4.39821400	-1.53004800	0.69221800
C	-2.15836700	-2.40665500	0.22370400
H	-2.45845300	-2.31695000	-0.83173000
O	-3.03550900	-2.26059700	1.11163500
C	-1.01243300	-3.31695800	0.55890500
H	-0.17396000	-3.18295200	-0.13718800
H	-0.67208700	-3.09979700	1.58056400
C	-1.54930900	-4.75210000	0.48211200
H	-0.75257600	-5.46472400	0.72779000
H	-2.37827800	-4.88495500	1.18990600
H	-1.91819500	-4.98386500	-0.52856700

Cartesian coordinates of the optimized geometry of TS-**10** at M06-2X-D3/def2-SVP level of theory:

Ga	-1.91186800	-0.61913000	-0.60063100
Cl	-1.14852800	-0.82259800	-2.70360800
Si	2.17102300	0.53424200	0.35836400
O	0.64964700	-0.12582500	0.25943100
N	-2.68408600	-2.39493500	-0.23781700
N	-3.66174000	0.23456500	-0.93355500
N	2.98768700	0.15063600	-1.23282000
N	3.23571300	-0.95297100	0.59836000
C	-3.74584200	-2.78233700	-0.94289100
C	-4.62574200	-1.88659600	-1.57320900
H	-5.45819200	-2.33370200	-2.11111200
C	-4.65204000	-0.48047400	-1.45363700
C	-4.01738800	-4.25819000	-1.09182900
H	-3.24700700	-4.68468200	-1.75394700
H	-5.00343800	-4.43920400	-1.53298600
H	-3.93925900	-4.78520200	-0.13239400
C	-5.89221800	0.22834000	-1.93581200
H	-6.25850500	0.92679300	-1.17010600
H	-6.68120800	-0.48893500	-2.18402400
H	-5.65949200	0.82725700	-2.82721200
C	-2.00431100	-3.31035400	0.62911400
C	-0.88794500	-4.03471500	0.17033600
C	-0.20860800	-4.85101500	1.08312700
H	0.66582600	-5.41237500	0.74629700
C	-0.62585100	-4.95675600	2.40381700
H	-0.08185200	-5.59595600	3.10076100
C	-1.73986500	-4.24172200	2.83846900
H	-2.05475000	-4.32735700	3.87900400
C	-2.44458200	-3.40564700	1.96999900
C	-0.39711900	-3.94400800	-1.26475500
H	-1.13231800	-3.36655500	-1.84098500
C	-0.27225200	-5.32565900	-1.91411800
H	0.51212000	-5.92802600	-1.43101300
H	-0.00195600	-5.22041700	-2.97494100
H	-1.21296000	-5.89281500	-1.85254500
C	0.93330300	-3.18456100	-1.34319000
H	0.88162500	-2.21084500	-0.83122800
H	1.19995200	-2.99786200	-2.39491800
H	1.73687000	-3.77592100	-0.87400600
C	-3.64854500	-2.61327500	2.46495900
H	-3.69553500	-1.69068400	1.86524700

C	-4.96761400	-3.36212500	2.23184100
H	-4.93547200	-4.35625500	2.70435000
H	-5.18533700	-3.49277600	1.16387400
H	-5.80562200	-2.80131200	2.67285100
C	-3.53487400	-2.21623800	3.93863400
H	-2.56336800	-1.75321600	4.16611500
H	-3.66135800	-3.08587900	4.60095700
H	-4.32954500	-1.50010800	4.19650000
C	-3.78760300	1.65023700	-0.75849700
C	-3.63789500	2.53007300	-1.84988000
C	-3.78510100	3.90181000	-1.61151600
H	-3.67630800	4.59946000	-2.44451900
C	-4.04529900	4.39281200	-0.33798600
H	-4.15390700	5.46655800	-0.17664700
C	-4.14417700	3.51302000	0.73706600
H	-4.31834700	3.90683000	1.73918000
C	-4.01338100	2.13533300	0.54942100
C	-3.26695500	2.05403800	-3.24612000
H	-3.32881700	0.95627400	-3.26815600
C	-4.19456700	2.62039700	-4.32669200
H	-4.06631100	3.70815100	-4.43215700
H	-5.25645500	2.43345800	-4.10969900
H	-3.95825900	2.16752200	-5.30030700
C	-1.81177400	2.42254400	-3.55830600
H	-1.68814000	3.51721100	-3.58756700
H	-1.51440400	2.01102400	-4.53435300
H	-1.12605600	2.01322700	-2.80468600
C	-4.12731300	1.16649600	1.71509100
H	-3.41132400	0.35604300	1.51630100
C	-5.51787700	0.52514800	1.79110000
H	-6.29394500	1.29960000	1.89200000
H	-5.58307900	-0.13956000	2.66651700
H	-5.74122100	-0.07863200	0.89985200
C	-3.74358300	1.78085200	3.05963700
H	-2.78270900	2.31139500	3.00016300
H	-3.64871500	0.98865700	3.81727800
H	-4.50767200	2.48828700	3.41691900
C	3.72575200	-0.77838800	-0.62706800
C	4.93378700	-1.43180400	-1.18953200
C	6.15877900	-0.76590000	-1.07132100
H	6.18005500	0.18379800	-0.52910900
C	7.30103800	-1.34747500	-1.61663000
H	8.26144900	-0.83779100	-1.52817200
C	7.22113800	-2.57926300	-2.26959700
H	8.11935000	-3.02859900	-2.69596700
C	5.99710900	-3.23912800	-2.37627900
H	5.93539800	-4.20114900	-2.88655300
C	4.84678300	-2.66603100	-1.83534100
H	3.87888900	-3.16335800	-1.92649400
C	3.15476100	0.87013500	-2.50419700
C	4.16228600	2.01060600	-2.32414600
H	3.80243300	2.71144200	-1.55748400
H	4.27732000	2.55463100	-3.27411300
H	5.14309200	1.62416100	-2.01541100
C	3.58721600	-0.07071100	-3.63232100
H	4.63230400	-0.39245400	-3.53383600
H	3.48653700	0.46035500	-4.58972800
H	2.93938700	-0.95984100	-3.66258300
C	1.77424800	1.44527200	-2.83000700
H	1.44645000	2.10758400	-2.01466400
H	1.03738200	0.64040800	-2.95799200
H	1.82205500	2.04292100	-3.75239600
C	3.71575700	-1.77828300	1.71667200
C	5.22303100	-1.61833500	1.95302600
H	5.48598600	-0.55337900	1.87940600
H	5.81191800	-2.18711300	1.22057200
H	5.47470200	-1.99998400	2.95391800
C	2.95161800	-1.27898200	2.94121300
H	3.24105600	-1.84906100	3.83478600

H	1.86952900	-1.39983500	2.78627200
H	3.17679300	-0.21578500	3.11603900
C	3.35592200	-3.24556500	1.46666400
H	3.68912800	-3.86055800	2.31619000
H	3.85172100	-3.62090900	0.55906300
H	2.26773800	-3.35853100	1.35289100
C	-0.65023400	0.14732500	0.76580900
H	-0.80432900	1.23999000	0.85151000
C	-0.80114300	-0.46084600	2.16350800
H	-0.38775300	-1.48390800	2.12696000
H	-1.86960100	-0.58317400	2.40108700
C	-0.14755600	0.35173100	3.27678000
H	0.90160800	0.58767400	3.05379700
H	-0.65096000	1.32069100	3.40135800
H	-0.17925300	-0.19145700	4.23395600
C	0.62152600	4.16546000	1.67769500
C	0.90024300	4.34726300	0.14663800
B	2.03970500	2.51723300	0.98988200
O	0.99825100	2.80552600	1.89424900
O	1.98553600	3.44637200	-0.08251800
H	3.27895100	2.71839700	1.60571100
C	4.56263800	2.53349900	1.26718200
O	4.71225600	1.33811700	0.85613700
H	4.59733300	3.36205700	0.51734600
C	5.17250100	2.95204200	2.60502500
H	6.26668700	2.97400100	2.46182300
H	4.85884900	3.98251300	2.83851300
C	4.80567400	2.00419600	3.73581300
H	5.14990900	0.98544800	3.51123200
H	5.24942700	2.31624500	4.69153800
H	3.71044300	1.97283800	3.86224800
C	1.33027800	5.75267400	-0.24983800
H	0.54833600	6.48543900	0.00145700
H	2.26302300	6.03909800	0.25086500
H	1.50138000	5.78851800	-1.33473300
C	1.52224000	5.04568600	2.54480800
H	1.23920800	6.10597400	2.48477800
H	1.43436600	4.71346600	3.58876000
H	2.57441500	4.94376100	2.24052100
C	-0.83484400	4.33629800	2.07658400
H	-0.93993100	4.18396100	3.16104200
H	-1.19422400	5.34794300	1.83198900
H	-1.47138300	3.60758700	1.55692200
C	-0.27550300	3.90465600	-0.72124700
H	-1.14360400	4.57034600	-0.61224800
H	0.03924300	3.91210000	-1.77369700
H	-0.59304200	2.87977200	-0.46960000

Cartesian coordinates of the optimized geometry of EtCHO at PBE-D3BJ/def2-SVP level of theory:

O	-1.84821200	-0.00558600	-0.26610200
C	-0.78451600	-0.27328000	0.25252200
H	-0.65965900	-1.21976400	0.87188800
C	0.47083800	0.56846100	0.16649500
H	0.60115700	1.03459600	1.17124200
H	0.28694600	1.39021000	-0.55476700
C	1.71738200	-0.25379100	-0.17834600
H	1.64438800	-0.69004300	-1.19503200
H	2.63013600	0.37226200	-0.14481600
H	1.86050100	-1.09091100	0.53627300

Cartesian coordinates of the optimized geometry of HBPin at PBE-D3BJ/def2-SVP level of theory:

C	0.79055800	-0.18889000	0.04922000
C	-0.79055900	-0.18888700	-0.04922200

O	-1.08590800	1.20158000	-0.38327600
O	1.08591200	1.20157800	0.38327500
B	0.00000200	1.94673200	0.00000000
H	0.00000400	3.15620900	-0.00000200
C	-1.47893700	-0.47284800	1.29073100
H	-2.55714200	-0.23983000	1.19476900
H	-1.37568300	-1.53463300	1.58804900
H	-1.06403300	0.16140200	2.09827400
C	-1.36502300	-1.08691400	-1.14054400
H	-1.09172000	-2.14615100	-0.96136300
H	-2.47014200	-1.01324500	-1.14097200
H	-1.00490700	-0.79318100	-2.14324900
C	1.36501700	-1.08691600	1.14054600
H	1.09171400	-2.14615300	0.96136500
H	2.47013600	-1.01324900	1.14097700
H	1.00489700	-0.79318100	2.14324800
C	1.47894000	-0.47285200	-1.29073100
H	2.55714500	-0.23983700	-1.19476500
H	1.37568400	-1.53463700	-1.58804900
H	1.06403900	0.16139900	-2.09827500

Cartesian coordinates of the optimized geometry of **S1** at PBE-D3BJ/def2-SVP level of theory:

Ga	0.89565600	0.20082600	-0.80654400
Cl	0.74555500	-0.18154700	-3.04508600
Si	-0.68440600	-1.06685200	0.48890000
N	2.84184300	-0.22310300	-0.50765400
N	1.19686800	2.17135100	-0.80436800
C	3.75488800	0.57034800	-1.08318200
C	3.44213300	1.82747700	-1.66262200
H	4.25810300	2.30743700	-2.21848700
C	2.29339300	2.61097400	-1.46546600
C	5.20470200	0.14817200	-1.15040800
H	5.41676600	-0.26524100	-2.15820400
H	5.44815700	-0.63365900	-0.40916900
H	5.87235300	1.01767800	-1.00357900
C	2.27966600	4.00341700	-2.05281700
H	3.29648700	4.34231600	-2.32061400
H	1.81417500	4.74211800	-1.37432700
H	1.66368800	3.98851900	-2.97740300
C	3.21976700	-1.44211700	0.15690000
C	3.46747400	-2.62228600	-0.59660000
C	3.78238700	-3.80487500	0.10217900
H	3.97482100	-4.72667700	-0.46801800
C	3.84566200	-3.82955500	1.49778500
H	4.08373800	-4.76590000	2.02523500
C	3.60295900	-2.65699300	2.22441300
H	3.65012500	-2.68299500	3.32164200
C	3.29201300	-1.44716400	1.57940900
C	3.39840600	-2.66494600	-2.11917700
H	3.12970400	-1.65294100	-2.48188800
C	4.74489600	-3.06865200	-2.74649900
H	5.01601700	-4.11158600	-2.48074300
H	5.57620900	-2.41791500	-2.41192400
H	4.68848400	-3.01337200	-3.85300600
C	2.28532400	-3.61444000	-2.58895000
H	1.32418400	-3.36263500	-2.10782800
H	2.52363300	-4.67029200	-2.34277100
H	2.14573700	-3.54202000	-3.68654800
C	3.08929300	-0.16907100	2.38406900
H	2.29430800	0.40496600	1.86379000
C	4.37176500	0.68485700	2.38622200
H	5.21814400	0.11727800	2.82576700
H	4.22963900	1.60229600	2.99320800
H	4.66831800	1.00289800	1.36872400
C	2.59891100	-0.41704600	3.81480200
H	3.37692200	-0.89509400	4.44614900

H	1.69139100	-1.04978300	3.79629200
H	2.34113000	0.54696900	4.29643800
C	0.26413300	3.12071200	-0.27176100
C	-0.92545300	3.43904400	-0.97725100
C	-1.83441800	4.34074300	-0.38943700
H	-2.76517900	4.58839600	-0.92326200
C	-1.56461500	4.94406300	0.84319100
H	-2.28322500	5.65278700	1.28210900
C	-0.37277500	4.64504300	1.51459300
H	-0.16463600	5.12217900	2.48350500
C	0.55358400	3.72695500	0.98706400
C	-1.22191300	2.86271900	-2.35455500
H	-0.34926300	2.25529800	-2.66702600
C	-1.42080400	3.96881500	-3.40469600
H	-2.32885900	4.57378500	-3.20084000
H	-1.53613000	3.52502600	-4.41457100
H	-0.55813000	4.66459000	-3.43249700
C	-2.42975200	1.91696700	-2.31135900
H	-2.24628900	1.06573600	-1.62766700
H	-2.63571000	1.49571000	-3.31546900
H	-3.34173200	2.45003200	-1.96782900
C	1.83508700	3.40468700	1.75117900
H	2.18636400	2.42050500	1.38061300
C	2.94797300	4.42930300	1.46064700
H	3.86581600	4.17279200	2.02850700
H	2.63258000	5.44964500	1.76404200
H	3.21835500	4.46185900	0.38911600
C	1.61635100	3.28451600	3.26657600
H	2.55268700	2.95623600	3.76023100
H	0.82703900	2.54907500	3.51052900
H	1.33696800	4.25573900	3.72432200
N	-2.29503500	-0.37360800	1.12597200
N	-2.26538300	-1.49723400	-0.73564700
C	-3.06652800	-0.90514700	0.14268200
C	-4.55689000	-0.87427500	0.11232200
C	-5.28848000	-1.70833000	0.98301800
H	-4.75004200	-2.37778800	1.66945700
C	-6.68963500	-1.70512500	0.95309000
H	-7.25104100	-2.36588400	1.63067100
C	-7.37393900	-0.86395300	0.06069200
H	-8.47418800	-0.85905900	0.04062500
C	-6.64976800	-0.03138300	-0.80717700
H	-7.18025400	0.63121900	-1.50750000
C	-5.24762800	-0.03881200	-0.78727100
H	-4.68007100	0.62057000	-1.45746400
C	-2.58074000	-2.41159900	-1.84917600
C	-1.29113400	-3.19623000	-2.13673000
H	-0.98023700	-3.79002500	-1.25533200
H	-0.47724700	-2.49620800	-2.40339500
H	-1.44523100	-3.88521100	-2.99068900
C	-3.70075100	-3.40923500	-1.49340100
H	-3.48132400	-3.91722000	-0.53226700
H	-3.76482600	-4.18439900	-2.28321600
H	-4.69255200	-2.92662100	-1.41696200
C	-2.95718400	-1.60856700	-3.11014200
H	-3.87949500	-1.01706100	-2.95029900
H	-3.13755100	-2.29893200	-3.95908300
H	-2.13058500	-0.92565200	-3.38572700
C	-2.64150100	0.47040500	2.29042000
C	-3.03098900	-0.42884800	3.48065800
H	-2.21029200	-1.14241300	3.69015400
H	-3.95836400	-0.99658100	3.27165300
H	-3.20503300	0.18498000	4.38762300
C	-3.76495300	1.47236500	1.96493400
H	-3.86737400	2.18479900	2.80769400
H	-4.74597600	0.98538800	1.81631500
H	-3.51429900	2.05693400	1.05670300
C	-1.37838000	1.26531300	2.65830900
H	-0.54824200	0.58127900	2.91766900

H	-1.58865500	1.92114900	3.52594500
H	-1.06751800	1.91015600	1.81502700
O	0.18451900	-1.33919600	2.01721800
C	-0.01715100	-2.59918200	1.27265300
H	0.94634500	-3.04339800	0.93780500
C	-0.88396200	-3.61913100	1.99054500
H	-1.03874700	-4.48411500	1.30702800
H	-1.89379600	-3.19490700	2.18836200
C	-0.25572900	-4.08924000	3.30668600
H	-0.09832200	-3.22570400	3.98444600
H	-0.89177400	-4.83111900	3.83185300
H	0.73736300	-4.55172700	3.12706000

Cartesian coordinates of the optimized geometry of **S2** at PBE-D3BJ/def2-SVP level of theory:

Ga	1.40554200	-0.25211200	-0.44459600
Cl	0.48966500	-1.47219400	-2.10879600
Si	-2.12870600	-1.12455000	1.88897600
O	-0.84791300	-0.26400100	1.09869800
N	1.50534200	1.55692000	-1.26895000
N	3.34476400	-0.63436700	-0.66996000
N	-3.19946100	-1.73731900	0.37296200
N	-3.54847400	0.09955400	1.45825500
C	2.35356800	1.72419200	-2.30090700
C	3.40551100	0.82632000	-2.60375400
H	3.96327100	1.04627800	-3.52282800
C	3.92985400	-0.21645000	-1.80732200
C	2.18461000	2.92387000	-3.20347700
H	1.30823600	2.77202000	-3.86676200
H	3.07703500	3.07886900	-3.83548600
H	1.98134300	3.84444900	-2.62388300
C	5.18896600	-0.90250700	-2.28177700
H	5.85840100	-1.16734700	-1.44213900
H	5.74025900	-0.28397800	-3.01260600
H	4.90930100	-1.85694100	-2.77688300
C	0.56399000	2.58638000	-0.92050000
C	-0.68802300	2.67098700	-1.58625300
C	-1.57860800	3.69374500	-1.20536500
H	-2.55076800	3.77254400	-1.71571500
C	-1.25670600	4.60220700	-0.19364800
H	-1.96991400	5.39056200	0.09109000
C	-0.02671000	4.49531400	0.46679100
H	0.21581100	5.20500900	1.27014300
C	0.90213700	3.49544100	0.12400600
C	-1.11520800	1.67830800	-2.65915900
H	-0.25419200	1.01855100	-2.88391500
C	-1.54345100	2.36839600	-3.96484100
H	-2.46326300	2.97439800	-3.82786700
H	-1.76163300	1.61125900	-4.74527700
H	-0.75823800	3.04652200	-4.35712800
C	-2.23492400	0.77088400	-2.12410800
H	-1.93110500	0.25500000	-1.19344800
H	-2.49767500	-0.00212400	-2.87436200
H	-3.14755100	1.36492600	-1.90611200
C	2.24279800	3.40490700	0.84728300
H	2.50089700	2.32671200	0.89626700
C	3.37424600	4.09864400	0.06574300
H	3.13766700	5.16970000	-0.10292100
H	3.55301700	3.62563900	-0.91787800
H	4.32555900	4.04628500	0.63473900
C	2.19258900	3.93492400	2.28672400
H	1.35127100	3.49687800	2.85951900
H	2.08740700	5.03907200	2.31719900
H	3.13179500	3.68428800	2.81792800
C	4.04138700	-1.46704900	0.26897000
C	3.91928400	-2.88108700	0.21595400
C	4.59166200	-3.64662800	1.18874900

H	4.50386100	-4.74368000	1.15916500
C	5.36456100	-3.04294500	2.18496200
H	5.88245500	-3.66021800	2.93468700
C	5.47230000	-1.64731300	2.22843800
H	6.07738000	-1.17680900	3.01725800
C	4.81606600	-0.83521500	1.28588600
C	3.08507700	-3.58504000	-0.84556900
H	2.70341500	-2.81687800	-1.54660200
C	3.91638200	-4.59063200	-1.65963900
H	4.27628000	-5.43281700	-1.03257900
H	4.80727100	-4.11221600	-2.11485900
H	3.30479600	-5.02386700	-2.47715900
C	1.85405200	-4.26196000	-0.21933800
H	2.14849300	-5.07408900	0.47789400
H	1.20679600	-4.69826200	-1.00639000
H	1.23962900	-3.53445400	0.34592300
C	4.96158100	0.68257100	1.34116300
H	4.07246900	1.10528400	0.82927700
C	6.20032100	1.16424000	0.56273400
H	7.12685400	0.72643600	0.98938700
H	6.28823900	2.26901800	0.61548000
H	6.15411500	0.88476200	-0.50627500
C	4.98911600	1.23546900	2.77315800
H	4.12003400	0.88907400	3.36699100
H	4.97209800	2.34375500	2.75423200
H	5.91049900	0.93856000	3.31482200
C	-4.10693300	-0.76628400	0.59222500
C	-5.51388200	-0.72440500	0.09384700
C	-6.57185000	-1.09401500	0.95004200
H	-6.34529300	-1.43055600	1.97266500
C	-7.89818200	-1.04940300	0.49684800
H	-8.71591400	-1.34614900	1.17104300
C	-8.18164000	-0.62868500	-0.81253800
H	-9.22304200	-0.58998200	-1.16625900
C	-7.13250500	-0.25918500	-1.66923600
H	-7.34899500	0.07250500	-2.69598900
C	-5.80425500	-0.31013000	-1.22183400
H	-4.98055900	-0.01362800	-1.88641000
C	-3.32615500	-3.06136000	-0.26003700
C	-4.41548100	-3.91142800	0.42368600
H	-4.21814300	-3.98268200	1.51253500
H	-4.42427900	-4.93826500	0.00441200
H	-5.42356500	-3.47680600	0.27691700
C	-3.61325700	-2.91998700	-1.76720000
H	-4.60725500	-2.47191600	-1.95576200
H	-3.59602900	-3.91895800	-2.24782900
H	-2.83571900	-2.29209200	-2.24415200
C	-1.95385200	-3.73827400	-0.08653600
H	-1.70325200	-3.84478200	0.98962400
H	-1.16299000	-3.13728200	-0.57750200
H	-1.96070300	-4.74901500	-0.54040300
C	-3.97859700	1.39988300	1.98932900
C	-4.93736900	1.20398400	3.18231400
H	-4.47289600	0.54436900	3.94237700
H	-5.89128300	0.74557700	2.85569600
H	-5.17193100	2.17759300	3.65925500
C	-2.69867100	2.11260800	2.46866200
H	-2.94654900	3.11079400	2.88080000
H	-1.98126500	2.23423200	1.63468500
H	-2.19892100	1.52667100	3.26702000
C	-4.64913100	2.26035200	0.90309700
H	-4.85146600	3.27325000	1.30543300
H	-5.61128200	1.83157500	0.56542900
H	-3.97867600	2.36586200	0.02803300
C	0.53777200	-0.51994100	1.33181200
H	0.70448700	-1.59047000	1.61358200
C	1.10574200	0.36645100	2.44385400
H	0.82152600	1.41481200	2.21822900
H	2.21535000	0.32263800	2.39689500

C	0.64877200	-0.00115900	3.85718600
H	-0.45768700	0.01413500	3.94414100
H	0.97757300	-1.02530600	4.12988500
H	1.05928500	0.70269200	4.61079100

Cartesian coordinates of the optimized geometry of Int-**3** at PBE-D3BJ/def2-SVP level of theory:

Ga	2.06786600	-0.20320200	-0.50846300
Cl	1.02637000	0.03143000	-2.50411200
Si	-1.86747900	0.20927300	0.63356900
O	-0.20861500	0.42752200	1.05555300
N	3.39451200	1.28221400	-0.62253000
N	3.39464300	-1.64925200	-0.84889300
N	-2.71265700	1.17335600	-0.76760900
N	-2.18679000	2.14797300	1.11115400
C	4.49699200	1.12181700	-1.38019400
C	4.89313300	-0.10685100	-1.95843600
H	5.76011100	-0.05175900	-2.62867100
C	4.43440300	-1.40891600	-1.66524300
C	5.39782400	2.31033400	-1.62888700
H	6.36040000	1.98995400	-2.06516800
H	5.59035200	2.86855500	-0.69207500
H	4.91990800	3.02800700	-2.32436200
C	5.15189000	-2.57129100	-2.30993700
H	5.25028400	-3.43088200	-1.62072300
H	6.15102400	-2.27847300	-2.67957300
H	4.55305700	-2.92559400	-3.17553200
C	3.13225100	2.56419600	-0.02994700
C	2.60546300	3.62933300	-0.81182100
C	2.46119200	4.89048200	-0.20190000
H	2.06469200	5.72850300	-0.79532300
C	2.79387000	5.09564500	1.14032200
H	2.67457000	6.09128500	1.59392000
C	3.24962600	4.02134900	1.91370100
H	3.47957400	4.17883000	2.97723400
C	3.42317400	2.74372800	1.35144400
C	2.14074700	3.44222500	-2.25234600
H	2.43983100	2.42861300	-2.58526200
C	2.74509500	4.47330300	-3.22012000
H	2.39012200	5.50150500	-2.99994900
H	2.44913400	4.24037700	-4.26304800
H	3.85304900	4.49352200	-3.17896100
C	0.60366100	3.48875100	-2.31893300
H	0.14637600	2.72564400	-1.66094400
H	0.25333400	3.29195100	-3.35247600
H	0.23302400	4.48981300	-2.01246900
C	3.94941900	1.58787300	2.19337700
H	3.52028900	0.65972600	1.75997400
C	5.48058900	1.46412400	2.09091400
H	5.97364800	2.39627500	2.43669600
H	5.80997500	1.26606500	1.05264400
H	5.84895300	0.62964400	2.72236300
C	3.49952200	1.65364300	3.65872000
H	2.40444400	1.80392600	3.74038900
H	4.00042300	2.47540800	4.21112200
H	3.75609600	0.70896300	4.17880200
C	3.16329000	-2.95772600	-0.30867800
C	2.29168300	-3.86863800	-0.95988700
C	2.06067200	-5.11827400	-0.35102900
H	1.38388100	-5.83488900	-0.84147300
C	2.67052300	-5.46271300	0.85871000
H	2.47478200	-6.44396400	1.31702000
C	3.52462500	-4.55054200	1.49262200
H	3.99136400	-4.82372500	2.45010000
C	3.78229300	-3.28584900	0.93428100
C	1.61213300	-3.53933900	-2.28323500
H	1.95141500	-2.53343100	-2.60082300

C	2.00046400	-4.54166300	-3.38448500
H	1.62278900	-5.56123700	-3.16090700
H	3.10004100	-4.61633700	-3.50614200
H	1.56732100	-4.23339500	-4.35792900
C	0.08458700	-3.46567800	-2.12611000
H	-0.33550300	-4.44541200	-1.81958600
H	-0.39025000	-3.17392100	-3.08474600
H	-0.21475100	-2.71371200	-1.36998300
C	4.70500900	-2.29416800	1.64051900
H	4.36558900	-1.27997100	1.34201500
C	6.16577100	-2.43551300	1.17246400
H	6.54779600	-3.45593500	1.38405000
H	6.81539200	-1.71002200	1.70411200
H	6.27752500	-2.24776400	0.08866900
C	4.63542700	-2.37923900	3.17216400
H	3.59352100	-2.32547700	3.54336600
H	5.20376100	-1.54165400	3.62521200
H	5.08757100	-3.31703200	3.55576600
C	-2.79928200	2.32452300	-0.05837600
C	-3.53405900	3.55697800	-0.47409000
C	-4.84781600	3.75342700	-0.00043900
H	-5.30534000	2.99230500	0.64888100
C	-5.55771300	4.91305900	-0.34113800
H	-6.58264500	5.05495700	0.03353800
C	-4.96066000	5.89255400	-1.15099400
H	-5.51627500	6.80530600	-1.41409600
C	-3.65284300	5.70245000	-1.62421300
H	-3.17930900	6.46490700	-2.26099800
C	-2.94313600	4.53896200	-1.29262300
H	-1.92468200	4.38400700	-1.67337200
C	-3.39459000	0.78661600	-2.02876100
C	-4.92020700	0.69811500	-1.81931200
H	-5.15618300	-0.03920400	-1.02657000
H	-5.41147800	0.36035300	-2.75494700
H	-5.35297300	1.68091600	-1.54951900
C	-3.05577900	1.78018900	-3.15790100
H	-3.50438600	2.77670700	-2.99366900
H	-3.44735000	1.39163000	-4.11934600
H	-1.95707200	1.88904100	-3.25151700
C	-2.86809300	-0.59765900	-2.45024700
H	-3.14315400	-1.37030700	-1.71011000
H	-1.76656300	-0.58777000	-2.56311600
H	-3.32303000	-0.86972400	-3.42388700
C	-1.63194400	3.17818600	2.02084300
C	-2.70303500	4.17201400	2.51410300
H	-3.57163400	3.63474500	2.94613100
H	-3.07035600	4.83587800	1.71015600
H	-2.27019700	4.81359100	3.30806800
C	-1.01666400	2.47339400	3.24487800
H	-0.49575800	3.22319900	3.87268400
H	-0.28851900	1.71038800	2.91367200
H	-1.78787300	1.98536100	3.86800900
C	-0.49989900	3.92668900	1.29227400
H	-0.01081300	4.65226200	1.97170200
H	-0.88041100	4.48718200	0.41726800
H	0.26788000	3.20883200	0.94616200
C	0.72677600	-0.63938500	0.91671400
H	0.21148700	-1.51392000	0.44460700
C	1.24828800	-1.11221600	2.27363900
H	1.87982200	-0.33108500	2.74533700
H	1.91108400	-1.98100900	2.08130500
C	0.13734100	-1.52247500	3.24467700
H	-0.46322300	-0.64614800	3.55835500
H	-0.56356100	-2.24792500	2.78339400
H	0.55860600	-1.98365800	4.16131500
C	-3.28475800	-0.47801700	1.79680500
O	-3.11532700	-1.91007800	1.90384300
H	-4.20049000	-0.27893300	1.18908700
C	-3.53653600	0.07537800	3.19588500

H	-2.64223300	-0.10170000	3.83039300
H	-3.64810700	1.17436300	3.10282400
C	-4.77241700	-0.52580400	3.86980800
H	-4.94539200	-0.08583100	4.87314700
H	-5.68498100	-0.35118800	3.26063600
H	-4.66009000	-1.62124000	3.99454600
H	-1.64554200	-1.09969800	-0.11005400
C	-4.31556500	-4.64034400	-0.05564200
C	-5.36428600	-3.50115800	-0.38661900
O	-4.67532300	-2.31136900	0.08332300
O	-3.63904700	-4.11267900	1.11404200
C	-5.68796300	-3.33813900	-1.86840100
H	-6.13931200	-4.26596800	-2.27377700
H	-6.41623800	-2.51439300	-2.00553700
H	-4.78691400	-3.09384800	-2.45920000
C	-6.65400000	-3.62414600	0.43391000
H	-6.42889800	-3.76832500	1.50910700
H	-7.23564100	-2.68757500	0.32803600
H	-7.28423600	-4.46738200	0.08966600
C	-3.25816300	-4.80418600	-1.15367100
H	-2.43843500	-5.43776300	-0.76278500
H	-3.67195700	-5.28482500	-2.06172600
H	-2.82120500	-3.82682600	-1.43699300
C	-4.93204400	-5.99014600	0.29852600
H	-5.53486500	-6.38191500	-0.54539700
H	-4.12839000	-6.72128000	0.51446000
H	-5.57594400	-5.92038300	1.19431200
B	-3.77574900	-2.73714700	1.05794200

Cartesian coordinates of the optimized geometry of TS-**2** at PBE-D3BJ/def2-SVP level of theory:

Ga	0.94977700	-0.02928700	-0.72242900
Cl	0.85355600	-0.80284800	-2.85759100
Si	-1.03305700	-0.40130300	0.52064100
N	2.96853600	-0.20184400	-0.46374200
N	1.09291300	1.98093800	-1.05778100
C	3.77390900	0.57602100	-1.21684500
C	3.35255200	1.73866600	-1.89635500
H	4.11611400	2.22562600	-2.51566100
C	2.15665000	2.46525600	-1.72241000
C	5.23875500	0.21908200	-1.34543800
H	5.35863900	-0.71485100	-1.92889100
H	5.69958200	0.02712000	-0.35742100
H	5.79712900	1.02477300	-1.85404400
C	2.13434200	3.87913200	-2.26117500
H	2.40222300	4.60150400	-1.46313300
H	1.13140500	4.16693800	-2.62703200
H	2.87220500	3.99193000	-3.07710800
C	3.59179400	-1.23806100	0.31733800
C	3.89962900	-2.50182500	-0.25545800
C	4.53985900	-3.46437700	0.55077400
H	4.78011900	-4.44741700	0.11739900
C	4.87577000	-3.19755900	1.88035100
H	5.37999600	-3.96266000	2.49006600
C	4.55710400	-1.95261600	2.43603100
H	4.80970600	-1.75023400	3.48658700
C	3.90884900	-0.95995100	1.67943600
C	3.54309900	-2.86975700	-1.69059600
H	3.12005000	-1.97163600	-2.18214600
C	4.75836800	-3.34107500	-2.50761900
H	5.17611800	-4.29015400	-2.11207900
H	5.58053900	-2.59715600	-2.51124300
H	4.46378100	-3.52381500	-3.56109300
C	2.44474800	-3.94534500	-1.70529600
H	1.58586700	-3.63795900	-1.08257400
H	2.82281500	-4.91171700	-1.31034100
H	2.07348400	-4.11335300	-2.73638700

C	3.55538500	0.38124700	2.31789900
H	2.67712600	0.77504400	1.76466400
C	4.68641000	1.41255400	2.14996400
H	5.62502300	1.05063900	2.61902000
H	4.41311200	2.37294900	2.63436100
H	4.89563600	1.62609300	1.08420800
C	3.15131000	0.24295900	3.79247700
H	4.00443400	-0.06497400	4.43104100
H	2.33793700	-0.49886600	3.91901600
H	2.78899300	1.21249600	4.18519200
C	0.06276200	2.90049400	-0.68086500
C	-1.17407400	2.93206200	-1.38832100
C	-2.11376900	3.92162400	-1.03993800
H	-3.06650400	3.97268600	-1.58535100
C	-1.85933400	4.85056300	-0.02344300
H	-2.60745900	5.61948000	0.22216500
C	-0.66138200	4.77794100	0.69304400
H	-0.47737900	5.48337500	1.51764100
C	0.30989100	3.80516400	0.39009200
C	-1.46896100	1.95076700	-2.51671300
H	-1.02143000	0.98006800	-2.21826100
C	-0.79632700	2.35187700	-3.84073800
H	-1.13659700	3.35477100	-4.17450400
H	-1.04791400	1.61734400	-4.63212400
H	0.30548800	2.36089600	-3.75261800
C	-2.96810200	1.70848200	-2.72600200
H	-3.46626100	1.44461900	-1.77107000
H	-3.12053000	0.87468300	-3.43882900
H	-3.48353900	2.59579300	-3.14909500
C	1.55402500	3.70112100	1.26569800
H	2.26694300	3.01249200	0.77019500
C	2.27106500	5.04595800	1.46336900
H	3.22037000	4.89782100	2.01752400
H	1.65734500	5.76021300	2.05020900
H	2.51458700	5.53187900	0.49693500
C	1.17808000	3.07537800	2.62069400
H	2.06409400	3.02145600	3.28440400
H	0.77426700	2.05125500	2.49842300
H	0.40586400	3.68212000	3.13702800
N	-2.83655600	0.14719800	0.99443300
N	-2.30939500	-1.68974600	-0.07313800
C	-3.33812300	-0.94007400	0.40986300
C	-4.78799200	-1.27539600	0.32304200
C	-5.34533800	-2.21348900	1.21652800
H	-4.69616300	-2.70714200	1.95437400
C	-6.71146500	-2.51844700	1.15643300
H	-7.13786400	-3.25074400	1.85838800
C	-7.53251800	-1.89635500	0.20146600
H	-8.60428100	-2.14081400	0.15234400
C	-6.98249300	-0.96079500	-0.68882700
H	-7.62047000	-0.46989700	-1.43900700
C	-5.61733100	-0.64473400	-0.62562200
H	-5.18594500	0.09249000	-1.31802300
C	-2.39553200	-2.69420800	-1.17316400
C	-1.03955500	-3.39801500	-1.27539900
H	-0.82528900	-3.99040100	-0.36758600
H	-0.22694700	-2.66936200	-1.43694300
H	-1.04323400	-4.08336600	-2.14535300
C	-3.46521500	-3.76920000	-0.89425700
H	-3.34887100	-4.18938300	0.12440500
H	-3.32934900	-4.59514500	-1.62043300
H	-4.49812900	-3.39487000	-1.00718600
C	-2.70044300	-1.98067800	-2.50435500
H	-3.66664200	-1.44159900	-2.45642800
H	-2.76531100	-2.72103900	-3.32712100
H	-1.89191200	-1.26714600	-2.75639200
C	-3.41406100	1.07056800	1.99187600
C	-3.94350100	0.28174700	3.20535200
H	-3.15188900	-0.39036400	3.59268200

H	-4.83443700	-0.32200800	2.94718700
H	-4.23263200	0.98256500	4.01419200
C	-4.52616300	1.93621100	1.37122500
H	-4.87441900	2.68510000	2.11083700
H	-5.39967500	1.32969500	1.06556300
H	-4.13912700	2.48180200	0.48794700
C	-2.25858100	1.97429800	2.44986300
H	-1.47010500	1.37120600	2.93943100
H	-2.62776400	2.73158500	3.16888600
H	-1.81239000	2.50637900	1.58894000
O	-0.42137600	-0.64832700	2.08636600
C	0.55765300	-1.52039200	1.43656700
H	1.57319700	-1.17480800	1.70513200
C	0.39362600	-2.97490400	1.82606400
H	1.05469300	-3.59158600	1.17899900
H	-0.64844900	-3.30030100	1.61862000
C	0.72734600	-3.23301000	3.30305700
H	0.55863600	-4.29284900	3.58830500
H	1.78983100	-2.99160800	3.51490200
H	0.09701400	-2.59576800	3.95631500

Cartesian coordinates of the optimized geometry of TS-**7** at PBE-D3BJ/def2-SVP level of theory:

Ga	2.33140400	-0.24044400	-0.50638000
Cl	1.42700400	0.31625900	-2.49488600
Si	-1.59573600	0.69641600	0.50970700
O	0.08065600	0.74785000	0.88114400
N	3.90207800	0.97543400	-0.36965600
N	3.40740300	-1.85040400	-1.00380300
N	-2.21416300	1.58902400	-1.01115000
N	-1.62400000	2.70539600	0.76842500
C	4.94879300	0.77862200	-1.18956800
C	5.17726500	-0.43198100	-1.88309500
H	6.05297800	-0.43571000	-2.54437700
C	4.53030700	-1.67773600	-1.72801000
C	5.94017300	1.89621800	-1.41675900
H	6.93932200	1.50043500	-1.67480200
H	6.02172300	2.56594500	-0.54116700
H	5.59268900	2.51703800	-2.26916200
C	5.14914500	-2.86950700	-2.42105200
H	5.18522700	-3.75522400	-1.75822100
H	6.16901900	-2.63964000	-2.77696300
H	4.53118500	-3.15913100	-3.29548000
C	3.80434100	2.14490600	0.45974000
C	3.22981500	3.33977800	-0.04857500
C	3.08676400	4.43440300	0.82665400
H	2.63579400	5.36537300	0.45004600
C	3.49967300	4.35855100	2.15988600
H	3.36992300	5.22251900	2.82904700
C	4.07842400	3.17770800	2.64312500
H	4.40529300	3.12755600	3.69144000
C	4.24491100	2.05465900	1.81256300
C	2.76689400	3.46830800	-1.49377800
H	2.99323100	2.51576900	-2.01098700
C	3.49621000	4.60424200	-2.23180200
H	3.24590300	5.59560800	-1.79988100
H	3.19868300	4.62409200	-3.30018000
H	4.59786300	4.49304900	-2.18398700
C	1.24471600	3.65865400	-1.57319300
H	0.70642600	2.88642900	-0.99123000
H	0.90278700	3.58950600	-2.62567700
H	0.95401800	4.65555000	-1.18151800
C	4.91544500	0.78734000	2.33584300
H	4.44180900	-0.06332700	1.80274600
C	6.41567100	0.75307300	1.98668700
H	6.94194000	1.62240700	2.43257300
H	6.59195100	0.77079100	0.89555700

H	6.88473700	-0.17072200	2.38405800
C	4.72538800	0.56327700	3.84177700
H	3.66169100	0.62969400	4.14218600
H	5.29723100	1.29971300	4.44319500
H	5.09922000	-0.44101300	4.12619500
C	2.92820700	-3.17666900	-0.73254600
C	2.11052900	-3.86522700	-1.67076200
C	1.70265300	-5.17791700	-1.36064100
H	1.07437700	-5.72537100	-2.07973000
C	2.07525300	-5.79566600	-0.16292300
H	1.75287600	-6.82587900	0.05211600
C	2.85329000	-5.09418600	0.76624800
H	3.13334700	-5.57837100	1.71294000
C	3.29136800	-3.78303600	0.50517200
C	1.64293900	-3.23318100	-2.97642000
H	2.15336000	-2.25594800	-3.08768100
C	1.98092200	-4.09744800	-4.20292200
H	1.41663600	-5.05294100	-4.20274100
H	3.05925900	-4.35190800	-4.24985100
H	1.71590400	-3.55968800	-5.13559100
C	0.13502200	-2.93493400	-2.92179400
H	-0.45844100	-3.86488300	-2.79429200
H	-0.19984300	-2.43846700	-3.85428400
H	-0.10457800	-2.24848200	-2.08802200
C	4.17106700	-3.04922200	1.51087300
H	3.99283500	-1.96526100	1.35162900
C	5.66890400	-3.29019100	1.24756200
H	5.91207000	-4.37098700	1.31422700
H	6.28568800	-2.75419500	1.99830100
H	5.97673000	-2.93039300	0.24758200
C	3.82077500	-3.37405100	2.96973700
H	2.73488800	-3.27272300	3.16623100
H	4.35611100	-2.68524600	3.65359800
H	4.12116300	-4.40500400	3.24830600
C	-2.21273800	2.81411400	-0.41503400
C	-2.87283900	4.04364500	-0.94871700
C	-4.18949300	4.33657300	-0.53758500
H	-4.71898600	3.63102600	0.11969300
C	-4.82456200	5.50680700	-0.97771000
H	-5.85333600	5.72324100	-0.65270000
C	-4.15206500	6.39497300	-1.83204100
H	-4.65034600	7.31382400	-2.17627500
C	-2.84278400	6.10507400	-2.24762800
H	-2.31000000	6.79678400	-2.91748200
C	-2.20497800	4.93536200	-1.81035900
H	-1.17584500	4.71570900	-2.12376800
C	-2.81597500	1.19339500	-2.31182300
C	-4.34163300	1.41698800	-2.30493100
H	-4.78122700	0.91864400	-1.41811700
H	-4.78777800	0.96857000	-3.21573500
H	-4.60646600	2.49098300	-2.29734900
C	-2.13536100	1.96343600	-3.46077100
H	-2.35134900	3.04697700	-3.42113800
H	-2.50890700	1.58556300	-4.43366200
H	-1.03830900	1.80979500	-3.42379400
C	-2.55522400	-0.30600700	-2.51931400
H	-3.14992100	-0.90947900	-1.80821400
H	-1.47712200	-0.53230200	-2.42021500
H	-2.87471000	-0.58693100	-3.54245700
C	-1.39338000	3.69492300	1.83761500
C	-2.66038800	3.86311400	2.70474100
H	-2.96860300	2.90199400	3.15896900
H	-3.50506900	4.25454400	2.10535100
H	-2.46495100	4.58108300	3.52691700
C	-0.24864300	3.15164900	2.71400100
H	-0.04978500	3.85314100	3.54851500
H	0.67619600	3.02446300	2.12129700
H	-0.50904300	2.16488800	3.14162500
C	-0.96820700	5.07431500	1.29667000

H	-0.64391700	5.71038200	2.14449800
H	-1.79032700	5.60003400	0.77807100
H	-0.11515600	4.97315000	0.59982500
C	0.97410700	-0.35356600	0.97041700
H	0.43196700	-1.30518700	0.76788200
C	1.57517200	-0.40798700	2.37699200
H	2.00607300	0.59010100	2.59901900
H	2.42472200	-1.11960200	2.38149800
C	0.58244400	-0.79862000	3.47236700
H	-0.28832300	-0.11553800	3.48794200
H	0.19050300	-1.82604300	3.31772700
H	1.05207900	-0.76802700	4.47677700
C	-3.24146200	0.46188100	1.54897800
O	-4.11622100	-0.28223300	0.71973500
H	-3.66564500	1.49213500	1.56534900
C	-3.11879300	0.02117000	3.01311100
H	-2.69634100	-1.00221500	3.07155000
H	-2.36560400	0.68845700	3.49015100
C	-4.43763000	0.09338800	3.78870800
H	-4.29275800	-0.18499600	4.85287800
H	-4.85622300	1.12252000	3.76290500
H	-5.18589600	-0.59446000	3.34904400
H	-1.50756900	-0.82145600	0.03230100
C	-6.07779700	-2.74206600	-0.52239100
C	-6.22950600	-3.13075800	1.00580600
O	-5.46313900	-2.10922500	1.65403200
O	-4.75288700	-2.19795300	-0.56690600
C	-7.66285600	-3.08168900	1.53557800
H	-8.32029300	-3.78137200	0.98007400
H	-7.67687300	-3.37386500	2.60479200
H	-8.08121900	-2.06126900	1.45894000
C	-5.60837900	-4.50252800	1.32388000
H	-4.59545500	-4.59190000	0.88665800
H	-5.51382500	-4.59964500	2.42334500
H	-6.22861100	-5.34086000	0.94815300
C	-7.05726000	-1.63248300	-0.93921200
H	-6.74722300	-1.24229800	-1.92883300
H	-8.10183200	-1.99563500	-1.01560900
H	-7.02054600	-0.79322900	-0.21759300
C	-6.17093900	-3.91676800	-1.49532600
H	-7.15600400	-4.42113200	-1.42217600
H	-6.04985100	-3.55175700	-2.53508700
H	-5.37646500	-4.66229000	-1.30412400
B	-4.45281400	-1.66666300	0.73903900
C	-2.15400200	-2.62325900	0.33977300
H	-2.42887800	-2.49040200	-0.72858600
O	-3.03404100	-2.43959700	1.22014800
C	-0.95634300	-3.45726500	0.68396000
H	-0.12540800	-3.26391900	-0.02019300
H	-0.62064800	-3.20960100	1.71008600
C	-1.37025800	-4.93996400	0.60634700
H	-0.50366200	-5.58420700	0.84712200
H	-2.19005600	-5.15702800	1.31883700
H	-1.71914500	-5.20816200	-0.41181600

Cartesian coordinates of the optimized geometry of TS-**10** at PBE-D3BJ/def2-SVP level of theory:

Ga	-1.90449800	-0.64883400	-0.57442900
Cl	-1.19992500	-0.81540500	-2.70282200
Si	2.19007500	0.61868400	0.35518800
O	0.68659700	-0.14999000	0.32087900
N	-2.55333400	-2.49324600	-0.20690100
N	-3.72617200	0.11796500	-0.87066100
N	3.02570100	0.19508000	-1.24899200
N	3.31488500	-0.85082000	0.63092900
C	-3.63045800	-2.93482700	-0.88579500
C	-4.58027300	-2.07627300	-1.47699400

H	-5.41012600	-2.57533100	-1.99371100
C	-4.69297600	-0.66917500	-1.37056600
C	-3.83509200	-4.42287600	-1.04860000
H	-3.16167000	-4.78937400	-1.85226100
H	-4.87499900	-4.66103200	-1.33554000
H	-3.56733100	-4.98340300	-0.13364000
C	-5.98069500	-0.05295300	-1.86710800
H	-6.21922400	0.88985100	-1.34120300
H	-6.82348600	-0.75971400	-1.75808700
H	-5.88439100	0.19163800	-2.94514700
C	-1.79798500	-3.38571700	0.62712600
C	-0.66496700	-4.07087700	0.11518800
C	0.07181700	-4.88836900	0.99520300
H	0.95314500	-5.42632700	0.61350800
C	-0.29677600	-5.03246800	2.33637600
H	0.29542500	-5.67367000	3.00680600
C	-1.42297400	-4.35692500	2.82476200
H	-1.70679500	-4.47375400	3.88074300
C	-2.19130700	-3.52481100	1.99094500
C	-0.23178800	-3.94810900	-1.33982000
H	-0.98562500	-3.33051700	-1.86603100
C	-0.17096200	-5.31631200	-2.03918100
H	0.61837100	-5.96508700	-1.60531700
H	0.06179200	-5.18783300	-3.11601200
H	-1.13130100	-5.86512500	-1.95902400
C	1.10799500	-3.20281000	-1.46086900
H	1.07876200	-2.22791700	-0.93697800
H	1.34432300	-3.00715800	-2.52664400
H	1.93423500	-3.80263000	-1.02556600
C	-3.41886900	-2.79913000	2.53670800
H	-3.54993900	-1.88958800	1.91407900
C	-4.70168100	-3.63696900	2.37691100
H	-4.61212700	-4.60024600	2.92099300
H	-4.92407000	-3.86323700	1.31788100
H	-5.57442100	-3.09074900	2.79083800
C	-3.26453400	-2.35367200	3.99826200
H	-2.32254100	-1.79643800	4.16646800
H	-3.27848100	-3.21599700	4.69621200
H	-4.10962800	-1.69566200	4.28616000
C	-3.91987500	1.53042800	-0.70595000
C	-3.79251700	2.41881400	-1.80950300
C	-3.96995900	3.79654200	-1.57193200
H	-3.87593400	4.49802400	-2.41502200
C	-4.25243500	4.29005300	-0.29490500
H	-4.38274700	5.37125600	-0.13564800
C	-4.36233500	3.40339300	0.78391700
H	-4.58014500	3.79565900	1.78762900
C	-4.20026200	2.01847700	0.60316200
C	-3.47461200	1.94654700	-3.22474500
H	-3.43317400	0.83934700	-3.21612600
C	-4.55428100	2.38750300	-4.22979000
H	-4.56269000	3.48954000	-4.36032000
H	-5.57228400	2.08718800	-3.91081300
H	-4.35947500	1.94031400	-5.22576500
C	-2.08813800	2.43137200	-3.68178700
H	-2.03800900	3.53969600	-3.71382000
H	-1.86496600	2.04883200	-4.69833800
H	-1.29237400	2.06224100	-3.00959600
C	-4.37379800	1.05299700	1.76909100
H	-3.73073400	0.17556500	1.54875600
C	-5.81768200	0.52503000	1.85290200
H	-6.53117300	1.35718500	2.02606800
H	-5.92094400	-0.19515300	2.69107000
H	-6.12129600	0.00379300	0.92495400
C	-3.92415300	1.62982000	3.11705200
H	-2.90682300	2.06338300	3.05927500
H	-3.90954100	0.83324500	3.88767300
H	-4.61293300	2.42037800	3.47985700
C	3.80250100	-0.70597000	-0.61347900

C	5.03292000	-1.34029200	-1.15344600
C	6.25581000	-0.65084000	-1.01439100
H	6.24920100	0.31131300	-0.47794400
C	7.42928100	-1.21805500	-1.53050300
H	8.38472200	-0.68270200	-1.42180800
C	7.39057200	-2.46431900	-2.17816600
H	8.31511300	-2.90456600	-2.58177600
C	6.17227800	-3.14983400	-2.30921400
H	6.13864800	-4.12563300	-2.81681000
C	4.99139800	-2.59068000	-1.79862800
H	4.03019900	-3.11209100	-1.91413800
C	3.16081300	0.86471100	-2.55837300
C	4.17809000	2.01864600	-2.45860600
H	3.84415800	2.76253900	-1.71002100
H	4.26510300	2.52732200	-3.44049000
H	5.17873700	1.64661500	-2.16776200
C	3.57228000	-0.13178400	-3.65603800
H	4.61643500	-0.47804100	-3.54599700
H	3.48349900	0.36491000	-4.64250500
H	2.90174200	-1.01450400	-3.65485500
C	1.76741600	1.42719000	-2.88665600
H	1.45861800	2.14969500	-2.10646900
H	1.01432900	0.61832200	-2.94980000
H	1.79504000	1.96629600	-3.85449400
C	3.78056700	-1.66878000	1.76944300
C	5.26296100	-1.40871300	2.10034600
H	5.44697700	-0.31648200	2.10338200
H	5.93524400	-1.87707300	1.35677500
H	5.50353500	-1.84182000	3.09216600
C	2.90833200	-1.25388600	2.96339400
H	3.19597300	-1.82607900	3.86648000
H	1.83976700	-1.45069600	2.75228500
H	3.04089100	-0.17566600	3.18268300
C	3.54051700	-3.16074300	1.47179200
H	3.84683900	-3.77055800	2.34574900
H	4.13611600	-3.49644900	0.60078400
H	2.46929400	-3.35364200	1.26955900
C	-0.63305000	0.19286900	0.74643000
H	-0.77754900	1.29623300	0.71448100
C	-0.88980200	-0.30202700	2.17538500
H	-0.56869000	-1.36371300	2.22870900
H	-1.98501000	-0.31658000	2.34720200
C	-0.23517200	0.51031200	3.29343300
H	0.85646500	0.61343700	3.15084100
H	-0.63492600	1.54099800	3.33384200
H	-0.40201200	0.02698200	4.27860500
C	0.52723500	4.29349300	1.55037700
C	0.77203400	4.43021800	-0.00837000
B	2.00628000	2.66461100	0.89080500
O	0.96680000	2.94348300	1.81877600
O	1.87341700	3.52320400	-0.24483800
H	3.24391400	2.88788200	1.41215200
C	4.62765900	2.69097600	1.26481800
O	4.80149300	1.47172300	0.93554000
H	4.73773800	3.49153100	0.47368000
C	5.03885400	3.19590900	2.65570300
H	6.15228000	3.24988700	2.65927400
H	4.67748900	4.24067300	2.77726300
C	4.54955000	2.31401400	3.80135000
H	4.93751500	1.28166500	3.69985000
H	4.87094700	2.70496000	4.78754100
H	3.44012900	2.25910200	3.80128100
C	1.19466300	5.82898000	-0.45974400
H	0.41123500	6.57691000	-0.22102000
H	2.14181800	6.14085000	0.01689100
H	1.34969900	5.83348500	-1.55673200
C	1.40464300	5.24436100	2.37918300
H	1.07566700	6.29836000	2.29044600
H	1.34170000	4.94586600	3.44406700

H	2.46670900	5.18030500	2.07199700
C	-0.93331100	4.42570900	1.97641800
H	-1.01656900	4.30056600	3.07458300
H	-1.33208500	5.42701500	1.71571900
H	-1.57327500	3.66534100	1.49300800
C	-0.42100600	3.95991300	-0.84588700
H	-1.29080200	4.63855800	-0.75630700
H	-0.12284000	3.92367700	-1.91055900
H	-0.75147900	2.94420700	-0.55482500

Cartesian coordinates of the optimized geometry of EtCHO at PBE0-D3BJ/6-31G* level of theory:

O	-1.81834700	-0.02850200	-0.28476000
C	-0.77758400	-0.24586100	0.28578300
H	-0.67897100	-1.11995500	0.97202500
C	0.47046600	0.58495900	0.15515100
H	0.63181900	1.08584800	1.12152700
H	0.29309000	1.35915800	-0.59745500
C	1.68876300	-0.27451000	-0.17868000
H	1.58050500	-0.74969200	-1.15910900
H	2.59870600	0.33219100	-0.19731500
H	1.83175400	-1.06706700	0.56488400

Cartesian coordinates of the optimized geometry of HBPin at PBE0-D3BJ/6-31G* level of theory:

C	0.78037100	-0.18603700	0.05230200
C	-0.78037000	-0.18604000	-0.05229900
O	-1.06999200	1.18633300	-0.40510700
O	1.06998800	1.18633900	0.40510500
B	-0.00000300	1.92912100	-0.00000100
H	-0.00000700	3.12117500	-0.00000300
C	-1.46994300	-0.44671400	1.28239300
H	-2.53626700	-0.22602400	1.17780000
H	-1.36045200	-1.48963000	1.59620400
H	-1.06594000	0.19915700	2.06845100
C	-1.35011100	-1.09636900	-1.12449900
H	-1.09015700	-2.14138200	-0.92271100
H	-2.44110200	-1.01304300	-1.13447200
H	-0.98104100	-0.82847400	-2.11690200
C	1.35012200	-1.09636200	1.12450000
H	1.09017200	-2.14137700	0.92271600
H	2.44111400	-1.01303200	1.13446500
H	0.98105900	-0.82846700	2.11690500
C	1.46993600	-0.44671100	-1.28239300
H	2.53626000	-0.22601400	-1.17780800
H	1.36045000	-1.48962800	-1.59620100
H	1.06592400	0.19915500	-2.06845000

Cartesian coordinates of the optimized geometry of **S1** at PBE0-D3BJ/6-31G* level of theory:

Ga	0.86048500	0.18920300	-0.83083600
Cl	0.75975400	-0.14425300	-3.05545400
Si	-0.68612100	-1.08869500	0.42430800
N	2.76438000	-0.24855600	-0.49722700
N	1.19155100	2.12205900	-0.81343000
C	3.68850200	0.51146300	-1.06596500
C	3.41040400	1.75374800	-1.66814700
H	4.22402800	2.20202500	-2.22587200
C	2.27674700	2.53654900	-1.48714700
C	5.12864200	0.07646800	-1.08957200
H	5.33425300	-0.44767600	-2.03023200
H	5.36553900	-0.60618900	-0.27224100
H	5.78959100	0.94483500	-1.04076800

C	2.28307900	3.92007400	-2.07711600
H	3.28666200	4.21292000	-2.39126900
H	1.89112000	4.66960000	-1.38663900
H	1.62948600	3.92440000	-2.95873000
C	3.13754400	-1.45843900	0.17438700
C	3.40564300	-2.61825100	-0.57510500
C	3.72194800	-3.79335400	0.11138700
H	3.92493600	-4.69852300	-0.45486400
C	3.76813500	-3.82370600	1.49604000
H	4.00803700	-4.74772700	2.01551100
C	3.50603600	-2.66679700	2.21972900
H	3.53983600	-2.70026200	3.30327600
C	3.19035600	-1.46749000	1.58307800
C	3.31706400	-2.65480200	-2.08713600
H	3.13809300	-1.64002600	-2.45551000
C	4.59574000	-3.18381300	-2.73908600
H	4.77024200	-4.23549100	-2.48442500
H	5.48147400	-2.62038200	-2.42721200
H	4.51604000	-3.11982300	-3.83009000
C	2.11095200	-3.49526800	-2.50265100
H	1.22210200	-3.16247800	-1.96615000
H	2.26856100	-4.55548700	-2.26964100
H	1.91904200	-3.40072500	-3.57681700
C	2.98211100	-0.20063200	2.38918300
H	2.18270000	0.36839600	1.90109500
C	4.26219000	0.64234000	2.38087700
H	5.10913600	0.05600800	2.75670200
H	4.15321300	1.52117000	3.02557700
H	4.51402600	0.99745800	1.37747000
C	2.52132700	-0.45976200	3.81990100
H	3.30808600	-0.91906600	4.43062500
H	1.63712700	-1.10216300	3.81738200
H	2.25882300	0.48982200	4.29840100
C	0.30706300	3.10396100	-0.27616700
C	-0.87841200	3.43786100	-0.95057800
C	-1.74056600	4.37018500	-0.36878800
H	-2.66756200	4.62470800	-0.87662400
C	-1.41998000	4.99417900	0.82763300
H	-2.09542500	5.72663700	1.26123900
C	-0.22550700	4.68365400	1.46456200
H	0.02435600	5.18133300	2.39708000
C	0.64925300	3.73033600	0.94283000
C	-1.22808800	2.82856000	-2.28900200
H	-0.38081900	2.22718100	-2.62952600
C	-1.49027400	3.89634500	-3.35227000
H	-2.38489300	4.48771900	-3.12420400
H	-1.64493400	3.42406900	-4.32866600
H	-0.64796800	4.59115100	-3.43930400
C	-2.42751100	1.89631100	-2.15127300
H	-2.21362900	1.08768600	-1.44630800
H	-2.67959400	1.44578200	-3.11642400
H	-3.30481600	2.44671500	-1.78740000
C	1.93311300	3.40065600	1.68215400
H	2.29664900	2.44387700	1.29456600
C	3.02204400	4.44598800	1.41983800
H	3.93064700	4.19306200	1.97841000
H	2.69215500	5.44102600	1.74207300
H	3.28913200	4.50407900	0.36144300
C	1.71581400	3.25098600	3.18806700
H	2.64714600	2.94044700	3.67235600
H	0.95054100	2.50390100	3.41234700
H	1.41561100	4.19681000	3.65282900
N	-2.23483200	-0.36429900	1.09718100
N	-2.28399900	-1.48673300	-0.73159400
C	-3.04386800	-0.87964200	0.15407000
C	-4.52527300	-0.82220400	0.17890700
C	-5.21876900	-1.63180900	1.08235200
H	-4.66445200	-2.29533400	1.73943300
C	-6.60794900	-1.60437100	1.11938000

H	-7.14091400	-2.24220500	1.81863300
C	-7.31266300	-0.76257300	0.26196500
H	-8.39818200	-0.73830300	0.29419600
C	-6.62313700	0.04596000	-0.63738600
H	-7.16833000	0.70536700	-1.30665100
C	-5.23320000	0.01438900	-0.68441900
H	-4.69449700	0.65162200	-1.37727800
C	-2.65207400	-2.35410100	-1.85358800
C	-1.39442600	-3.14187500	-2.20682800
H	-1.04981100	-3.73729000	-1.35598900
H	-0.60196200	-2.45179400	-2.50545800
H	-1.59533300	-3.81427100	-3.04745700
C	-3.77064800	-3.33581200	-1.49612100
H	-3.52567400	-3.87827500	-0.57589900
H	-3.87784100	-4.06800800	-2.30389700
H	-4.73624200	-2.84250300	-1.36362400
C	-3.03623100	-1.50445100	-3.06658000
H	-3.94887300	-0.93023300	-2.88008000
H	-3.21066300	-2.14644200	-3.93735300
H	-2.21808300	-0.81644400	-3.29767400
C	-2.50353300	0.47740800	2.26904700
C	-2.77947000	-0.41330900	3.48260100
H	-1.94494900	-1.10680800	3.62266200
H	-3.70300400	-0.98623700	3.35064500
H	-2.88700000	0.19664100	4.38682700
C	-3.65343900	1.45857400	2.03375200
H	-3.69104600	2.16186600	2.87252700
H	-4.62603300	0.96640600	1.96743900
H	-3.48410100	2.03689300	1.11871700
C	-1.23407100	1.29001800	2.51551700
H	-0.38047300	0.63413600	2.70796700
H	-1.37943000	1.94583900	3.38026700
H	-1.01525400	1.91673700	1.64784700
O	0.19509700	-1.39229700	1.89719400
C	-0.00352200	-2.62946400	1.13082900
H	0.95031300	-3.04584100	0.78884100
C	-0.84385700	-3.65997300	1.84807000
H	-1.01788400	-4.50355900	1.16465700
H	-1.83237700	-3.24290200	2.08804900
C	-0.16967700	-4.14790900	3.12622500
H	0.00139600	-3.30471300	3.80354700
H	-0.77574100	-4.89577200	3.65056900
H	0.80666000	-4.59276200	2.90058900

Cartesian coordinates of the optimized geometry of **S2** at PBE0-D3BJ/6-31G* level of theory:

Ga	1.38179600	-0.29496300	-0.51216900
Cl	0.62001500	-1.43398700	-2.27026300
Si	-2.06180000	-1.15627700	1.81246800
O	-0.78719900	-0.28405400	1.09051700
N	1.40744100	1.52754800	-1.22458100
N	3.31690600	-0.54038300	-0.68506500
N	-3.12792100	-1.72003000	0.30983200
N	-3.44216700	0.06870500	1.44003400
C	2.26090700	1.78504400	-2.21722800
C	3.35549900	0.97080500	-2.54415800
H	3.90784600	1.25259900	-3.43228100
C	3.90230100	-0.06850800	-1.78310400
C	2.07463800	3.02965500	-3.04183600
H	1.21227000	2.91223100	-3.70723600
H	2.95754100	3.22641300	-3.65238100
H	1.87192300	3.90092800	-2.41408400
C	5.20284500	-0.66341800	-2.24461200
H	5.82791800	-0.98295300	-1.40865100
H	5.76219000	0.03315900	-2.87231900
H	4.98031600	-1.55799900	-2.84078100
C	0.42105400	2.50815700	-0.88468900

C	-0.77830200	2.58792200	-1.61405200
C	-1.68934600	3.59221800	-1.28109500
H	-2.61810200	3.66991300	-1.84003600
C	-1.43275000	4.48832000	-0.25437900
H	-2.15516300	5.26281000	-0.01142300
C	-0.25502500	4.38012100	0.47246200
H	-0.06585400	5.07372000	1.28571400
C	0.68674300	3.39319600	0.17862200
C	-1.12397900	1.60218100	-2.70905200
H	-0.23071400	1.02131600	-2.95445400
C	-1.60475900	2.28248600	-3.99203800
H	-2.56846700	2.78566600	-3.85075800
H	-1.74017600	1.53471100	-4.78110700
H	-0.89267900	3.03270000	-4.35405600
C	-2.17658700	0.61689100	-2.19993300
H	-1.85556300	0.11914800	-1.28172700
H	-2.37206200	-0.15153300	-2.95588800
H	-3.11599100	1.14474900	-1.99005500
C	1.97515500	3.30344200	0.97297400
H	2.28815000	2.25428800	0.95179600
C	3.10108900	4.11705000	0.32803600
H	2.80583000	5.16638500	0.21071000
H	3.37139700	3.72309100	-0.65592500
H	4.00100900	4.08737400	0.95373300
C	1.79339600	3.71038600	2.43465500
H	0.93410400	3.20574200	2.88886700
H	1.64518700	4.79130900	2.53977400
H	2.68594900	3.44976500	3.01150700
C	4.03469000	-1.33684500	0.25674100
C	3.92779100	-2.73792900	0.22721400
C	4.57304100	-3.47509000	1.22224700
H	4.48924700	-4.55883600	1.21509400
C	5.32430400	-2.85098900	2.20678200
H	5.82345700	-3.44110100	2.97049100
C	5.44134600	-1.46685500	2.20865400
H	6.03880600	-0.98324200	2.97640900
C	4.79624100	-0.68463300	1.25034200
C	3.16509200	-3.45884800	-0.86364800
H	2.81464100	-2.71819100	-1.58693500
C	4.06695300	-4.43930500	-1.61753200
H	4.40488000	-5.25889000	-0.97253700
H	4.95860600	-3.94052600	-2.01334300
H	3.52149100	-4.88225500	-2.45822300
C	1.93106900	-4.17313700	-0.31263000
H	2.20959600	-4.95427600	0.40511000
H	1.36660300	-4.63926700	-1.12714600
H	1.26075900	-3.47251000	0.19208500
C	4.95148800	0.82520100	1.27780600
H	4.16769500	1.25178500	0.64303700
C	6.29953700	1.25650900	0.69131500
H	7.12829400	0.83734700	1.27424200
H	6.39142600	2.34881700	0.70860300
H	6.41839300	0.92735600	-0.34471400
C	4.78723900	1.40774700	2.68171000
H	3.83764500	1.11009500	3.13596500
H	4.81789500	2.50197800	2.63720000
H	5.59554000	1.09187400	3.35061600
C	-4.02481100	-0.76850800	0.58158200
C	-5.44485000	-0.72064500	0.14954700
C	-6.44954000	-1.05932500	1.05968600
H	-6.17541400	-1.37464200	2.06251200
C	-7.78613800	-1.00777700	0.67775500
H	-8.56091600	-1.28019900	1.38884300
C	-8.12841100	-0.60780800	-0.61141400
H	-9.17258000	-0.56241800	-0.90776100
C	-7.12983200	-0.26793400	-1.52059400
H	-7.39228100	0.04480400	-2.52728500
C	-5.79190400	-0.32855200	-1.14502200
H	-5.00985900	-0.06382600	-1.84973000

C	-3.29631000	-3.04722400	-0.28714900
C	-4.27738400	-3.90283000	0.51895100
H	-3.96248100	-3.94905800	1.56714900
H	-4.30792800	-4.92378100	0.12120100
H	-5.29182400	-3.49354300	0.47632100
C	-3.74570200	-2.94415400	-1.74567700
H	-4.76088100	-2.54849800	-1.83212400
H	-3.73353600	-3.93939900	-2.20363600
H	-3.06199800	-2.30000400	-2.30670100
C	-1.90438200	-3.68158900	-0.25159200
H	-1.56578300	-3.81129500	0.78357800
H	-1.18477200	-3.04697700	-0.77902200
H	-1.92094800	-4.66641600	-0.73006400
C	-3.81955400	1.38116800	1.95744000
C	-4.76834900	1.24123200	3.15174900
H	-4.33596100	0.57056400	3.90173600
H	-5.73741200	0.83904800	2.84047700
H	-4.94037100	2.21817400	3.61811600
C	-2.51394300	2.03908500	2.41361400
H	-2.70600900	3.06211600	2.75375300
H	-1.78458800	2.05955100	1.59971700
H	-2.07307500	1.48093800	3.24822800
C	-4.46007600	2.24481400	0.87073900
H	-4.62201000	3.25647500	1.25892600
H	-5.42700100	1.84923800	0.54906700
H	-3.79669800	2.31526700	0.00412300
C	0.56098800	-0.72593200	1.22373400
H	0.60000700	-1.81590100	1.39134200
C	1.28936600	-0.06739600	2.38534500
H	1.29956200	1.01805200	2.24154800
H	2.33529900	-0.39835500	2.33028700
C	0.73583200	-0.38876500	3.76686300
H	-0.29485900	-0.03694400	3.87763800
H	0.72924400	-1.46958700	3.94731500
H	1.34023700	0.08417900	4.55025200

Cartesian coordinates of the optimized geometry of Int-**3** at PBE0-D3BJ/6-31G* level of theory:

Ga	2.02075300	-0.22925000	-0.52158700
Cl	1.07887200	0.02875600	-2.52858400
Si	-1.85218800	0.25534100	0.60074600
O	-0.22315600	0.42466800	1.02456000
N	3.37966500	1.17948700	-0.61340200
N	3.28499200	-1.69794900	-0.83247900
N	-2.65359100	1.25015000	-0.76304800
N	-2.12117200	2.16644900	1.11615900
C	4.45711800	0.98966200	-1.37666900
C	4.81463200	-0.23524800	-1.95593700
H	5.66296700	-0.20864200	-2.62861200
C	4.31227300	-1.50551700	-1.65339300
C	5.39703500	2.14004500	-1.62044100
H	6.34107400	1.78177500	-2.03440100
H	5.59806600	2.68905600	-0.69649000
H	4.96126800	2.85428400	-2.32531100
C	4.99231800	-2.68905600	-2.28342600
H	5.04337800	-3.54204800	-1.60355900
H	5.99854000	-2.43516400	-2.62264200
H	4.40726300	-3.00640000	-3.15548300
C	3.17595800	2.46283800	-0.01725100
C	2.70039700	3.54241800	-0.78512900
C	2.60384700	4.79234400	-0.16957000
H	2.24455600	5.63971600	-0.74723800
C	2.94429000	4.97082500	1.16301500
H	2.86437400	5.95383600	1.61932800
C	3.36233600	3.88364400	1.91961600
H	3.60151500	4.02244800	2.96942200
C	3.48122100	2.61698900	1.34932000

C	2.24567500	3.38472100	-2.22312900
H	2.54943600	2.39702000	-2.58289900
C	2.83960700	4.44118700	-3.15786500
H	2.45783000	5.44316500	-2.93019800
H	2.56728100	4.21691800	-4.19490300
H	3.93230600	4.48741000	-3.09700500
C	0.71717900	3.43453800	-2.29135200
H	0.26210800	2.67462500	-1.65070800
H	0.37654900	3.25469400	-3.31705200
H	0.36057500	4.42336500	-1.97604900
C	3.96772400	1.43593800	2.16377800
H	3.48317400	0.54730800	1.74528400
C	5.47765100	1.23382500	2.00976000
H	6.02242500	2.13625400	2.31110900
H	5.74977200	0.99412400	0.97776400
H	5.81961000	0.40662300	2.64261100
C	3.58348500	1.52133900	3.63835500
H	2.51824500	1.74289600	3.76368000
H	4.15697700	2.29348600	4.16445200
H	3.79376500	0.56766000	4.13430600
C	3.00683900	-2.99285900	-0.29871700
C	2.10950100	-3.85508800	-0.94950300
C	1.81280400	-5.07919400	-0.34468300
H	1.10997200	-5.75174800	-0.82958900
C	2.39852100	-5.44888700	0.85596700
H	2.15481300	-6.40505600	1.31082200
C	3.29815300	-4.59171200	1.47890800
H	3.74954300	-4.88711400	2.42118200
C	3.61151300	-3.35032800	0.92656200
C	1.47919500	-3.50618300	-2.28212100
H	1.89741900	-2.55592400	-2.62619400
C	1.79701300	-4.56515900	-3.34162000
H	1.33065000	-5.52792100	-3.10185500
H	2.87510900	-4.73624400	-3.43502300
H	1.41474300	-4.24730600	-4.31807600
C	-0.03236200	-3.31750100	-2.15570300
H	-0.51699000	-4.24711900	-1.83605900
H	-0.45827500	-3.02385200	-3.12152400
H	-0.28631600	-2.53564900	-1.43486400
C	4.58368200	-2.41565400	1.62627400
H	4.30813300	-1.39359900	1.34251300
C	6.02512500	-2.64003400	1.15803000
H	6.33257900	-3.67716900	1.33667800
H	6.70967000	-1.98329300	1.70744800
H	6.14732500	-2.42653500	0.09332400
C	4.51914800	-2.50967800	3.15054900
H	3.49391500	-2.42580800	3.52358100
H	5.11117200	-1.70303700	3.59713000
H	4.93846500	-3.45341900	3.51796200
C	-2.71297000	2.38317400	-0.04191100
C	-3.40078400	3.63981000	-0.43704100
C	-4.68940800	3.87283000	0.04982900
H	-5.16216700	3.12669100	0.68229600
C	-5.35158900	5.05518000	-0.26035500
H	-6.35389000	5.22743300	0.12132500
C	-4.72767900	6.01793600	-1.04985000
H	-5.24240200	6.94473300	-1.28692600
C	-3.44317500	5.78895200	-1.53564600
H	-2.95254500	6.53498600	-2.15439200
C	-2.78228300	4.60210000	-1.23554100
H	-1.78747200	4.41527400	-1.62558800
C	-3.34581200	0.90707400	-2.01917200
C	-4.86327400	0.86410800	-1.81436700
H	-5.12038600	0.13491100	-1.03934700
H	-5.35454600	0.55618500	-2.74495300
H	-5.26053000	1.84504900	-1.53631000
C	-2.97698600	1.89540000	-3.12973700
H	-3.38850600	2.89162800	-2.95567800
H	-3.37646100	1.53308700	-4.08326600

H	-1.88815400	1.96840800	-3.22040700
C	-2.86704200	-0.47870500	-2.45566800
H	-3.19260900	-1.24800200	-1.75276200
H	-1.77637500	-0.51046100	-2.53273500
H	-3.29771900	-0.70380600	-3.43793700
C	-1.50626200	3.15926000	2.01368300
C	-2.48643100	4.24436700	2.47021900
H	-3.40190100	3.79712200	2.87354400
H	-2.76216200	4.93012700	1.66624300
H	-2.01896800	4.83436300	3.26656900
C	-0.97772600	2.43625700	3.25416400
H	-0.40462400	3.14590500	3.86087400
H	-0.32122900	1.61634100	2.95654500
H	-1.78760800	2.04357700	3.87286900
C	-0.30694200	3.78486400	1.29996100
H	0.20775000	4.48973900	1.96078600
H	-0.61683400	4.33079600	0.40399600
H	0.40200700	3.00349200	1.01182600
C	0.69448300	-0.64967200	0.88292000
H	0.17302800	-1.53133600	0.47338400
C	1.24289400	-1.07417800	2.23349000
H	1.86606800	-0.28061500	2.65883400
H	1.90057900	-1.93159700	2.05454700
C	0.15823600	-1.46986700	3.22782200
H	-0.43583000	-0.60225300	3.52846300
H	-0.53154400	-2.20286800	2.79469000
H	0.59531500	-1.90418500	4.13443300
C	-3.26422900	-0.41461300	1.74646200
O	-3.10713900	-1.84093100	1.85451100
H	-4.17719300	-0.21466300	1.16120800
C	-3.50597800	0.12643600	3.14449600
H	-2.62515600	-0.05828400	3.77072200
H	-3.62203400	1.21263700	3.06398100
C	-4.73746200	-0.48225300	3.80523600
H	-4.91135800	-0.05362000	4.79863600
H	-5.63530800	-0.30414000	3.19951900
H	-4.62047200	-1.56419100	3.91920100
H	-1.67814100	-1.02694600	-0.16792500
C	-4.39585100	-4.52843900	-0.05634100
C	-5.40498900	-3.37719500	-0.37723400
O	-4.68250700	-2.21247100	0.06036300
O	-3.69954400	-4.02022400	1.09358100
C	-5.75200700	-3.21924200	-1.84587300
H	-6.24144400	-4.12236800	-2.22699400
H	-6.44175200	-2.37868600	-1.97289800
H	-4.86328700	-3.01953000	-2.44825900
C	-6.67467500	-3.45280700	0.46425500
H	-6.43421000	-3.58339800	1.52443500
H	-7.22713400	-2.51465800	0.35387100
H	-7.32306000	-4.27647500	0.14877700
C	-3.36084500	-4.72338300	-1.15858600
H	-2.56726900	-5.37522000	-0.78079600
H	-3.79607800	-5.18495200	-2.05093100
H	-2.90461000	-3.77015300	-1.44326900
C	-5.03861600	-5.85436400	0.30662800
H	-5.64542700	-6.23283900	-0.52353500
H	-4.25963800	-6.59196700	0.52304200
H	-5.67131800	-5.76186700	1.19232100
B	-3.79225600	-2.64933000	1.02644500

Cartesian coordinates of the optimized geometry of TS-**2** at PBE0-D3BJ/6-31G* level of theory:

Ga	0.92817300	-0.01445500	-0.69644500
Cl	0.86323400	-0.75119800	-2.82107600
Si	-1.01244200	-0.44771200	0.52291000
N	2.91550100	-0.12612400	-0.43428300
N	1.01492500	1.95828600	-1.06274300

C	3.69597600	0.66041900	-1.18196500
C	3.25834000	1.78386600	-1.89011700
H	4.00385600	2.27996600	-2.49891800
C	2.04453500	2.45898400	-1.73895700
C	5.17482500	0.38012500	-1.25521500
H	5.36769800	-0.56969800	-1.76232500
H	5.61131500	0.29331000	-0.25643800
H	5.68969800	1.17434800	-1.79823200
C	1.98697200	3.86025700	-2.28894500
H	2.27022400	4.58256200	-1.51512800
H	0.98425800	4.12685500	-2.62890600
H	2.69103600	3.96676000	-3.11802800
C	3.58336200	-1.15207200	0.31691200
C	3.92854300	-2.37133000	-0.29707900
C	4.59586200	-3.33585200	0.46104100
H	4.85402300	-4.28422600	-0.00232900
C	4.94372800	-3.10410700	1.78297300
H	5.47315000	-3.86323600	2.35232900
C	4.60937000	-1.89350300	2.37270700
H	4.88379100	-1.70888700	3.40774400
C	3.91833100	-0.90930300	1.66500200
C	3.58648200	-2.68379500	-1.73911000
H	3.23679300	-1.76697100	-2.22116000
C	4.77939100	-3.21111600	-2.53876500
H	5.10842100	-4.19362100	-2.18117400
H	5.64308000	-2.53854200	-2.49106400
H	4.49810900	-3.32431700	-3.59152300
C	2.43110900	-3.68149600	-1.78688300
H	1.59892600	-3.31854000	-1.18319100
H	2.73884800	-4.65854200	-1.39350100
H	2.07466100	-3.81489700	-2.81377600
C	3.55269400	0.38474700	2.36714600
H	2.87243100	0.93356600	1.70821800
C	4.78742600	1.26274900	2.58596400
H	5.51917400	0.76076800	3.22995400
H	4.50758900	2.20683600	3.06864700
H	5.28271600	1.50441600	1.63975900
C	2.82303100	0.12523200	3.68805400
H	3.42157800	-0.49010000	4.36808900
H	1.86770400	-0.38068500	3.51965500
H	2.61611300	1.06854900	4.20291200
C	-0.01628800	2.86280100	-0.67713900
C	-1.25152800	2.88670500	-1.35816600
C	-2.18558000	3.85639900	-0.98659000
H	-3.13745800	3.90511200	-1.50507600
C	-1.92451800	4.76850300	0.02875200
H	-2.66820300	5.51514700	0.29389500
C	-0.72003500	4.70786100	0.71203800
H	-0.52804700	5.40149200	1.52606900
C	0.24680300	3.75834700	0.37737900
C	-1.55698100	1.90426000	-2.47300400
H	-1.21358900	0.92138700	-2.13035300
C	-0.79166600	2.21303200	-3.76131700
H	-1.01594800	3.22555300	-4.11941400
H	-1.08170500	1.50191400	-4.54239400
H	0.28518100	2.10718000	-3.62299300
C	-3.04779000	1.78071400	-2.76940900
H	-3.62033600	1.58532700	-1.85529000
H	-3.21662700	0.95346200	-3.46542700
H	-3.45067500	2.68660900	-3.23808200
C	1.51481500	3.66240900	1.20225300
H	2.23767300	3.04665300	0.65769000
C	2.16741400	5.01750800	1.47177300
H	3.13358800	4.87499400	1.96856200
H	1.55347200	5.64292600	2.12974400
H	2.34115300	5.57875100	0.54717000
C	1.20064000	2.93870900	2.51181800
H	2.09285100	2.88309300	3.14367700
H	0.84831000	1.92102900	2.31556000

H	0.41932800	3.46465000	3.07251600
N	-2.76125100	0.09179200	1.04136800
N	-2.28761100	-1.69159000	-0.09262300
C	-3.28763300	-0.95603700	0.43493000
C	-4.73425800	-1.27907300	0.37988400
C	-5.25543400	-2.23883800	1.25091600
H	-4.59148900	-2.74500800	1.94559000
C	-6.61017900	-2.54648100	1.21723900
H	-7.00993200	-3.29261700	1.89777400
C	-7.44997700	-1.90532300	0.30927400
H	-8.50752700	-2.15142300	0.27941200
C	-6.93272300	-0.94754400	-0.55846200
H	-7.58383600	-0.44393700	-1.26709500
C	-5.57901900	-0.62787100	-0.51981700
H	-5.17449500	0.12363900	-1.19090500
C	-2.41726300	-2.62114300	-1.24136700
C	-1.09758300	-3.36095000	-1.40540700
H	-0.88694500	-3.99659100	-0.54291000
H	-0.27533000	-2.66306000	-1.55248200
H	-1.14954500	-3.99520300	-2.29632300
C	-3.51303900	-3.66979400	-1.02672700
H	-3.40931800	-4.14790100	-0.04709800
H	-3.39951100	-4.44372400	-1.79328100
H	-4.52230400	-3.26463800	-1.11539300
C	-2.69434500	-1.81184000	-2.50911400
H	-3.64230600	-1.26978200	-2.43084900
H	-2.75278500	-2.47322400	-3.38049000
H	-1.88033400	-1.10121700	-2.67515200
C	-3.30859900	0.98586000	2.06836400
C	-3.80970900	0.17914500	3.26850000
H	-3.02225000	-0.49656000	3.61860100
H	-4.69952500	-0.40666000	3.02137200
H	-4.07159100	0.85843600	4.08721800
C	-4.41841100	1.87015100	1.49837800
H	-4.73272300	2.59691500	2.25570200
H	-5.29582800	1.28699700	1.20686600
H	-4.04935800	2.42292000	0.62852000
C	-2.14115100	1.86309700	2.51133400
H	-1.35839900	1.25228500	2.97043900
H	-2.48344300	2.60410600	3.24108100
H	-1.71519400	2.39531200	1.65672100
O	-0.36251300	-0.80253900	2.01553000
C	0.61687100	-1.59117000	1.26396200
H	1.60247300	-1.23329400	1.56089900
C	0.50147200	-3.05996500	1.58657000
H	1.13502900	-3.63109500	0.89715800
H	-0.53220400	-3.38695400	1.41937600
C	0.90361600	-3.37453700	3.02721100
H	0.76830700	-4.43748300	3.26295400
H	1.95684900	-3.12351500	3.19767500
H	0.29697200	-2.78989800	3.72728700

Cartesian coordinates of the optimized geometry of TS-**7** at PBE0-D3BJ/6-31G* level of theory:

Ga	2.28441600	-0.28652700	-0.50536700
Cl	1.45223700	0.28465900	-2.47968100
Si	-1.56665700	0.70051200	0.49586700
O	0.07304900	0.68929600	0.85024600
N	3.85657600	0.86867100	-0.32869800
N	3.31258000	-1.88657800	-0.99988700
N	-2.14053400	1.56566500	-1.02296500
N	-1.53788500	2.67061100	0.73060200
C	4.88983100	0.66850500	-1.14421200
C	5.10564400	-0.52262400	-1.84930500
H	5.97245600	-0.53304000	-2.49884700
C	4.42969500	-1.74041700	-1.71074500
C	5.91258100	1.75427800	-1.34258400

H	6.89761500	1.32850200	-1.54574600
H	5.97815300	2.42475000	-0.48430600
H	5.62408200	2.35845100	-2.21109600
C	5.03813100	-2.94029500	-2.38376700
H	5.05855700	-3.80670200	-1.71752100
H	6.05375900	-2.72468300	-2.71992100
H	4.43915900	-3.22428000	-3.25595900
C	3.77726500	2.04641300	0.47999800
C	3.24467300	3.23325800	-0.05308100
C	3.11644300	4.33875100	0.79140000
H	2.69625900	5.26022300	0.39686900
C	3.51280500	4.27947900	2.11852800
H	3.39699700	5.14701500	2.76227200
C	4.06464600	3.10779300	2.62234200
H	4.38406000	3.07340600	3.65915800
C	4.21216900	1.97575900	1.82060800
C	2.82039200	3.34896300	-1.50219700
H	3.06611700	2.41525900	-2.01426900
C	3.55474900	4.48601600	-2.21753700
H	3.28079800	5.46335800	-1.80341900
H	3.29132500	4.49348700	-3.28118000
H	4.64232000	4.38860900	-2.13504200
C	1.30939900	3.54052900	-1.62010400
H	0.76066000	2.79592300	-1.03750300
H	0.99936400	3.44558100	-2.66669400
H	1.02477100	4.53878800	-1.26726300
C	4.87655800	0.72421100	2.36591100
H	4.43531000	-0.13215400	1.84584200
C	6.37655500	0.71033300	2.05250600
H	6.86833400	1.59599100	2.47146100
H	6.56819500	0.69036700	0.97696400
H	6.84758500	-0.17805600	2.48933200
C	4.66348000	0.52196700	3.86481800
H	3.60767200	0.59318500	4.14292300
H	5.22236300	1.25571800	4.45699600
H	5.02762900	-0.46824400	4.15981000
C	2.81062300	-3.19788800	-0.73549200
C	2.02432800	-3.87892500	-1.68293000
C	1.59142100	-5.17081600	-1.37279500
H	0.98428200	-5.71193200	-2.09334400
C	1.91674800	-5.77240000	-0.16597500
H	1.57650200	-6.78182100	0.04928400
C	2.67002700	-5.07594700	0.77086500
H	2.91111800	-5.54586400	1.71950000
C	3.12658600	-3.78510800	0.50702500
C	1.59828700	-3.24748300	-2.99218200
H	2.16064600	-2.31893800	-3.13052400
C	1.86498400	-4.14962500	-4.19852100
H	1.23516500	-5.04639600	-4.18335000
H	2.90827000	-4.48142600	-4.24182400
H	1.64039500	-3.60982100	-5.12468100
C	0.11841900	-2.86632100	-2.92540200
H	-0.51321300	-3.75435000	-2.79666900
H	-0.19052500	-2.35319700	-3.84190100
H	-0.06807700	-2.18274600	-2.09350200
C	3.98152800	-3.04194000	1.51328800
H	3.79255900	-1.97712900	1.34523400
C	5.47704300	-3.26339800	1.27212500
H	5.72544700	-4.33011600	1.31822700
H	6.06467100	-2.74479700	2.03886700
H	5.79198700	-2.87696400	0.29907000
C	3.62284000	-3.37090500	2.96064700
H	2.54431400	-3.30415600	3.13753200
H	4.12196400	-2.67141500	3.63981500
H	3.94980300	-4.37919200	3.23959100
C	-2.11496400	2.78732700	-0.44206200
C	-2.74274000	4.02357700	-0.97696700
C	-4.02383600	4.35852200	-0.53125500
H	-4.54914700	3.68418200	0.13930300

C	-4.62786100	5.53529700	-0.96104700
H	-5.62608800	5.78531700	-0.61350600
C	-3.95586700	6.38583700	-1.83501900
H	-4.42668800	7.30626000	-2.16852900
C	-2.68038200	6.05218900	-2.28276000
H	-2.15177700	6.71168500	-2.96519300
C	-2.07460700	4.87451600	-1.85779500
H	-1.07624500	4.61973600	-2.19627100
C	-2.76150600	1.18574400	-2.30821100
C	-4.26981100	1.44923700	-2.29650600
H	-4.71370400	0.97514300	-1.41546900
H	-4.72205500	1.00939000	-3.19279600
H	-4.50267500	2.51729700	-2.29722300
C	-2.06995100	1.91931800	-3.46061400
H	-2.26177500	2.99433000	-3.43767700
H	-2.45051000	1.53788300	-4.41446100
H	-0.98950100	1.74479900	-3.42352200
C	-2.54616100	-0.31143800	-2.50058100
H	-3.17344600	-0.88012600	-1.81032900
H	-1.49092500	-0.56570100	-2.37166000
H	-2.84236200	-0.58478000	-3.51896300
C	-1.25148600	3.65823600	1.77633000
C	-2.47523800	3.86194700	2.67710200
H	-2.79775500	2.92038000	3.13004500
H	-3.31321800	4.28260600	2.11259900
H	-2.22865100	4.55905200	3.48599600
C	-0.09770100	3.08987400	2.60626400
H	0.19356500	3.81010000	3.37821500
H	0.76379300	2.87205500	1.97117500
H	-0.39160100	2.15846300	3.09792400
C	-0.81263800	5.01622700	1.22243600
H	-0.47028600	5.63942500	2.05578800
H	-1.62239400	5.54850200	0.71965200
H	0.02056800	4.89743200	0.52593200
C	0.96039700	-0.41046100	0.95864700
H	0.41912600	-1.35328700	0.82545500
C	1.57767300	-0.39559600	2.34816500
H	2.00895200	0.59795200	2.51167200
H	2.41129100	-1.10238700	2.37861300
C	0.60192100	-0.73398600	3.46570900
H	-0.26264400	-0.06600400	3.45398100
H	0.22519700	-1.75950800	3.36649800
H	1.07732700	-0.65004000	4.44969200
C	-3.19374200	0.51134200	1.52887100
O	-4.09957500	-0.20018900	0.71822000
H	-3.58368600	1.54097600	1.54361000
C	-3.09308400	0.08064200	2.99042800
H	-2.72892000	-0.94879500	3.06254300
H	-2.33015500	0.71331500	3.46826800
C	-4.41363600	0.22364900	3.73980300
H	-4.29971300	-0.05636100	4.79351100
H	-4.76887800	1.26203600	3.70477500
H	-5.17713300	-0.41765900	3.29199100
H	-1.53742900	-0.86331400	0.05669600
C	-6.08500100	-2.59394100	-0.46523900
C	-6.21153600	-2.99550300	1.04014800
O	-5.41641200	-2.01189600	1.68254200
O	-4.75045300	-2.11458500	-0.53898200
C	-7.62369800	-2.92383100	1.59948000
H	-8.29923300	-3.59824800	1.06006600
H	-7.61745200	-3.21997300	2.65394900
H	-8.01690400	-1.90627000	1.53803700
C	-5.61684000	-4.37644700	1.32262400
H	-4.62612700	-4.47297900	0.86922300
H	-5.50735500	-4.49345700	2.40546200
H	-6.25389000	-5.18501000	0.94674100
C	-7.01927900	-1.44162600	-0.83480200
H	-6.73082900	-1.06133500	-1.82019300
H	-8.06906800	-1.75320800	-0.87514700

H	-6.91651800	-0.62502500	-0.11364400
C	-6.25579600	-3.73899800	-1.45013400
H	-7.25222200	-4.18823900	-1.36637000
H	-6.13666800	-3.36526400	-2.47286500
H	-5.50563700	-4.51709300	-1.28595500
B	-4.40657200	-1.59303900	0.75933500
C	-2.16012700	-2.46955700	0.31171200
H	-2.46194400	-2.40676800	-0.73756000
O	-3.04252000	-2.32534400	1.20034900
C	-0.98538900	-3.32787700	0.66133500
H	-0.15122700	-3.17167800	-0.02593400
H	-0.65615500	-3.09548400	1.67813900
C	-1.45209900	-4.78654100	0.58855100
H	-0.62047600	-5.45062800	0.83609600
H	-2.27263800	-4.96388600	1.28935300
H	-1.79890900	-5.04177200	-0.41921200

Cartesian coordinates of the optimized geometry of TS-**10** at PBE0-D3BJ/6-31G* level of theory:

Ga	-1.88403700	-0.61295200	-0.60217700
Cl	-1.23817500	-0.84839900	-2.71080100
Si	2.16152000	0.55552800	0.34431300
O	0.66468700	-0.15264200	0.26257500
N	-2.59864600	-2.39360800	-0.21593300
N	-3.64619000	0.19581600	-0.90478000
N	2.98574600	0.15467300	-1.23257300
N	3.25441200	-0.90671500	0.61534500
C	-3.65767700	-2.80848000	-0.91433100
C	-4.56667400	-1.94077000	-1.52411900
H	-5.38790000	-2.40776600	-2.05447200
C	-4.62447400	-0.54259800	-1.41636200
C	-3.90626500	-4.28347700	-1.06951500
H	-3.29111300	-4.65280700	-1.89995400
H	-4.95249200	-4.48773700	-1.30555000
H	-3.61779900	-4.84856600	-0.18140800
C	-5.88646900	0.11854300	-1.89892500
H	-6.13716600	0.99739300	-1.30063600
H	-6.72184400	-0.58404900	-1.87509400
H	-5.75765400	0.45593900	-2.93336100
C	-1.89334900	-3.29976900	0.63439200
C	-0.76314300	-3.98566300	0.15790600
C	-0.04949200	-4.78680800	1.05276500
H	0.83183400	-5.31704900	0.70110800
C	-0.45138900	-4.92434200	2.37312200
H	0.11838200	-5.54970800	3.05497600
C	-1.59264900	-4.26812300	2.81870900
H	-1.90810700	-4.39034100	3.85066400
C	-2.33135400	-3.44569900	1.96849800
C	-0.32364500	-3.90048900	-1.28869200
H	-1.06800400	-3.32106400	-1.84016800
C	-0.25283200	-5.28712100	-1.93293100
H	0.53173000	-5.90476800	-1.48014000
H	-0.02398800	-5.19340800	-3.00045200
H	-1.19919000	-5.83011800	-1.83257600
C	1.01231100	-3.17093300	-1.43128900
H	0.98819200	-2.18663500	-0.95663000
H	1.24818700	-3.02888200	-2.49217600
H	1.82053700	-3.75395300	-0.97284000
C	-3.58088400	-2.74778200	2.47668100
H	-3.75818600	-1.87785800	1.83440900
C	-4.81583800	-3.64768400	2.35772200
H	-4.67517800	-4.57502700	2.92548900
H	-5.02970000	-3.91492800	1.31984900
H	-5.69864800	-3.13524400	2.75774700
C	-3.44737000	-2.25485200	3.91819100
H	-2.53140300	-1.67625400	4.07120500
H	-3.44371300	-3.08643900	4.63205800

H	-4.30218600	-1.61903400	4.17499700
C	-3.80638500	1.60292800	-0.72518400
C	-3.67094400	2.49207300	-1.80695900
C	-3.83163300	3.85731900	-1.55537100
H	-3.73056000	4.55991100	-2.37825900
C	-4.10236800	4.33229900	-0.28083700
H	-4.22322600	5.39864900	-0.11012800
C	-4.21148100	3.44072400	0.77987600
H	-4.41194200	3.81862800	1.77775500
C	-4.05817500	2.06986300	0.58077600
C	-3.33778000	2.02959600	-3.21175700
H	-3.36301500	0.93572800	-3.23271000
C	-4.33724600	2.55588000	-4.24517000
H	-4.26594400	3.64416000	-4.35454200
H	-5.37299600	2.32186600	-3.97664300
H	-4.12990100	2.11522700	-5.22649800
C	-1.91579000	2.44555800	-3.59414800
H	-1.82015300	3.53766100	-3.62434000
H	-1.65910700	2.05031700	-4.58302700
H	-1.18575900	2.05472900	-2.88262900
C	-4.21377200	1.08757500	1.72365800
H	-3.57035800	0.23480200	1.48422900
C	-5.64467800	0.54982100	1.80686200
H	-6.35864800	1.36820700	1.95575600
H	-5.74362800	-0.14427200	2.65000800
H	-5.92609900	0.00972300	0.89830600
C	-3.77027000	1.65008700	3.06989100
H	-2.77708300	2.10428700	3.00797300
H	-3.73074200	0.84970600	3.81620700
H	-4.46929400	2.40728000	3.44347000
C	3.74780400	-0.75034200	-0.61339000
C	4.96346000	-1.38884000	-1.16055400
C	6.17738900	-0.70621000	-1.04340300
H	6.18450900	0.24635200	-0.51892700
C	7.33284200	-1.27909800	-1.56327500
H	8.27965300	-0.75439400	-1.47160900
C	7.28074500	-2.52106000	-2.19313000
H	8.18689900	-2.96329300	-2.59792100
C	6.06872600	-3.19768900	-2.30309700
H	6.02561500	-4.16514800	-2.79519700
C	4.90617900	-2.63344200	-1.78719900
H	3.95283800	-3.14425800	-1.88622900
C	3.09789700	0.79953900	-2.54485000
C	4.17347200	1.88711300	-2.51006000
H	3.94174300	2.62011600	-1.73253800
H	4.20860800	2.40264100	-3.47681100
H	5.16070700	1.46198700	-2.30962000
C	3.38740800	-0.22490700	-3.64157400
H	4.39399700	-0.64287100	-3.56796200
H	3.29842400	0.26602800	-4.61634000
H	2.65912000	-1.04225300	-3.60410700
C	1.73236600	1.43442000	-2.80279800
H	1.53722700	2.21536100	-2.06145600
H	0.94062200	0.68185900	-2.75235900
H	1.71651000	1.89668300	-3.79560400
C	3.73459700	-1.72163200	1.73852800
C	5.21812000	-1.49098800	2.03727400
H	5.42849800	-0.41798300	2.02558200
H	5.85899600	-1.98296800	1.30070000
H	5.45815700	-1.91055900	3.02087600
C	2.89980400	-1.29460000	2.94130000
H	3.19351600	-1.86710400	3.82653900
H	1.83709400	-1.47502500	2.75394400
H	3.05276600	-0.23196200	3.15683500
C	3.46471200	-3.20035000	1.45469700
H	3.78479200	-3.80354000	2.31201300
H	4.02252400	-3.54258800	0.57766900
H	2.39716100	-3.37188700	1.28860200
C	-0.64029800	0.18971000	0.72008300

H	-0.75608400	1.27957200	0.77144100
C	-0.86165900	-0.39243500	2.11186300
H	-0.51043900	-1.43033200	2.08459100
H	-1.93688400	-0.45093400	2.31121400
C	-0.20439900	0.37571500	3.25023300
H	0.85862100	0.54885200	3.07185100
H	-0.65451600	1.36407600	3.37237400
H	-0.30273900	-0.17190400	4.19543800
C	0.60300100	4.21561500	1.61644100
C	0.87595600	4.39632100	0.08606800
B	2.01532200	2.56887500	0.93145000
O	0.97473900	2.85218200	1.84302300
O	1.93459600	3.46586600	-0.16805700
H	3.23127900	2.77158200	1.48974700
C	4.57087200	2.58963100	1.25914000
O	4.74105900	1.38063000	0.90857500
H	4.64384300	3.38331000	0.48212000
C	5.05690500	3.07055900	2.62375600
H	6.15665000	3.07482800	2.59001500
H	4.74068000	4.11241100	2.76701700
C	4.56903400	2.20104900	3.77019000
H	4.91501200	1.17095000	3.64830700
H	4.92669600	2.57002400	4.73745500
H	3.47183300	2.19073100	3.79565500
C	1.35586900	5.78582400	-0.30615400
H	0.60028100	6.54427900	-0.07024800
H	2.28667600	6.04536000	0.20316900
H	1.54241300	5.81530900	-1.38437300
C	1.49909800	5.09170500	2.49015200
H	1.22423100	6.14977800	2.42761000
H	1.39803100	4.76632200	3.53025500
H	2.55087400	4.98756400	2.20635000
C	-0.84876000	4.39215300	2.02479800
H	-0.95060200	4.21604400	3.10113300
H	-1.19522000	5.41025800	1.81296200
H	-1.50135300	3.69246500	1.49812100
C	-0.31265600	4.01057300	-0.78572500
H	-1.15163800	4.70280600	-0.66730400
H	-0.00197600	4.02835900	-1.83429100
H	-0.67408300	3.00178900	-0.56516400

V. References

(1) G. M. Sheldrick, *Acta Crystallogr.*, 1990, **A46**, 467–473.

(2) G. M. Sheldrick, SHELXL-2017, Program for the Refinement of Crystal Structures University of Göttingen, 2017. See also: G. M. Sheldrick, *Acta Cryst.*, 2015, **C71**, 3–8.

(3) C. B. Hübschle, G. M. Sheldrick and B. Dittrich, *J. Appl. Cryst.*, 2011, **44**, 1281–1284.
